# A revision of the Australian digger wasps in the genus *Sphex* (Hymenoptera, Sphecidae)

**DOI:** 10.3897/zookeys.521.5995

**Published:** 2015-09-17

**Authors:** Thorleif H. Dörfel, Michael Ohl

**Affiliations:** 1 Museum für Naturkunde, Leibniz-Institut für Evolutions- und Biodiversitätsforschung, Invalidenstraße 43, 10115 Berlin, Germany

**Keywords:** Systematics, *Sphex*, Australia, monograph

## Abstract

The Australian species of the sphecid wasp genus *Sphex* are revised. Thirty-five species are recognized, of which 11 are new: *Sphex argentatissimus*, *Sphex brevipetiolus*, *Sphex caelebs*, *Sphex corporosus*, *Sphex flammeus*, *Sphex fortunatus*, *Sphex gracilis*, *Sphex imporcatus*, *Sphex jucundus*, *Sphex latilobus* and *Sphex pretiosus*. A dichotomous key covering all Australian species of the genus has been generated. The geographic distribution of all species is discussed based on all available locality records in relation to the Australian climate zones.

## Introduction

*Sphex* is a cosmopolitan genus that currently encompasses 118 species worldwide (Pulawski 2014). Most species are handsome, large and often colorful and attract the attention of collectors. The species are geographically distributed as follows (some species occur in more than one geographic area): Palearctic: 19, Nearctic: 13, Neotropic: 31, Afrotropic: 30, Indomalaya: 22 and Australasia + Oceania: 34 (Bohart and Menke 1976; Pulawski 2014).

Most species of *Sphex* are gregarious and nest in large groups of several individuals. Ground nests contain 3-10 separate cells which are built in side branches of the main tunnel, usually approximately 10-15 cm deep, but sometimes deeper depending on species and soil type (Kazenas 2001). Females travel up to 2.5 km from the nest when searching for prey (Kazenas 2001). The number of prey items per cell varies from 3 or 4 to 6 or 7. Many species prey on tettigoniids, a few on gryllacridids and some on gryllids (Bohart and Menke 1976; Evans et al. 1982; Kazenas 2001).

The number of valid Australian species ranges from 24 (Cardale 1985) to 26 (Pulawski 2014). The aim of this study is to revise the genus in Australia. The revision includes an up-to-date dichotomous key for all species.

In the past, several authors focused on the taxonomy of *Sphex*, the most important publications being F. Smith (1856, 1859, 1868), F. F. Kohl (1890, 1895), R. E. Turner (1908, 1910, 1910, 1912, 1915), J. van der Vecht (1955, 1957) and R. V. Hensen (1991). Keys were published, which, among others, included 14 species (Kohl 1890), 16 species (Turner 1910) and 8 species (Hensen 1991) of *Sphex* which were known or assumed to be Australian. However, all of these works are outdated or at least of limited value. Most of the species descriptions by Frederick Smith do not contain sufficient information to distinguish unequivocally the species within the genus, and some of the length ratios given by Kohl seem to be inaccurate. Turner’s (1910) key covers only females, some of which he apparently did not examine personally, and Hensen (1991) focuses on the Malesian Sphecinae, addressing the Australian fauna only partially.

In this study, all previously described species of *Sphex* from Australia were examined, and all 24 species native to Australia based on Cardale (1985) are redescribed. Additionally, 11 new species from Australia are described.

## Methods

### Sources of material

The material used in this study comes from the following institutions. Abbreviations of institution names mostly follow Pulawski (2014). All individuals are pinned.

AMS The Australian Museum, Sydney, Australia

ANIC Australian National Insect Collection, Commonwealth Scientific and Industrial Research Organization, Canberra, ACT, Australia

BMNH The Natural History Museum, formerly British Museum (Natural History), London, Great Britain

CAS California Academy of Sciences, San Francisco, California, USA

MSNG Museo Civico di Storia Naturale di Genova, Genova, Italy

NHMW Naturhistorisches Museum, Vienna, Austria

ZMB Museum für Naturkunde, Berlin, Germany

The following are depositories of types that are mentioned in the species descriptions but have not been examined for this study.

OXUM Hope Department of Entomology, Oxford, Great Britain

RMNH Nationaal Naturhistorisch Museum (formerly Rijksmuseum van Natuurlijke Historie), Leiden, Netherlands

ZMUC Zoological Museum, University of Copenhagen, Copenhagen, Denmark

A total of approximately 900 specimens have been examined; nearly 600 were unidentified, the remaining ones had previously been determined (not always correctly). Roughly 120 individuals had been collected in the Malesian region, the Bismarck Archipelago and the Solomon Islands; the remainder are from Australia.

### Abbreviations of Australian states

ACT Australian Capital Territory

NSW New South Wales

NT Northern Territory

QLD Queensland

SA South Australia

TAS Tasmania

VIC Victoria

WA Western Australia

### Technical devices and programs

A stereoscopic microscope Leica MZ12 and a light source Leica KL 1500 LCD were used for optical examination. The measurements were done with an ocular micrometer.

Photographs of whole insects or different body parts were taken with a Canon EOS 400D Digital camera and a Canon Macro Ring Lite MR-14EX flash device. Detailed multi-focus photographs of the diagnostic characters were taken with a Leica DFC 490 Digital camera on a Leica Z16Apo stereoscopic microscope using Leica Application Suite Version 4.5.0 and merged with Helicon Focus 5.3.14.

The digital drawings were produced with the use of a graphical tablet (Wacom Intuos5 Touch M), Adobe Photoshop CS4 (Version 11.0.2) and Adobe Illustrator CS4 (Version 14.0.0), as described by Coleman (2003).

The species key was generated using DELTA (Version 1.02).

All maps were created with QGIS (Version 2.0.1-Dufour) and the use of Google Earth (Version 7.1.2.2041). The political map of Australia was downloaded from www.naturalearthdata.com.

Images were edited and cleaned of dirt using Adobe Photoshop CS6, Version 13.0.1 and Adobe Illustrator CS6, Version 16.0.1.

The “material examined” section was formatted using the AutoMatEx spreadsheet (Brown 2013) and Microsoft Excel 2010.

## Taxonomy

### Diagnosis of *Sphex*

*Sphex* differs from the closely related *Isodontia* in the length of the petiole and the ratio between the anterior and the posterobasal veinlet of submarginal cell III. *Sphex* has a petiole that is shorter than the combined lengths of hindtarsomeres II–IV, while in most *Isodontia* the petiole is equal to or longer than the hindtarsomeres II–IV combined. The anterior veinlet is usually shorter than the posterobasal in *Sphex*, but conspicuously longer than it in *Isodontia*. The nominate subgenus Sphex (Sphex) ﻿shares the former of these two characters with the subgenus Sphex (Fernaldina),﻿ which comprise two Old World species only, and the anterior veinlet can be shorter or equal to the posterobasal one in Sphex (Fernaldina)﻿. Nonetheless, the fact that a complete spiracular groove is only present in Sphex (Sphex)﻿ differentiates this genus from Isodontia (Bohart and Menke 1976; Ohl 1996).

### Sexual dimorphism in *Sphex*

Several characters that are diagnostic for species of *Sphex* are strongly sexually dimorphic. Most of these characters are listed for Sphecidae *sensu stricto* (Pulawski 2014) by Bohart and Menke (1976; as Sphecinae), but a few are not mentioned there. The most important characters are covered in the following paragraph.

In general, males are more extensively black and have paler wings than females (Bohart and Menke 1976). They also tend to have denser pubescence on the mesosoma and especially on the sterna of the metasoma.

Males often lack the conspicuous structures on the free clypeal margin that many females possess. Although there are a few species in which the male clypeal margin carries lobes, it is mostly truncate and entire. These differences are presumably affiliated with the female life style (see below).

The scutellar morphology is also often sexually dimorphic. It is commonly convex and medially impressed in males, whereas most females have a scutellum that is flatter and lacks impressions.

Finally, the apical metasomal sternum of females lacks diagnostic features almost entirely, but that of males is an important character for species delimitation.

### General remarks

Many of the specimens examined show effects of abrasion. The following characters, which appear to be diagnostic, are especially affected: clypeal lobes, pubescence on all parts of the body, and the female foretarsal rake. Females seem to be more prone to this effect, apparently as a result of the nest digging.

Localities outside of Australia and those of specimens which could not be reliably determined were not included in the maps. Localities of Australian specimens that have not been examined in this study were included if they appear to be plausibly within the known range of the species.

Within each species group, the species are arranged alphabetically. Locality records in the “Material examined” section are arranged in the following sequence under each state: unspecified localities are listed first, very imprecise records are listed after that, and finally localites in alphabetical order. Records that begin with information about a specific distance or direction are listed at the respective locations that they use as reference.

### Notes regarding descriptions and key to species

The classification of the species in this paper follows Hensen (1991) in his subdivision of the Malesian and Australian species of the genus into three species-groups, the *Sphex argentatus*, *Sphex resplendens* and *Sphex subtruncatus* species groups. All species from Australia can be readily assigned to one of the groups.

The measurements of the petiole were done from above from the center of the joint at the posterior end of the propodeum to the anterior margin of tergum I (Fig. 7). The morphological nomenclature was taken from Bohart and Menke (1976).

Termini for describing bristles are used in this work in the following manner: Very short, fine and usually appressed ones with a moderate to high density are called “tomentum”; they are found mostly on the metasomal terga of the wasp. The term “pubescence” is applied when characterizing bristles that cover a larger area like the face or the propodeal enclosure. They are considerably longer than those in the tomentum, can be appressed or erect, and vary between low and very high density. Finally, “setae” describe smaller groups of bristles that are isolated in some way, either literally by forming fringes or figuratively by being differently colored than the surrounding pubescence, the latter of which is often the case on the clypeus. Setae are usually long and erect.

For each species, only synonyms which are relevant for the currently valid name of the respective species are listed, or which are referred to in the descriptions. The complete lists of synonyms of all species can be found in Pulawski (2014). Twelve species are known from one sex only. The unknown females of *Sphex caelebs*, *Sphex fortunatus*, *Sphex latilobus* and *Sphex semifossulatus* as well as the unknown males of *Sphex ahasverus*, *Sphex brevipetiolus*,﻿ *Sphex darwiniensis*, *Sphex decoratus*, *Sphex flammeus*,﻿ *Sphex gilberti*, *Sphex imporcatus* and *Sphex rhodosoma* are not included in the key.

### Key to species

**Table:** 

1	♀♀: antenna with 10 flagellomeres; outer side of foretarsomere I with markedly long spines (Fig. 1A)	**2**
–	♂♂: antenna with 11 flagellomeres; spines on outer side of foretarsomere I as long as other spines, not markedly long (Fig. 1B)	**44**
♀♀		
2(1)	Metanotum without tubercles (indistinct median impression may be present in some specimens) (Fig. 1C)	**3**
–	Metanotum with markedly developed median pair of tubercles (Fig. 1D, E)	**34**
3(2)	Claw teeth perpendicular to inner margin of claw (Fig. 2A)	**4**
–	Claw teeth obliquely oriented to inner margin of claw (Fig. 2B)	**15**
***Sphex resplendens* group**		
4(3)	Legs black or dark brown; scape black or dark brown	**5**
–	Legs at least partly orange or bright ferruginous; scape for most part orange	**14**
5(4)	Cellular wing area largely hyaline (Fig. 2C–E)	**6**
–	Cellular wing area fuscous (Fig. 2F)	**10**
6(5)	Metasoma entirely black or dark blue; length of petiole approximately equal to flagellomere II	**7**
–	Apical three metasomal terga and apical three to four metasomal sterna orange; petiole slightly shorter than flagellomere II	***Sphex mimulus* R. Turner**
7(6)	Propodeal enclosure finely sculptured, without transverse ridges (Fig. 3A); clypeal surface plain	**8**
–	Propodeal enclosure markedly ridged (Fig. 3B–C); clypeal surface ventrally with curved wrinkles (Fig. 4E, 19B)	***Sphex imporcatus* sp. n.**
8(7)	Appressed pubescence and erect setae on clypeus silvery-white (Fig. 6A–C)	***Sphex gracilis* sp. n.**
–	Apressed pubescence silvery-white, but erect setae on clypeus dark brown or black (Fig. 6E)	**9**
9(8)	Wing veins orange to light brown (Fig. 2D); pubescence on propodeal enclosure black (Fig. 5E); wing membrane at least partially with yellow tinge in cellular wing area (Fig. 2D)	***Sphex gilberti* R. Turner**
–	Wing veins brown (Fig. 2C, E, F); pubescence on propodeal enclosure silvery-white (Fig. 5A–C); wing membrane without yellow tinge (Fig. 2C, E)	***Sphex luctuosus* F. Smith**
10(5)	Metasoma entirely black or dark blue	**11**
–	Metasoma at least partly ferruginous	**13**
11(10)	Pubescence on propodeal enclosure silvery-white (Fig. 5A–C)	**12**
–	Pubescence on propodeal enclosure black (Fig. 5E)	***Sphex resplendens* Kohl**
12(11)	Petiole longer than flagellomere II; free clypeal margin without lobes (Fig. 6A)	***Sphex rugifer* Kohl**
–	Length of petiole approximately equal to flagellomere II; free clypeal margin with pair of lobes (Fig. 6D–E)	***Sphex fumipennis* F. Smith**
13(10)	Petiole shorter than flagellomere II; wing membrane at least partially with yellow tinge in cellular wing area (Fig. 2D); free clypeal margin with pair of lobes (Fig. 6E)	***Sphex mimulus* R. Turner**
–	Petiole longer than flagellomere II; wing membrane without yellow tinge (Fig. 2C, E, F); free clypeal margin without lobes (Fig. 6A)	***Sphex rugifer* Kohl**
14(4)	Mesosoma orange	***Sphex rhodosoma* (R. Turner)**
–	Mesosoma black	***Sphex darwiniensis* R. Turner**
***Sphex subtruncatus* group**		
15(3)	Legs black or dark brown	**16**
–	Legs at least partly orange or bright ferruginous	**32**
16(15)	Appressed pubescence and erect setae on clypeus silvery-white or golden (Fig. 6A–D)	**17**
–	Apressed pubescence silvery-white, but erect setae on clypeus dark brown or black, at least some of them and at least at tips (Fig. 6E)	**28**
17(16)	Wing veins brown to black (Fig. 2C, E, F)	**18**
–	Wing veins at least partially bright orange (Fig. 2D)	**24**
18(17)	Scutellum flat (Fig. 3E)	**19**
–	Scutellum convex medially (Fig. 3D, F)	**20**
19(18)	Petiole longer than flagellomere II; pubescence on propodeal enclosure not concealing sculpture (Fig. 5C)	***Sphex jucundus* sp. n.**
–	Petiole shorter than flagellomere II; pubescence on propodeal enclosure completely concealing sculpture (Fig. 5A, B, D)	***Sphex ermineus* Kohl**
20(18)	Pubescence on propodeal enclosure silvery-white (Fig. 5A–C)	**21**
–	Pubescence on propodeal enclosure golden (Fig. 5D)	**23**
21(20)	Wing membrane at least partially with yellow tinge in cellular wing area (Fig. 2D)	***Sphex cognatus* F. Smith**
–	Wing membrane without yellow tinge (Fig. 2C, E, F)	**22**
22(21)	Scutellum without impression (Fig. 3D–E); clypeus without glabrous stripe (Fig. 2G), with golden appressed pubescence (Fig. 6D); free clypeal margin with pair of lobes (Fig. 6D–E)	***Sphex argentatissimus* sp. n.**
–	Scutellum with median impression (Fig. 3F); clypeus with medial glabrous stripe (Fig. 2H), with appressed silvery-white pubescence (Fig. 6A–C, E); free clypeal margin without lobes (Fig. 6A)	***Sphex bilobatus* Kohl**
23(20)	Wing membrane at least partially with yellow tinge in cellular wing area (Fig. 2D); clypeus without glabrous stripe (Fig. 2G)	***Sphex cognatus* F. Smith**
–	Wing membrane without yellow tinge (Fig. 2C); ventral part of clypeus glabrous (Fig. 6A)	***Sphex vestitus* F. Smith**
24(17)	Petiole longer than flagellomere II	**25**
–	Petiole shorter than flagellomere II	***Sphex brevipetiolus* sp. n.**
25(24)	Scutellum without impression (Fig. 3D–E)	**26**
–	Scutellum with median impression (Fig. 3F)	**27**
26(25)	Pubescence on propodeal enclosure completely concealing sculpture (Fig. 5A, B, D)	***Sphex cognatus* F. Smith**
–	Pubescence on propodeal enclosure not concealing sculpture (Fig. 5C, E)	***Sphex formosellus* van der Vecht**
27(25)	Pubescence on propodeal enclosure completely concealing sculpture (Fig. 5A, B, D)	***Sphex cognatus* F. Smith**
–	Pubescence on propodeal enclosure not concealing sculpture (Fig. 5C)	***Sphex pretiosus* sp. n.**
28(16)	Cellular wing area largely hyaline (Fig. 2C–E)	**29**
–	Cellular wing area fuscous (Fig. 2F)	***Sphex ahasverus* Kohl**
29(28)	Scutellum flat (Fig. 3E); petiole shorter than flagellomere II	**30**
–	Scutellum convex medially (Fig. 3D, F); petiole longer than flagellomere II	**31**
30(29)	Pubescence on propodeal enclosure completely concealing sculpture (Fig. 5A, B, D)	***Sphex ermineus* Kohl**
–	Pubescence on propodeal enclosure not concealing sculpture (Fig. 5C)	***Sphex corporosus* sp. n.**
31(29)	Wing membrane with yellow tinge in cellular wing area (Fig. 2D)	***Sphex modestus* F. Smith**
–	Wing membrane without yellow tinge (Fig. 2C, E, F)	***Sphex finschii* Kohl**
32(15)	Metasoma entirely black; scape black or dark brown	***Sphex basilicus* (R. Turner)**
–	Metasoma at least partly ferruginous; scape for most part orange	**33**
33(32)	Clypeus with silvery-white appressed pubescence (Fig. 6A–C, E)	***Sphex flammeus* sp. n.**
–	Clypeus with golden appressed pubescence (Fig. 6D)	***Sphex staudingeri* Gribodo**
***Sphex argentatus* group**		
34(2)	Legs black or dark brown; metasoma entirely black or dark blue; scape black or dark brown	**35**
–	Legs at least partly orange or bright ferruginous; metasoma at least partly ferruginous; scape for most part orange	**42**
35(34)	Appressed pubescence and erect setae on clypeus silvery-white or golden (Fig. 6A–D)	**36**
–	Appressed pubescence silvery-white, but erect setae on clypeus dark brown or black, at least some of them and at least at tips (Fig. 6E)	**39**
36(35)	Wing veins brown to black (Fig. 2C, E, F)	**37**
–	Wing veins at least partially bright orange (Fig. 2D)	**38**
37(36)	Clypeus without glabrous stripe (Fig. 2G)	***Sphex argentatus* Fabricius**
–	Clypeus with medial glabrous stripe (Fig. 2H)	***Sphex carbonicolor* van der Vecht**
38(36)	Petiole longer than flagellomere II	***Sphex pretiosus* sp. n. (member of *Sphex subtruncatus* group)**
–	Petiole shorter than flagellomere II	***Sphex brevipetiolus* sp. n. (member of *Sphex subtruncatus* group)**
39(35)	Scutellum flat (Fig. 3E); petiole shorter than flagellomere II	***Sphex corporosus* sp. n.**
–	Scutellum convex medially (Fig. 3D, F); petiole longer than flagellomere II	**40**
40(39)	Wing membrane with yellow tinge in cellular wing area (Fig. 2D)	***Sphex modestus* F. Smith**
–	Wing membrane without yellow tinge (Fig. 2C, E, F)	**41**
41(40)	Clypeus with silvery-white appressed pubescence (Fig. 6A–C, E); pubescence on propodeal completely concealing sculpture (Fig. 5C)	***Sphex finschii* Kohl**
–	Clypeus with golden appressed pubescence (Fig. 6D); pubescence on propodeal enclosure not concealing sculpture (Fig. 5B)	***Sphex ephippium* F. Smith**
42(34)	Propodeal enclosure finely sculptured, without transverse ridges (Fig. 3A)	**43**
–	Propodeal enclosure markedly transversely ridged (Fig. 3C)	***Sphex sericeus* (Fabricius)**
43(42)	Wing membrane at least partially with yellow tinge in cellular wing area (Fig. 2D), metasoma partly black	***Sphex decoratus* F. Smith**
–	Wing membrane without yellow tinge (Fig. 2C, E, F), metasoma entirely ferruginous	***Sphex flammeus* sp. n. (member of *Sphex subtruncatus* group)**
♂♂		
44(1)	Metanotum without tubercles (indistinct median impression may be present in some specimens) (Fig. 3)	**45**
–	Metanotum with markedly developed median pair of tubercles (Fig. 4/5)	**70**
45(44)	Claw teeth perpendicular to inner margin of claw (Fig. 1)	**46**
–	Claw teeth obliquely oriented to inner margin of claw (Fig. 2)	**51**
***Sphex resplendens* group**		
46(45)	Metasoma entirely black or dark blue	**47**
–	Three apical metasomal terga pale yellowish-orange	***Sphex mimulus* R. Turner**
47(46)	Cellular wing area largely hyaline (Fig. 2C–E)	**48**
–	Cellular wing area fuscous (Fig. 2F)	**49**
48(47)	Appressed pubescence and erect setae on clypeus silvery-white (Fig. 6A)	***Sphex gracilis* sp. n.**
–	Appressed pubescence silvery-white, but erect setae on clypeus dark brown or black, at least some of them and at least at tips (Fig. 6E)	***Sphex luctuosus* F. Smith**
49(47)	Propodeal enclosure and clypeus with silvery-white pubescence (Fig. 5A–C and Fig. 6A–C)	**50**
–	Pubescence on propodeal enclosure black (Fig. 5E); clypeus with appressed golden pubescence (Fig. 6D)	***Sphex resplendens* Kohl**
50(49)	Forewing apically and hindwing distally brighter, remainder of the wing area markedly fuscous (Fig. 16A, B)	***Sphex fumipennis* F. Smith**
–	Wings fuscous except for hindwing, which is hyaline basally (Fig. 24B, C)	***Sphex rugifer* Kohl**
***Sphex subtruncatus* group**		
51(45)	Legs black or dark brown	**52**
–	Legs at least partly orange or bright ferruginous	**66**
52(51)	Apical part of metasomal sternum VIII divided into two large lobes (Fig. 4A)	**53**
–	Apical part of metasomal sternum VIII entire (Fig. 4B–D)	**65**
53(52)	Apex of metasomal sternum VIII broadly or narrowly pointed (Fig. 4B, C)	**54**
–	Apex of metasomal sternum VIII notched (Fig. 4D, 39B)	**59**
54(53)	Appressed pubescence and erect setae on clypeus silvery-white or golden (Fig. 6A–D); lateral margin of metasomal sternum VIII shallowly concave (Fig. 4C)	**55**
–	Appressed pubescence silvery-white, but erect setae on clypeus dark brown or black, at least some of them and at least at tips (Fig. 6E); lateral margin of metasomal sternum VIII straight or slightly convex (Fig. 4B, D)	**58**
55(54)	Clypeus with golden pubescence (Fig. 6D), without glabrous stripe (Fig. 2G)	**56**
–	Clypeus with silvery-white pubescence (Fig. 6A–C), with medial glabrous stripe (Fig. 6C)	**57**
56(55)	Free clypeal margin without lobes (Fig. 6A); pubescence on propodeal enclosure not concealing sculpture (Fig. 5C)	***Sphex formosellus* van der Vecht**
–	Free clypeal margin with pair of lobes (Fig. 6D); pubescence on propodeal enclosure completely concealing sculpture (Fig. 5A, B, D)	***Sphex cognatus* F. Smith**
57(55)	Wings light brown, markedly fuscous along subcosta and below submedial cell (Fig. 36)	***Sphex fortunatus* sp. n.**
–	Wings entirely hyaline (Fig. 2C)	***Sphex jucundus* sp. n.**
58(54)	Wing membrane at least partially with yellow tinge in cellular wing area (Fig. 2D)	***Sphex modestus* F. Smith**
–	Wing membrane without yellow tinge (Fig. 2C, E, F)	***Sphex finschii* Kohl**
59(53)	Wing veins brown to black (Fig. 2C, E, F)	**60**
–	Wing veins at least partially bright orange (Fig. 2D)	**64**
60(59)	Lateral margin of metasomal sternum VIII straight or slightly convex (Fig. 4B, D)	**61**
–	Lateral margin of metasomal sternum VIII shallowly concave (Fig. 4C)	**62**
61(60)	Pubescence on propodeal enclosure silvery-white (Fig. 5A); clypeus without glabrous stripe (Fig. 2G); free clypeal margin plain, without lobe (Fig. 6A)	***Sphex argentatissimus* sp. n.**
–	Pubescence on propodeal enclosure golden (Fig. 5D); ventral part of clypeus glabrous (Fig. 6A); free clypeal margin broadly emarginated, with a broad triangular median lobe (Fig. 6B)	***Sphex vestitus* F. Smith**
62(60)	Petiole longer than flagellomere II	***Sphex argentatissimus* sp. n.**
–	Petiole shorter than flagellomere II	**63**
63(62)	Clypeus with appressed silvery-white pubescence (Fig. 6A–C, E); pubescence on propodeal enclosure not concealing sculpture (Fig. 5C)	***Sphex corporosus* sp. n.**
–	Clypeus with appressed golden pubescence (Fig. 6D); pubescence on propodeal enclosure completely concealing sculpture (Fig. 5A, B)	***Sphex ermineus* Kohl**
64(59)	Petiole longer than flagellomere II; pubescence on propodeal enclosure golden (Fig. 5D); wing membrane at least partially with yellow tinge in cellular wing area (Fig. 2D)	***Sphex pretiosus* sp. n.**
–	Petiole shorter than flagellomere II; pubescence on propodeal enclosure silvery-white (Fig. 5A–C); wing membrane without yellow tinge (Fig. 2C)	***Sphex corporosus* sp. n.**
65(52)	Free clypeal margin entirely black (Fig. 2G)	***Sphex bilobatus* Kohl**
–	Free clypeal margin at least partially bright orange (Fig. 2H)	***Sphex latilobus* sp. n.**
66(51)	Apical part of metasomal sternum VIII entire (Fig. 4B–D)	**67**
–	Apical part of metasomal sternum VIII divided into two large lobes (Fig. 4A)	**69**
67(66)	Metasoma entirely black; erect setae on clypeus dark brown or black, at least some of them and at least at tips (Fig. 6E)	***Sphex caelebs* sp. n.**
–	Metasoma at least partly ferruginous; erect setae on clypeus silvery-white or golden (Fig. 6A–D)	**68**
68(67)	Wing veins brown (Fig. 2C, E, F); scape for most part orange; pubescence on propodeal enclosure and appressed pubescence on clypeus golden (Fig. 5D and Fig. 6D)	***Sphex staudingeri* Gribodo**
–	Wing veins at least partially bright orange (Fig. 2D); scape black or dark brown; pubescence on propodeal enclosure and appressed pubescence on clypeus silvery-white (Fig. 5A–C and Fig. 6A–C, E)	***Sphex semifossulatus* van der Vecht**
69(66)	Pubesence on propodeal enclosure silvery-white (Fig. 5A–C); free clypeal margin at least partially bright orange and without lobes (Fig. 2H)	***Sphex latilobus* sp. n.**
–	Pubescence on propodeal enclosure golden (Fig. 5D); free clypeal margin entirely black and with single median lobe (Fig. 6B)	***Sphex basilicus* (R. Turner)**
***Sphex argentatus* group**		
70(44)	Appressed pubescence and erect setae on clypeus silvery-white (Fig. 6A–C)	**71**
–	Appressed pubescence silvery-white, but erect setae on clypeus dark brown or black, at least some of them and at least at tips (Fig. 6E)	**74**
71(70)	Apex of metasomal sternum VIII broadly or narrowly pointed (Fig. 4B, C)	**72**
–	Apex of metasomal sternum VIII notched (Fig. 4D, 39B)	**73**
72(71)	Propodeal enclosure finely sculptured, without transverse ridges (Fig. 3A); clypeus without glabrous stripe (Fig. 2G); free clypeal margin concave towards center, with short median lobe (Fig. 6B)	***Sphex argentatus* Fabricius**
–	Propodeal enclosure markedly transversely ridged (Fig. 3C); clypeus with medial glabrous stripe (Fig. 2H); free clypeal margin concave towards center, but without median lobe (Fig. 6A)	***Sphex sericeus* (Fabricius)**
73(71)	Lateral margin of metasomal sternum VIII straight or slightly convex (Fig. 4D); petiole slightly longer than flagellomere II	***Sphex carbonicolor* van der Vecht**
–	Lateral margin of metasomal sternum VIII shallowly concave (Fig. 4C); petiole shorter than flagellomere II	***Sphex corporosus* sp. n.**
74(70)	Wing membrane at least partially with yellow tinge in cellular wing area (Fig. 2D)	***Sphex modestus* F. Smith**
–	Wing membrane without yellow tinge (Fig. 2C, E, F)	**75**
75(74)	Clypeus with appressed silvery-white pubescence (Fig. 6A–C, E); pubescence on propodeal enclosure not concealing sculpture (Fig. 5C)	***Sphex finschii* Kohl**
–	Clypeus with appressed golden pubescence (Fig. 6D); pubescence on propodeal enclosure completely concealing sculpture (Fig. 5B)	***Sphex ephippium* F. Smith**

**Figure 1.**
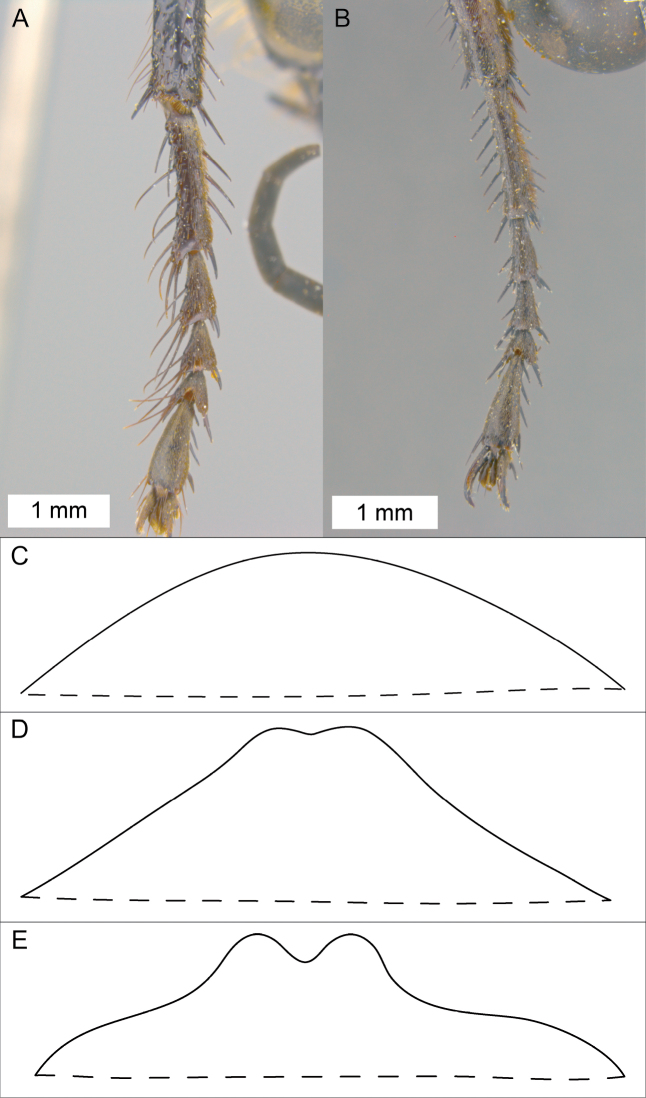
Various diagnostic characters. **A** foreleg of *Sphex vestitus*, ♀ **B** foreleg of *Sphex luctuosus*, ♂ **C–E** different profiles of the metanotum **C** metanotum without tubercles **D** metanotum with indistinct tubercles **E** metanotum with distinct tubercles.

**Figure 2.**
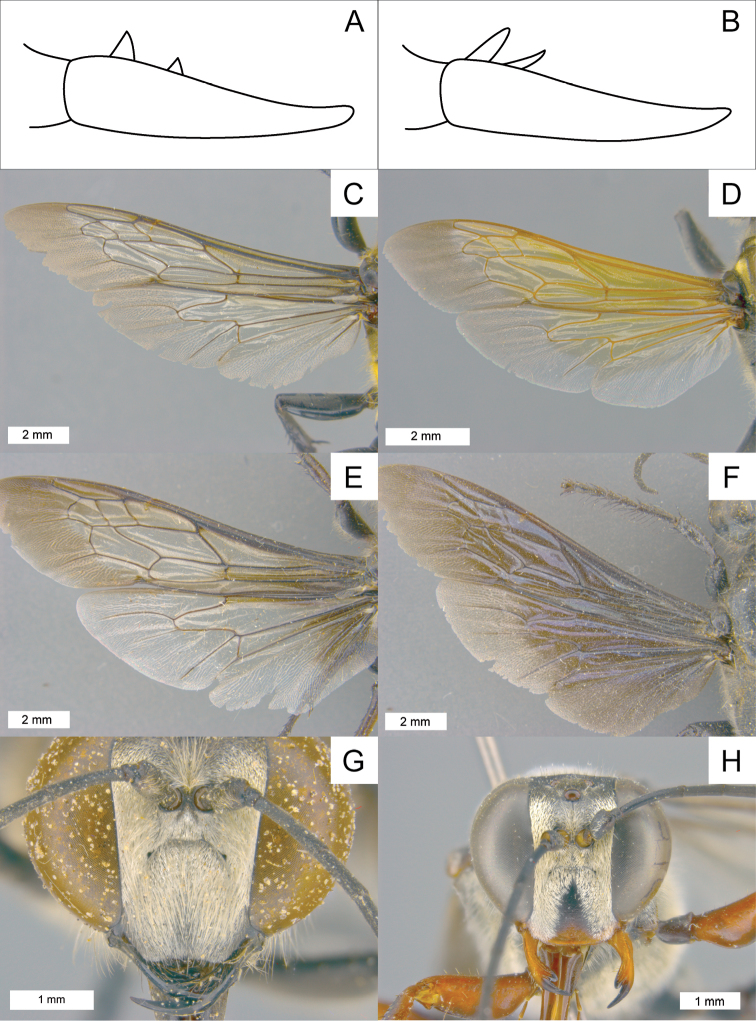
Various diagnostic characters. **A** claw teeth perpendicular **B** claw teeth angled **C**–**F** fore- and hindwing of different species **C** *Sphex vestitus*, ♀, wings hyaline **D** *Sphex formosellus*, ♀, wings with yellow tinge **E** *Sphex luctuosus*, ♂, wings darkened near base **F** *Sphex fumipennis*, ♀, wings mostly darkened **G** frontal view of *Sphex argentatus*, ♂ **H** frontal view of *Sphex latilobus*, ♂.

**Figure 3.**
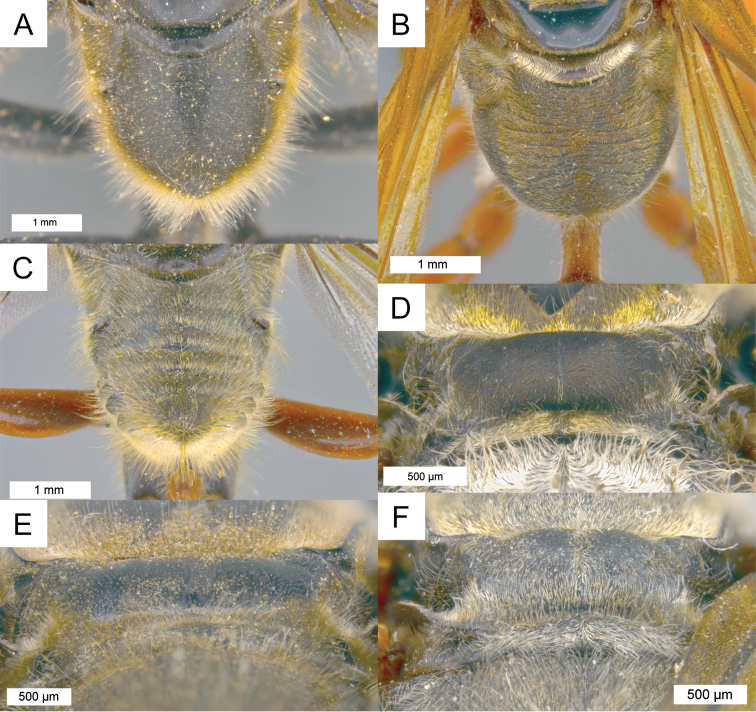
Various diagnostic characters. **A–C** dorsal view of propodeum **A** *Sphex argentatus*, ♂ no ridges on propodeal enclosure **B** *Sphex darwiniensis*, ♀, fine ridges on propodeal enclosure **C** *Sphex sericeus*, ♂, marked ridges on propodeal enclosure **D–F** dorsoposterior view of scutellum **D** *Sphex argentatissimus*, ♀, scutellum convex without notable impressions **E** *Sphex mimulus*, ♀, scutellum flat **F** *Sphex latilobus*, ♂, scutellum convex with notable impression.

**Figure 4.**
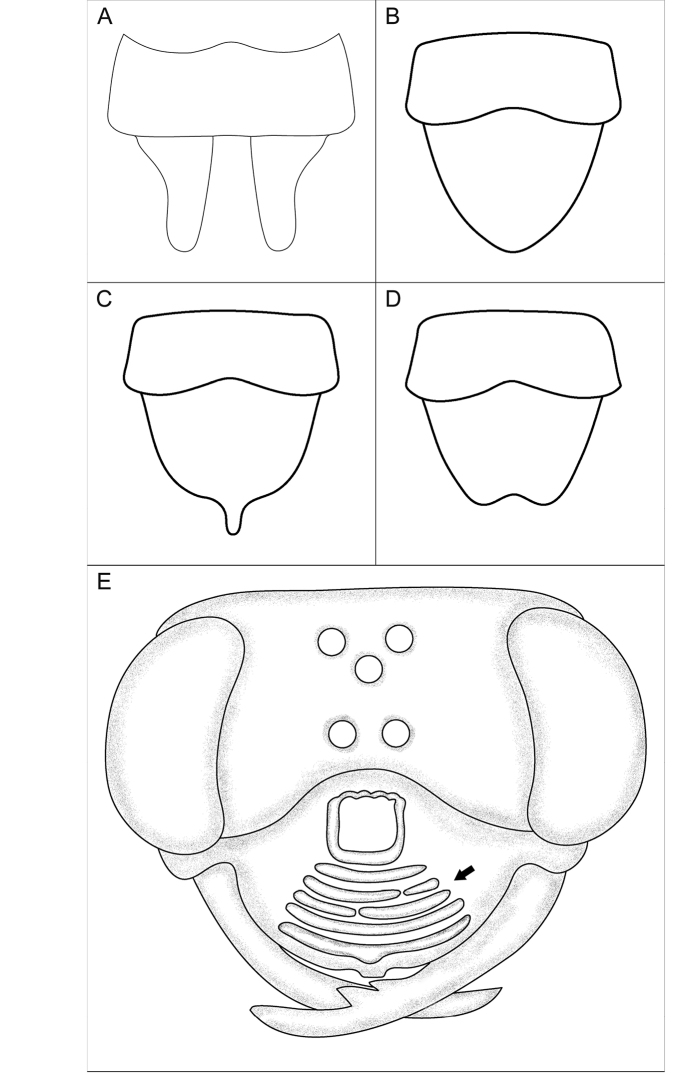
Various diagnostic characters. **A–D** ventral view of metasomal sterna VII and VIII **A** sternum VIII modified into two lobes **B** sternum VIII with convex lateral margin **C** sternum VIII with concave lateral margin **D** sternum VIII with notch at apical margin **E** head of *Sphex imporcatus*, ♀, clypeus with conspicuous ridges (arrow).

**Figure 5.**
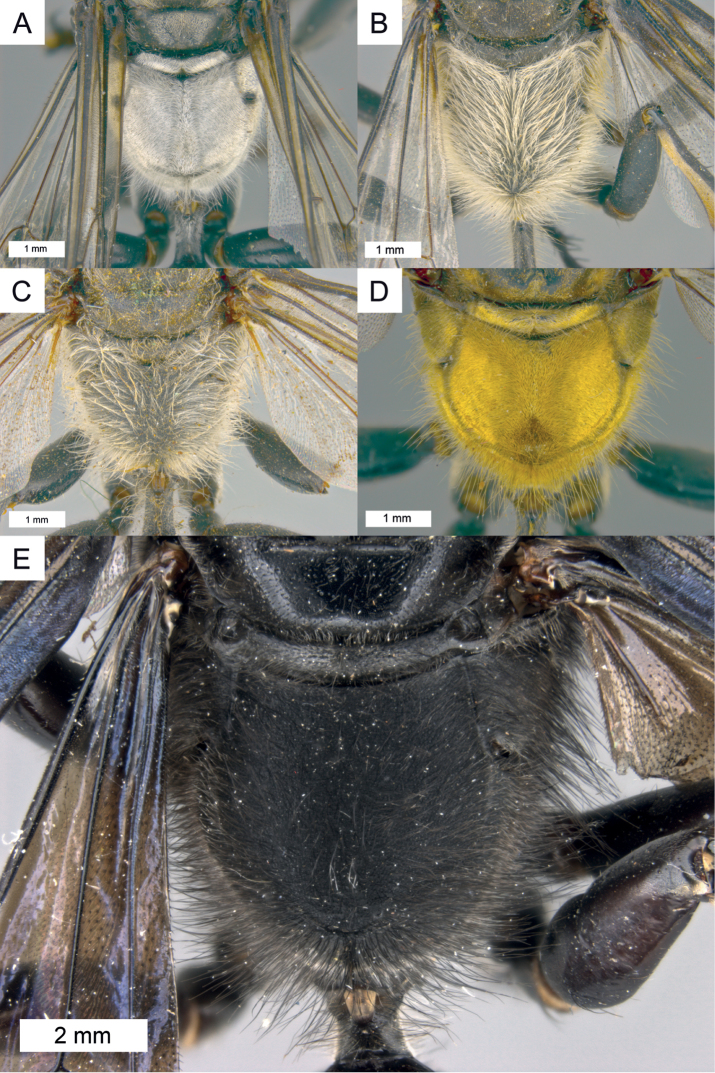
Dorsal view on propodeum in different species **A–C** pubescence silvery **A** *Sphex argentatissimus*, ♂, pubescence mostly short and dense **B** *Sphex ephippium*, ♂, pubescence mostly long and dense **C** *Sphex bilobatus*, ♂, pubescence sparse **D** *Sphex vestitus*, ♀, pubescence golden **E** *Sphex resplendens*, ♀, pubescence black.

**Figure 6.**
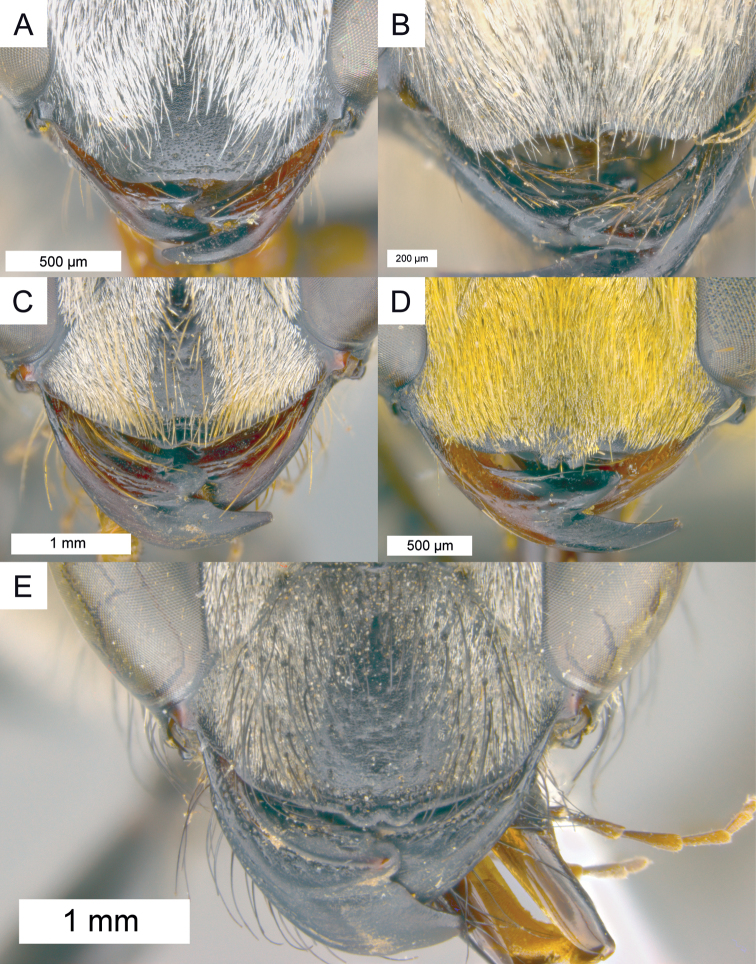
Frontal view of clypeus in different species. **A–D** color of erect setae on clypeus largely matching color of appressed setae. **A** *Sphex gracilis*, ♂, free clypeal margin simple **B** *Sphex argentatus*, ♂, free clypeal margin with single lobe **C** *Sphex brevipetiolus*, ♀, free clypeal margin shaped indistinctly **D** *Sphex cognatus*, ♂, free clypeal margin with two lobes **E** *Sphex mimulus*, ♀, erect setae on clypeus black.

**Figure 7.**
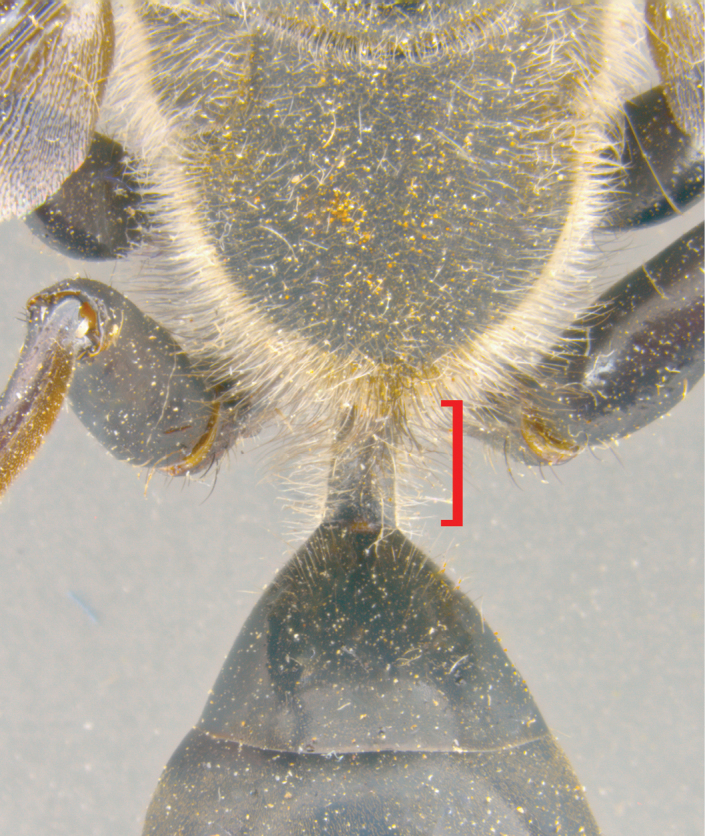
How to measure petiole length. Dorsal view, the red bracket demonstrates the length of the part that is measured.

## Species descriptions

### Species group of *Sphex argentatus*

Australian species of this group are characterized by a markedly raised metanotum in conjunction with a longitudinal median impression of varying depth and width (Hensen 1991). In this paper, the resulting paired elevations are called tubercles, and the depth of the impression between them can be used to furthermore differentiate between the group members. It should be noted, however, that for species with an only inconspicuosuly raised metanotum, the presence of a longitudinal impression on it was considered to be insufficient as a single character for the species group assignment. Species with this combination of features have been assigned to species groups based on their observed intraspecific variation. Currently, there are six Australian species in this group.

Since the other attributes mentioned by Hensen (1991) in his diagnosis of the *Sphex argentatus* group are also present in at least some species from the other two groups like a convex and medially impressed scutellum, placoids on the male antenna (see also diagnosis of *Sphex resplendens* group) or are diagnostic for the entire genus (like a bidentate mandible) they have been largely ignored in the delimitation of the species groups. The characters of the metanotum have been found to be sufficient for this.

#### Sphex argentatus

Taxon classificationAnimaliaHymenopteraSphecidae

Fabricius, 1787

Sphex argentatus Fabricius, 1787: 274, sex not indicated (as *argentata*, incorrect original termination). Lectotype: ♀, India: Coromandel (= southeastern coast): no specific locality (ZMUC), designated by van der Vecht 1961: 28. Not examined.
Sphex umbrosus Christ, 1791: 293, sex not indicated. Holotype or syntypes: origin not indicated (destroyed). Synonymized with *Sphex argentatus* by van der Vecht 1961: 28, and 1973: 345. Not examined.

##### Material examined.

**[COUNTRY UNKNOWN]:** **[state unknown]:** [no specific locality], 1♂, 01.04.1892 (ANIC). **AUSTRALIA:** **[state unknown]:** [no specific locality], 2♀ (BMNH); **NSW:** Sydney, 1♀ (BMNH); **NT:** Port Darwin, 1♀, 1♂ (BMNH); **QLD:** [no specific locality], 1♂, E. Saunders (BMNH); Brisbane, 1♂, 1957, F. G. T. Smith (BMNH), 1♀, 08.02.1923, A. N. Burns (ANIC); Burleigh Heads, 1♂, 10.03.1956, J. Keir (ANIC); Byfield State Forest, 1♀, 01.01.1976, G. Daniels (AMS); Cairns, 1♂, 01.04.1963, E. C. Corbet (BMNH); 8 km W of Cooktown, 1♂, 17.07.1982, N. W. Rodd (AMS); Iron Range, 1♀, 26.04.1975, M. S. Moulds (AMS); Mackay, 1♂, 01.04.1892 (ANIC); Meringa, 1♂, 19.03.1927, A. N. Burns (ANIC); Rockhampton, 3♂, 12.01.1973, M. Moulds (AMS); Westwood, 1♀, 01.03.1925, A. N. Burns (ANIC); Wondecla near Herberton, 1♂, 06.01.1990, M. S. & B. J. Moulds (AMS). **INDONESIA:** **Papua:** 30 km S Nabire, 1♀, 26.07.1998, Balke (NHMW); **West Java Province:** Bogor, Java, 1♀, 1931, G. L. Windred (ANIC). **PAPUA NEW GUINEA:** **Morobe Province:** Finschhafen, 1♀, Loganeg (ANIC).

##### Diagnosis.

*Sphex argentatus* is distinguished from other Australian *Sphex* by the combination of tubercles on the metanotum and the clypeus having no glabrous stripe.

##### Description.

Body black. Base of fore- and hindwing membrane darkened, forewing with fuscous spot beyond marginal cell. Wing veins brown to black. Appressed pubescence and erect setae on clypeus and frons silvery-white, no medial glabrous stripe on clypeus. Pubescence on collar and scutum silvery, on scutum slightly denser laterally than medially. Tubercles on metanotum distinct. Propodeal enclosure with thin, erect silvery setae, leaving sculpture well visible.

*Female*: Body length 21.6–32.4 mm. Forebasitarsal rake with 10 long spines. Free clypeal margin with two inconspicuous lobes medially, distance between them less than 1/8 length of flagellomere II. Distance between hind-ocelli 0.8× their shortest distance to compound eyes. Scutellum flat, with shallow medial impression near posterior margin. Length of petiole 1.4× length of flagellomere II. Tomentum sparse on metasomal tergum I, absent on tergum II.

*Male*: Body length 23.8–26.2 mm. Free clypeal margin truncate, slightly concave toward center, with short median lobe. Distance between hind-ocelli 1.4× their shortest distance to compound eyes. Scutellum convex, with shallow medial impression. Length of petiole 1.65× length of flagellomere II. Tomentum moderately dense on metasomal tergum I and II. Metasomal tergum V with only a few, tergum VI with considerable number of black setae. Metasomal sternum VII with large fringe of dark setae laterally, sterna anterior of it each with a lesser amount of setae. Metasomal sternum VIII entire, its lateral margin straight.

**Figure 8.**
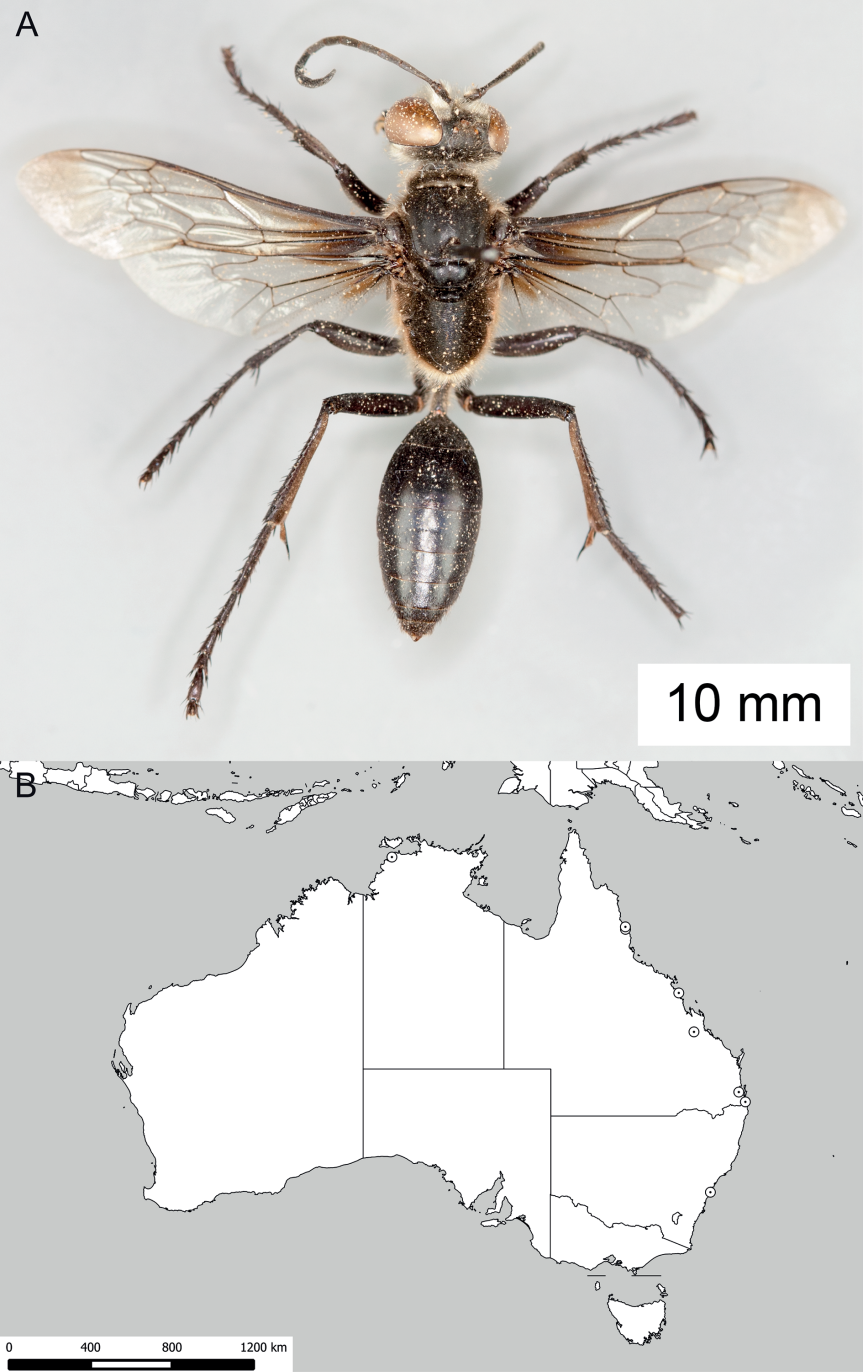
*Sphex argentatus*. **A** ♂, habitus **B** collecting localities.

##### Notes on type material.

The types of *Sphex argentatus* and its synonyms were not examined, because of the character combination in the redescription of the species by Kohl (1890) (as *Sphex umbrosus*, synonymized with *Sphex argentatus* by van der Vecht (1961, 1973): black body, bituberculate metanotum, uniformly silvery pubescence on face, sculpture on propodeal dorsum visible through moderately dense pubescence) is sufficient to unambiguously identify this species.

#### Sphex carbonicolor

Taxon classificationAnimaliaHymenopteraSphecidae

van der Vecht, 1973

Sphex carbonarius F. Smith, 1856: 247, ♀ (as *carbonaria*, incorrect original termination), junior primary homonym of *Sphex carbonarius* Scopoli, 1763. Holotype or syntypes: ♀, Australia: Sydney (BMNH). Not examined.
Sphex carbonicolor van der Vecht, 1973: 342 Substitute name for *Sphex carbonarius* F. Smith.

##### Material examined.

**[COUNTRY UNKNOWN]:** **[state unknown]:** [no specific locality], 2♀ (AMS). **AUSTRALIA:** **NSW:** Barraba, 1♀, March 37, M. Showers (AMS); Blue Mountains, 1♂, 08.01.1983, N. W. Rodd (AMS); Caldwell, 1♀, 1♂, 12.01.1952, V. Robb (AMS); Casula, 1♂, 11.02.1958, M. I. Nikitin (BMNH), 1♂, 24.02.1958, M. I. Nikitin (BMNH); 40 km E of Coonabarabran, 1♀, 18.11.1990, N. W. Rodd (AMS); Glen Innes, 1♀, 26.12.1979, A. W. Cameron (AMS); Tamarama near Sydney, 1♀, 19.12.2003 (AMS); **NT:** Central Australia, 1♀, 23.06.1927, G. Horns (ANIC); Finke Gorge National Park, Alice Springs, 1♀, 20.10.1973, G. Griffin (ANIC); **QLD:** Blackdown Tableland Expedition Range, 1♂, 08.01.1976, G. Daniels (AMS); Burnett River, 1♀, 1891/1892, R. Lennon (BMNH); Cairo Station, 1♀, 15.01.-31.01.1954, C. Mc.C. (ANIC); Captain Billy Landing, Cape York, 11°38'S, 142°51'E, 1♂, 11.03.1992, G. Daniels & M. A. Schneider (ANIC); Charters Towers, 1♀, 20.07.1902, W. W. Froggatt (ANIC); Division of Dawson, 1♀, Rothschild & Bequest (BMNH); Kensington Downs, 2♀ (AMS); Rockhampton, 1♀ (ZMB); **SA:** Adelaide, 1♀, 08.01.1988, A. D. Austin (BMNH), 1♀, 1♂ (ZMB); Urrbrae, 1♀, 1944 (ANIC); Wilpena Pound Resort, Flinders Ranges, 1♂, 24.01.1995, L. Packer (ZMB); **WA:** 30 km N of Carnavon, Blow Holes Road, 1♂, 21.07.1978, G. A. Holloway (AMS); Carnarvon, 1♀, 01.08.1953, A. Snell (ANIC); Geraldton, 1♂, 1917, J. Clark (ANIC); Glen Forrest, 1♂, 11.12.1949, I. M. (ANIC); Lyndon Station, NW Basin, 1♀, 01.07.1950, G. Thomas (ANIC); Marloo Station, 1♂, 01.01.1935, Gebr. Goerling (ZMB), 1♂, 02.03.1935, A. Goerling (ZMB), 1♂, 01.02.1937, Gebr. Goerling (ZMB); Ongerup, 33°57.9'S, 118°28.8'E, 1♂, 28.11.2008, D. M. Bray & W. J. Pulawski (CAS).

##### Diagnosis.

This species differs from other members of the *Sphex argentatus* group in having distinct tubercles on the metanotum, combined with completely hyaline wings except for a slight brown tinge at the apical margin of the forewing in some specimens. Specimens of *Sphex sericeus* that may have the same color pattern are identifiable by the conspicuous transverse ridges on their propodeum, which are lacking in *Sphex carbonicolor*. *Sphex argentatus* and *Sphex finschii* have a darkened wing base, while *Sphex decoratus* differs by having orange legs (which are dark brown or black in *Sphex carbonicolor*).

##### Description.

Body length 27.6–35.6 mm. Body black. Wing membrane hyaline, forewing with fuscous spot beyond marginal cell. Wing veins brown. Clypeus bulging directly above free margin. Appressed pubescence and erect setae on clypeus and frons silvery. Clypeus with medial glabrous stripe. Distance between hind-ocelli nearly equal to their shortest distance to compound eyes. Pubescence on collar and scutum silvery, on scutum denser laterally than medially. Scutellum slightly convex, with medial impression. Tubercles on metanotum distinct. Propodeal enclosure densely covered with long, erect silvery-white setae, leaving sculpture visible. Length of petiole 1.1× length of flagellomere II. Tomentum moderately dense on metasomal tergum I and II, but very short.

*Female*: Foretarsal rake with 12 long spines. Free clypeal margin with small notch medially.

*Male*: Free clypeal margin entire. Metasomal sterna II–VII mostly glabrous. Metasomal terga V and VI with few bristles. Metasomal sternum VIII notched apically, its lateral margin straight.

**Figure 9.**
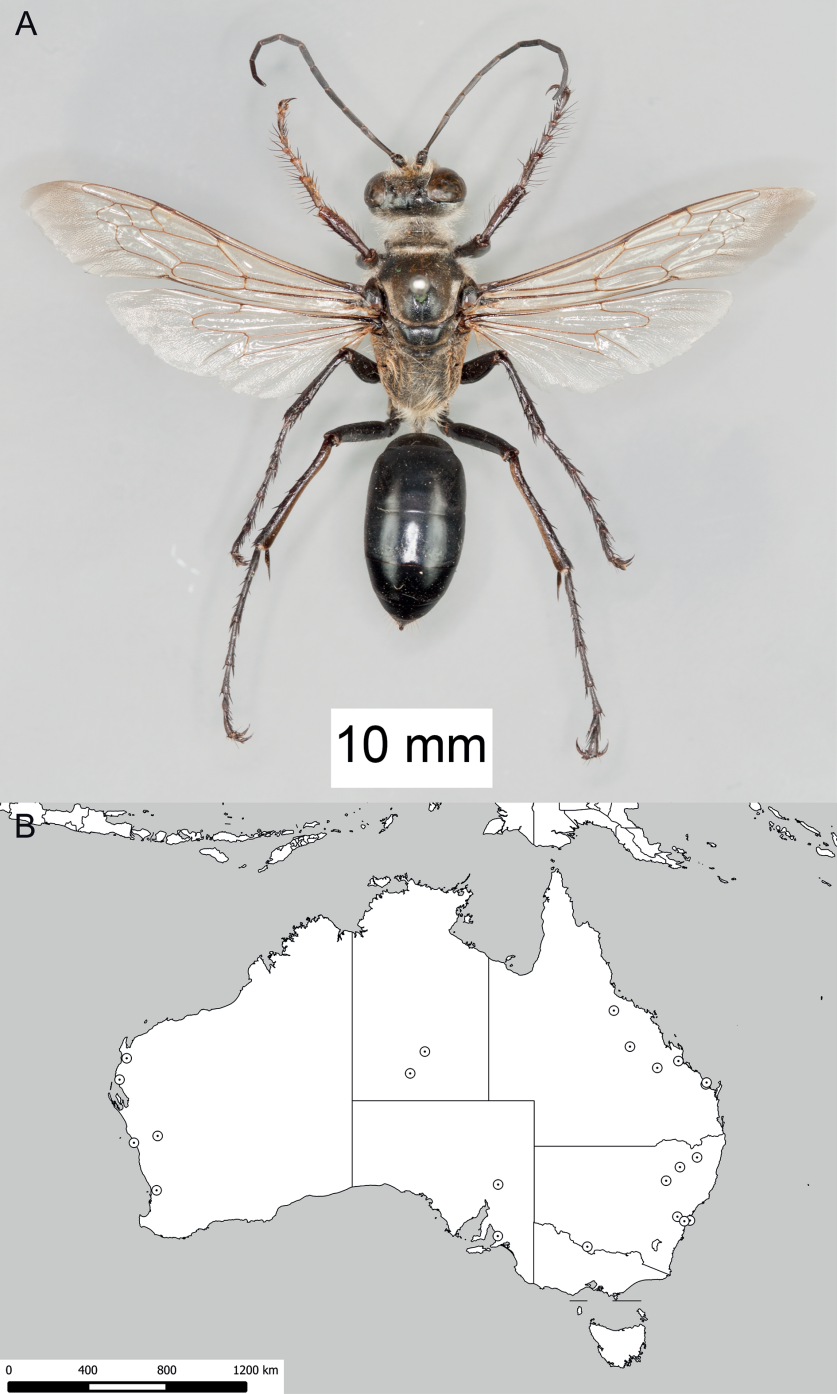
*Sphex carbonicolor*. **A** ♀, habitus **B** collecting localities.

##### Notes on type material.

The type of *Sphex carbonicolor* was not examined, because the character combination listed in the original description (black body, a glabrous area on the clypeus, a bituberculate metanotum, hyaline wings) is almost unique. *Sphex ephippium*, which shares these features, is sufficiently differentiated by its character combination (see below).

#### Sphex decoratus

Taxon classificationAnimaliaHymenopteraSphecidae

F. Smith, 1873

Sphex decoratus F. Smith, 1873: 461, [♀] (as *decorata*, incorrect original termination). Holotype or syntypes: ♀, Australia: northwest coast: no specific locality (BMNH). Presumed holotype examined.

##### Material examined.

*Holotype* (presumed). ♀, **AUSTRALIA:** **WA:** “NW Coast”, (BMNH).

##### Other material.

**AUSTRALIA:** **QLD:** “North Queensland”, 1♀, (BMNH).

##### Diagnosis.

*Sphex decoratus* (of which only the female is known) can be recognized by the combination of markedly raised, distinct tubercles on the metanotum, a mostly orange metasoma of which segment II is black, and a plain propodeal surface. *Sphex sericeus* greatly varies in color and may look superficially similar to *Sphex decoratus*, but differs in having a markedly ridged propodeal dorsum, whereas that of *Sphex decoratus* lacks notable ridges.

##### Description.

*Female*: Body length 24.6–27.2 mm. Body black, but the following are orange: base of mandible, clypeus, scape, pedicel, flagellomere I, flagellomere II–IV above, tegula, subalar area, pronotal lobe, area below anteroventral metapleural pit, petiole, at least parts of metasomal segments I and IV–VI, legs except for base of coxa as well as claw teeth and distal half of claw. Wing membrane yellow near base, hyaline at apex. Wing veins bright orange. Forebasitarsal rake with 11 long spines. Free clypeal margin plain or with insignificant emarginations. Appressed pubescence and erect setae on clypeus and frons golden. Clypeus glabrous medioventrally. Distance between hind-ocelli slightly smaller than their shortest distance to compound eyes. Pubescence on collar and scutum golden, the latter with longer, denser pubescence laterally and posteriorly. Scutellum convex, with distinct medial impression. Tubercles on metanotum distinct. Propodeal enclosure with dense, appressed golden pubescence and sparse, erect golden setae; sculpture completely concealed. Length of petiole twice length of flagellomere II.

*Male*: Unknown.

**Figure 10.**
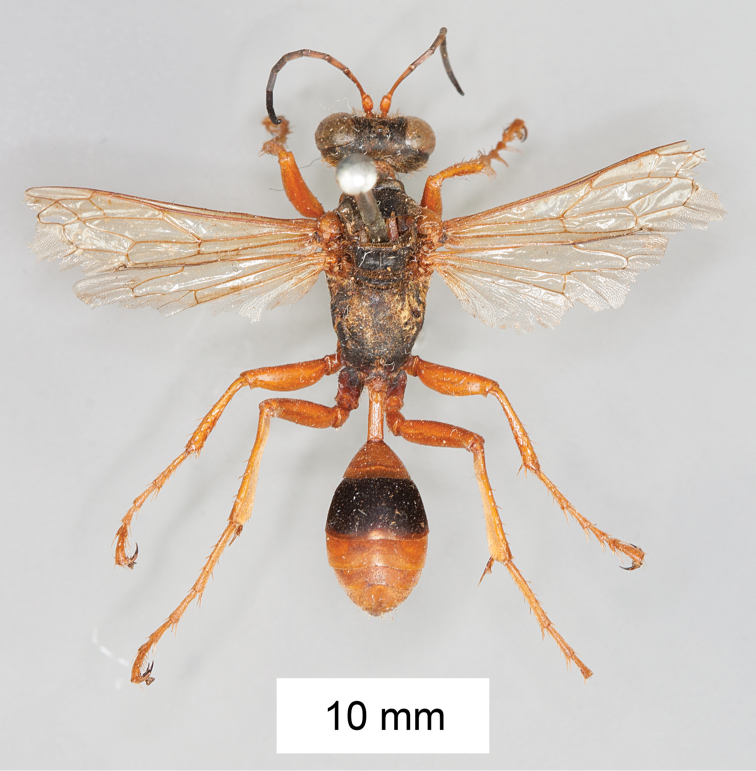
Habitus of *Sphex decoratus*, ♀.

##### Notes on type material.

In the original description, F. Smith did not mention the sex of the holotype. It is listed as a male by Pulawski (2014). However, examination of the type shows that it is clearly a female.

##### Geographic distribution.

Only two specimens of *Sphex decoratus* could be studied, and no specific geographic information is available. The origin of the holotype is given as ‘northwest coast of Australia’ in the original description, that of the other examined specimen as ‘North Queensland’.

#### Sphex ephippium

Taxon classificationAnimaliaHymenopteraSphecidae

F. Smith, 1856

Sphex ephippium F. Smith, 1856: 249, ♀, junior primary homonym of *Sphex ephippius* Linnaeus, 1767 (now in *Sphecodes*). Holotype or syntypes: ♀, Australia: Northern Territory: Port Essington (BMNH). Not examined.

##### Material examined.

**AUSTRALIA:** **[state unknown]:** [no specific locality], 1♀ (BMNH); “North Australia”, 1♀ (BMNH); **NSW:** North Beach, Bellinger R., 1♂, 15.01.1971, D. K. McAlpine (AMS); **NT:** 29 km NW Mataranka, 14°45.5'S, 132°51.1'E, 1♂, 05.04.2008, W. J. Pulawski & G. A. Williams (CAS); Dick Creek, 73 km WSW Timber Creek, 15°50'7"S, 129°54'5"E, 1♂, 12.04.2008, G. Williams & W. Pulawski (AMS); Groote Eylandt, 1♀, 28.01.1925, G. H. Wilkins (BMNH), 1♂, 12.02.1925, G. H. Wilkins (BMNH); 17 miles NNE of Newcastle Waters, 1♀, 13.03.1967, M. S. Upton (ANIC); Port Darwin, 1♂, Dec 02 (ANIC), 1♀ (BMNH); **QLD:** [no specific locality], 2♂, E. Saunders (BMNH); “Mid Queensland”, 1♂ (BMNH); Bamaga, Cape York, 1♀, 26.03.1964, I. F. B. Common & M. S. Upton (ANIC); Brisbane, 1♂, Feb–Mar 43, E. F. Riek (ANIC); Bundaberg, 1♀, 01.02.1971, H. Frauca (ANIC); Byfield State Forest, 1♀, 05.01.1976, G. Daniels (AMS); Cairns, 1♂, 01.01.1902 (ANIC); Cape York, 1♀, 01.08.1986, N. W. Rodd (AMS), 1♂, 05.08.1986, N. W. Rodd (AMS), 1♀, 29.05.1991, N. W. Rodd (AMS); Chili Beach near Portland Roads, 2♂, 25.08.1983, N. W. Rodd (AMS), 1♀, 1♂, 26.08.1983, N. W. Rodd (AMS); Claudie River, 3 miles W of Mount Lamond, 1♀, 13.01.1972, D. K. McAlpine & G. A. Holloway (AMS); 8 km W of Cooktown, 1♀, 17.07.1982, N. W. Rodd (AMS);Dunk Island, 1♀, 31.12.1949-05.01.1950, G. B. (ANIC); Eidsvold, 1♂, 01.01.2023, Mackerras (AMS); Hammond Island, 1♀, 14.03.1963, R. J. Docherty (BMNH); Mackay, 1♀, 1947, A. Marriage (AMS); Prince of Wales Island, 1♀, 13.02.1975, Torres (AMS); Thursday Island, 1♀, 14.01.1939 (BMNH); Walkers Creek, 35 km NNE of Normanton, 1♀, 02.01.1990, M. S. & B. J. Moulds (AMS); **SA:** Adelaide, 1♀ (ZMB); **WA:** Bullsbrook, 1♂, 13.01.1966, O. W. Richards (BMNH); 3 km NWbyW of Millstream, 21°34'S, 117°03'E, 1♂, 05.04.1971, E. F. Riek (ANIC).

##### Diagnosis.

*Sphex ephippium* is unique in the combination of a tuberculate metanotum, appressed golden pubescence on the clypeus interspersed with longer dark setae, and long, silvery-white pubescence concealing the sculpture of the propodeal enclosure.

##### Description.

Body black. Wing membrane darkened at base, with fuscous band at apex. Wing veins dark brown. Appressed pubescence on clypeus and frons golden, erect setae on clypeus black and on frons golden. Clypeus with medial glabrous stripe. Pubescence on collar and scutum silvery, denser laterally and posteriorly on latter. Scutellum convex. Metanotum markedly raised, tubercles indistinct. Propodeal enclosure densely covered with long, silvery-white pubescence aligned anteriorly, mostly concealing sculpture. Tomentum moderately dense on metasomal tergum I and II.

*Female*: Body length 22.8–30.6 mm. Free clypeal margin slightly scoop-shaped, with indistinct emarginations. Distance between hind-ocelli slightly smaller than their shortest distance to compound eyes. Forebasitarsal rake with nine long spines. Scutellum without impression. Length of petiole 1.7× length of flagellomere II. Tomentum absent on apical half of metasomal tergum II.

*Male*: Body length 21.2–24.6 mm. Free clypeal margin truncate, concave towards center. Distance between hind-ocelli 1.15× their shortest distance to compound eyes. Scutellum with or without medial impression. Length of petiole almost twice length of flagellomere II. Metasomal terga V and VI covered with black bristles. Metasomal sterna II–V with a few erect black setae, VI–VIII more densely covered with silvery setae and brown ones with silvery tips. Metasomal sternum VIII entire, its lateral margin straight.

**Figure 11.**
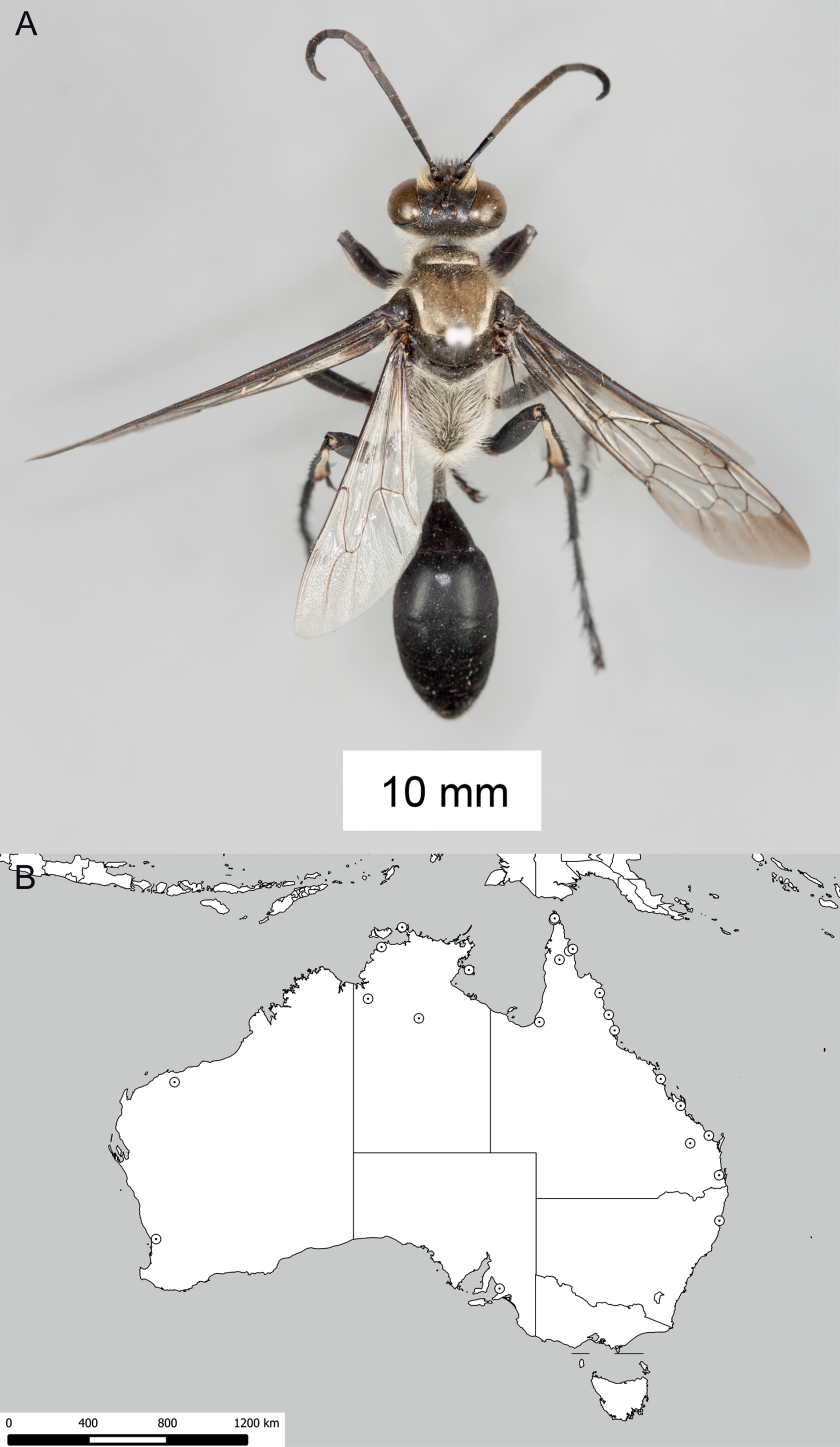
*Sphex ephippium*. **A** ♂, habitus **B** collecting localities.

##### Notes on type material.

The type of *Sphex ephippium* was not examined, because the character combination in the original description (darker setae among the golden pubescence of the face, dense silvery pubescence on propodeal enclosure) is sufficient to unambiguously identify this species.

#### Sphex finschii

Taxon classificationAnimaliaHymenopteraSphecidae

Kohl, 1890

Sphex finschii Kohl, 1890: 412, sex not indicated (as *Finschii*, incorrect original capitalization). Lectotype: ♂, Papua New Guinea: New Britain: no specific locality (ZMB), designated by Hensen 1991: 21. Lectotype examined.

##### Material examined.

Lectotype. ♂, **PAPUA NEW GUINEA:** **[province unknown]:** New Britain [no specific locality] (ZMB).

##### Other material.

**INDONESIA:** **Papua:** Yerelua, 1♂, 26.07.1998, Balke & Konyorah (NHMW). **PAPUA NEW GUINEA:** **Bougainville Province:** Bougainville Island, 1♂, 1908, L. Cohn (ZMB), 1♂, 26.07.1923, E. O. Pockley (AMS); Buoni, Bougainville Island, 1♀, 20.10.1922, E. O. Pockley (AMS); Sininai, Bougainville Island, 1♀, 26.09.1922, E. O. Pockley (AMS), 1♀, 26.09.1923, E. O. Pockley (AMS); **Central Province:** Port Moresby, 1♀, 25.02.1939, C. Lupson (AMS); **East New Britain Province:** Vudanplata, 15 km W Keravat, 4°12'S, 152°00'E, 1♀, 1♂, 05.-13.06.2003, T. Osten (ZMB); Vunabaur, 30 km S Kokopo, 4°28'S, 152°19'E, 1♀, 1♂, 07.-12.06.2003, T. Osten (ZMB); **Oro Province:** Mount Lamington, 1♀, May 1927, C. T. McNamara (AMS); **West New Britain Province:** Lamavoro, 10 km S Hoskins, 5°28'S, 150°26'E, 1♂, 21.06.2003, T. Osten (ZMB); Makasili, 20 km E Hoskins, 5°28'S, 150°26'E, 2♀, 2♂, 19.-24.06.2003, T. Osten (ZMB).

##### Diagnosis.

This species is well characterized by its wings, which are largely hyaline but darkened at the base. It shares this trait with three other species. One of them is *Sphex luctuosus* that can be distinguished by claw teeth perpendicularly oriented to the inner margin of the claw, a character of the *Sphex resplendens* group (as a member of the *Sphex argentatus* group, *Sphex finschii* possesses obliquely oriented claw teeth). The second species, *Sphex argentatus*, is identifiable by its distinctly tuberculate metanotum, while the tubercles are indistinct in *Sphex finschii*. The third species, *Sphex fortunatus*, has erect setae that are uniformly silvery on its clypeus, whereas *Sphex finschii* has black setae.

**Figure 12.**
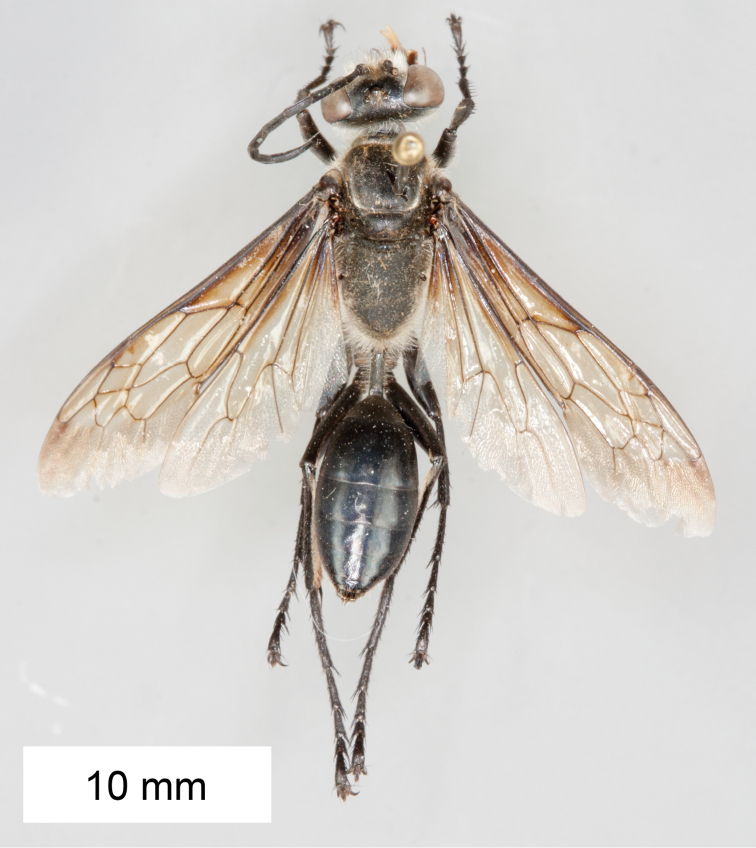
Habitus of *Sphex finschii*, ♂.

##### Description.

Body black. Costal cell of fore- and hindwing dark. Base of forewing membrane darkened, sometimes up to the medial cell’s distal margin, fuscous band at apex. Hindwing membrane darkened mainly near base. Wing veins dark brown to black. Appressed pubescence on clypeus and frons silvery, erect setae on clypeus black and on frons silvery. Clypeus with glabrous ridge dorsomedially. Pubescence on mesosoma silvery, denser laterally on scutum. Scutellum convex, with shallow medial impression. Metanotum markedly raised, tubercles indistinct. Pubescence on propodeal enclosure sparse and fine, leaving sculpture fully visible. Length of petiole nearly 1.7× length of flagellomere II. Tomentum moderately dense on metasomal tergum II.

*Female*: Body length 21.6–29.2 mm. Forebasitarsal rake with nine long spines. Free clypeal margin with 3 faint lobes medially, distance between them less than 1/8 length of flagellomere II. Distance between hind-ocelli 0.9× their shortest distance to compound eyes. Tomentum moderately dense on metasomal tergum I.

*Male*: Body length 20.4–25.2 mm. Free clypeal margin truncate, concave medially. Area adjacent to it glabrous. Distance between hind-ocelli 1.1× their shortest distance to compound eyes. Tomentum dense on metasomal tergum I. Metasomal terga V and VI with few black bristles. Metasomal sterna II–IV mostly glabrous, V–VII with increasingly dense fringes of dark and silvery setae laterally on apical margin. Metasomal sternum VIII entire, covered with silvery pubescence, its lateral margin straight.

##### Geographic distribution.

Although *Sphex finschii* is listed by Cardale (1985) and Hensen (1991), no specimen was found that has actually been collected in Australia. Individuals examined during this study come from New Guinea, the Solomon Islands, or Indonesia.

#### Sphex modestus

Taxon classificationAnimaliaHymenopteraSphecidae

F. Smith, 1856

Sphex modestus F. Smith, 1856: 248, ♀ (as *modesta*, incorrect original termination). Holotype or syntypes: ♀, Australia: no specific locality (BMNH). Not examined.
Sphex bannitus Kohl, 1890: 62, ♀. Holotype: ♀, New Holland, now Australia: no specific locality (ZMB). Synonymized with *Sphex modestus* by Turner (1910: 346). Holotype examined.

##### Material examined.

*Holotype* (of *Sphex bannitus*). ♀, **AUSTRALIA:** **[state unknown]:** [no specific locality] (ZMB).

##### Other material.

**AUSTRALIA:** **[state unknown]:** [no specific locality], 3♀, 2♂ (ZMB); “NW Australia, Carlshalton”, 2♀, E. Clement (ZMB); **NSW:** 56 miles W of Cobar, Baznatos Tank, 1♀, 01.01.1966, O. W. Richards (BMNH); Haystack Ridge near Mount Tomah, 1♀, 29.11.1977, N. W. Rodd (AMS); 35 km N of Menindee, 1♂, 26.11.1988, N. W. Rodd (AMS); 35 km WNW Menindee, 32°12.4'S, 142°10.8'E, 2♂, 18.12.2011, V. Ahrens & W. J. Pulawski (CAS); Mount York, Blue Mountains, 1♀, 29.01.1982, N. W. Rodd (AMS); Pooncarie, 1♂, 26.11.1991, N. W. Rodd (AMS), 1♂, 27.11.1991, N. W. Rodd (AMS); Round Hill Nature Reserve, 1♀, 27.12.1976, G. Daniels (AMS); 112 km N of Wentworth, 1♂, 28.11.1991, N. W. Rodd (AMS); **NT:** 20 km W of Barkly Homestead, 1♀, 18.06.1989, N. W. Rodd (AMS); Dick Creek, 73 km WSW Timber Creek, 15°50'7"S, 129°54'5"E, 1♂, 12.04.2008, G. Williams & W. Pulawski (AMS); Hermannsburg, 1♂, Leonhardi (ZMB); Keep River National Park, 15°54'1"S, 129°04'4"E, 1♀, 11.04.2008, G. Williams & W. Pulawski (AMS); Port Darwin, 2♂ (BMNH); 131 km N Tennant Creek, 18°28.8'S, 133°52.1'E, 1♀, 01.04.2008, W. J. Pulawski & G. A. Williams (CAS); **QLD:** “Mid Queensland”, 1♀, 1♂ (BMNH); Mission Beach, 1♀, 16.08.1975, G. O’Reilly (AMS); **SA:** 20 km S Adelaide, 1♀, 01.01.1999, B. N. Danforth (CAS); Adelaide, 1♂ (ZMB); Cocata Conservation Park, 33°17.0'S, 135°19.7'E, 1♂, 03.01.2011, V. Ahrens & W. J. Pulawski (CAS); Port Augusta Botanic Garden, 32°27.6'S, 137°45.1'E, 1♂, 25.01.2011, V. Ahrens & W. J. Pulawski (CAS); 27 km WSW Whyalla, 33°06.5'S, 137°19.0'E, 1♂, 28.12.2010, V. Ahrens & W. J. Pulawski (CAS); Wilpena Pound Resort, 1♀, 18.01.1976, M. S. Moulds (AMS); **WA:** Bullsbrook, 2♀, 12.12.1951, H. F. Broadbent (BMNH), 1♀, 12.01.1966, O. W. Richards (BMNH), 1♀, 3♂, 13.01.1966, O. W. Richards (BMNH), 1♀, 13.02.1966, O. W. Richards (BMNH); 11 km S Geraldton, 28°51.6'S, 114°38.8'E, 1♀, 02.11.2008, V. Ahrens & W. J. Pulawski (CAS); 10 km S Jurien Bay, 30°22.8'S, 115°04.5'E, 4♀, 31.10.2008, V. Ahrens & W. J. Pulawski (CAS); 15 km S Kalbarri, 27°50.4'S, 114°09.0'E, 3♀, 04.11.2008, V. Ahrens & W. J. Pulawski (CAS); Kalbarri National Park: Ross Graham Lookout, 27°48.6'S, 114°28.3'E, 1♀, 07.11.2008, V. Ahrens & W. J. Pulawski (CAS); Marloo Station, 2♀, Mar 35, A. Goerling (ZMB); W of New Norcia, 1♂, 12.01.1966, O. W. Richards (BMNH); Shire of Dandaragan, Highway #1, 31.1 km S of Cataby, 1♂, 07.01.2010, L. Breitkreuz (ZMB); Shire of Northampton, Kalbarri National Park, 27°40'39"S, 114°16'18"E, 1♀, 09.01.2010, L. Breitkreuz (ZMB); Shire of Waroona, Yalgorup National Park, 32.880879°S, 115.676464°E, 1♂, 25.01.2010, S. Krause (ZMB); Shire of Waroona, Yalgorup National Park, 32.880160°S, 115.682545°E, 1♀, 27.01.2010, S. Krause (ZMB); Southern Cross, 1♂, 10.-22.01.1936, R. E. Turner (BMNH); Urawa Nature Reserve ca 5 km N Mullewa, 28°29.6'S, 115°29.5'E, 3♂, 11.11.2008, V. Ahrens & W. J. Pulawski (CAS); Waroona, 1♀, 26.12.1908, G. F. Berthoud (BMNH), 1♀, 11.01.1909, G. F. Berthoud (BMNH).

##### Diagnosis.

*Sphex modestus* is unique among the Australian *Sphex* in the combination of the following characteristics: claw teeth obliquely oriented to inner claw margin, wing membrane with a yellow tinge towards their base, appressed pubescence on clypeus silvery-white interspersed with erect black setae, legs and metasoma entirely black or dark brown, and propodeal enclosure not concealing sculpture. *Sphex pretiosus* is superficially similar, but, among other things, the color of its erect setae on the clypeus matches that of the silvery and silvery-golden appressed pubescence, whereas the erect setae on the clypeus of *Sphex modestus* are black.

##### Description.

**Figure 13.**
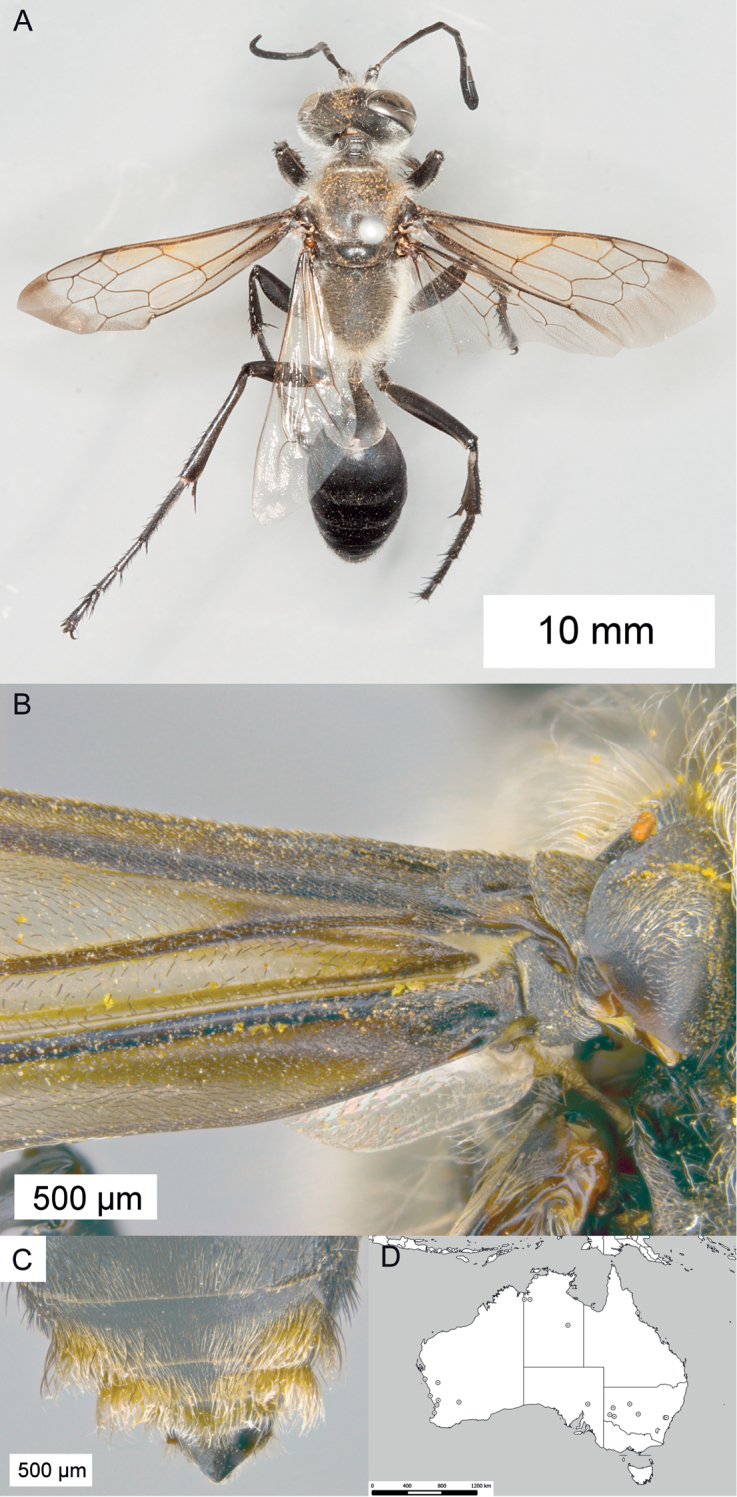
*Sphex modestus*. **A** ♂, habitus **B** darkened base of forewing **C** ♂, ventral view of metasomal sterna IV–VIII, with V–VII carrying dense fringes of setae **D** collecting localities.

Body black. Forewing membrane dark at the very base (Fig. 13B), with yellow tinge in basal half, remainder hyaline and with fuscous band at apex. Wing veins orange, dark brown near very base and around marginal cell of forewing. Clypeus with medial glabrous stripe. Appressed pubescence on clypeus and frons silvery, erect setae on clypeus black and on frons silvery. Pubescence on collar, scutum, scutellum and metanotum silvery, on scutum denser laterally and posteriorly. Scutellum convex, with medial impression. Tubercles on metanotum indistinct. Pubescence on propodeal enclosure silvery-white to dirty beige, not completely concealing sculpture. Length of petiole 1.8× length of flagellomere II.

*Female*: Body length 19.6–24.6 mm. Forebasitarsal rake with 10 long spines. Free clypeal margin with two faint lobes medially, distance between them less than 1/8 length of flagellomere II. Distance between hind-ocelli 1.25× their shortest distance to compound eyes. Tomentum sparse on metasomal tergum I, absent on tergum II.

*Male*: Body length 20.6–26.2 mm. Free clypeal margin truncate, slightly concave towards center. Distance between hind-ocelli 1.4× their shortest distance to compound eyes. Tomentum dense on metasomal tergum I, moderately dense on tergum II. Metasomal terga V and VI covered with black bristles. Metasomal sterna II–VII with increasingly dense silvery pubescence, forming dense fringes laterally on sterna V–VII (Fig. 13C). Metasomal sternum VIII entire, with rather sparse silvery pubescence, its lateral margin straight.

##### Notes on type material.

The type of *Sphex modestus* F. Smith, 1856 was not examined, but we did study the type of *Sphex bannitus* Kohl, 1890, which was synonymized with *Sphex modestus* by R. Turner (1910).

#### Sphex sericeus

Taxon classificationAnimaliaHymenopteraSphecidae

(Fabricius, 1804)

Sphex aurulentus Fabricius, 1793: 201, sex not indicated, junior primary homonym of *Sphex aurulentus* Fabricius, 1787 (now in *Liris*). Holotype or syntypes: India: Tranquebar (depository unknown: van der Vecht 1961: 30). Not examined.
Pepsis sericeus Fabricius, 1804: 211, sex not indicated (as *sericea*, incorrect original termination). Lectotype: ♀, “in maris pacifici Insulis” (ZMUC), designated by van der Vecht 1961: 30. Not examined.

##### Material examined.

**AUSTRALIA:** **[state unknown]:** [no specific locality], 1♂ (BMNH); **NT:** Port Darwin, 5♀, 6♂ (BMNH); **QLD:** [no specific locality], 1♂ (ANIC); “North Queensland”, 1♀ (BMNH); Allingham near Mackay, 1♂, 25.10.1984, N. W. Rodd (AMS); Archer Point, 10 km S of Cooktown, 1♂, 05.09.1983, N. W. Rodd (AMS); Barron Rivers, 1♂ (ANIC); Biloela, 1♂, 08.12.1926, E. Ballard (BMNH); Blackdown Tableland Expedition Range, 1♀, 08.01.1976, G. Daniels (AMS); Cairns, 2♂, 1919, Jarvis (ANIC); Cape Ferguson, Townsville, 1♀, 3♂, 21.-22.03.1978, N. Duke (ANIC); Cape York, 1♀, 02.06.1985, N. W. Rodd (AMS), 1♀, 3♂, 02.08.1986, N. W. Rodd (AMS), 2♂, 28.05.1991, N. W. Rodd (AMS), 1♀, 31.05.1991, N. W. Rodd (AMS), 1♂, May 1902 (BMNH), 1♂ (BMNH); Cooktown, 1♂, 18.07.1982, N. W. Rodd (AMS); Duaringa, 1♂, 26.12.1946, C. W. Smith (AMS); Ellis Beach N of Cairns, 1♀, 12.05.1967, D. H. Colless (ANIC); Endeavour River, Cooktown, 1♂, 05.10.1922, F. P. Spry (ANIC); Gayndah, 2♀ (AMS); 5 km SEbyS of Helenvale, 15°44'S, 145°15'E, 1♀, 25.08.1992, J. Cardale & P. Zborowski (ANIC); Inkerman, 1♂, W. Stalker (BMNH); Mackay, 1♂, May 1893 (BMNH), 1♀ (BMNH); Mitchell River Settlement, 1♀, 01.04.1969, A. L. Dyce (ANIC); 4 miles NE of Mount Lamond, 1♀, 02.12.1971, D. K. McAlpine & G. A. Holloway (AMS), Quarantine Bay near Cooktown, 1♂, 13.06.1985, N. W. Rodd (AMS); Redlynch, 1♂, Dec 38, H. F. Sternitzky (BMNH); Thursday Island, 1♂, 14.01.1939 (BMNH); Townsville, 1♂, 03.04.1902, F. P. Dodd (BMNH); Westwood, 1♂, 09.12.1923, A. N. Burns (ANIC); **WA:** Baudin Island, 1♀ (BMNH), Roeburne, 1♀ (BMNH). **INDONESIA:** **Papua:** Yerelua, 1♂, 26.07.1998, Balke & Konyorah (NHMW). **PAPUA NEW GUINEA:** **Western Province:** Daru Island, 1♀, 05.05.–18.05.1921, E. O. Pockley (AMS).

##### Diagnosis.

*Sphex sericeus* is unique among its Australian congeners in having three to four broad, continuous transverse ridges on the propodeal enclosure which are even visible through the pubescence. Female specimens of *Sphex rugifer*, *Sphex darwiniensis* and *Sphex imporcatus* possess vaguely similar structures, but those are much finer. *Sphex rugifer* also has approximately 20 ridges on the propodeal enclosure, while those of *Sphex darwiniensis* and *Sphex imporcatus* are discontinuous in the center.

##### Description.

Clypeus with medial glabrous stripe. Pubescence on scutum slightly denser laterally. Scutellum slightly convex, with shallow medial impression near posterior margin. Tubercles on metanotum distinct. Propodeal enclosure with three to four broad, continuous transverse ridges; propodeal pubescence not concealing sculpture. Tomentum moderately dense on metasomal terga I and II.

*Female*: Body length 20.2–25.7 mm. Body black, but the following are orange: basal half of mandible, clypeus, scape, pedicel, parts of flagellomere I, scutellum, metanotum, petiole, metasomal segment I, anterior two thirds of metasomal segment II, legs excluding claw teeth and distal half of claw. Wing membrane with yellow tinge and markedly fuscous band at apex. Wing veins orange, dark near apex. Forebasitarsal rake with nine long spines. Free clypeal margin conspicuously notched medially, bulging above. Appressed pubescence and erect setae on clypeus and frons golden. Distance between hind-ocelli 0.7× their shortest distance to compound eyes. Pubescence on mesosoma golden. Length of petiole 1.4× length of flagellomere II.

*Male*: Body length 16.8–23.6 mm. Wing membrane largely hyaline, with slight yellow tinge around costal cell. Forewing with faint fuscous band at apex. Wing veins dark brown to orange. Free clypeal margin concave towards center. Appressed pubescence and erect setae on clypeus and frons silvery-white. Distance between hind-ocelli nearly equal to their shortest distance to compound eyes. Pubescence on mesosoma silvery. Length of petiole 1.6×–1.7× length of flagellomere II. Metasomal terga V and VI with few silvery bristles. Metasomal sterna II–VIII largely glabrous. Metasomal sternum VIII entire, its lateral margin sometimes slightly concave.

**Figure 14.**
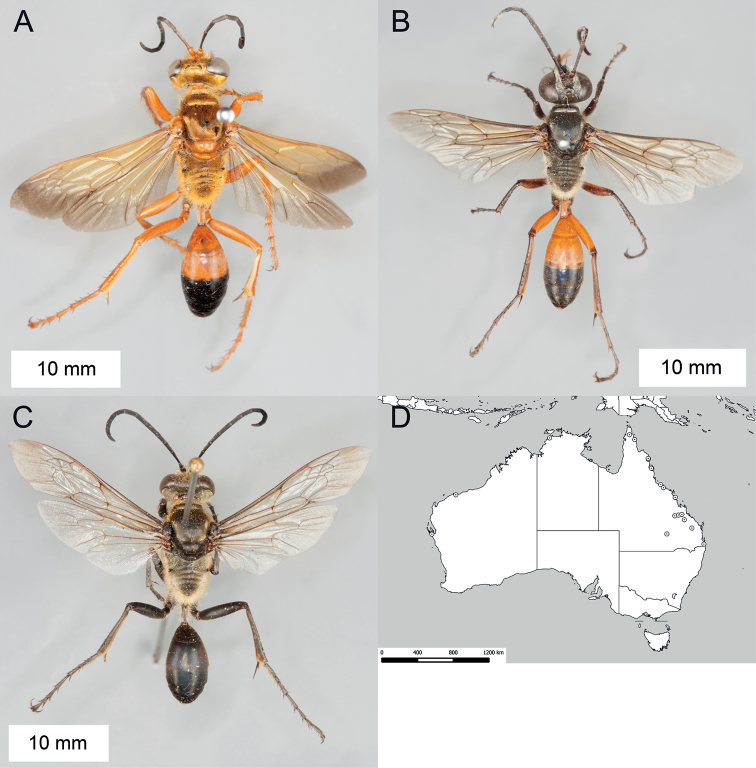
*Sphex sericeus*. **A–C** habitus **A** ♀ **B** ♂, collected in Darwin, NT **C** ♂, collected in Westwood, QLD **D** collecting localities.

##### Variation.

Males of this species markedly vary in color, though most Australian specimens belong to either one of two forms: the first form has the following parts bright red: anterior side of midfemur, entire hindfemur, petiole, metasomal segments I and II (Fig. 14B), while the body of the second form is entirely black (Fig. 14C). The color of the females seems to be more or less uniform among the collecting localities.

##### Notes on type material.

The types of *Sphex sericeus* and its synonyms were not examined, because the character combination in the redescription by Kohl (1890) (broad transverse ridges on propodeal enclosure, bituberculate metanotum) is sufficient to unambiguously identify this species. Van der Vecht (1961) noted that the lectotype of this species agrees with the species current (i.e., Kohl’s) interpretation.

### Species group of *Sphex resplendens*

This group currently contains ten species, including two new ones described in this paper. In accordance with Hensen (1991), species of this group have the distal claw teeth oriented perpendicularly to the claw, specifically to the point where the claw tooth emerges. In contrast, all species of the *Sphex argentatus* and the *Sphex subtruncatus* group possess claw teeth oriented obliquely to the claw. There are only few specimens where the orientation of the claw teeth is ambiguous, and most of these cases can probably be attributed to the effects of deterioration during the wasps’ lives.

There are a few other characters which are often found in species of the *Sphex resplendens* group, like a conspicuously flat and shining scutellum without impressions in the female. However, these traits are not universal for all members and also exist in a few species that, based on the alignment of their claw teeth, clearly belong in one of the other groups. Another feature Hensen (1991) cited in his diagnosis of the group was the absence of antennal placoids in males, but this was found out not to be a universal characteristic among the members. Rather, placoids are often only less distinct in these species, which makes a differentiation difficult. Therefore, attributes other than the alignment of the claw teeth have been given less importance for species group determination than in Hensen’s key (1991).

#### Sphex darwiniensis

Taxon classificationAnimaliaHymenopteraSphecidae

R. Turner, 1912

Sphex darwiniensis R. Turner, 1912: 56, ♀. Holotype or syntypes: ♀, Australia: Northern Territory: Darwin (BMNH). Presumed holotype examined.

##### Material examined.

*Holotype* (presumed). ♀, **AUSTRALIA:** **NT:** Port Darwin, 1911, F. P. Dodd (BMNH).

The collecting locality is shown in Fig. 20B.

##### Diagnosis.

*Sphex darwiniensis* (of which only the female is known) differs from all other Australian *Sphex* in having the features diagnostic for the *Sphex resplendens* group combined with orange legs and metasoma, partly yellowish wings and approximately ten distinct, fine transverse ridges on the propodeal enclosure. The similar *Sphex rugifer* has a uniformly dark forewing, black legs and a black petiole. *Sphex rhodosoma*, in contrast to both the former species, is almost completely orange, including the entire mesosoma.

##### Description.

*Female*: Body length 22.2 mm. Body black, but the following are orange: apical half of mandible, clypeus, scape, pedicel, base of flagellomere I, legs from trochanter onward except for claw teeth and apical half of claw, tegula, subalar area, petiole, gaster. Wing membrane yellow, darkened beyond submarginal cell I. Wing veins orange, brown in darkened area of wing. Forebasitarsal rake with seven long spines. Free clypeal margin medially with two lobes which are slightly convex above, distance between them less than 1/8 length of flagellomere II. Appressed pubescence and erect setae on clypeus and frons silvery-white. Clypeus with medial glabrous stripe. Distance between hind-ocelli slightly smaller than their shortest distance to compound eyes. Pubescence on collar and scutum silvery-white, the latter glabrous except laterally and posteriorly. Scutellum flat, shiny, without impressions. Propodeal enclosure with short, silvery-white pubescence and approximately ten distinct transverse ridges which are interrupted medially, sculpture almost completely visible. Length of petiole approximately 0.9× length of flagellomere II. Tomentum on metasomal terga I and II sparse and short.

*Male*: Unknown.

**Figure 15.**
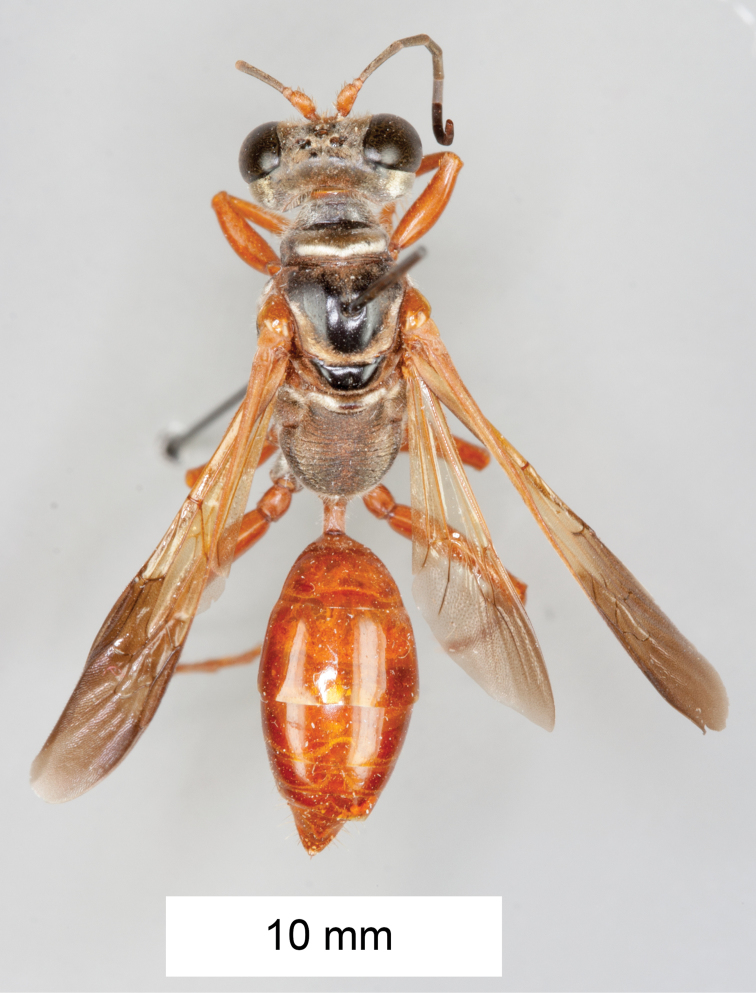
Habitus of *Sphex darwiniensis*, ♀.

#### Sphex fumipennis

Taxon classificationAnimaliaHymenopteraSphecidae

F. Smith, 1856

Sphex fumipennis F. Smith, 1856: 249, ♀, ♂. Syntypes: Australia: South Australia: Adelaide (BMNH). One syntype examined.

##### Material examined.

Syntype. ♀, **AUSTRALIA:** **SA:** Adelaide (BMNH).

##### Other material.

**[COUNTRY UNKNOWN]:** **[state unknown]:** [no specific locality], ♀, 21.06.1959 (AMS). **AUSTRALIA:** **[state unknown]:** [no specific locality], 3♀, 2♂ (BMNH); **NSW:** Avoca Beach, 1♀, 11.12.1987, S. Hunter (AMS); Bathurst, 1♀, 02.02.1965, C. H. Smithers (AMS); 6 km NE of Bilpin, Blue Mountains, 3♂, 11.01.1980, N. W. Rodd (AMS); 7 km N of Bilpin, Blue Mountains, 1♀, 23.02.1979, N. W. Rodd (AMS), 1♀, 06.03.1979, N. W. Rodd (AMS); 7 km NE of Bilpin, Blue Mountains, 1♂, 28.12.1981, N. W. Rodd (AMS); 10 km N of Broken Hill, 2♀, 1♂, 11.03.2001, M. Ohl (ZMB); Cabramatta, 1♂, 10.03.1963, E. C. Corbet (BMNH); Cheltenham, 1♀, 25.03.1950 (AMS); 25 km SW of Dubbo, 1♀, 08.11.1981, N. W. Rodd (AMS); 30 km N of Euston, 1♂, 28.11.1988, N. W. Rodd (AMS); Kurnell, 3♂, 16.12.1978, D. A. Doolan (AMS); Lane Cove, 1♂, 07.01.1944 (AMS), 1♀, 24.02.1945 (AMS); Lord Howe Island, 1♂, 22.12.1921, A. Musgrave (AMS); Maire Road, Lord Howe Island, 1♂, 22.12.1921, A. Musgrave (AMS); 35 km N of Menindee, 1♂, 26.11.1988, N. W. Rodd (AMS); Middle Beach Road, Lord Howe Island, 1♀, 26.03.1979, T. Kingston (AMS), 1♀, 14.04.1979, T. Kingston (AMS); Mount Annan, Botanic Garden, 1♂, 12.12.2007, L. von Richter (AMS); Mount Kaputar National Park, 1♀, 25.03.1978, G. Daniels (AMS); Mount Keira, 1♀, 22.12.1986, G. A. & A. Holloway (AMS); Mount Tomah, Blue Mountains, 1♀, 01.01.1981, N. W. Rodd (AMS); Murray Beach, Jervis Bay, 1♀, 18.02.1987, N. W. Rodd (AMS); 50 km N of Pooncarie, 2♂, 26.11.1988, N. W. Rodd (AMS); Pooncarie, 3♂, 26.11.1991, N. W. Rodd (AMS), 1♀, 28.11.1992, N. W. Rodd (AMS); Round Hill Nature Reserve, 2♀, 25.10.1977, G. Daniels (AMS); South Durras, 35.40S, 150.17E, 1♀, 18.03.2001, M. Ohl (ZMB); Sydney, 1♀, 01.02.1913, A. Musgrave (AMS); Vincentia, Jervis Bay, 2♂, 17.02.1987, N. W. Rodd (AMS); 112 km N of Wentworth, 2♀, 28.11.1991, N. W. Rodd (AMS); Winston Hills, 1♂, 25.02.1985, C. A. P. Urquhart (AMS); Woronora, 1♀, 03.01.1982, M. L. Bason (AMS), 1♀, 27.03.1982, M. L. Bason (AMS), 1♀, 04.05.1982, M. L. Bason (AMS); **NT:** 20 km W of Barkly Homestead, 1♀, 18.11.1989, N. W. Rodd (AMS); 41 miles Bore Barkly Hwy, 1♂, 10.07.1989, N. W. Rodd (AMS); Port Darwin, 2♀, 8♂ (BMNH) **QLD:** “Mid Queensland”, 1♀, 2♂ (BMNH); “North Queensland”, 2♀, 2♂ (BMNH); Archer Point, 10 km S of Cooktown, 1♀, 3♂, 05.09.1983, N. W. Rodd (AMS); Bramston Beach, 1♀, 26.08.1987, N. W. Rodd (AMS); Cape York, 1♀, 05.06.1985, N. W. Rodd (AMS); Capricorn Group, NW Islet, 1♀, Dec 25, A. Musgrave (AMS); Claudie River near Mount Lamond, 1♀, 12.10.1974, G. Daniels (AMS); Cooktown, 1♀, 18.07.1982, N. W. Rodd (AMS); Grantleigh, 1♀, 20.11.1978, R. Eastwood (AMS); Hammond Island, 1♀, 10.10.1964, R. J. Docherty (BMNH); Herberton, 1♀, 18.08.1984, N. W. Rodd (AMS); Mission Beach, 1♀, 16.08.1975, G. O’Reilly (AMS); Poison Creek Road, Cooktown, 1♂, 16.06.1985, N. W. Rodd (AMS); 64 km SW of Ravenshoe, 1♂, 08.01.1976, D. K. McAlpine (AMS); 10 km W of Torrens Creek township, E of Hughenden, 1♀, 07.01.1987, M. S. & B. J. Moulds (AMS); Yeppoon, 1♂, 17.11.1978, R. Eastwood (AMS); **SA:** Adelaide, 1♂ (ZMB); Anajatra, Mann Ranges, 1♀, 10.-11.05.1983, G. A. Holloway (AMS); Lake Gilles Conservation Park, 3♂, 01.02.1995, L. Packer, M. Schwarz, P. Hurst, Y. Pamula (ZMB), 1♂, Mar 1995, L. Packer, Y. Pamula (ZMB); Middleback Ranges, 1♂, Mar 1995, M. Schwarz, B. Kranz (ZMB); Wilpena Pound Resort, 1♀, 18.01.1976, M. S. & B. J. Moulds (AMS); **WA:** Bunbury, 1♂, 01.01.1961, A. Snell (AMS); 15 km S Kalbarri, 27°50.4'S, 114°09.0'E, 1♂, 04.11.2008, V. Ahrens & W. J. Pulawski (CAS); 26 km NE of Laverton, 28°28'43"S, 122°29'09’’, 1♀, 28.09.2005, L. Packer (ZMB); Marloo Station, 1♀, 1♂, 01.01.1935, Gebr. Goerling (ZMB), 1♀, 01.04.1935, A. Goerling (ZMB); 50 km E of Mullewa, 1♀, 03.09.1981, G. A. Holloway (AMS); Shire of Waroona, Yalgorup National Park, 32°52'51"S, 115°40'35"E, 1♂, 25.01.2010, L. Breitkreuz (ZMB); Shire of Waroona, Yalgorup National Park, 32.839339°S, 115.639100°E, 1♀, 26.01.2010, S. Krause (ZMB); Swan River, 1♂, Dämel (ZMB); Urawa Nature Reserve ca 5 km N Mullewa, 28°29.6'S, 115°29.5'E, 1♂, 11.11.2008, V. Ahrens & W. J. Pulawski (CAS); Waroona, 1♂, 28.02.1909, G. F. Berthoud (BMNH); Wilga, 1♀, 1♂, 19.01.1980, K. Carnaby (AMS), 2♂, 14.01.1983, K. & E. Carnaby (AMS); Yallalong Homestead, 1♂, 29.11.1999, M. S. Moulds & M. Humphrey (AMS).

##### Diagnosis.

**Figure 16.**
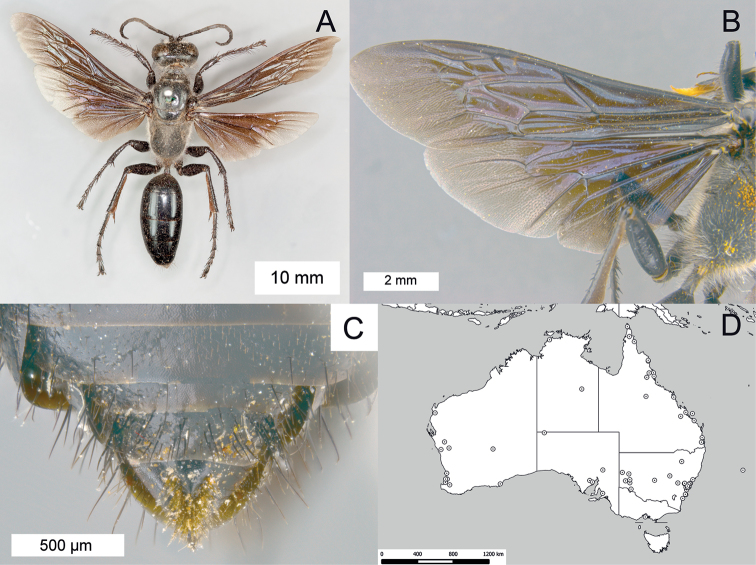
*Sphex fumipennis*. **A** ♀, habitus **B** ♂, wings **C** ♂, metasomal sterna V–VIII, ventral view **D** geographic distribution.

*Sphex fumipennis* is distinguished from almost all other Australian *Sphex* in having largely dark wings, a black metasoma and silvery-white pubescence on the propodeal enclosure. There is only one other species with similar features, *Sphex rugifer*, and while most females of this species have a red metasoma, the males as well as some females have a black metasoma like *Sphex fumipennis*. A reliable characteristic of *Sphex fumipennis* is that the hindwing becomes almost completely hyaline towards the apical margin (Fig. 16B), while that of *Sphex rugifer* is, at least in the distal half, uniformly dark (Fig. 24A–C). Males of *Sphex fumipennis* also have a conspicuous fringe of silvery setae near the apex of metasomal sternum VIII (Fig. 16C), whereas males of *Sphex rugifer* lack this pubescence (Fig. 24D).

##### Description.

Body black, legs black or maroon. Wing membrane dark, with violet lustre near base, apical margin of forewing membrane and distal part beyond cellular area of hindwing membrane hyaline. Wing veins dark brown to black. Appressed pubescence on clypeus and frons silvery, erect setae on clypeus and frons black. Pubescence on top of collar silvery, long lateral setae black. Pubescence on scutum silvery, interspersed with few black setae near anterior margin. Silvery pubescence on scutum denser laterally. Propodeal enclosure sparsely covered with silvery-white pubescence, leaving sculpture completely visible. Posterior end of propodeum with black setae.

*Female*: 22.2–28.4 mm. Forebasitarsal rake with eight long spines. Free clypeal margin slightly scoop-shaped, with two distinct lobes medially and small bulge slightly above them; distance between lobes less than 1/8 length of flagellomere II. Clypeus except for bulge entirely pubescent. Distance between hind-ocelli nearly equal to their shortest distance to compound eyes. Scutellum flat, without impressions. Length of petiole almost equal to flagellomere II. Tomentum sparse on metasomal tergum I, absent on tergum II.

*Male*: Body length 15.2–21.2 mm. Free clypeal margin truncate, concave towards center. Medioventral part of clypeus glabrous. Distance between hind-ocelli 1.25× their shortest distance to compound eyes. Scutellum convex, with (sometimes shallow) medial impression. Length of petiole 1.25× length of flagellomere II. Tomentum moderately dense on metasomal terga I and II. Metasomal terga V and VI with a few black bristles. Metasomal sterna II–VI with few appressed silvery setae, sterna VII with several erect black setae, sternum VIII with a dense fringe of silvery setae in center. Metasomal sternum VIII entire, raised from side to center, its margin straight.

#### Sphex gilberti

Taxon classificationAnimaliaHymenopteraSphecidae

R. Turner, 1908

Sphex gilberti R. Turner, 1908: 468, ♀. Holotype or syntypes: ♀, Australia: Queensland: Mackay (BMNH). Presumed holotype examined.

##### Material examined.

*Holotype* (presumed). ♀, **AUSTRALIA:** **QLD:** Mackay, Feb 1892 (BMNH).

##### Other material.

**AUSTRALIA:** **NSW:** Lansdowne, 1♀, 06.02.1981, G. & T. Williams (AMS); **QLD:** Capricorn Group, NW Islet, 2♀, Dec 25, A. Musgrave (AMS), 1♀, Nov-Dec 25, A. Musgrave (AMS); Clump Point, 1♀, 06.03.1964, I. F. B. Common & M. S. Upton (ANIC); Montville, 1♀, L. Smith (ANIC).

The collecting localities are shown in Fig. 24E.

##### Diagnosis.

This species (of which only the female is known) is characterized by a black metasoma with a dark blue lustre, extensively yellow wings, and dark pubescence on scutum and propodeal enclosure. *Sphex resplendens*, which is otherwise similar, has uniformly dark wings. The pubescence on the propodeal enclosure of *Sphex modestus* is at least partially silvery or yellowish, and its hindwing membrane is missing the yellow tinge. *Sphex modestus* is also distinguished by the presence of tubercles on its metanotum, even though sometimes they are only faintly recognizable, while in *Sphex gilberti* the metanotum is plain.

##### Description.

*Female*: Body length 22.4–26.8 mm. Body black, legs brown. Wing membrane yellow, with slightly fuscous band at apex. Wing veins orange to light brown. Forebasitarsal rake with eigtht long spines. Free clypeal margin medially with two lobes which are separated only by a small notch. Appressed pubescence on clypeus and frons silvery, erect setae black. Clypeus with conspicuous indentation medioventrally and vertical glabrous stripe dorsoventrally. Distance between hind-ocelli approximately 0.6× their shortest distances to compound eyes. Pubescence on collar silvery. Scutum with sparse, erect, black setae and denser, silvery-white setae laterally. Scutellum slightly convex, without impressions. Propodeal enclosure with sparse, erect black pubescence, sculpture completely visible. Length of petiole almost equal to flagellomere II. Tomentum moderately dense on metasomal tergum I, sparse on tergum II.

*Male*: Unknown.

**Figure 17.**
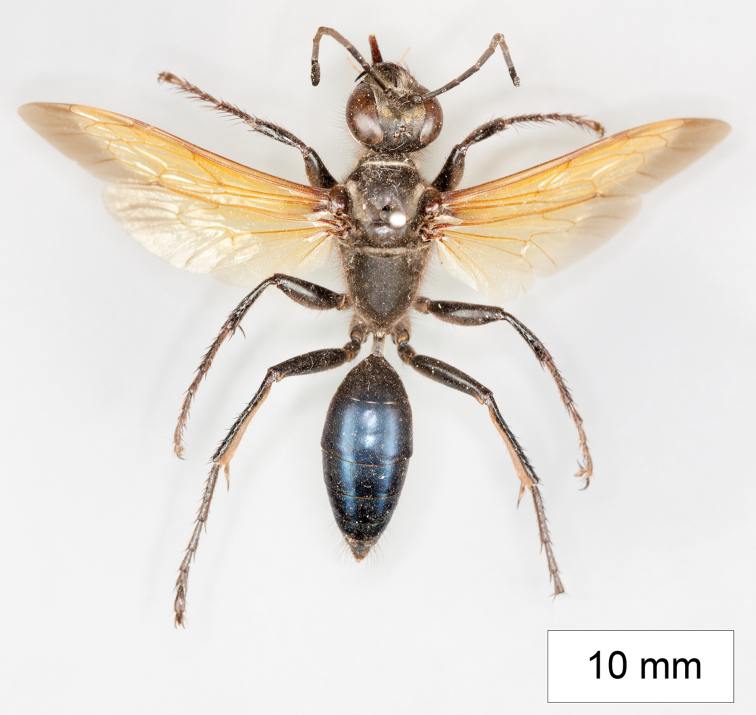
Habitus of *Sphex gilberti*, ♀.

#### Sphex gracilis sp. n.

Taxon classificationAnimaliaHymenopteraSphecidae

http://zoobank.org/90735F36-DD7A-434E-8AEE-B36314076386

##### Material examined.

*Holotype*. ♂, **AUSTRALIA:** **NSW:** 35 km N of Menindee, 26.11.1988, N. W. Rodd (AMS). *Paratypes*. **AUSTRALIA:** **NSW:** Broken Hill, 1♂, 15.02.1942, C. E. Chadwick (AMS); 40 km W of Cobar, 3♂, 12.11.1985, N. W. Rodd (AMS); 30 km N of Euston, 1♀, 28.11.1988, N. W. Rodd (AMS); Fowlers Gap, 114 km SW of Broken Hill, Barrier Range, 2♂, 21.12.1988, G. J. & R. L. Langston (CAS); Gilgandra, 4♀, 1♂, Nov-Dec 1986, G. A. Holloway (AMS); 40 km E of Gol Gol, 1♂, 27.11.1992, N. W. Rodd (AMS); Lightning Ridge, 1♂, 14.10.1989, I. D. Buddle (AMS); 20 km N of Menindee, 1♀, 10.11.1985, N. W. Rodd (AMS), 35 km N of Menindee, 2♂, 26.11.1988, N. W. Rodd (AMS); Pooncarie, 1♂, 27.11.1991, N. W. Rodd (AMS), 3♀, 28.11.1992, N. W. Rodd (AMS); 112 km N of Wentworth, 1♂, 28.11.1991, N. W. Rodd (AMS); 15 km E of Hillston, 1♀, 29.11.1988, N. W. Rodd (AMS); **SA:** Calperum Station 16 km N Renmark, 34°02.9'S, 140°42.2'E, 1♀, 03.12.2010, V. Ahrens & W. J. Pulawski (CAS), 1♂, 04.12.2010, V. Ahrens & W. J. Pulawski (CAS); 17 km E of Renmark, 1♀, 23.11.1977, D. K. McAlpine & M. A. Schneider (AMS); 3 km N Renmark, 34°09.5'S, 140°44.2'E, 1♂, 02.12.2010, V. Ahrens & W. J. Pulawski (CAS).

##### Diagnosis.

**Figure 18.**
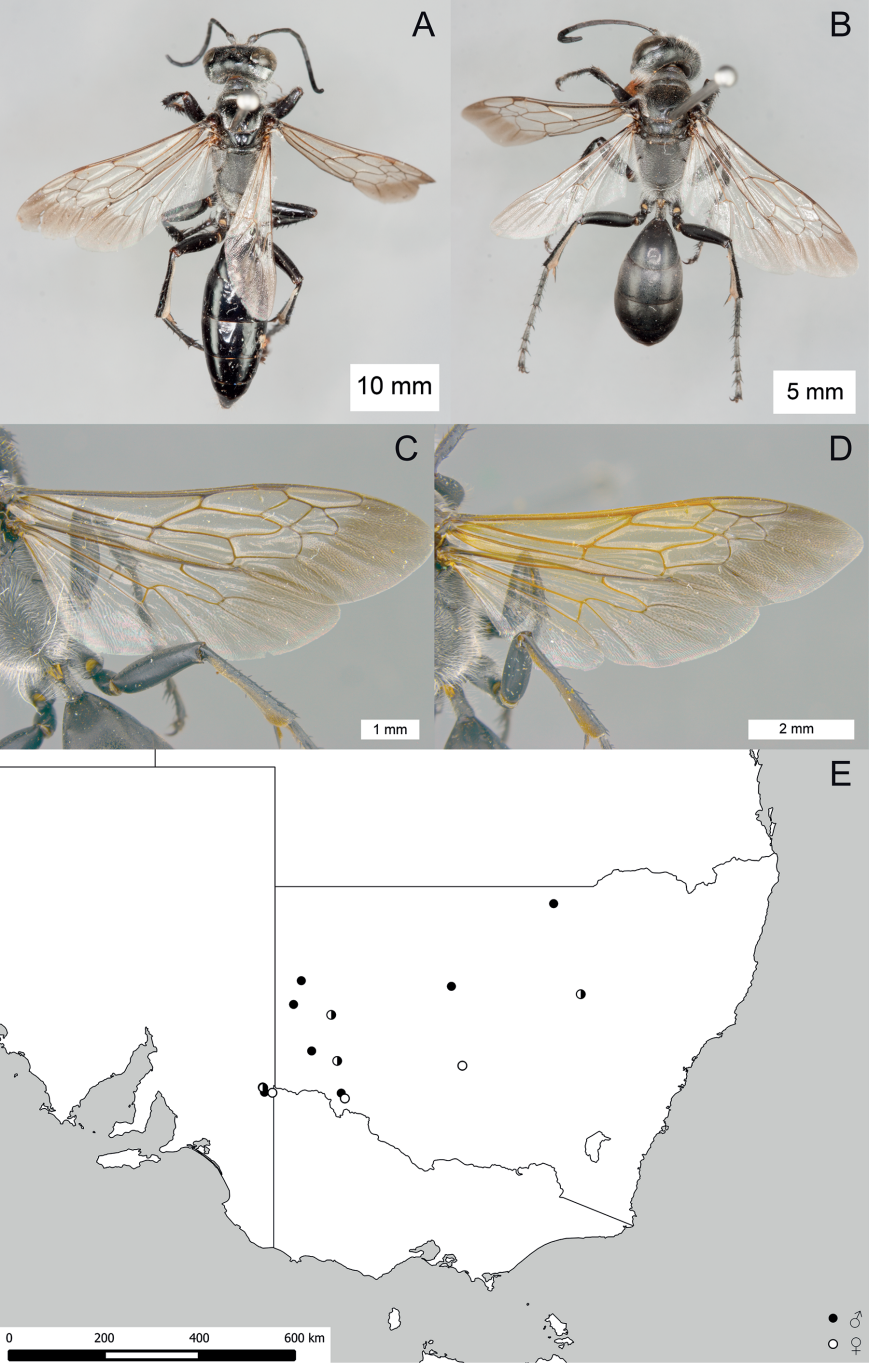
*Sphex gracilis*. **A** ♀, habitus **B** ♂, habitus **C** ♂ with brown wing veins and without yellow tinge on wings **D** ♂ with orange wing veins and a yellow tinge on wings **E** collecting localities, the combined symbol indicates that males and females were found in the same locality.

This species differs from other Australian *Sphex* of the *Sphex resplendens* group in having a combination of the following features: erect setae on clypeus uniformly silvery-white, metasoma black, and clypeal surface without wrinkles. Furthermore, the metasoma of female *Sphex gracilis* is considerably slenderer than that of other examined species (Fig. 18A).

##### Description.

Body black. Wing membrane hyaline, with fuscous band at apex. Wing veins orange to light brown. Appressed pubescence and erect setae on clypeus and frons silvery. Pubescence on mesosoma silvery-white. Scutellum with shallow medial impression. Pubescence on propodeal enclosure short and sparse, leaving sculpture easily visible.

*Female*: Body length 19.9–25.9 mm. Mandible basally and ventral part of clypeus reddish, distal tarsomeres sometimes inconspicuously orange. Wing veins darker near apex. Forebasitarsal rake with 5–8 spines which are markedly short and stout. Free clypeal margin faintly convex medially. Clypeus mostly glabrous, only laterally with pubescence. Distance between hind-ocelli equal to their shortest distance to compound eyes. Pubescence on scutum denser laterally and posteriorly. Scutellum flat. Length of petiole nearly equal to flagellomere II. Tomentum very sparse on metasomal tergum I, absent on tergum II.

*Male*: Body length 18.5–20.6 mm. Forewing membrane sometimes with yellow tinge, hindwing membrane entirely hyaline. Wing veins light brown, sometimes orange, often darker near apex, sometimes uniformly colored. Free clypeal margin simple, concave towards center. Clypeus with narrow medial glabrous stripe, ventral part also glabrous. Distance between hind-ocelli 0.9× their shortest distance to compound eyes. Pubescence on scutum denser laterally. Scutellum convex. Length of petiole 1.4× length of flagellomere II. Tomentum dense on metasomal tergum I, moderately dense on tergum II. Metasomal tergum V and VI with few bristles. Metasomal sterna II–VI mostly glabrous, apical margin of metasomal sternum VII and VIII covered with silvery pubescence. Metasomal sternum VIII entire, its lateral margin inconspicuously concave.

##### Discussion.

At first, the males and females of this species look quite different. While the habitus of the male matches that of most examined species of *Sphex*, the female’s metasoma is much slenderer and seems longer than that of other species. Nonetheless, they most likely are the same species, as indicated by the following features.

One of the most important characters that connect both sexes is the uniformly silvery pubescence on the clypeus. In at least six of the ten other Australian species in the *Sphex resplendens* group, the erect setae on the clypeus are black. Of the remaining four species, two are colored bright orange. One of the other two has extremely unusual structures on the clypeal surface. Concerning the last remaining species other than *Sphex gracilis*, male and female individuals were found that have both silvery and golden pubescence on the clypeus as well as on the propodeum, a unique trait among the studied material.

Moreover, this is also the only species in the *Sphex resplendens* group where some males and females (see below for explanation) have the wing membrane entirely hyaline (excluding the apical margin), even at the base, and without any yellow. Both also have a very similar sculpture on the propodeal enclosure, and they also share the same length, density and orientation of its pubescence. Finally, the collecting localities show that both sexes occur in close proximity (Fig. 18E).

There seems to be some variability among individuals, but they are nonetheless presumed to be of a single species. In both sexes, there are specimens that come from the same collecting series and are distinctively different in their wing coloration. One group possesses brown wing veins and wings that have no hint of yellow (Fig. 18C), the other group has orange wing veins and (in males) a yellow tinge in the basal half of the forewing membrane (Fig. 18D).

##### Etymology.

*Gracilis* is a Latin adjective meaning “slender”. It refers to the habitus of the female metasoma.

#### Sphex imporcatus sp. n.

Taxon classificationAnimaliaHymenopteraSphecidae

http://zoobank.org/E8309EF2-A030-4D2F-905D-28C66DB3B34A

##### Material examined.

*Holotype*. ♀, **AUSTRALIA:** **SA:** [no specific locality], 1937, M. F. L. (BMNH). *Paratypes*. **AUSTRALIA:** **NSW:** Fowlers Gap, 2♀, Dec 1981, M. L. Bason (AMS).

The collecting localities are shown in Fig. 23B.

##### Diagnosis.

*Sphex imporcatus* (of which only the female is known) is unique among Australian *Sphex* in having a remarkably modified clypeus (Fig. 4E, 19B). Dorsomedially on the clypeus, there is a deep groove of which the lower rim is encompassed by 5–8 markedly arcuate, broad wrinkles that almost reach the free clypeal margin. Furthermore, *Sphex imporcatus* possesses several distinct transverse ridges on the propodeum, a characteristic it only shares with *Sphex sericeus*, *Sphex darwiniensis* and *Sphex rugifer*. *Sphex sericeus*, as a member of the *Sphex argentatus* group, is set apart by having a pair of distinct tubercles on the metanotum and a partially ferruginous metasoma, while the metanotum of *Sphex imporcatus* lacks tubercles and its metasoma is black. The metasoma of *Sphex darwiniensis* is bright ferruginous, and *Sphex rugifer* can be distinguished by having fuscous wings, whereas *Sphex imporcatus* has hyaline wings with a slight yellow tinge.

##### Description.

*Female*: Body length 24.3–25.6 mm. Body black except for tarsomeres V, which are at least partly orange, and also base of mandible and ventral part of clypeus and the wrinkles on it, which range from orange to maroon. Forewing membrane with yellow tinge on basal half and a slightly fuscous band at apex, hindwing membrane slightly yellowish near base. Wing veins bright orange, almost yellow. Forebasitarsal rake with 6–7 spines which are markedly short and stout. Free clypeal margin inconspicuously notched medially. Clypeus dorsomedially with deep groove of which the lower end is encompassed by 5–8 markedly arcuate, broad wrinkles that almost reach the free clypeal margin. Appressed pubescence and erect setae on clypeus and frons silvery-white. Clypeus with medial glabrous stripe. Distance between hind-ocelli 0.9× their shortest distance to compound eyes. Pubescence on collar, scutum, metanotum and propodeal enclosure silvery-white, scutum glabrous except laterally and posteriorly. Scutellum flat, without impressions. Propodeal enclosure with 6–9 discontinuous transverse ridges, pubescence not completely concealing sculpture. Length of petiole 1.15× length of flagellomere II. Tomentum very sparse, on metasomal tergum I only present near center, absent on tergum II.

*Male*: Unknown.

**Figure 19.**
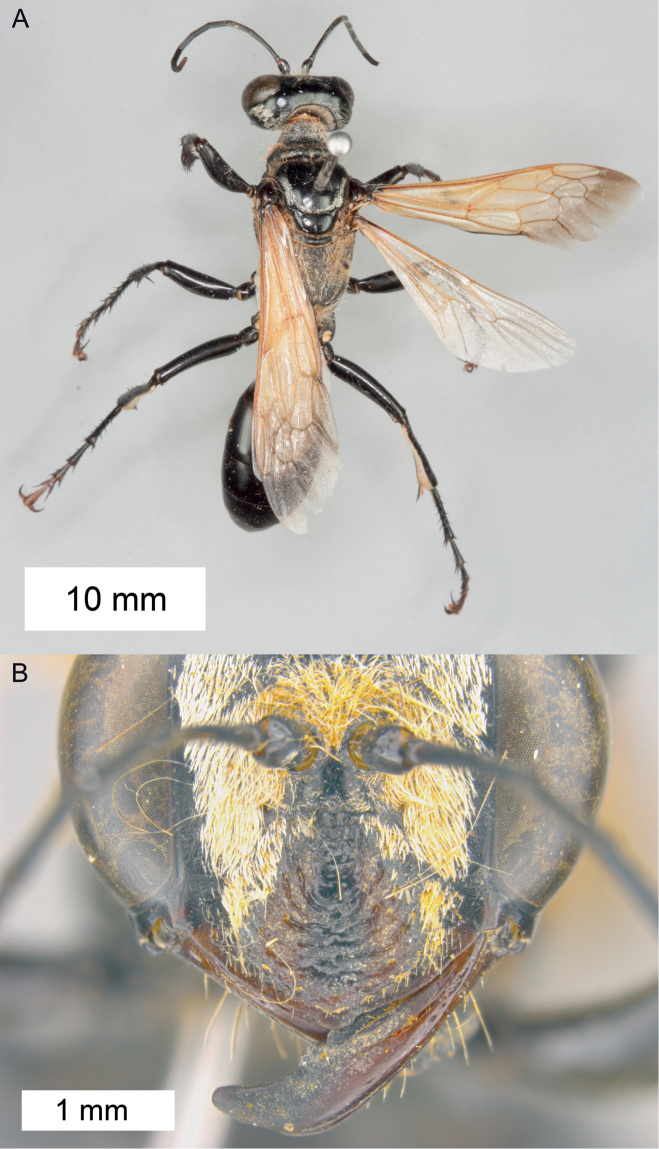
*Sphex imporcatus*, ♀. **A** habitus **B** frontal view of clypeus.

##### Discussion.

All three available specimens were heavily deteriorated, seemingly by abrasion. It is unknown whether the lesser length of the spines in the tarsal rake is also a result of abrasion, or whether it is characteristic of the species. Also, the original state of the pubescence on the clypeus and propodeum was probably very different from the current one.

##### Etymology.

*Imporcatus* comes from the Latin noun *porca* (ridge between two furrows) and means “put into furrows”, referring to the ridges on clypeus and propodeal enclosure of this species.

#### Sphex luctuosus

Taxon classificationAnimaliaHymenopteraSphecidae

F. Smith, 1856

Sphex luctuosus F. Smith, 1856: 250, ♀, ♂ (as *luctuosa*, incorrect original termination). Syntypes: Australia: Western Australia: Swan River (BMNH). One syntype examined.

##### Material examined.

Syntype. ♀, **AUSTRALIA:** **WA:** Swan River (BMNH).

##### Other material.

**[COUNTRY UNKNOWN]:** **[state unknown]:** [no specific locality], 1♂, 20.11.1927 (ANIC). **AUSTRALIA:** **[state unknown]:** [no specific locality], 4♀, 4♂ (BMNH); **NSW:** “National Park”, 1♀, 20.12.1914, C. Gibbons (AMS); Berowra, 1♂, 11.12.1923, Nicholson (AMS); 6 km NE of Bilpin, Blue Mountains, 1♂, 12.11.1980, N. W. Rodd (AMS); Heathcote, 1♀, 1♂, 03.01.1947 (AMS); Lorien Wildlife Refuge, 3 km N of Lansdowne, 1♀, 27.12.1991, G. Williams (AMS); Sydney, 3♀, 1♂, C. Gibbons (AMS); Woy Woy, 1♂, 08.03.1924, Nicholson (AMS); **QLD:** Brisbane, 1♀, 07.02.1923, A. N. Burns (ANIC); Caloundra, 1♂, 17.12.1955, J. Keir (ANIC); Cape York, 1♀, 1904, Elgner (ANIC); Halliday Bay, 50 km NE of Mackay, 1♀, 19.09.1983, N. W. Rodd (AMS); 40 km E of Miriam Vale, 1♂, 03.11.1984, N. W. Rodd (AMS); **VIC:** Rye, 1♀, 07.02.1954, A. N. Burns (ANIC); **WA:** Hamelin Telegraph Station, 26°23.9'S, 114°09.9'E, 1♀, 10.11.2008, V. Ahrens & W. J. Pulawski (CAS); 20 km WNW of Israelite Bay, 1♂, 29.12.1990, M. S. & B. J. Moulds (AMS); Lyndon Station, NW Basin, 1♀, 01.07.1950, G. Thomas (ANIC).

##### Diagnosis.

*Sphex luctuosus* differs from all other species in the *Sphex resplendens* group in having wings for the most part hyaline and lacking a yellow tinge, but darkened basally. *Sphex gracilis* also has hyaline wings, but not fuscous basally.

##### Description.

Body black. Appressed pubescence on clypeus and frons silvery, erect setae on clypeus and frons black. Wing membrane darkened basally, forewing with dark costal and marginal cell and a fuscous band at apex. Hindwing membrane hyaline except basally. Clypeus with small glabrous spot medioventrally. Pubescence on top of collar silvery, long lateral setae black. Pubescence on scutum denser laterally. Pubescence on propodeal enclosure silvery-white, thin and sparse enough to leave sculpture completely visible, interspersed with few black setae near posterior end.

*Female*: Body length 24.6–32.4 mm. Wing veins brown to light brown. Forebasitarsal rake with 9–11 long spines. Free clypeal margin with two distinct lobes medially, bulging slightly above; distance between lobes less than 1/8 length of flagellomere II. Distance between hind-ocelli approximately 0.8× their shortest distance to compound eyes. Anterior half of scutum covered with silvery pubescence. Scutellum flat, without impressions. Length of petiole almost equal to flagellomere II. Tomentum sparse on metasomal tergum I, absent on tergum II.

*Male*: Body length 23.2–26.8 mm. Wing veins dark brown. Free clypeal margin truncate, slightly concave medially. Distance between hind-ocelli 1.25× their shortest distance to compound eyes. Anterior half of scutum covered with black pubescence. Scutellum convex, with shallow medial impression. Length of petiole 1.4× length of flagellomere II. Tomentum moderately dense on metasomal terga I and II. Metasomal terga V and VI with few black bristles. Metasomal sterna II–VI largely glabrous, sternum VII covered with erect black setae, sternum VIII with dense fringe of silvery setae medially. Metasomal sternum VIII entire, its lateral margin straight.

**Figure 20.**
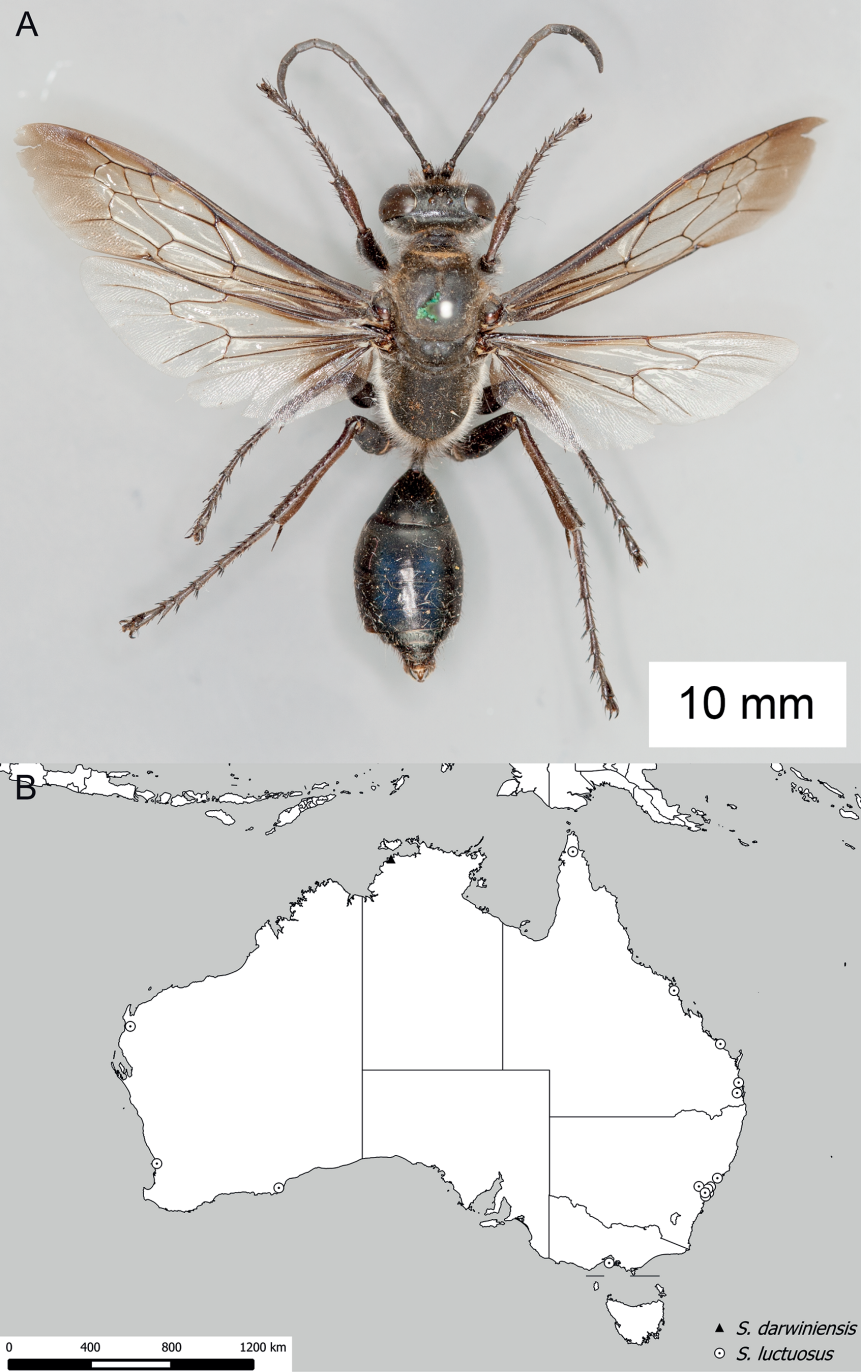
*Sphex luctuosus*. **A** ♂, habitus **B** collecting localities, those of *Sphex darwiniensis* are also shown.

#### Sphex mimulus

Taxon classificationAnimaliaHymenopteraSphecidae

R. Turner, 1910

Sphex mimulus R. Turner, 1910: 419, ♀. Holotype or syntypes: ♀, Australia: Queensland: Cairns (BMNH). Not examined.

##### Material examined.

**AUSTRALIA:** **QLD:** Cape York, 1♀, 1♂, 1904, Elgner (ANIC), 2♀, 1908, Elgner (ANIC); Claudie River, 4 miles W of Mount Lamond, 1♀, 31.12.1971 (AMS); Iron Range, Cape York Peninsula, 1♀, 01.-09.06.1971, S. R. Monteith (ANIC), 3♀, 26.-31.05.1971, S. R. Monteith (ANIC); Mid Claudie River, Iron Range, 1♀, 26.08.1974, G. Daniels (AMS), 1♀, 16.09.1974, G. Daniels (AMS); Mount Lamond, Iron Range, 1♀, 18.09.1974, G. Daniels (AMS); 1 mile NE of Mount Lamond, 1♂, 21.12.1971, D. K. McAlpine, G. A. Holloway, D. P. Sands (AMS); Mungumby Lodge near Helenvale, 1♀, 09.06.1991, N. W. Rodd (AMS).

The collecting localities are shown in Fig. 24E.

##### Diagnosis.

Females of *Sphex mimulus* are unique among the Australian members of the *Sphex resplendens* group in having black legs and the first three segments of the metasoma while the apical three segments are bright ferruginous. *Sphex decoratus* and some females of *Sphex rugifer* vary among similar colors, but as a member of the *Sphex argentatus* group, the former has a pair of distinct tubercles on the metanotum, whereas *Sphex mimulus*, as a member of the *Sphex resplendens* group, lacks the metanotal tubercles. *Sphex rugifer* lacks the yellow tinge in the cellular wing area that is present in *Sphex mimulus*. Until now, the male of *Sphex mimulus* is the only one in the *Sphex resplendens* group with a partially orange or yellowish metasoma.

##### Description.

Base of wing membrane darkened, apex slightly fuscous, cellular wing area with pale yellowish tinge. Clypeus with small indentation medioventrally. Appressed pubescence on clypeus and frons silvery, erect setae on clypeus and frons black. Pubescence on mesosoma silvery, on scutum longer and denser laterally. Pubescence on propodeal enclosure sparse and thin, leaving sculpture completely visible. Tomentum on metasomal tergum I moderately dense, on tergum II sparse.

*Female*: Body length 26.4–32.8 mm. Body black, but apical three metasomal terga and apical 3–4 metasomal sterna orange. Wing veins light to dark brown. Forebasitarsal rake with nine spines. Free clypeal margin with two lobes medially, bulging slightly above; distance between lobes less than 1/8 length of flagellomere II. Clypeus with medial, narrow, more or less glabrous stripe. Distance between hind-ocelli slightly greater than their shortest distance to compound eyes. Scutellum completely flat, without impressions. Petiole slightly shorter than flagellomere II.

*Male*: Body length 19.6–25.4 mm. Body black, but apical three metasomal terga pale yellowish-orange. Wing veins dark brown. Free clypeal margin truncate. Clypeus medially with mostly glabrous stripe. Distance between hind-ocelli 0.75× their shortest distance to compound eyes. Scutum with faint longitudinal posteromedian impression. Scutellum convex, with medial impression. Length of petiole 1.3× length of flagellomere II. Metasomal terga V and VI with few silvery bristles. Metasomal sterna II and III mostly glabrous, IV–VII with increasingly dense (but still rather sparse) fringes of brown setae laterally, sternum VIII with dense, silvery fringe near apical margin. Metasomal sternum VIII relatively long and tongue-shaped, entire, its lateral margin straight.

**Figure 21.**
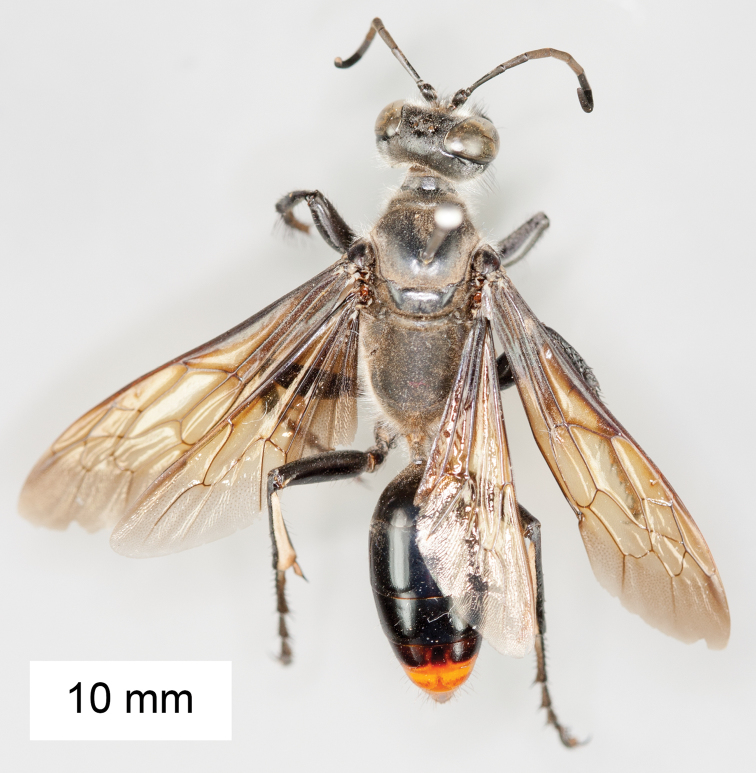
Habitus of *Sphex mimulus*, ♀.

##### Notes on type material.

The type of *Sphex mimulus* was not examined, because the characters in the original description (metasoma black anteriorly, apical metasomal segments red) are sufficient to unambiguously identifiy this species.

#### Sphex resplendens

Taxon classificationAnimaliaHymenopteraSphecidae

Kohl, 1885

Sphex nitidiventris F. Smith, 1859: 158, ♀, junior primary homonym of *Smith nitidiventris* Spinola, 1853. Holotype or syntypes: ♀, Indonesia: Maluku: Aru Island (OXUM). Synonymized with *Sphex gratiosissimus* by Turner (1910: 346), and with *Sphex refulgens* by Turner (1919: 238). Not examined.
Sphex resplendens Kohl, 1885: 200. Substitute name for *Sphex nitidiventris* F. Smith.

##### Material examined.

**[COUNTRY UNKNOWN]:** **[state unknown]:** [no specific locality], 1♀, 09.01.1952 (AMS), 2♂ (ANIC). **AUSTRALIA:** **NSW:** 50 km NW of Taree, Doyles River, 31°31'S, 152°14'E, 1♀, 10.03.2010, D. Bray (AMS), Rosebank, 1♀, 10.11.1990, N. W. Rodd (AMS); **QLD:** “North Queensland”, 1♀ (BMNH), [no specific locality], 1♂ (ZMB); Bulburin State Forest via Many Peaks, 1♂, 02.-05.04.1972, S. R. Monteith (ANIC); Capricorn Group, NW Islet, 4♂, Dec 1925, A. Musgrave (AMS); Cooktown, 1♂, 18.07.1982, N. W. Rodd (AMS); Mackay, 1♀, 1947, A. Marriage (AMS), 4♂ (BMNH); Meringa, 1♂, 14.02.1926, A. N. Burns (ANIC); Mid Claudie River, Iron Range, 1♀, 25.09.1974, G. Daniels (AMS); Rockhampton, 1♀ (ZMB); **TAS:** Key Island, 1♀, 1♂, Rolle (ZMB). **PAPUA NEW GUINEA:** **[province unknown]:** [no specific locality], 1♀, Jan-Feb 1910, Neuhauss (ZMB), 1♀, Bürgers (ZMB).

The collecting localities are shown in Fig. 23B.

##### Diagnosis.

*Sphex resplendens* differs from all other examined species in having completely fuscous both fore- and hindwings, a black metasoma that often has a metallic blue lustre, and dark setae on the propodeal enclosure. *Sphex gilberti* is similar, but has bright yellowish wings.

##### Description.

Body black, legs dark maroon. Metasoma often with blue lustre. Wing membrane uniformly dark, with violet lustre. Wing veins black. Erect setae on clypeus black. Pubescence on collar silvery, with erect black setae. Scutum with silvery pubescence that is denser laterally, anteriorly interspersed with black setae. Propodeal enclosure covered with erect black setae, not concealing sculpture. Length of petiole nearly equal to flagellomere II.

*Female*: Body length 22.1–26.9 mm. Forebasitarsal rake with eight long spines. Free clypeal margin with two incosnpicuous lobes medially, bulging above; distance between lobes less than 1/8 length of flagellomere II. Appressed pubescence on clypeus and frons sparse, thin and silvery. Clypeus medially with large glabrous area. Distance between hind-ocelli 0.9× their shortest distance to compound eyes. Scutellum and metanotum with sparse, erect black setae. Tomentum sparse on metasomal tergum I and II.

*Male*: Body length 20.5–25.4 mm. Free clypeal margin truncate, concave towards center. Appressed pubescence on clypeus and frons dense and golden. Clypeus with medial glabrous stripe, most of ventral clypeal area glabrous. Distance between hind-ocelli 0.8× their shortest distance to compound eyes. Scutellum and metanotum with sparse, erect silvery setae, laterally with erect black setae. Tomentum moderately dense on metasomal tergum I and II. Metasomal terga V and VI with few black bristles. Metasomal sterna II and III mostly glabrous, sterna IV–VII with brassy pubescence and long black setae at apical margin, densest on sternum VI. Metasomal sternum VIII entire, with few short silvery setae posteriorly, its lateral margin slightly concave there.

**Figure 22.**
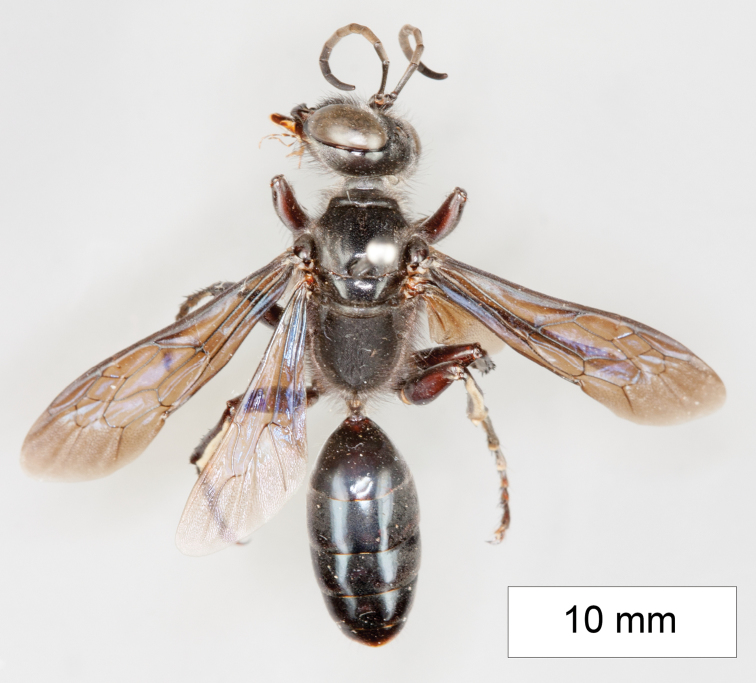
Habitus of *Sphex resplendens*, ♀.

##### Notes on type material.

The types of *Sphex resplendens* and its synonyms were not examined because the character combination in the original description (black pubescence on propodeum, fuscous wings) is sufficient to unambiguously identifiy this species.

#### Sphex rhodosoma

Taxon classificationAnimaliaHymenopteraSphecidae

(R. Turner, 1915)

Chlorion rhodosoma R. Turner, 1915: 65, ♀. Syntypes: ♀, Australia: Western Australia: Cue and Cunderdin (BMNH). Syntype examined. – As *Sphex rhodosoma*: Bohart and Menke (1976: 116) (new combination, in checklist of world Sphecidae).

##### Material examined.

Syntype. ♀, **AUSTRALIA:** **WA:** Cue, H. W. Brown (BMNH).

##### Diagnosis.

*Sphex rhodosoma* (of which only the female is known) is unique among the Australian *Sphex* in combining the features of the *Sphex resplendens* group with a mostly orange body including orange legs and a petiole which is slightly longer than flagellomere II. A partially orange mesosoma is also found in *Sphex sericeus*. In that species, however, the propodeal enclosure is ridged and the metanotum has a pair of distinct tubercles, while *Sphex rhodosoma* lacks both these features.

##### Description.

*Female*: Body length 20.2 mm. Body orange, but the following are black: apical mandibular tooth, frons, vertex, gena, flagellomere II above and flagellomeres III–X entirely, parts of metasomal terga II, III and IV, parts of metasomal sternum II. Wing membrane yellow, with slightly fuscous band near apex. Wing veins brown, orange near base. Forebasitarsal rake with six long spines. Free clypeal margin with two lobes medially, distance between them less than 1/8 length of flagellomere II. Appressed pubescence and erect setae on clypeus and frons silvery-white. Clypeus with medial glabrous stripe. Distance between hind-ocelli equal to their shortest distance to compound eyes. Pubescence on collar and scutum silvery-white, on the latter longer and denser laterally and posteriorly. Scutellum flat, shiny, without impressions. Propodeal enclosure with short, appressed silvery-white pubescence, sculpture almost completely visible. Length of petiole approximately 1.1× length of flagellomere II. Tomentum on metasomal sternum I moderately dense, on sternum II sparse.

*Male*: Unknown.

**Figure 23.**
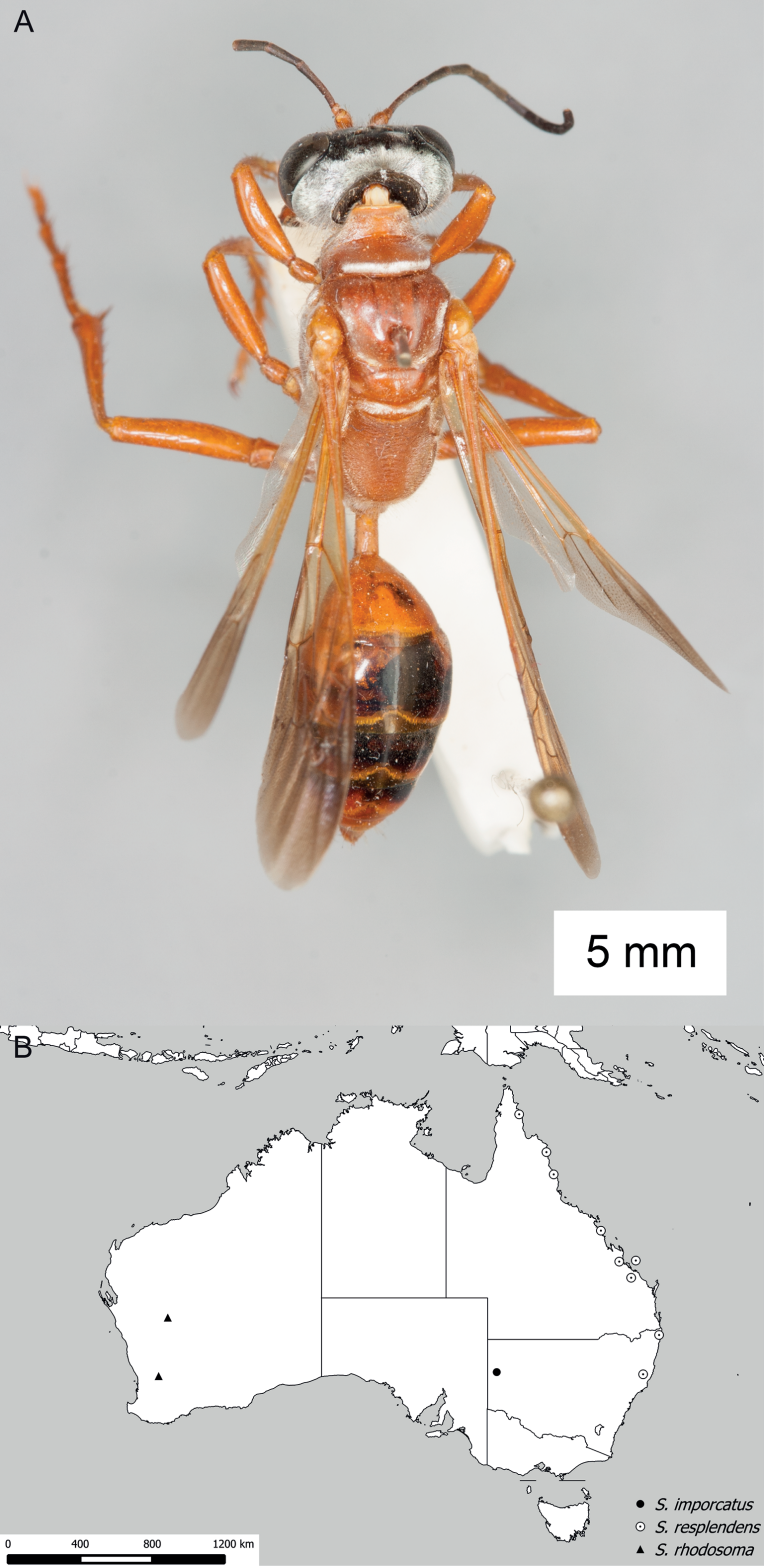
*Sphex rhodosoma*. **A** ♀, habitus. **B** collecting localities, those of *Sphex imporcatus* and *Sphex resplendens* are also shown.

#### Sphex rugifer

Taxon classificationAnimaliaHymenopteraSphecidae

Kohl, 1890

Sphex rugifer Kohl, 1890: 393, ♀. Syntypes: Australia: no specific locality (ZMB) and Western Australia: Swan River (NHMW). One syntype examined.

##### Material examined.

Syntype: ♀, **AUSTRALIA:** **[state unknown]:** [no specific locality], Preiss (ZMB).

##### Other material.

**AUSTRALIA:** **WA:** Applecross, 1♂, 28.01.1940, K. R. Norris (ANIC); Bunbury, 1♀, 2♂, 01.01.1961, A. Snell (AMS); Eradu, 1♀, J. Clark (ANIC); Fremantle City, 1♀, 28.01.1935, K. R. Norris (ANIC); 1♂, 09.02.1935, K. R. Norris (ANIC), 2♂, 09.02.1936, K. R. Norris (ANIC); Geraldton, 1♂, 1914 (ANIC), 1♀, 09.02.1931, L. F. Graham (ANIC); Perth, 1♀, 09.04.1914, J. Clark (ANIC); Shire of Waroona, Yalgorup National Park, 32°52'51"S, 115°40'35"E, 1♀, 4♂, 25.01.2010, L. Breitkreuz (ZMB); Shire of Waroona, Yalgorup National Park, 32°52'49"S, 115°40'57"E, 1♀, 27.01.2010, L. Breitkreuz (ZMB); Shire of Waroona, Yalgorup National Park, 32.880879°S, 115.676464°E, 1♂, 27.01.2010, S. Krause (ZMB); Shire of Waroona, Yalgorup National Park, 32°52'49"S, 115°40'57"E, 2♂, 27.01.2010, L. Breitkreuz (ZMB); Shire of Waroona, Yalgorup National Park, 32.880879°S, 115.676464°E, 1♂, 27.01.2010, S. Krause (ZMB); Shire of Waroona, Yalgorup National Park, 32°52'49"S, 115°40'57"E, 5♂, 27.01.2010, L. Breitkreuz (ZMB); Shire of Waroona, Yalgorup National Park, 32.880879°S, 115.676464°E, 1♂, 27.01.2010, S. Krause (ZMB).

##### Diagnosis.

**Figure 24.**
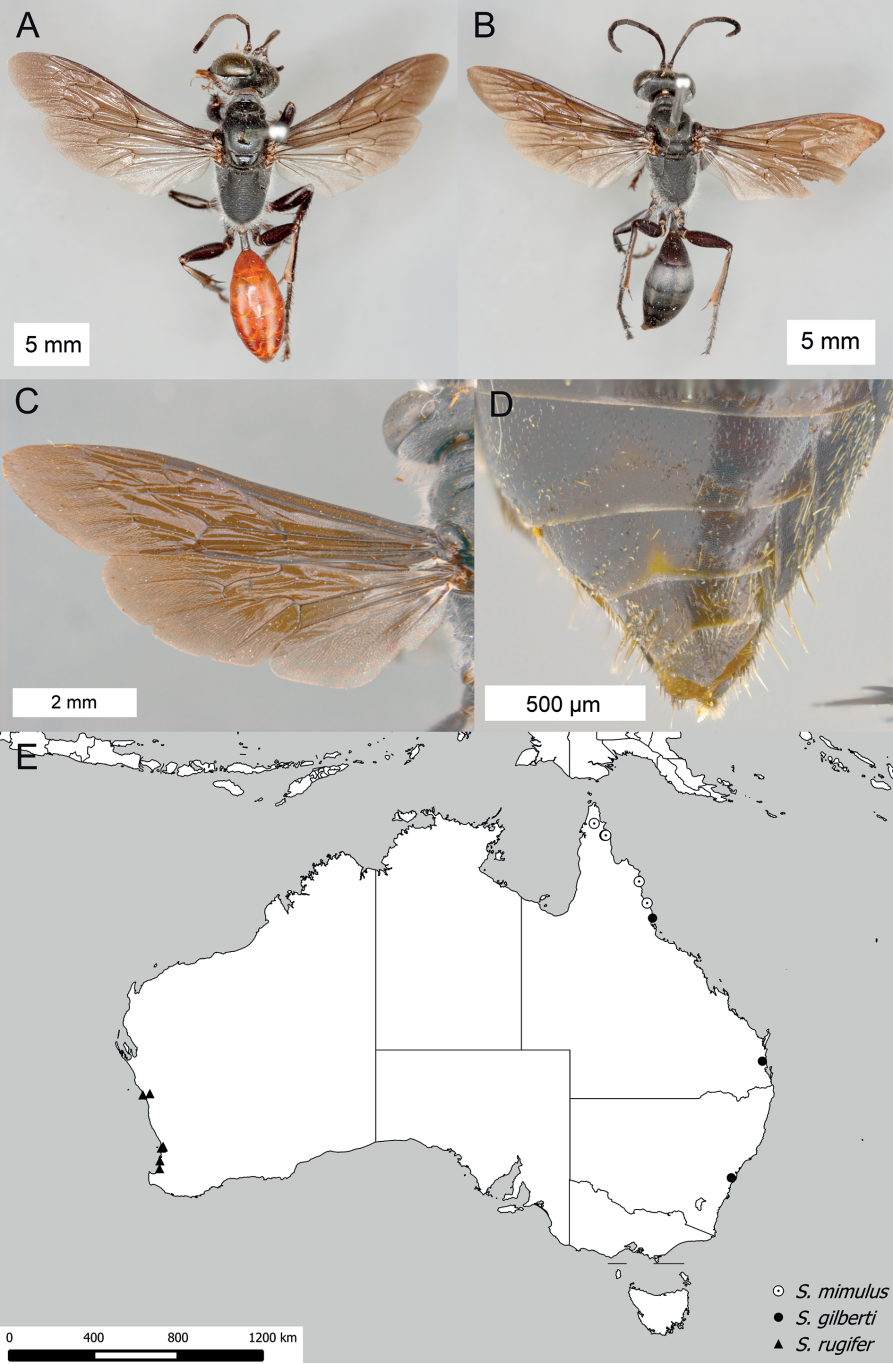
*Sphex rugifer*. **A** ♀, habitus **B** ♂, habitus **C** ♂, wings **D** ♂, ventral view of metasomal sterna V–VIII **E** collecting localities; those of *Sphex mimulus* and *Sphex gilberti* are also shown.

*Sphex rugifer* differs from other Australian *Sphex* through a combination of the following characters: uniformly darkened forewing membrane without yellow tinge, hindwing membrane fuscous beyond the cellular area (Fig. 24A–C), and silvery-white pubescence on the propodeal enclosure. Females can also be identified, in addition to the aforementioned features, in having a red metasoma, though the tone is often very dark. Both sexes of *Sphex resplendens* differ in having dark pubescence on the propodeum; and in *Sphex fumipennis*, the distal part of the hindwing membrane is hyaline (Fig. 16B). Males of *Sphex fumipennis* also have a conspicuous fringe of silvery setae near the apex of metasomal sternum VIII (Fig. 16C), whereas males of *Sphex rugifer* lack this pubescence (Fig. 24D).

##### Description.

Pro- and mesosoma black, but the following are light to dark brown: legs excluding coxae and basal half of claws excluding claw teeth. Basal half of mandible reddish. Forewing membrane uniformly fuscous. Hindwing membrane hyaline basally, remainder fuscous. Wing veins dark brown. Appressed pubescence on clypeus and frons silvery, erect setae on clypeus black, on frons silvery. Clypeus with narrow medial glabrous stripe. Pubescence on mesosoma silvery, on scutum denser laterally. Scutellum slightly convex, with shallow medial impression. Pubescence on propodeal enclosure sparse enough to leave sculpture clearly visible. Length of petiole 1.4× length of flagellomere II.

*Female*: Body length 18.0–20.6 mm. Metasoma ranges from bright red to black with few dark red blurs. Forebasitarsal rake with 9–10 long spines. Free clypeal margin slightly scoop-shaped, with inconspicuous notch medially. Distance between hind-ocelli equal to their shortest distance to compound eyes. Propodeal enclosure with approximately 20 fine transverse ridges. Tomentum moderately dense on metasomal terga I and II.

*Male*: Body length 14.5–19.0 mm. Metasoma black. Free clypeal margin truncate, slightly concave medially. Distance between hind-ocelli 1.1× their shortest distance to compound eyes. Propodeal enclosure with approximately 8–10 faint transverse ridges. Tomentum dense on metasomal terga I and II. Metasomal terga V and VI with few bristles. Metasomal sterna II–VI mostly glabrous, sternum VII with erect silvery setae laterally. Metasomal sternum VIII entire, with silvery pubescence, its lateral margin straight.

### Species group of *Sphex subtruncatus*

This group encompasses all species that cannot be assigned to the other two, and is only defined by the lack of specializations found in the other two groups (Hensen 1991). Members are characterized by claw teeth that are obliquely orientated to the claw margin and a mostly plain metanotum without tubercles (see diagnosis of *Sphex argentatus* group for details). With 18 Australian species, it is also the largest of the three groups. Nine species are new.

#### Sphex ahasverus

Taxon classificationAnimaliaHymenopteraSphecidae

Kohl, 1890

Sphex ahasverus Kohl, 1890: 397, ♀. Holotype or syntypes: Australia: southern Australia: no specific locality (NHMW). Presumed holotype examined.

##### Material examined.

*Holotype* (presumed). ♀, **AUSTRALIA:** **SA:** [no specific locality], 1804, Fichtel (NHMW).

##### Diagnosis.

This species (of which only the female is known) is unique among the members of the *Sphex subtruncatus* group in the distance between hind-ocelli being less than 60 % of their shortest distance to the compound eyes. Also, the combination of dense, erect, short black setae on the scutum and the dense golden pubescence on the propodeum makes it easy to identify.

##### Description.

*Female*: Body length 29.4 mm. Body black. Wing membrane with brown tinge, forewing also with slightly darker band at apex. Wing veins brown. Forebasitarsal rake with eight long spines; it is, however, likely that at least one additional spine was present, but has broken off. Free clypeal margin with two inconspicuous lobes medially, distance between them less than 1/8 length of flagellomere II. Appressed pubescence on clypeus and frons golden, long erect setae black. Clypeus with narrow medial glabrous stripe. Distance between hind-ocelli less than 0.6× their shortest distance to compound eyes. Pubescence on collar silvery. Scutum with short black pubescence which is denser laterally than medially. Scutellum convex, with shallow medial impression. Propodeum with dense, appressed pubescence and longer, more sparse, erect golden setae. Propodeal sculpture completely concealed by pubescence. Length of petiole 1.5× length of flagellomere II. Tomentum moderately dense on metasomal tergum I and II.

*Male*: Unknown.

**Figure 25.**
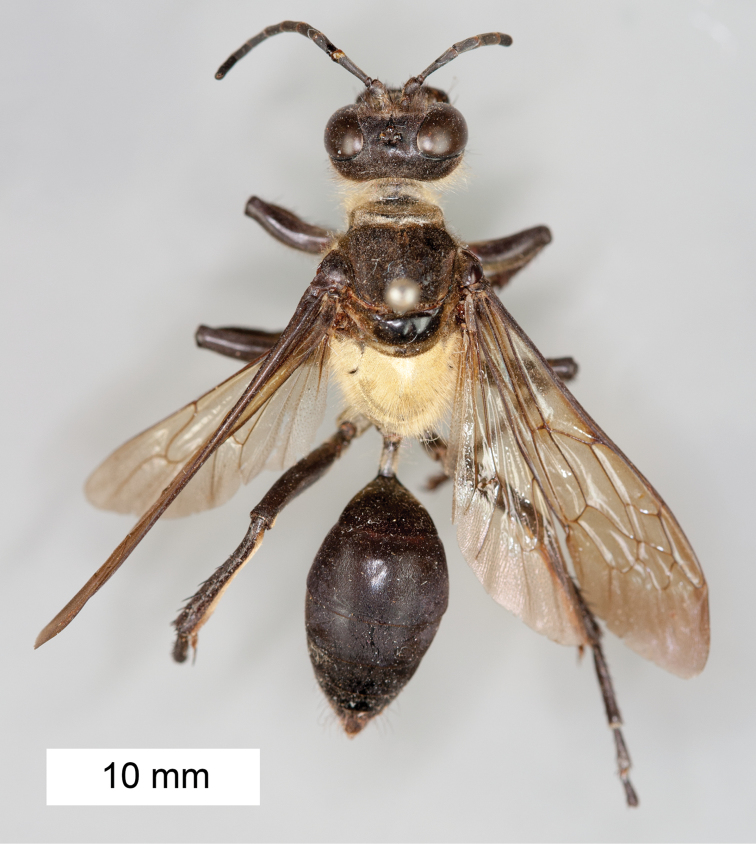
Habitus of *Sphex ahasverus*, ♀.

##### Geographic distribution.

*Sphex ahasverus* is known solely from the type locality, which is listed only as South Australia.

#### Sphex argentatissimus sp. n.

Taxon classificationAnimaliaHymenopteraSphecidae

http://zoobank.org/A8F23CE4-EC19-4246-9D30-041A554E7A79

##### Material examined.

*Holotype*. ♂, **AUSTRALIA:** **NT:** 27.5 km SE of Katherine, 14°34'0"S, 132°28'5"E, 08.04.2008, G. Williams & W. Pulawski (AMS). *Paratypes*. **AUSTRALIA:** **NT:** Buley Rockpools, Litchfield National Park, 2♀, 19.-22.04.2008, G. Williams & W. Pulawski (AMS); Litchfield National Park: Wangi Falls, 13°09.7'S, 130°40.9'E, 1♀, 22.04.2008, W. J. Pulawski & G. A. Williams (CAS); 29 km NW Mataranka, 14°45.5'S, 132°51.1'E, 2♂, 06.04.2008, W. J. Pulawski & G. A. Williams (CAS).

##### Diagnosis.

Females of *Sphex argentatissimus* are unique among the members of the *Sphex subtruncatus* group in combining the following characters: appressed pubescence on clypeus golden, wing membrane without yellow tinge, and pubescence on propodeal enclosure silvery-white. Males differ from those of the other species in possessing, additionally to the aforementioned features, a petiole that is considerably longer than flagellomere II and a conspicuous notch on the apical margin of metasomal sternum VIII. The former feature separates *Sphex argentatissimus* from *Sphex ermineus*, of which the petiole is shorter than flagellomere II, the latter one from *Sphex cognatus* and *Sphex formosellus*, in which the apical margin of metasomal sternum VIII is pointed.

##### Description.

Body black. Wing membrane hyaline, with fuscous band at apex. Wing veins dark brown to black. Appressed pubescence on clypeus and frons and erect setae on frons golden. Clypeus almost entirely covered with pubescence. Pubescence on collar and scutum brassy, except laterally and posteriorly on scutum, where it is denser and silvery-white. Scutellum convex. Propodeal enclosure covered with silvery-white pubescence, completely concealing sculpture.

*Female*: Body length 20.8–21.8 mm. Forebasitarsal rake with nine long spines. Free clypeal margin concave medially, with two lobes there, distance between which is less than 1/8 length of flagellomere II. Clypeus with erect golden setae. Distance between hind-ocelli 1.1× their shortest distance to compound eyes. Scutellum without impressions. Length of petiole 1.6× length of flagellomere II. Tomentum dense on metasomal tergum I and II.

*Male*: Body length 23.3–24.6 mm. Free clypeal margin truncate. Clypeus with few erect silvery-golden setae. Distance between hind-ocelli equal to their shortest distance to compound eyes. Posterior half of scutum with longitudinal median impression. Scutellum with medial impression near posterior margin. Length of petiole 1.4× length of flagellomere II. Tomentum moderately dense on metasomal tergum I, sparse on tergum II. Metasomal terga V and VI with few bristles. Metasomal sternum VII with small fringe of silvery setae posterolaterally, sterna VI–I each with decreasing amount of setae. Apical margin of metasomal sternum VIII conspicuously notched so that two small lobes are formed, its lateral margin slightly concave posteriorly.

**Figure 26.**
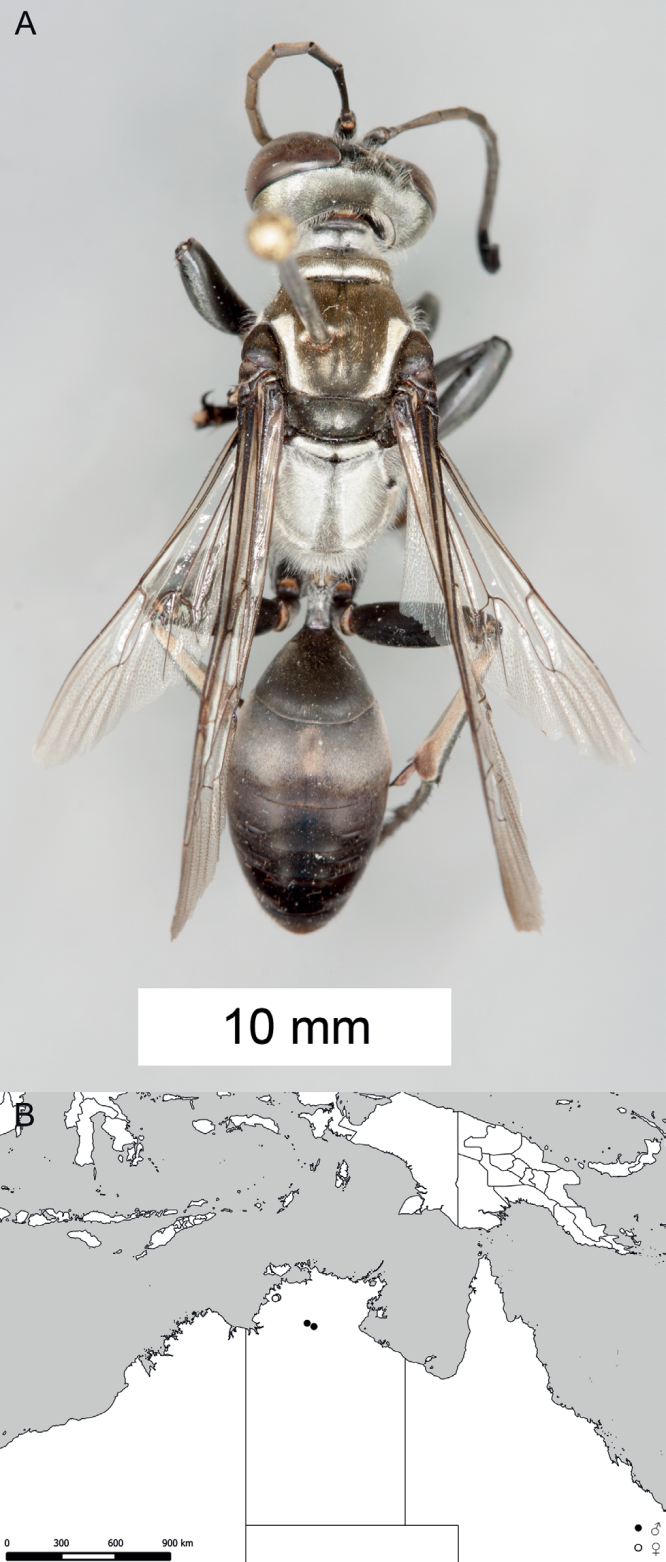
*Sphex argentatissimus*. **A** ♂, habitus **B** collecting localities.

##### Discussion.

The males of this species have very reliable and distinctive features in which they differ from the other Australian *Sphex*. These features, however, are male sexual characters (free clypeal margin and metasomal sternum VIII) and thus do not help in establishing the sex association. Still, several characters indicate that both males and females belong to a single species.

In the *Sphex subtruncatus* group, there are only two species whose clypeal and propodeal pubescence resembles that of *Sphex argentatissimus*. One of them, *Sphex pretiosus*, is easily identifiable in possessing both silvery-white and golden pubescence on the propodeum. The females of the other one, *Sphex cognatus*, have golden, silvery-golden or silvery pubescence on the propodeal enclosure and are indeed very similar to those of *Sphex argentatissimus*. However, of the many examined females of *Sphex cognatus*, none had propodeal pubescence with the conspicuous white tone of silver that is characteristic of the males and females of *Sphex argentatissimus*.

Wing coloration is a similar case. Females of *Sphex cognatus* usually have a partially yellow tinged wing membrane, but sometimes it is almost entirely hyaline. On the other hand, the wings of *Sphex argentatissimus* have no hint of yellow at all.

Fig. 26B shows that the males and females of this species occur at least in the same overall region.

##### Etymology.

*Argentatissimus* is the superlative of the Latin adjective *argentatus* (silvery) and refers to the fact that the silvery color of the propodeal pubescence is much more intense in this one than in the similar species.

#### Sphex basilicus

Taxon classificationAnimaliaHymenopteraSphecidae

(R. Turner, 1915)

Chlorion basilicus R. Turner, 1915a: 65, ♀. Holotype or syntypes: ♀, Australia: Queensland: probably Cape York Peninsula (BMNH). Not examined. – As *Sphex basilicus*: Bohart and Menke (1976: 116) (new combination, in checklist of world Sphecidae).

##### Material examined.

**AUSTRALIA:** **QLD:** Claudie River near Mount Lamond, 1♂, 02.06.1966, D. K. McAlpine (AMS); 1 mile NE of Mount Lamond, 1♀, 26.12.1971, D. K. McAlpine, G. A. Holloway, D. P. Sands (AMS); 4 miles W of Mount Lamond, 3♀, 2♂, 12.01.1972, D. K. McAlpine & G. A. Holloway (AMS), 1♂, 13.01.1972, D. K. McAlpine & G. A. Holloway (AMS).

The collecting localities are shown in Fig. 35B.

##### Diagnosis.

**Figure 27.**
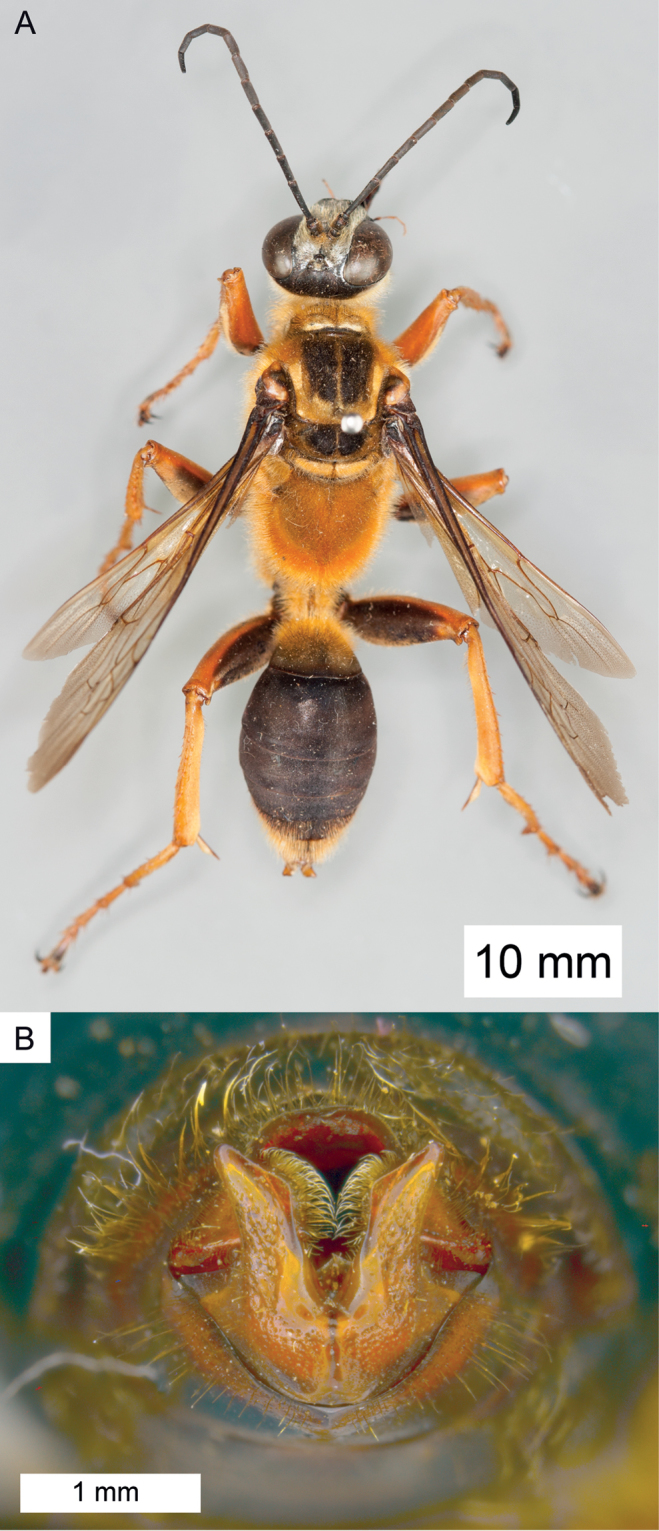
*Sphex basilicus*, ♂. **A** habitus **B** posterior view of metasomal sternum VIII.

*Sphex basilicus* differs from most other Australian *Sphex* by the color of its legs, which are orange from the distal half of the femur up to but excluding the claws, while the remaining parts are black or dark brown (sometimes, tarsomeres V are also black). As opposed to other species with orange legs, the metasoma and scutellum of *Sphex basilicus* are completely black. The males of this species are also recognizable by the shape of metasomal sternum VIII, which, like *Sphex bilobatus* and *Sphex latilobus*, carries two prominent lobes that are visible from above. Unlike these two species, where the lobes are the only visible part of sternum VIII and mostly straight, the lobes of *Sphex basilicus* are conspicuously curved upward, and a large, undivided portion of sternum VIII is also visible (Fig. 27B).

##### Description.

Body black, but the following are orange: distal part of forefemur, mid- and hindfemora at least below; tibiae, tarsi, and proximal half of claw. Forewing membrane yellow near base, fore- and hindwings with fuscous band at apex. Wing veins brown. Clypeus with narrow medial glabrous stripe. Distance between hind-ocelli 0.7× their shortest distance to compound eyes. Pubescence on collar and scutum golden, on scutum denser laterally than medially. Scutellum convex, with medial impression. Propodeal enclosure with long, somewhat dense, golden setae; sculpture not completely concealed.

*Female*: Body length 27.2–31.2 mm. Forebasitarsal rake with eight long spines. Appressed pubescence and erect setae on clypeus and frons golden. Free clypeal margin medially with indistinct emarginations and an indentation directly above, distance between lobes less than 1/8 length of flagellomere II. Length of petiole 1.2× length of flagellomere II. Tomentum golden, dense on metasomal tergum I, moderately dense on tergum II.

*Male*: Body length 34.4–43.6 mm. Metasomal sternum VIII orange. Appressed pubescence and erect setae on clypeus and frons silvery-white. A single lobe emerging medially and slightly posteriorly from free clypeal margin. Length of petiole 1.6× the length of flagellomere II. Tomentum golden, very dense on metasomal tergum I where it is also interspersed with dense, long, erect golden setae, moderately dense on tergum II. Metasomal terga V and VI with long, golden setae facing posteriorly, especially at apical margin. Metasomal sterna mostly glabrous, several long golden setae laterally on sterna V–VII. Metasomal sternum VIII with two lobes that are curved upward and diagonally truncate posteriorly.

##### Notes on type material.

The type of *Sphex basilicus* was not examined because the character combination in the original description (black body, golden pubescence on clypeus and propodeum, partially ferruginous legs) is sufficient to unambiguously identifiy this species.

#### Sphex bilobatus

Taxon classificationAnimaliaHymenopteraSphecidae

Kohl, 1890

Sphex canescens F. Smith, 1856: 246, ♀, ♂, junior primary homonym of *Sphex canescens* Scopoli, 1786. Syntypes: Australia: no specific locality (BMNH). Not examined.
Sphex bilobatus Kohl, 1895: 59, ♀, ♂. Syntypes: Australia: South Australia: Adelaide (ZMB). Synonymized with *Sphex canescens* F. Smith, 1856, by Turner (1910a: 346). Syntypes examined.

##### Material examined.

Syntypes (of *Sphex bilobatus*). ♀, ♂ **AUSTRALIA:** **SA:** Adelaide (ZMB).

##### Other material.

**[COUNTRY UNKNOWN]:** **[state unknown]:** [no specific locality], 1♀, 14.11.1944 (AMS). **AUSTRALIA:** **ACT:** Cotter Road, Weston Creek, 1♀, 10.01.1931, G. Jefferies (ANIC); **NSW:** 6 km N of Bilpin, Blue Mountains, 1♂, 27.11.1979, N. W. Rodd (AMS); 6 km NE of Bilpin, Blue Mountains, 1♂, 14.11.1980, N. W. Rodd (AMS), 1♀, 22.01.1988, N. W. Rodd (AMS); Blue Mountains, 1♀, 01.02.1915, A. Musgrave (AMS); 3 km S of Blue Mountains, 1♂, 19.01.1976, N. W. Rodd (AMS); Calumet, 26 km NE of Binnaway, 1♂, 29.12.1932, C. F. Garnsey (AMS); Clarence, Blue Mountains, 1♂, 23.09.1980, N. W. Rodd (AMS), 1♀, 21.01.1983, N. W. Rodd (AMS), 1♂, 07.01.1984, N. W. Rodd (AMS), 1♀, 10.01.1985, N. W. Rodd (AMS), 2♀, 27.01.1985, N. W. Rodd (AMS), 1♀, 27.01.1992, N. W. Rodd (AMS); 40 km W of Cobar, 1♂, 12.11.1985, N. W. Rodd (AMS); French’s Forest, Sydney, 1♀, 1♂, 09.11.1912, A. Musgrave (AMS); Goarra Ridge Trail, Royal National Park, 1♀, 29.01.1979, G. Daniels (AMS); 25 km SE Grafton, Yuraygir Crown Reserve, 25°53'S, 153°05'E, 1♀, 06.01.2009, D. Bray (AMS); Guyra, 1♀, 01.02.1992, E. N. McKie (AMS); Jinki Creek, Blue Mountains, 1♀, 10.01.1982, N. W. Rodd (AMS); Kuringgai Chase, 20 miles N of Sydney, 1♀, 04.-08.01.1970, H. E. Evans (ANIC); Lane Cove, 1♂, 12.12.1943 (AMS); Lightning Ridge, 1♀, 14.10.1989, I. D. Buddle (AMS); Loftus, 1♀, 11.12.1957, G. Dobcal (AMS); Mount Banks, Blue Mountains, 1♀, 27.11.1980, N. W. Rodd (AMS); Mount Tomah, Blue Mountains, 1♀, 20.12.1980, N. W. Rodd (AMS); Mount Towinhingy, near Kandos Weir, 1♀, 30.12.1977, G. Daniels (AMS); Mount Victoria, Blue Mountains, 1♀, 28.12.1981, N. W. Rodd (AMS); Mount Vincent, near Running Stream, 1♂, 29.12.1977, G. Daniels (AMS); Mount York, Blue Mountains, 1♂, 22.12.1980, G. & T. Williams (AMS), 1♀, 20.01.1982, N. W. Rodd (AMS); Pearl Beach, 1♀, 04.01.1979, John Alcock (ANIC); 11 km E of Three Ways, 32°47'S, 150°29'E, 1♂, 31.12.1977, G. Daniels (AMS); Warrumbungle National Park, 1♀, 13.-18.12.1977, G. A. Holloway (AMS); **SA:** Adelaide, ♂ (ZMB); **VIC:** Churchill National Park, Melbourne, 1♀, 1♂, 06.01.1976, M. S. & B. J. Moulds (AMS); Fernshaw, 1♀, 26.01.1955, A. N. Burns (ANIC); Healesville, 1♀, 1♂, Dec 1913, M. Arnold (BMNH); Janalli, 1♂, 04.12.1932, Stoyles (AMS); near Lake Hindmarsh, Big Desert National Park, 1♂, 29.-30.11.1992, Moulds, McAlpine, McEvey (AMS); **WA:** Yallalong Homestead, 1♂, 29.11.1999, M. S. Moulds & M. Humphrey (AMS).

The collecting localities are shown in Fig. 34B.

##### Diagnosis.

**Figure 28.**
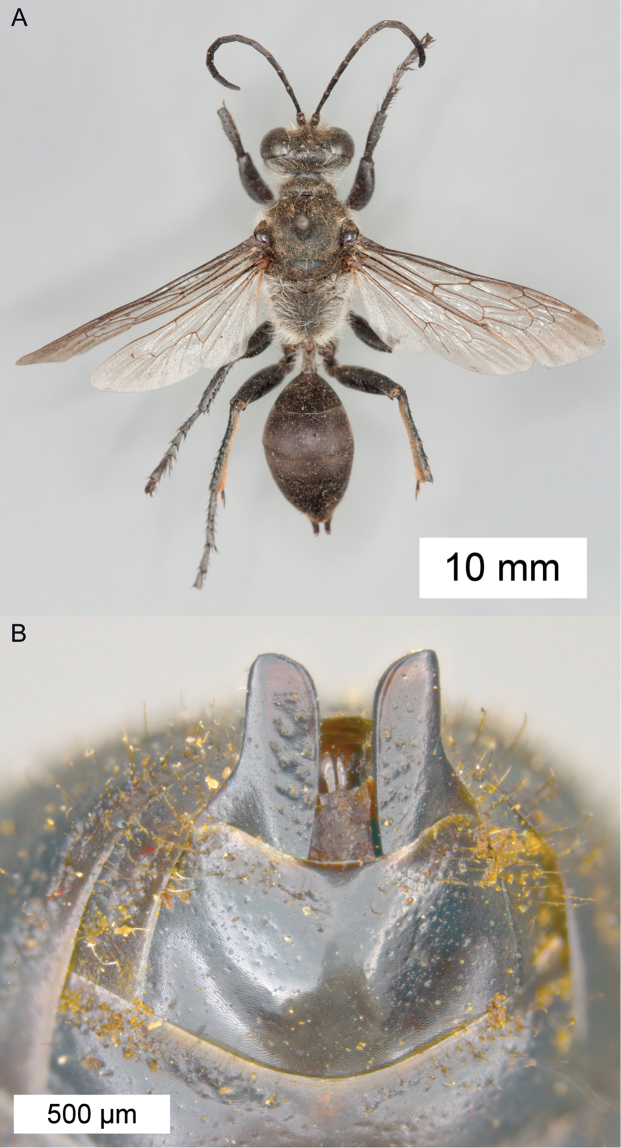
*Sphex bilobatus*, ♂. **A** habitus **B** ventral view of metasomal sterna VII and VIII.

Females of *Sphex bilobatus* differ from the other Australian *Sphex* by the following character combination: metanotum without tubercles, metasoma black, wing veins light to dark brown, appressed pubescence and erect setae on clypeus silvery-white, and scutellum convex medially. Males of *Sphex bilobatus* are also characterized by their metasomal sternum VIII, which consists of two large separate lobes (Fig. 28B). This character is only shared with males of two other Australian species. One of them, *Sphex basilicus*, has a single lobe emerging from below the center of the free clypeal margin, is much larger in size and has orange pubescence on the propodeal enclosure, whereas *Sphex bilobatus* does not have lobes on the free clypeal margin, and its propodeal pubescence is silvery. In the other one, *Sphex latilobus*, the free clypeal margin is bright orange, while that of *Sphex bilobatus* is black.

##### Description.

Body length 19.0–22.6 mm. Body black. Wing membrane hyaline, forewing with slightly fuscous band at apex. Wing veins light to dark brown. Free clypeal margin medially with broad, insignificant lobe that emerges slightly posteriorly of clypeal surface. Appressed pubescence and erect setae on clypeus and frons silvery-white. Clypeus with medial glabrous stripe. Pubescence on collar and scutum silvery-white, on scutum not significantly denser laterally than medially. Scutellum convex, with medial impression. Propodeal enclosure with sparse, silvery-white pubescence, sculpture well visible. Length of petiole 1.4× length of flagellomere II. Tomentum rather sparse on metasomal tergum I, moderately dense on tergum II.

*Female*: Hindwing entirely hyaline. Forebasitarsal rake with 11 long spines. Free clypeal margin slightly scoop-shaped. Distance between hind-ocelli slightly smaller than their shortest distance to compound eyes.

*Male*: Hindwing with slight fuscous band at apex. Distance between hind-ocelli equal to their shortest distance to compound eyes. Metasomal terga V and VI with few bristles. Metasomal sterna II–VII with few, erect silvery setae posterolaterally. Visible part of metasomal sternum VIII forming two separated lobes.

##### Notes on type material.

The types of *Sphex canescens* F. Smith, 1856 (the original, invalid name of the species) were not examined, but we did study the types of *Sphex bilobatus* Kohl, 1890, which was synonymized with *Sphex canescens* by R. Turner (1910).

#### Sphex brevipetiolus sp. n.

Taxon classificationAnimaliaHymenopteraSphecidae

http://zoobank.org/4E8DDB2F-C1FE-45C9-9B96-62EBF4793BF5

##### Material examined.

*Holotype*. ♀, **AUSTRALIA:** **WA:** 3 miles N of Moora, 05.01.1966, J. A. Grant (BMNH).

The collecting locality is shown in Fig. 35B.

##### Diagnosis.

*Sphex brevipetiolus* (of which only the female is known) is one of the few species in the *Sphex subtruncatus* group with a petiole shorter than flagellomere II. It differs from females of *Sphex ermineus* and *Sphex corporosus*, which are similar, in having bright orange veins near the wing base and a markedly convex scutellum with a distinctly developed impression medially. *Sphex ermineus* has black wing veins, and the scutellum of *Sphex corporosus* is flatter and often lacks an impression.

##### Description.

*Female*: Body length 31.6 mm. Body black. Wing membrane hyaline, with fuscous spot beyond marginal cell. Wing veins orange, darker near apex. Forebasitarsal rake with nine long spines. Free clypeal margin with two small lobes medially, distance between them less than 1/8 length of flagellomere II. Appressed pubescence and erect setae on clypeus and frons silvery-white. Clypeus with medial glabrous stripe. Distance between hind-ocelli 0.8× their shortest distance to compound eyes. Pubescence on collar, scutum, metanotum and propodeum silvery-white, on scutum denser laterally. Scutellum convex, with medial impression. Pubescence on propodeal enclosure not concealing sculpture. Length of petiole 0.8× length of flagellomere II. Tomentum moderately dense on metasomal tergum I, slightly denser on tergum II.

*Male*: Unknown.

**Figure 29.**
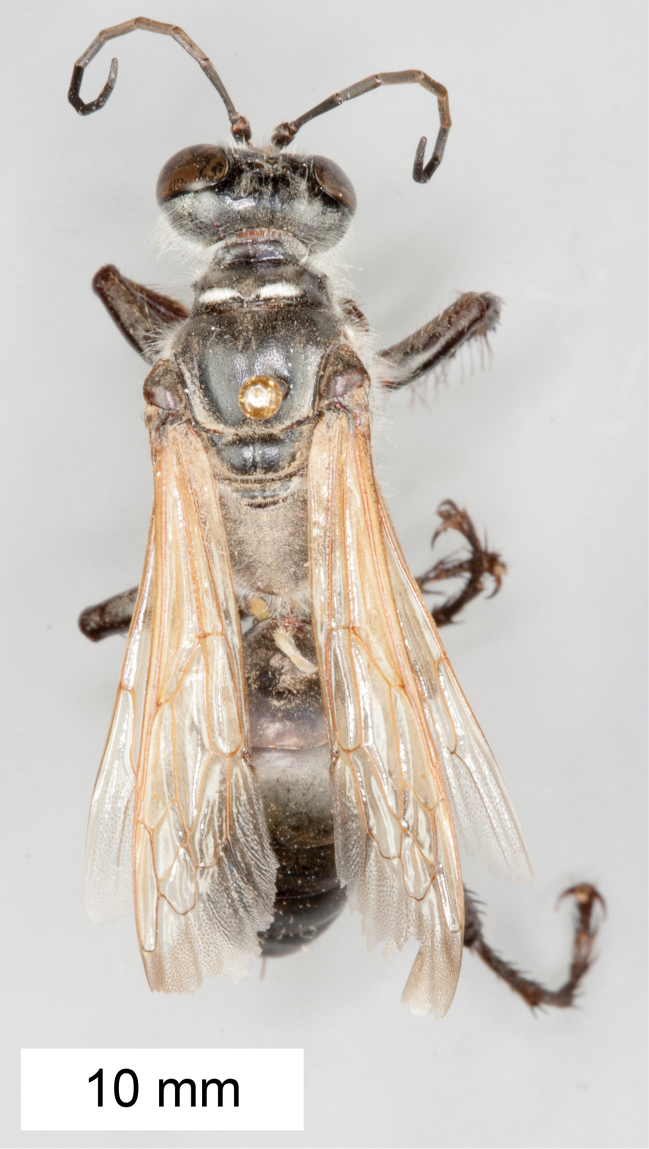
Habitus of *Sphex brevipetiolus*, ♀.

##### Discussion.

In the *Sphex subtruncatus* group, there are eight species of which females are yet unknown or where matching of males and females was first proposed in this study. *Sphex brevipetiolus* can theoretically be the female of one of them. Of these, only three have wing veins that are not uniformly dark. One of them (*Sphex flammeus*) has a bright orange metasoma and largely orange legs. The second one (*Sphex pretiosus*) has a sharp transition between golden and silvery pubescence on the propodeum and, like the former, a petiole that is considerably longer than flagellomere II. Finally, the reasons for ruling out *Sphex corporosus* are given in the respective discussion.

##### Etymology.

*Brevipetiolus* is a composite of the Latin adjective *brevis* (short) and the noun *petiolus* (stem), referring to the short petiole of this species.

#### Sphex caelebs sp. n.

Taxon classificationAnimaliaHymenopteraSphecidae

http://zoobank.org/098ACC9F-159B-4CE4-AEE7-43671DE423AA

##### Material examined.

*Holotype*. ♂, **AUSTRALIA:** **WA:** Westonia, 31°11'53"S, 118°45'31"E, 15.03.2007, L. C. & M. G. Brooker (AMS).

The collecting locality is shown in Fig. 42D.

##### Diagnosis.

**Figure 30.**
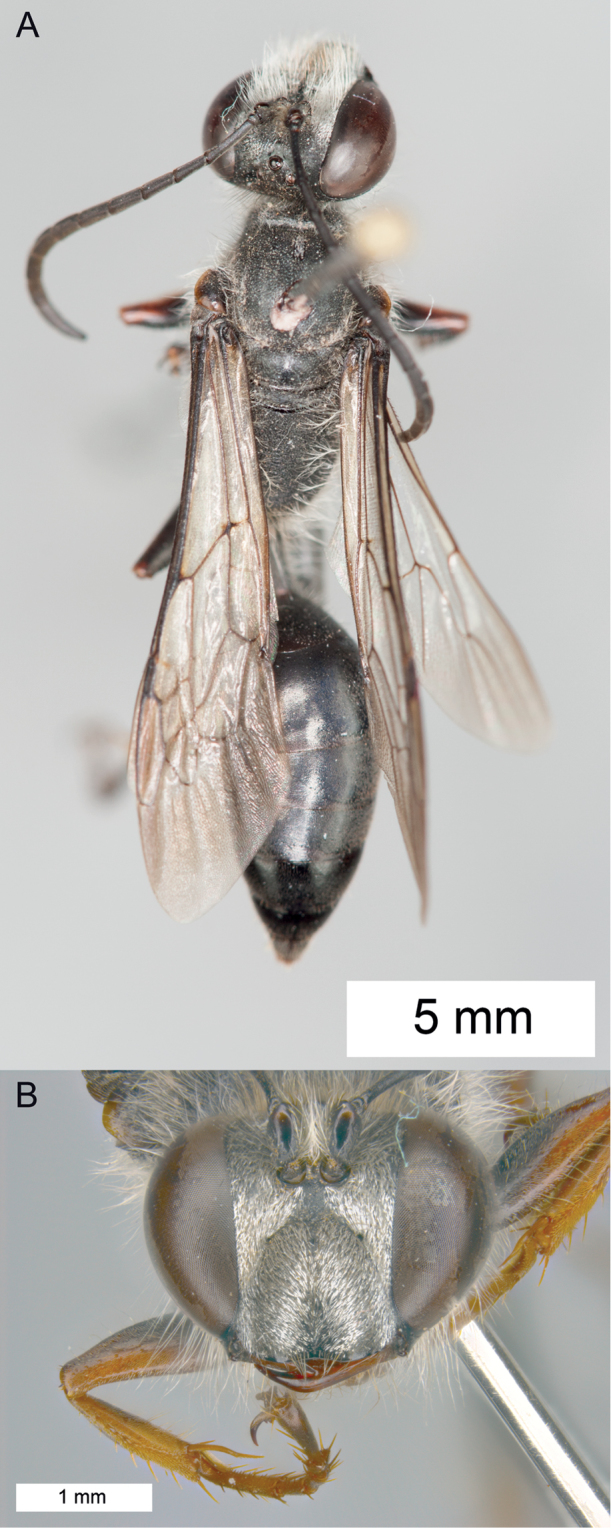
*Sphex caelebs*, ♂. **A** habitus **B** frontal view.

The presence of a few dark erect setae on the clypeus combined with partially orange legs (mainly parts of the anterior surface of the foreleg, as seen in Fig. 30B, and the inner hindtibial spur including pecten) make *Sphex caelebs* unique among the male Australian *Sphex* (the female of this species is unknown). The habitus and mesosomal sculpture resemble some *Sphex modestus*, which possesses an only inconspicuously raised metanotum and dense white or yellowish tufts of setae on the metasomal sterna that are absent in *Sphex caelebs*.

##### Description.

*Female*: Unknown.

*Male*: Body length 18.1 mm. Body black, but the following are orange-brownish: basal half of mandible, anterior surface of foreleg from distal half of femur up to tarsomere IV, distal half of anterior surface of midfemora, inner hindtibial spur including pecten, basal half of claw. Wing membrane hyaline, forewing with slightly fuscous band at apex. Wing veins dark brown. Free clypeal margin simple, concave medially. Appressed pubescence on clypeus and frons silvery-white, a few dark erect setae on clypeus, remaining erect setae on clypeus as well as those on frons uniformly silvery-white. Clypeus medioventrally with narrow glabrous stripe. Distance between hind-ocelli nearly equal to their shortest distance to compound eyes. Pubescence on mesosoma silvery-white, on scutum slightly denser laterally. Scutellum convex, with shallow medial impression. Pubescence on propodeal enclosure sparse, leaving sculpture fully visible. Length of petiole 1.6× length of flagellomere II. Tomentum moderately dense on metasomal tergum I and II. Metasomal terga V and VI sparsely covered with erect silvery setae. Metasomal sterna II–VI mostly glabrous, apical half of sternum VII and all sternum VIII covered with silvery pubescence. Metasomal sternum VIII entire, its lateral margin straight.

##### Discussion.

It must also be assessed if *Sphex caelebs* is the undescribed male of an already known species. In the *Sphex subtruncatus* group, there are seven species of which males are yet unknown or where matching of males and females was first proposed in this study. In none of these, however, is the leg coloration comparable to *Sphex caelebs*, and additional characters indicate its status as a separate species. For example, only two of the seven species also have dark erect setae on the clypeus. These two, however, are much larger than *Sphex caelebs*; one of them, *Sphex ahasverus*, has darkened wings and golden pubescence on the propodeum and the other one, *Sphex corporosus*, has an unusually short petiole, the characters that are lacking in *Sphex caelebs*.

##### Etymology.

*Caelebs* is a Latin noun meaning “unmarried man”, referring to the fact that the species is currently known only from a single male.

#### Sphex cognatus

Taxon classificationAnimaliaHymenopteraSphecidae

F. Smith, 1856

Sphex cognatus F. Smith, 1856: 248, ♀ (as *cognata*, incorrect original termination). Holotype or syntypes: Australia: no specific locality (BMNH). Not examined.
Sphex formosus F. Smith, 1856: 254 (as *formosa*, incorrect original termination). Holotype or syntypes: ♂, Indonesia: Seram: no specific locality (BMNH). Synonymized with *Sphex cognatus* by Turner (1910a: 345). Not examined.

##### Material examined.

**[COUNTRY UNKNOWN]:** **[state unknown]:** [no specific locality], 2♀, 2♂ (AMS). **AUSTRALIA:** **[state unknown]:** [no specific locality], 1♀, 1♂ (BMNH); “Coopers Pk”, 1♂, 21.02.1965, J. Varley (AMS); **NSW:** Ashcroft, 1♂, 01.01.1977, S. Brousek (AMS); Ballina, 1♀, 16.12.1930, C. E. Chadwick (AMS), 1♀, 07.12.1989, D. J. Scambler (AMS); Bill Weiley Bridge, Esk River near Fluka, 2♀, 31.12.1978, B. J. Day (AMS); Bilpin near Kurrajong, 1♀, 2♂, 28.01.1981, N. W. Rodd (AMS), 1♂, 03.02.1981, N. W. Rodd (AMS); 6 km NE of Bilpin, Blue Mountains, 1♀, 20.01.1983, N. W. Rodd (AMS), 1♀, 06.04.1984, N. W. Rodd (AMS); Brushgrove, 1♂, 04.04.1973, G. A. Holloway (AMS); Cabramatta, 1♀, 01.12.1962, M. I. Nikitin (BMNH); Cheltenham, 1♂, 14.01.1951 (AMS); 3-5 km NE of Harrington, 1♂, 02.02.1991, G. Williams (AMS); Kensington, 1♀, 19.02.1976, Fluhoelhower (AMS), 1♀, 20.02.1976, Fluhoelhower (AMS), 1♀ (AMS); Lane Cove, 1♂, 05.02.1944 (AMS), 1♀, 21.01.1945 (AMS); Lennox Head, 1♀, 04.04.2001, M. Elliott (AMS); McKanes Bridge, Coxs Road, 2♀, 26.01.1991, R. de Keyzer & G. A. Clark (AMS); Middle Pocket, 1♀, 12.01.1929, C. E. Chadwick (AMS); North Narrabeen, 1♂, 28.02.1954, M. I. Nikitin (BMNH); Pearl Beach, 1♀, 16.03.1974, D. Feughelman (AMS), 1♀, 28.01.1989, C. A. P. Urquhart (AMS), 1♂, 29.01.1989, C. A. P. Urquhart (AMS); Randwick, 1♀, 08.01.1965, J. Varley (AMS); 16 km W of South Grafton, 1♀, 05.01.1978, G. Daniels (AMS); Woronora, 1♂, 22.01.1982, M. L. Bason (AMS); **NT:** Groote Eylandt, 1♀, 06.02.1925, G. H. Wilkins (BMNH); Litchfield National Park: Wangi Falls, 13°09.7'S, 130°40.9'E, 1♂, 21.04.2008, W. J. Pulawski & G. A. Williams (CAS); 29 km NW Mataranka, 14°45.5'S, 132°51.1'E, 1♂, 05.04.2008, W. J. Pulawski & G. A. Williams (CAS); Port Darwin, 1♂ (BMNH); **QLD:** [no specific locality], 2♂ (BMNH); “Mid Queensland”, 2♂ (BMNH); “North Queensland”, 1♂ (BMNH); Agnes Water, 40 km E of Miriam Vale, 1♂, 04.01.1984, N. W. Rodd (AMS), 1♀, 04.11.1984, N. W. Rodd (AMS); Archer River crossing Coen Cape York Road, 1♂, 30.10.1974, M. S. Moulds (AMS); Ball Bay near Cape Hillsborough, 1♀, 26.06.1985, N. W. Rodd (AMS); Ball Bay near Mackay, 1♀, 09.10.1984, N. W. Rodd (AMS); Bertie Creek, 11°46'S, 142°36'E, 1♀, 21.03.1992, S. F. McEvey (AMS); Bloomfield, 1♀, 01.12.1980, T. M. Moulds (AMS); Brisbane, 1♀, 2♂, 09.09.1953, F. G. T. Smith (BMNH); Byfield State Forest, 1♀, 31.12.1975, G. Daniels (AMS); Cairns, 1♀, 09.-30.12.1962, E. C. Corbet (BMNH); Cape York, 1♂, 28.05.1991, N. W. Rodd (AMS); Cape York Peninsula, Iron Range, 1♀, 20.05.1974, M. Walford-Huggins (AMS); Claudie River near Mount Lamond, 1♀, 21.12.1971, D. K. McAlpine & G. A. Holloway (AMS); Middle Claudie River, Iron Range, 1♀, 23.09.1974, M. S. Moulds (AMS), 1♂, 07.10.1974, G. Daniels (AMS); Clermont, 1♂, K. K. Spence (AMS); Clohesy River, Kuranda, 1♂, 09.11.1972, O. W. Richards (BMNH); near Dalga, 24°31'39.1"S, 151°28'15.6"E, 1♂, 13.04.2006, D. R. Britton & J. R. Weiner (AMS); Iron Range, 1♂, 16.10.1974, M. S. Moulds (AMS), 1♂, 20.04.1975, M. S. Moulds (AMS); Lizard Island, NNE of Cooktown, 1♂, 17.11.1974, M. S. & B. J. Moulds (AMS), 1♀, 18.11.1974, M. S. & B. J. Moulds (AMS); Mackay, 1♂, 1947, A. Marriage (AMS); Moore Park, Bundaberg, 1♀, 20.04.1973 (AMS); Murray Island, 1♀, Aug-Oct 1907, Hedley & McCullock (AMS); 11 km from Portland Roads on Iron Range Road, 1♀, 21.09.1974, M. S. Moulds (AMS); Proserpine, 1♀, 08.12.1971, D. K. McAlpine & G. A. Holloway (AMS); Redlynch, 1♂, Dec 38, R. F. Sternitzky (BMNH); Walsh River, Kuranda, 1♂, 14.11.1972, O. W. Richards (BMNH); Wondecla near Herberton, 1♂, 06.01.1990, M. S. & B. J. Moulds (AMS); Woodgate, 35 km E of Childers, 1♀, 06.11.1984, N. W. Rodd (AMS); Yeppoon, 3♀, 17.11.1978, R. Eastwood (AMS); **SA:** Adelaide, 1♀, 20.05.1898 (ZMB). **INDONESIA:** **Maluku Province:** Ambon Island, 1♂ (ZMB); Jilo, Seram Island, 1♂, 1884, C. Rubbe (ZMB); **North Maluku Province:** Sanana Island, 1♂, Doherty (ZMB); **Papua:** 30 km S Nabire, 1♀, 26.07.1998, Balke (NHMW); Yerelua, 2♀, 26.07.1998, Balke & Konyorah (NHMW); **West Papua Province:** Wondiwoi Mountains, Wasior, 1♂, 01.07.1926, E. Mayr (ZMB), 1♀, 01.07.1928, E. Mayr (ZMB). **PAPUA NEW GUINEA:** **[province unknown]:** [no specific locality], 1♀, 1896, Biró (ZMB), 1♂, 29.06.1899, Ramu Expedition (ZMB), 1♀, 1♂, Ramu Expedition (ZMB); **East New Britain Province:** Mutupit, 10 km W Warangoi, 4°29'S, 152°07'E, 2♂, 06.06.2003, T. Osten (ZMB); “Queen Emmas Bath”, 5 km W Kokopo, 4°28'S, 152°19'E, 1♂, 09.-10.06.2003, T. Osten (ZMB); Rabaul, 1♂, May 1902, H. Schoede (ZMB), 1♀, 2♂, May 1905, H. Schoede (ZMB); Ralum, 1♂, 14.05.1886, F. Dahl (ZMB), 3♀, 2♂, 14.05.1896, F. Dahl (ZMB), 2♀, 5♂, 1896-1897, F. Dahl (ZMB), 1♀, 23.05.1891, F. Dahl (ZMB), 1♀, 1♂, F. Dahl (ZMB); Vudanplata, 15 km W Keravat, 4°12'S, 152°00'E, 1♀, 05.-13.06.2003, T. Osten (ZMB); Vunabaur, 30 km S Kokopo, 4°28'S, 152°19'E, 4♂, 07.-12.06.2003, T. Osten (ZMB); **Maluku Province:** around Tehoru, Seram Island, 1♀, 23.02.1989, Schillhammer (ZMB); **Morobe Province:** Finschhafen, 1♂, 1910, Hertle (ZMB); Sialum, 2♀, Nov-Dec 1909, Neuhauss (ZMB); **Oro Province:** Mount Lamington, 1♂, 1927, C. T. McNamara (AMS), 1♂, 01.07.1927, C. T. McNamara (AMS); **West New Britain Province:** 5 km E Kimbe, 5°32'S, 150°10'E, 2♀, 1♂, 18.-27.06.2003, T. Osten (ZMB), Lamavoro, 10 km S Hoskins, 5°28'S, 150°26'E, 1♂, 21.06.2003, T. Osten (ZMB), Makasili, 20 km E Hoskins, 5°28'S, 150°26'E, 1♂, 19.-24.06.2003, T. Osten (ZMB), Namundo Mill, 20 km W Kimbe, 5°30'S, 150°00'E, 1♀, 22.-25.06.2003, T. Osten (ZMB);3 km S Mosa, 5°38'S, 150°14'E, 1♀, 17.-20.06.2003, T. Osten (ZMB). **SOLOMON ISLANDS:** **Guadalcanal:** Lavoro Plantation, 1♂, 1925, C. E. Hart (AMS), 1♂, 07.11.1923, C. E. Hart (AMS), 1♂, 1926–1927, C. E. Hart (AMS); **Western Province:** Gizo, 1♀, 01.01.1974, N. L. H. Krauss (BMNH).

The collecting localities are shown in Fig. 40C.

##### Diagnosis.

Males of *Sphex cognatus* differ from all other Australian *Sphex* in combining the following features: two lobes emerge from the center of the free clypeal margin, the entire clypeus is densely covered with pubescence, and the pubescence on the propodeum conceals the sculpture. Females are distinguished from those of similar species by the same characters and additionally in having hyaline wings that are at least partially yellow tinged. By contrast, *Sphex ahasverus* possesses uniformly darkened wings, and the females of *Sphex argentatissimus* have no hint of yellow on their wings. Also, the similar *Sphex vestitus* has a longitudinal impression with slightly raised borders on the posterior half of its scutum, whereas *Sphex cognatus* has an even scutum.

##### Description.

Body length 21.8–27.6 mm. Body black. Forewing with rather distinct, hindwing with fainter fuscous band at apex. Free clypeal margin medially with two lobes, distance between them less than 1/8 length of flagellomere II. Appressed pubescence and erect setae on clypeus and frons golden. Clypeus entirely pubescent. Distance between hind-ocelli 1.1× their shortest distance to compound eyes. Pubescence on collar and scutum golden, the latter with denser, brighter pubescence laterally. Scutellum markedly convex. Propodeal enclosure with dense appressed and more sparse, erect, either golden or silvery pubescence, sculpture almost completely concealed. Length of petiole 1.6× length of flagellomere II. Tomentum quite dense on metasomal tergum I and II, but very short.

*Female*: Fore- and hindwing membrane yellowish, sometimes only inconspicuously. Wing veins brown, sometimes partially orange. Lobes on free clypeal margin in same plane as clypeal surface. Forebasitarsal rake with 8–10 long spines. Scutellum without impression.

*Male*: Forewing membrane hyaline or with slight yellow tinge, hindwing membrane hyaline. Wing veins brown. Lobes on free clypeal margin emerge slightly posteriorly of clypeal surface. Scutellum with shallow medial impression. Metasomal tergum V with few, tergum VI with considerable number of silvery-golden bristles. Metasomal sterna IV–VIII each with increasingly dense brown pubescence apically. Apical margin of metasomal sternum VIII arcuate, its lateral margin concave.

**Figure 31.**
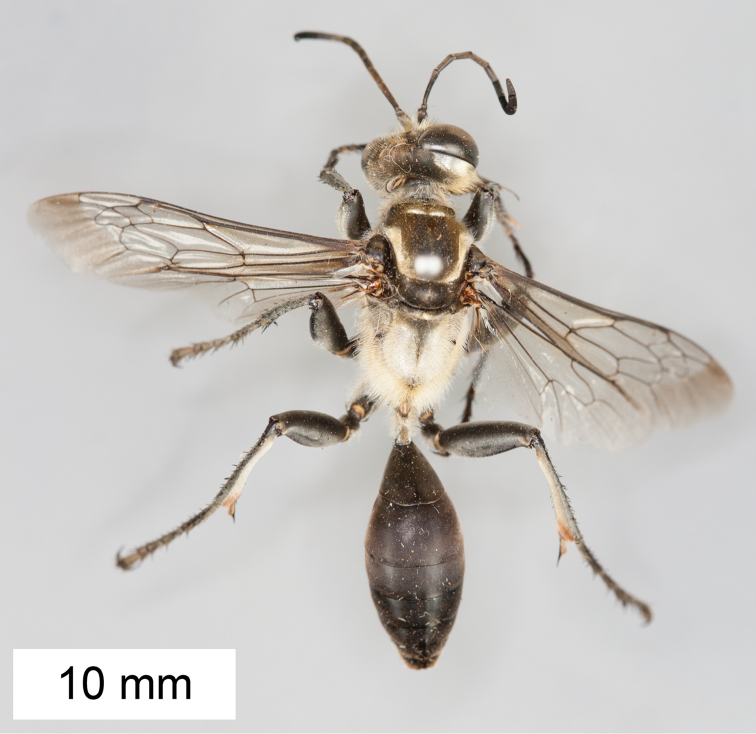
Habitus of *Sphex cognatus*, ♂.

##### Notes on type material.

The types of *Sphex cognatus* and its synonyms were not examined, because the character combination in the redescription of the type of its synonym *Sphex formosus* F. Smith, 1856 by Kohl (1890) (black body, golden pubescence on face and propodeum, convex scutellum, wing membrane sometimes yellowish), which was synonymized with *Sphex cognatus* by Turner (1910), is sufficient to unambiguously identify this species.

#### Sphex corporosus sp. n.

Taxon classificationAnimaliaHymenopteraSphecidae

http://zoobank.org/4909EF6D-25AA-40EA-A189-1F490DFC3A4F

##### Material examined.

*Holotype*. ♂, **AUSTRALIA:** **NSW:** Pooncarie, 26.11.1992, N. W. Rodd (AMS). *Paratypes*. **AUSTRALIA:** **NSW:** “20 SW” of Bourke, 2♀, 1♂, 28.10.1949, E. F. Riek (ANIC); Broken Hill, 1♀, 27.02.1941, C. E. Chadwick (AMS), 1♂, 26.12.1942, C. E. Chadwick (AMS), 1♂, 31.10.1943, C. E. Chadwick (AMS), 1♂, 03.12.1947, C. E. Chadwick (AMS), 1♂, 11.11.1985, N. W. Rodd (AMS), Hay, 1♂, 27.11.1992, N. W. Rodd (AMS); 30 km N of Euston, 1♂, 28.11.1988, N. W. Rodd (AMS); 20 km N of Menindee, 1♀, 1♂, 10.11.1985, N. W. Rodd (AMS); Narromine, 1♀ (AMS); Pooncarie, 3♀, 1♂, 28.11.1992, N. W. Rodd (AMS); Round Hill Nature Reserve, 1♀, 27.12.1976, G. Daniels (AMS), 1♀, 25.10.1977, G. Daniels (AMS); 75 km W of Wilcannia, 4♂, 09.11.1985, N. W. Rodd (AMS); **QLD:** 20 S of Tickalara, 1♂, 14.09.1949, E. F. Riek (ANIC); **SA:** Adelaide, 1♀ (ZMB); Lake Gilles Conservation Park, 1♀, 01.02.1995, L. Packer, M. Schwarz, P. Hurst, Y. Pamula (ZMB); 60 km W of Nullarbor, 31°34'S, 130°15'E, 1♀, 14.12.1995, M. S. & B. J. Moulds & K. A. Kopestonsky (AMS); Wilpena Pound Resort, 1♀, 18.01.1976, M. S. & B. J. Moulds (AMS); **VIC:** [no specific locality], 1♀, 1909, C. French (ANIC); Swan Island, 1♂ (BMNH); **WA:** Champion Bay Beach, 1♂ (BMNH); Hamelin Telegraph Station, 26°23.9'S, 114°09.9'E, 1♀, 1♂, 08.11.2008, V. Ahrens & W. J. Pulawski (CAS), 3♂, 10.11.2008, V. Ahrens & W. J. Pulawski (CAS); Kalbarri National Park: Ross Graham Lookout, 27°48.6'S, 114°28.3'E, 1♂, 06.11.2008, V. Ahrens & W. J. Pulawski (CAS); Marloo Station, 1♀, 01.01.1936, A. Goerling (ZMB), 1♀, Feb–Mar 35, A. Goerling (ZMB), 1♀, Oct–Nov 34, Gebr. Goerling (ZMB); Shire of Northampton, Kalbarri National Park, 27°39'13"S, 114°27'24"E, 1♀, 10.01.2010, L. Breitkreuz (ZMB); Tuckanarra Hill, 2♂, 16.11.1961, A. Snell (AMS); Urawa Nature Reserve ca 5 km N Mullewa, 28°29.6'S, 115°29.5'E, 1♂, 11.11.2008, V. Ahrens & W. J. Pulawski (CAS); Wydgee Station near Mount Magnet, 2♂, 16.11.1961, A. Snell (AMS). **INDONESIA:** **Bali Island:** Dedari, 1♀, 01.01.1948 (BMNH).

##### Diagnosis.

**Figure 32.**
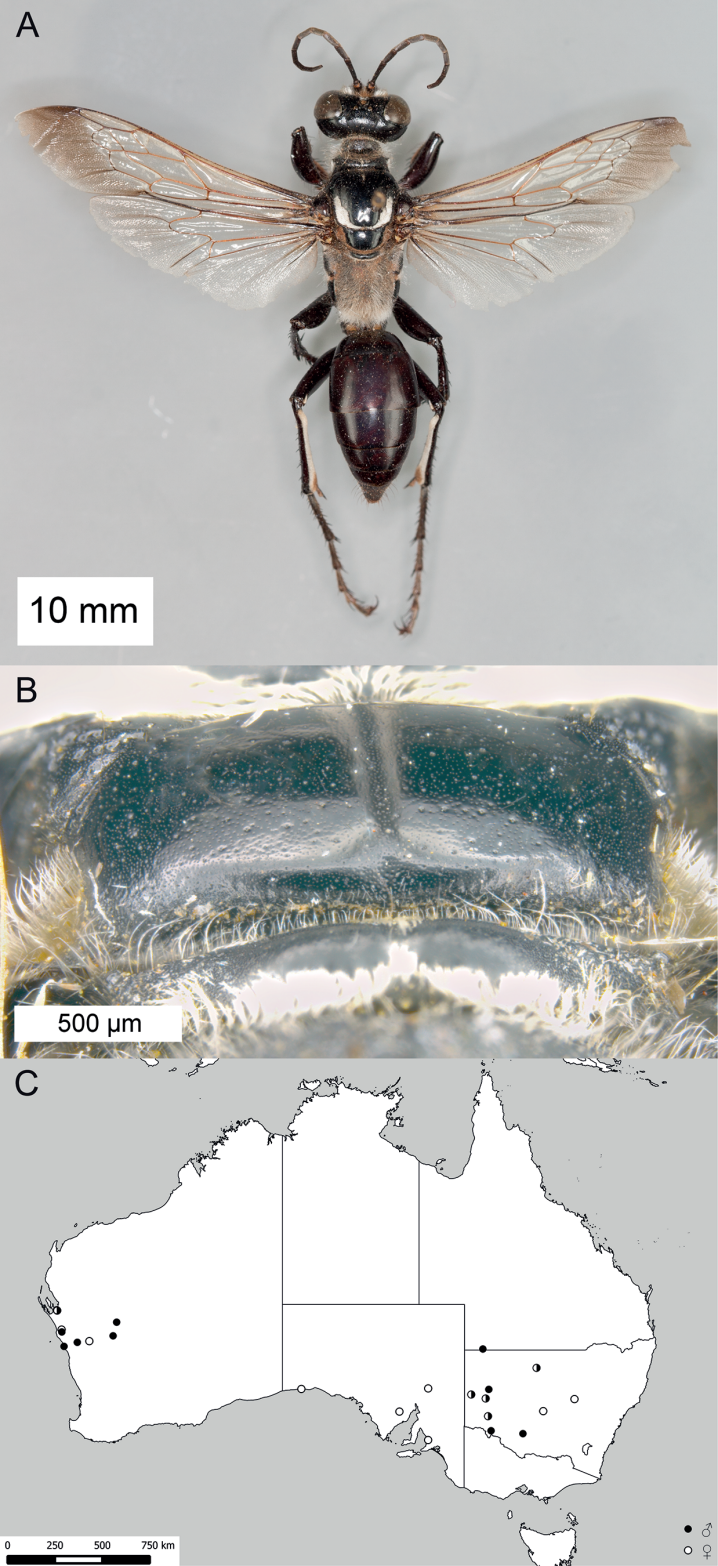
*Sphex corporosus*. **A** ♀, habitus **B** ♀, posterodorsal view of scutellum and metanotum **C** collecting localities, the combined symbol indicates that males and females were found in the same locality.

*Sphex corporosus* can be distinguished from most other members of the *Sphex subtruncatus* group through a combination of the following characters: petiole shorter than flagellomere II and pubescence on propodeal enclosure not concealing sculpture. Presence of these traits is sufficient to identify males, while females of *Sphex corporosus* resemble those of *Sphex brevipetiolus*. The former, however, has a flat scutellum (Fig. 32B) as well as dark erect setae on the clypeus, whereas in the latter the scutellum is convex and only silvery-white erect setae are present on the clypeus.

##### Description.

Body black, mandible medially with ferruginous stripe, legs and metasoma dark maroon to black. Wing membrane hyaline, forewing with fuscous spot beyond marginal cell. Wing veins dark orange to dark brown. Appressed pubescence on clypeus and frons as well as erect setae on frons silvery. Pubescence on mesosoma silvery-white. Propodeal enclosure covered with long, erect, silvery-white pubescence, not concealing sculpture. Pubescence denser on posterior end of propodeum.

*Female*: Body length 27.5–31.8 mm. Apical margin of forewing fuscous beyond submarginal cell III, medial cell II and cubital cell II. Forebasitarsal rake with 10–11 long spines. Free clypeal margin slightly scoop-shaped, clypeus elevated medially above margin. Erect setae on clypeus black. Most of central clypeal area glabrous. Distance between hind-ocelli 0.9× their shortest distance to compound eyes. Scutum glabrous except for area between admedian lines and lateral and posterior margins. Scutellum flat, sometimes with shallow medial impression. Length of petiole 0.7× length of flagellomere II. Tomentum moderately dense medially on metasomal tergum I, metasomal tergum II glabrous. Metasomal terga V and VI with few bristles.

*Male*: Body length 20.3–27.4 mm. Apical margin of forewing nearly hayaline beyond submarginal cell III, medial cell II and cubital cell II. Free clypeal margin truncate, slightly concave towards center. Erect setae on clypeus silvery. Clypeus with narrow medial glabrous stripe which broadens towards free clypeal margin. Distance between hind-ocelli 1.3× their shortest distance to compound eyes. Pubescence on scutum much denser laterally. Scutellum convex, with medial impression. Length of petiole 0.8× length of flagellomere II. Metasomal tergum I and anterior half of metasomal tergum II very densely covered with silvery-white tomentum. Metasomal sternum VII with small fringe of dark bristles posterolaterally. Metasomal sternum VIII notched apically, its lateral margin concave.

##### Variation.

In most of the examined specimens, the metanotum is moderately raised, and in a few with an inconspicuous median impression. A raised metanotum with an impression is usually distinctive for species of the *Sphex argentatus* group, but since the raising of the metanotum and the depth of the impression in the most extreme known cases of this species are only comparable to the least extreme cases of examined specimens of the *Sphex argentatus* group, *Sphex corporosus* is tentatively placed in the *Sphex subtruncatus* group.

##### Discussion.

Save for two other species besides *Sphex corporosus*, all members of the *Sphex subtruncatus* group have a petiole longer than flagellomere II. One of them, *Sphex ermineus*, is set apart by having exceptionally long and dense pubescence on the propodeal dorsum. Of the other one, *Sphex brevipetiolus*, only a single female is known. This specimen differs in a few features from the specimens that have been designated the females of *Sphex corporosus*, and partially surpasses them in resemblance to the males of *Sphex corporosus*. Arguments in favor of pairing males and females of *Sphex corporosus* are addressed in the subsequent paragraph.

The following attributes are shared by *Sphex brevipetiolus* and males of *Sphex corporosus*, but not between males and females of the latter: erect setae on clypeus uniformly silvery-white, and scutellum convex and medially impressed. However, the scutellar structure is usually not constant among sexes in the Australian *Sphex*. On the other hand, *Sphex brevipetiolus* has several features that differentiate it from both males and females of *Sphex corporosus*: considerably shorter tomentum on metasomal tergum I, brassy setae on metasomal sterna, and wing veins that are bright orange in the basal half of the wing. For these reasons, males and females of *Sphex corporosus* are considered to be one species, and *Sphex brevipetiolus* is treated as a separate one.

Lastly, the geographic range of both sexes is also indicative of the status as a single species, particularly since the number of examined individuals was rather large. Males and females were often found in close proximity to each other, or even in the same areas (Fig. 32C).

##### Etymology.

*Corporosus* is a Latin adjective, meaning “corpulent”. A few members of this species from the ANIC were already labeled with this name, although it is unclear by whom. There are no related publications, but the species will be named *Sphex corporosus* to minimize the risk of confusion in case there are more specimens already so labeled. It undoubtedly refers to the large size of the species.

#### Sphex ermineus

Taxon classificationAnimaliaHymenopteraSphecidae

Kohl, 1890

Sphex ermineus Kohl, 1890: 412, ♀. Syntypes: Australia: Western Australia: Swan River (NHMW). Not examined.

##### Material examined.

**AUSTRALIA:** **[state unknown]:** [no specific locality], 1♀ (ZMB);”North Australia”, 1♀ (BMNH); **NSW:** Bourke, 1♀, 03.01.1954, C. M. Moore (AMS); 25 miles SE of Coonabarabran, 1♀, 01.01.1973, A. Smith (AMS); Round Hill Nature Reserve, 1♀, 26.12.1976, G. Daniels (AMS); **NT:** Groote Eylandt, 1♀, 28.01.1925, G. H. Wilkins (BMNH); 27.5 km SE of Katherine, 14°34'0"S, 132°28'5"E, 1♂, 08.04.2008, G. Williams & W. Pulawski (AMS); Port Darwin, 1♀, 2♂ (BMNH); Stuart Point Road 14.5 km N Arnhem Highway, 12°43.6'S, 131°50.0'E, 1♀, 27.04.2008, W. J. Pulawski & G. A. Williams (CAS); **QLD:** “North Queensland”, 1♂ (BMNH); Bluff Range, Biggenden, 25°36'S, 152°03'E, 1♂, 21.12.1970, H. Frauca (ANIC); Cape York, 1♀, 01.08.1986, N. W. Rodd (AMS); Duaringa, 4♀, 26.12.1946, C. W. Smith (AMS); Goldsborough Road, Mulgrave River, 1♂, 19.01.1962, E. B. Britton & J. G. Brooks (BMNH); Heathlands, 11°45'S, 142°35'E, 1♀, 15.–26.01.1992, I. Naumann & T. Weir (ANIC); **WA:** Bunbury, 1♂, 01.01.1961, A. Snell (AMS); Marloo Station, 1♀, 02.03.1935, A. Goerling (ZMB).

The collecting localities are shown in Fig. 42D.

##### Diagnosis.

*Sphex ermineus* differs from all other Australian *Sphex* in the combination of a petiole distinctly shorter than flagellomere II, and the dense, long, silvery-white pubescence on the propodeal enclosure that conceals the sculpture. *Sphex corporosus* has a similar appearance, but its propodeal pubescence is less dense, leaving parts of the sculpture still visible.

##### Description.

Body length 28.8–30.8 mm. Body black. Wing membrane hyaline, forewing with dark fuscous spot beyond marginal cell. Wing veins black. Distance between hind-ocelli approximately 1.2× their shortest distance to compound eyes. Pubescence on collar and scutum silvery-white, denser laterally and posteriorly. Propodeal enclosure densely covered with silvery-white pubescence, sculpture completely concealed. Length of petiole approximately 0.9× length of flagellomere II. Tomentum on metasomal tergum II mostly absent.

**Figure 33.**
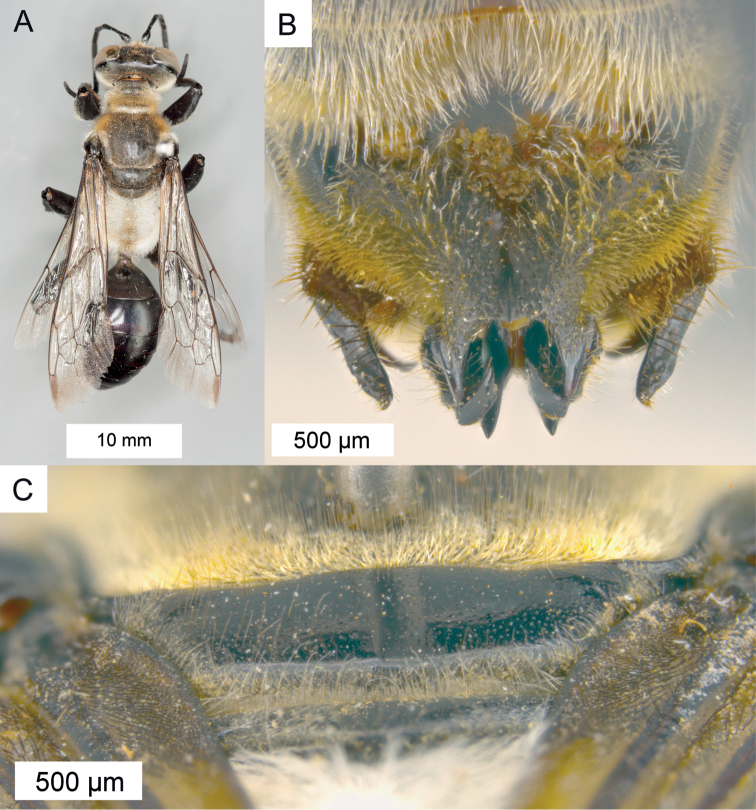
*Sphex ermineus*. **A** ♂, habitus **B** ♂, ventral view of metasomal sternum VIII **C** ♀, posterodorsal view of scutellum and metanotum.

*Female*: Forewing with fuscous band at apex. Forebasitarsal rake with 12 long spines. Free clypeal margin simple, bulging above center. Appressed pubescence on clypeus and frons silvery-white, erect setae on frons silvery-white, most erect setae on clypeus silvery-white, a few light or dark brown. Clypeus medioventrally with glabrous spot. Central area of scutum and scutellum glabrous. Scutellum and metanotum conspicuously flat, without impressions (Fig. 33C). Tomentum on metasomal tergum I moderately dense.

*Male*: Forewing almost hyaline at apex except for fuscous spot beyond marginal cell. Free clypeal margin concave medially. Appressed pubescence and erect setae on clypeus and frons golden. Clypeus with narrow medial glabrous stripe. Central area of scutum and scutellum pubescent. Tomentum on metasomal tergum I dense. Metasomal sternum I medially densely covered with long, silvery setae. Sterna II–VIII with increasingly dense fringes of silvery setae laterally, sterna VII and VIII completely covered. Metasomal sternum VIII anteriorly with large hole, conspicuously notched apically, its lateral margin concave (Fig. 33B).

##### Notes on type material.

The type of *Sphex ermineus* was not examined, because the character combination in the original description (dense silvery-white propodeal pubescence, flat scutellum, flat metanotum) is sufficient to unambiguously identify this species.

#### Sphex flammeus sp. n.

Taxon classificationAnimaliaHymenopteraSphecidae

http://zoobank.org/5D75E15A-9010-44E1-BB6B-CA0A92F54C80

##### Material examined.

*Holotype*. ♀, **AUSTRALIA:** **NT:** 50 km E of Three Ways, 11.04.1995, L. Packer (ZMB). *Paratypes*. **AUSTRALIA:** **SA:** Hermannsburg, 2♀, Leonhardi (ZMB).

##### Diagnosis.

Females of *Sphex flammeus* (the male is unknown) differ from other Australian *Sphex* by the following combination of features: appressed pubescence on clypeus silvery-white, scape, legs and metasoma largely orange, and wing membrane without a yellow tinge. *Sphex decoratus*, *Sphex sericeus*,﻿ *Sphex darwiniensis* and *Sphex rhodosoma* are also largely orange, but they are members of different species groups. *Sphex staudingeri* differs in having golden pubescence on clypeus and scutum instead of silvery-white one and moderately fuscous wings instead of hyaline ones, while also being considerably larger.

##### Description.

*Female*: Body length 19.6–20.2 mm. Body orange, but the following are black: mandible and claws distally, head excluding clypeus, antenna from the pedicel onward, scutum, sometimes scutellum and metanotum. Wing membrane hyaline, forewing with faint fuscous band at apex. Wing veins range from orange to dark brown. Forebasitarsal rake with 11 long spines. Free clypeal margin straight. Appressed pubescence and erect setae on clypeus and frons silvery-white. Clypeus with medial glabrous stripe. Distance between hind-ocelli 0.9× their shortest distance to compound eyes. Pubescence on mesosoma partly golden, partly silvery, on scutum denser laterally and posteriorly. Scutellum convex, with distinct medial impression. Pubescence on propodeal enclosure short; mostly concealing sculpture. Length of petiole approximately 1.2× length of flagellomere II. Tomentum moderately dense on metasomal tergum I, sparse on tergum II.

*Male*: Unknown.

**Figure 34.**
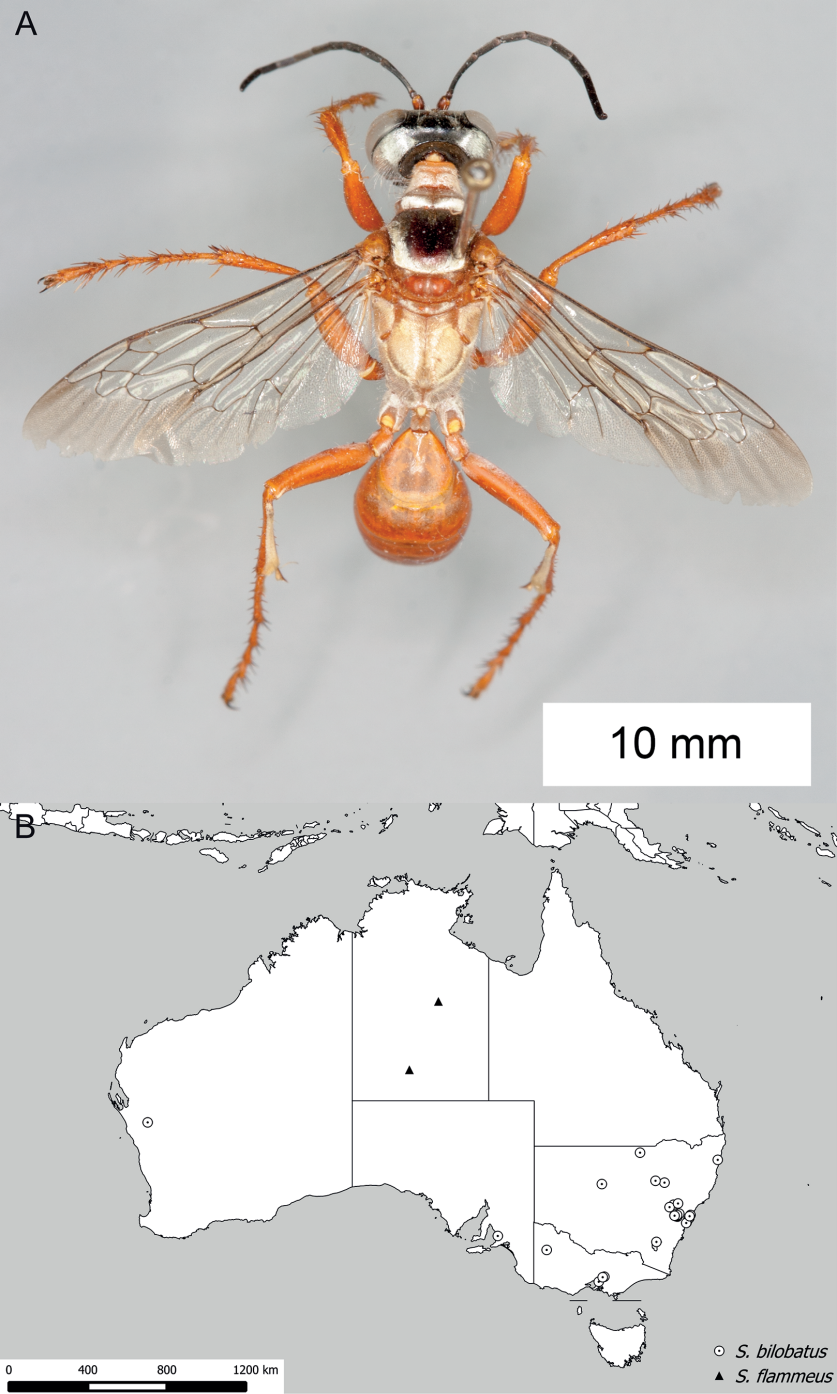
*Sphex flammeus*. **A** ♀, habitus **B** collecting localities, those of *Sphex bilobatus* are also shown.

##### Variation.

One of the three examined specimens has a median impression on the metanotum; this trait is also distinctive for species of the *Sphex argentatus* group. However, it is there accompanied by a conspicuous raising of the metanotum, which *Sphex flammeus* lacks. Therefore, this species is tentatively placed in the *Sphex subtruncatus* group.

##### Discussion.

The character combination of the available females makes it clear that this species has not yet been described. Based on species group membership, there are eight species of which females are yet unknown or where matching of males and females was first proposed in this study. *Sphex flammeus* can theoretically be the female of one of them. However, only one of them, *Sphex semifossulatus*, is also colored bright orange. Still, both differ in the color of scapes and propodeum, as well as in the orientation of the mesosomal pubescence, which is denser and more appressed in *Sphex flammeus*.

##### Etymology.

*Flammeus* is a Latin adjective, meaning “flame-colored”. It refers to the color of this species.

#### Sphex formosellus

Taxon classificationAnimaliaHymenopteraSphecidae

van der Vecht, 1957

Sphex formosellus van der Vecht, 1957: 366, ♀, ♂. Holotype: ♀, Indonesia: Timor: no specific locality (RMNH). Not examined.

##### Material examined.

**[COUNTRY UNKNOWN]:** **[state unknown]:** [no specific locality], 2♀, 1♂ (ZMB). **AUSTRALIA:** **NSW:** Bumberry, 1♀, 30.12.1976, G. Daniels (AMS); Clarence, Blue Mountains, 1♀, 16.01.1983, N. W. Rodd (AMS); Mount Tomah, Blue Mountains, 1♂, 06.04.1982, N. W. Rodd (AMS); **NT:** Edith Falls, 38 km NNW Katherine, 14°1'1"S, 132°03'6"E, 1♂, 16.04.2008, G. Williams & W. Pulawski (AMS); **QLD:** Moorooka, 2♀, 3♂, Feb–Mar 44, E. F. Riek (ANIC); **VIC:** Gunbower, 1♂, 03.03.1933 (BMNH); Oakleigh, 1♀, 11.04.1917, C. French, junr. (ANIC); **WA:** Bunbury, 2♂, 01.01.1957, A. Snell (AMS), 1♂, 03.01.1957, A. Snell (AMS), 1♂, 09.01.1957, A. Snell (AMS), 1♂, 01.01.1961, A. Snell (AMS), 2♀, 10.–22.12.1958, A. Snell (AMS). **INDONESIA:** **East Nusa Tenggara:** Timor, 1♂ (ZMB).

##### Diagnosis.

*Sphex formosellus* differs from other members of the *Sphex subtruncatus* group in combining the following characters: the clypeus is entirely covered with dense pubescence, the pubescence on the propodeal enclosure is sparse enough to leave the sculpture visible, and the legs are black. In contrast, the legs of *Sphex staudingeri* and at least the foretibia of *Sphex caelebs* are bright orange.

##### Description.

Body length 18.2–21.0 mm. Body black. Wing membrane with slight fuscous band at apex. Appressed pubescence and erect setae on clypeus and frons golden. Clypeus entirely pubescent. Pubescence on scutum longer and denser laterally. Scutellum convex. Propodeal enclosure with sparse pubescence, sculpture clearly visible. Length of petiole twice length of flagellomere II.

*Female*: Membrane of cellular wing area yellow, remainder hyaline. Wing veins yellowish-orange, darker near apex. Forebasitarsal rake with nine long spines. Free clypeal margin with two lobes medially, distance between them less than 1/8 length of flagellomere II. Distance between hind-ocelli equal to their shortest distance to compound eyes. Pubescence on collar, scutum and propodeal enclosure silvery-golden. Scutellum without impressions. Tomentum dense on metasomal terga I and II.

*Male*: Wing membrane hyaline. Wing veins light brown. Free clypeal margin truncate. Distance between hind-ocelli 1.3× their shortest distance to compound eyes. Pubescence on collar, scutum and propodeal enclosure silvery-white. Scutellum with medial impression. Tomentum very dense on metasomal tergum I and II. Metasomal terga V and VI covered with silvery-golden bristles. Metasomal sterna VI–VIII with fringes of silvery setae laterally, densest on sternum VII. Metasomal sternum VIII entire, its lateral margin concave.

**Figure 35.**
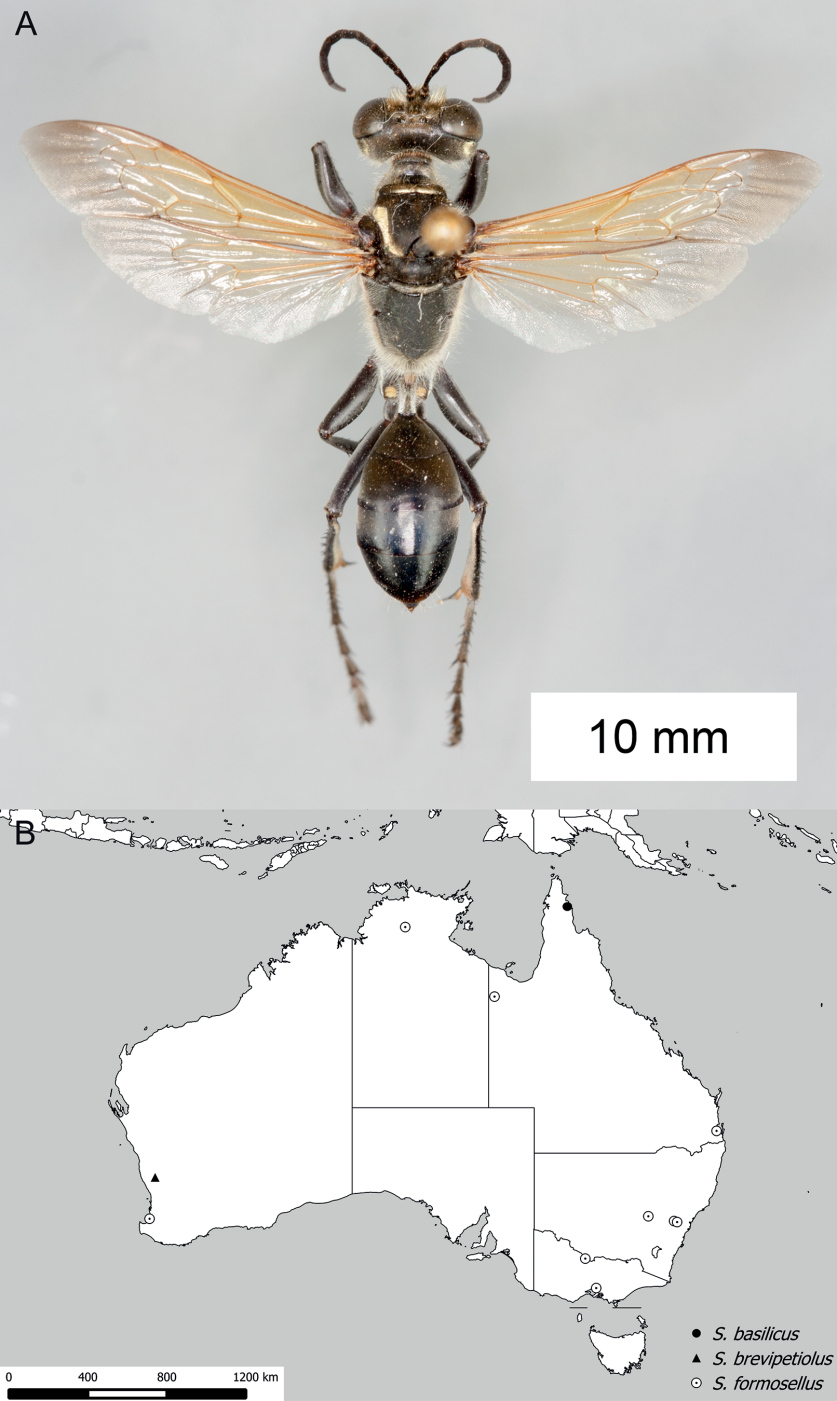
*Sphex formosellus*. **A** ♀, habitus **B** collecting localities, those of *Sphex basilicus* and *Sphex brevipetiolus* are also shown.

##### Notes on type material.

The types of *Sphex formosellus* were not examined, because the character combination for females in the original description (black body, two lobes on free clypeal margin, wing membrane hyaline with yellow tinge, clypeus and propodeal enclosure with brassy pubescence, pubescence less dense than in *Sphex cognatus*) is sufficient to unambiguously identify this species.

#### Sphex fortunatus sp. n.

Taxon classificationAnimaliaHymenopteraSphecidae

http://zoobank.org/3824764C-38ED-461F-B2B2-1AF16F650177

##### Material examined.

*Holotype*. ♂, **AUSTRALIA:** **QLD:** “North Queensland” (BMNH).

##### Diagnosis.

This species differs from other members of the *Sphex subtruncatus* group mainly in the color of the wing membrane, which is markedly fuscous near the base and around the subcosta as well as below the submedial cell. This seems to be the only character that differentiates the species from *Sphex jucundus*, which has the wing membrane hyaline in this area. The absence of tubercles on the metanotum and the lack of erect dark setae on the clypeus distinguish *Sphex fortunatus* from *Sphex finschii* in the *Sphex argentatus* group, which has similar wing coloration.

##### Description.

♀. Unknown.

*Male*: Body length 20.6 mm. Body black, mandible dark ferruginous in center, femora maroon. Wing membrane light brown, markedly fuscous around subcosta and below submedial cell. Wing veins dark brown to black, cellular area around veins on forewing fuscous. Free clypeal margin slightly concave towards center, with minute lobe there. Appressed pubescence and erect setae on clypeus and frons silvery. Clypeus glabrous ventrally and with narrow glabrous stripe medially. Distance between hind-ocelli slightly smaller than their shortest distance to compound eyes. Pubescence on mesosoma silvery-white, on scutum longer and denser laterally and posteriorly. Scutellum convex, with shallow medial impression. Pubescence on propodeal enclosure sparse, sculpture completely visible. Length of petiole 1.5× length of flagellomere II. Tomentum moderately dense on metasomal tergum I, sparse on tergum II. Metasomal terga V and VI with few bristles. Metasomal sterna II–VI mostly glabrous, VII and VIII with moderately sparse silvery pubescence. Metasomal sternum VIII entire, its lateral margin concave.

**Figure 36.**
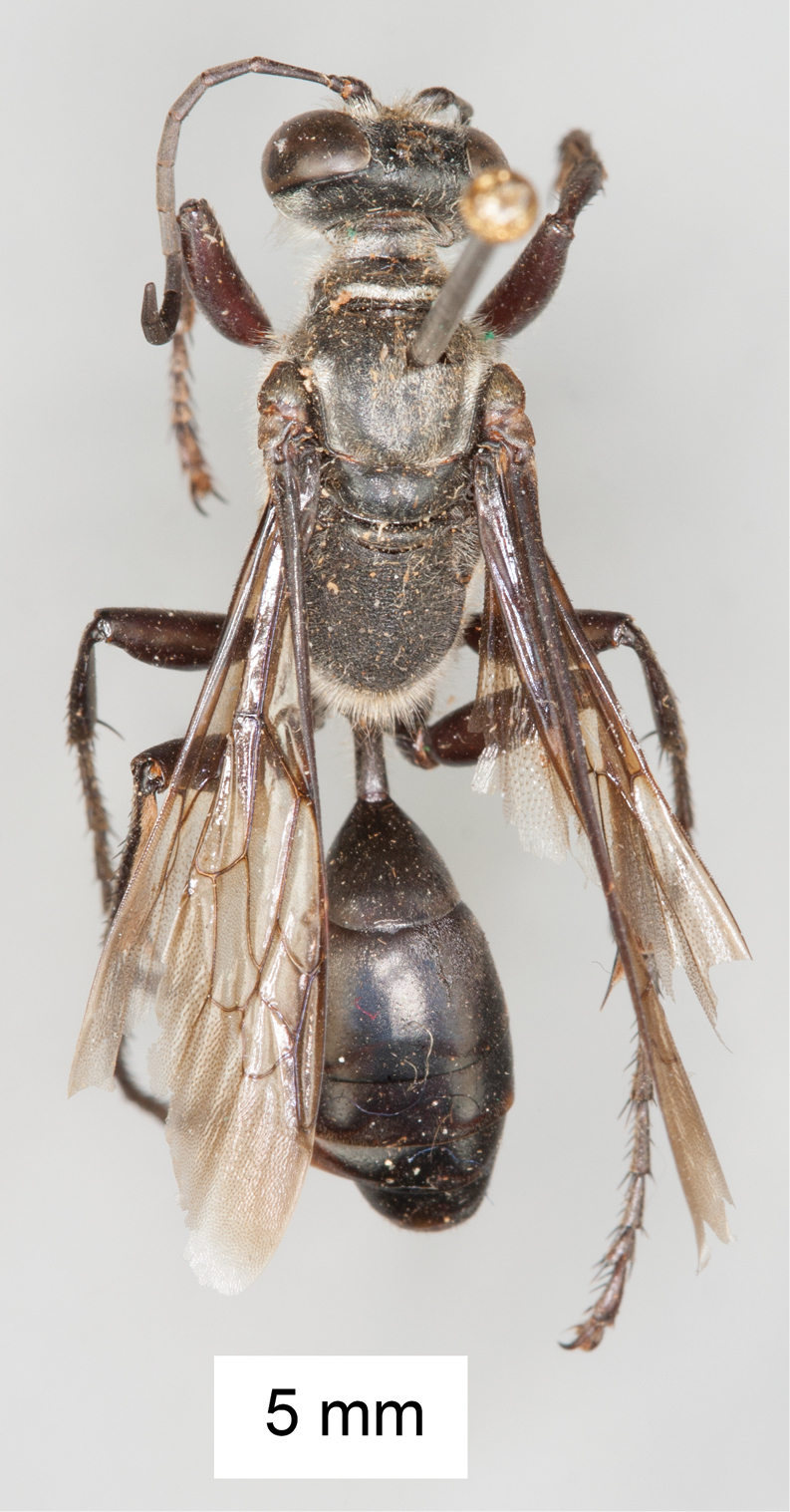
Habitus of *Sphex fortunatus*, ♂.

##### Geographic distribution.

Only one specimen of *Sphex fortunatus* could be studied, and no specific geographic information is available. Its origin is given as “North Queensland”.

##### Discussion.

There are seven species in the *Sphex subtruncatus* group of which males are yet unknown or where matching of males and females was first proposed in this study. *Sphex fortunatus* can theoretically be the male of one of them. Two of them have a petiole that is markedly shorter than flagellomere II, while two others differ in having pubescence on the propodeal enclosure that is dense enough to conceal the sculpture. One of the three remaining species (*Sphex flammeus*) is mostly orange in its body color, and another one (*Sphex pretiosus*) has a mix of golden and silvery pubescence on the propodeum and wing veins that are bright orange in the basal wing half. The last species, *Sphex jucundus*, differs, as already mentioned, in having wings that are completely hyaline except near the apex.

##### Etymology.

*Fortunatus* is a Latin adjective, meaning “happy” or “lucky”. The name was chosen in reference to *Sphex jucundus*, which is very similar in appearance.

#### Sphex jucundus sp. n.

Taxon classificationAnimaliaHymenopteraSphecidae

http://zoobank.org/9BA8B6CF-6341-4799-91B4-23A7E013E82F

##### Material examined.

*Holotype*. ♂, **AUSTRALIA:** **WA:** 6 km N of Winning HS, 23°06'S, 114°33'E, 30.03.1971, E. F. Riek (ANIC). *Paratypes*. **[COUNTRY UNKNOWN]:** **[state unknown]:** [no specific locality], 1♀ (ZMB). **AUSTRALIA:** **NSW:** Bourke, 1♀, 03.01.1954, K. M. Moore (AMS); Broken Hill, 1♂, 09.03.2001, M. Ohl (ZMB); 10 km N of Broken Hill, 2♀, 3♂, 11.03.2001, M. Ohl (ZMB), 4♀, 10♂, 12.03.2001, M. Ohl (ZMB), 1♀, 1♂, 13.03.2001, M. Ohl (ZMB); **NT:** Alexandria, Nicholson, 2♂, W. Stalker (BMNH); Port Darwin, 1♀, 01.02.1902 (BMNH), 2♂ (BMNH); **QLD:** Camooweal, 1♂, 18.05.1972, G. B. & S. R. Monteith (ANIC); 45 km S of Collinsville, 1♀, 16.01.1987, M. S. & B. J. Moulds (AMS); Westwood, 1♀, 25.11.1923, A. N. Burns (ANIC); **SA:** Adelaide, 1♂ (ZMB); Clements Gap Conservation Park, 33°28.7'S, 138°03.9'E, 2♂, 18.12.2010, V. Ahrens & W. J. Pulawski (CAS); Cocata Conservation Park, 33°17.0'S, 135°19.7'E, 1♂, 03.01.2011, V. Ahrens & W. J. Pulawski (CAS); 55 km ESE of Kimba, on Kimba/Iron Knob road, 1♂, 12.12.1995, M. S. & B. J. Moulds & K. A. Kopestonsky (AMS); Lake Gilles Conservation Park, 1♀, 01.02.1995, L. Packer, M. Schwarz, P. Hurst, Y. Pamula (ZMB); **WA:** Bullsbrook, 1♂, 13.02.1966, O. W. Richards (BMNH); Bunbury, 2♂, 03.01.1957, A. Snell (AMS), 1♂, 01.01.1961, A. Snell (AMS); Deep Dene, Karridale, 1♂, 19.01.1965, L. M. O’Halloran (ANIC); Kalamunda, 1♀, 09.–28.02.1914, R. E. Turner (BMNH), 1♀, 14.03.–14.04.1914, R. E. Turner (BMNH); 40 km SE Kalbarri, 27°50.9'S, 114°28.5'E, 1♀, 05.11.2008, V. Ahrens & W. J. Pulawski (CAS); Marloo Station, 1♀, 01.01.1935, Gebr. Goerling (ZMB), 1♀, 1♂, 01.02.1935, Gebr. Goerling (ZMB), 1♀, 01.01.1936, A. Goerling (ZMB), 1♀, Dec 34, Gebr. Goerling (ZMB); Meekatharra, 1♂, 18.01.1961, A. Snell (AMS); 55 km S of Newman, 1♀, 1♂, 08.04.1971, E. F. Riek (ANIC); Perth, 1♂, 10.–18.02.1936, R. E. Turner (BMNH); S of Perth, 1♀, 12.01.1905, H. M. Giles (BMNH), Serpentine Falls, Darling Ranges, 1♀, 20.01.1971, G. A. Holloway (AMS); Shire of Shark Bay, Francois Peron National Park, 25.906233°, 113.526010°, 1♀, 12.01.2010, L. Breitkreuz (ZMB); Tuckanarra Hill, 2♂, 16.11.1961, A. Snell (AMS); Urawa Nature Reserve ca 5 km N Mullewa, 28°29.6'S, 115°29.5'E, 1♂, 11.11.2008, V. Ahrens & W. J. Pulawski (CAS)

##### Diagnosis.

The combination of black legs, a black metasoma, wings hyaline at the base, exclusively silvery-white pubescence on the clypeus and a petiole considerably longer than flagellomere II separates *Sphex jucundus* from all other species in the *Sphex subtruncatus* group except *Sphex bilobatus*. In contrast to *Sphex bilobatus* which possesses a convex scutellum with a medial impression, females of *Sphex jucundus* have a flat scutellum without any impressions; while the visible part of metasomal sternum VIII in male *Sphex bilobatus* is modified into two long lobes that are even visible from above, while sternum VIII of *Sphex jucundus* is entire.

##### Description.

Body length 18.7–20.0 mm. Body black, mandible ferruginous basally. Wing membrane completely hyaline, only with slightly fuscous band at apex of forewing. Wing veins light to dark brown. Appressed pubescence and erect setae on clypeus and frons silvery-white. Clypeus with medialglabrous stripe. Pubescence on mesosoma silvery-white, on scutum denser laterally and posteriorly. Tomentum moderately dense on metasomal tergum I, sparse on tergum II.

*Female*: Free clypeal margin simple, clypeus elevated medially above margin, pubescent near margin. Distance between hind-ocelli almost equal to their shortest distance to compound eyes. Forebasitarsal rake with 12 long spines. Scutellum flat, without impressions. Pubescence on propodeal enclosure mostly concealing sculpture. Length of petiole 1.25× length of flagellomere II.

*Male*: Free clypeal margin truncate, concave towards center. Clypeus near margin glabrous. Distance between hind-ocelli 1.25× their shortest distance to compound eyes. Scutellum convex, with medial impression. Pubescence on propodeal enclosure sparse enough to leave sculpture visible. Length of petiole 1.4× length of flagellomere II. Several erect silvery setae on metasomal terga V–VII. Silvery pubescence on margin of metasomal sternum VII and on metasomal sternum VIII. Metasomal sternum VIII entire, its lateral margin concave.

**Figure 37.**
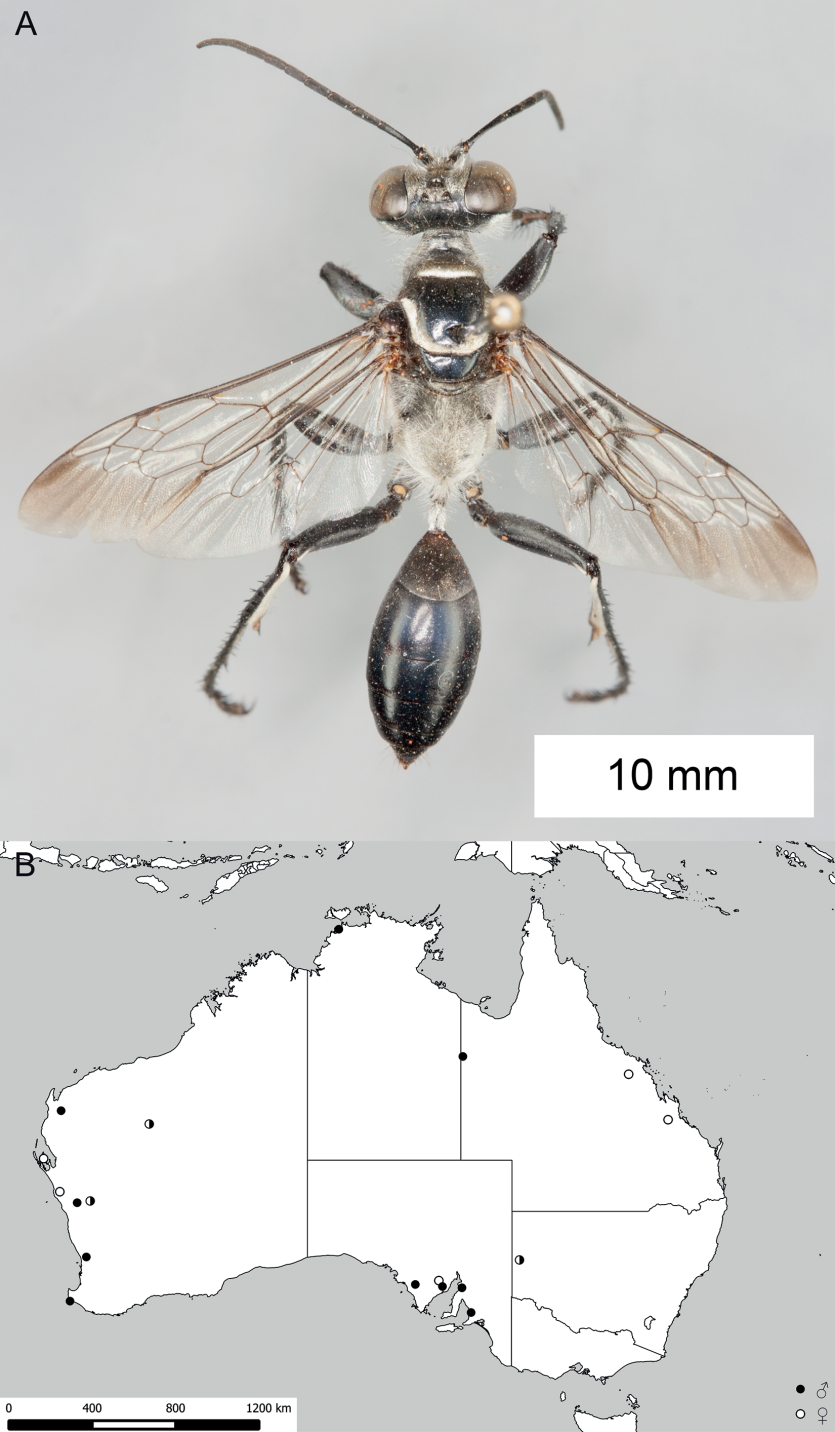
*Sphex jucundus*. **A** ♀, habitus **B** collecting localities, the combined symbol indicates that males and females were found in the same locality.

##### Discussion.

In this species, males and females closely resemble each other, except for commonly sexually dimorphic characters such as the scutellum. Due to this dimorphism, however, the females are easier to define as a new species than the males. At most, only four other females in the *Sphex subtruncatus* group are known to have a flat scutellum. Two of those are distinguished in having golden pubescence on the propodeal enclosure, and the petiole of the other two is considerably shorter than flagellomere II.

One character was found in which males and females of *Sphex jucundus* differ from each other and which is usually not a sexually dimorphic feature among Australian *Sphex*, the density of the pubescence on the propodeal enclosure. However, this argument alone would not be sufficient to demonstrate that they belong to different species. The identical coloration of the wings in males and females as well as the conspicuously dense tomentum both have on their metasomal tergum I are only a few of their shared features. Finally, a great number of males and females come from the same or from nearby localities, which is another indication that both form one species (Fig. 26).

##### Etymology.

*Jucundus* is a Latin adjective, meaning “pleasant” or “merry”. The name was apparently chosen by J. van der Vecht when he so labeled individuals of this species in the 1970s, but without describing it. The name was adopted to credit J. van der Vecht.

#### Sphex latilobus sp. n.

Taxon classificationAnimaliaHymenopteraSphecidae

http://zoobank.org/3BEDDBA6-2E7B-475A-8135-899F63A7C06F

##### Material examined.

*Holotype*. ♂, **AUSTRALIA:** **WA:** Bolgart, 14.12.1961, E. B. Britton & A. Douglas (BMNH). *Paratypes*. **AUSTRALIA:** **WA:** Bolgart, 2♂, 14.12.1961, E. B. Britton & A. Douglas (BMNH); Cervantes, 1♂, 26.12.1988, I. L. Hamer (BMNH).

The collecting localities are shown in Fig. 40C.

##### Diagnosis.

**Figure 38.**
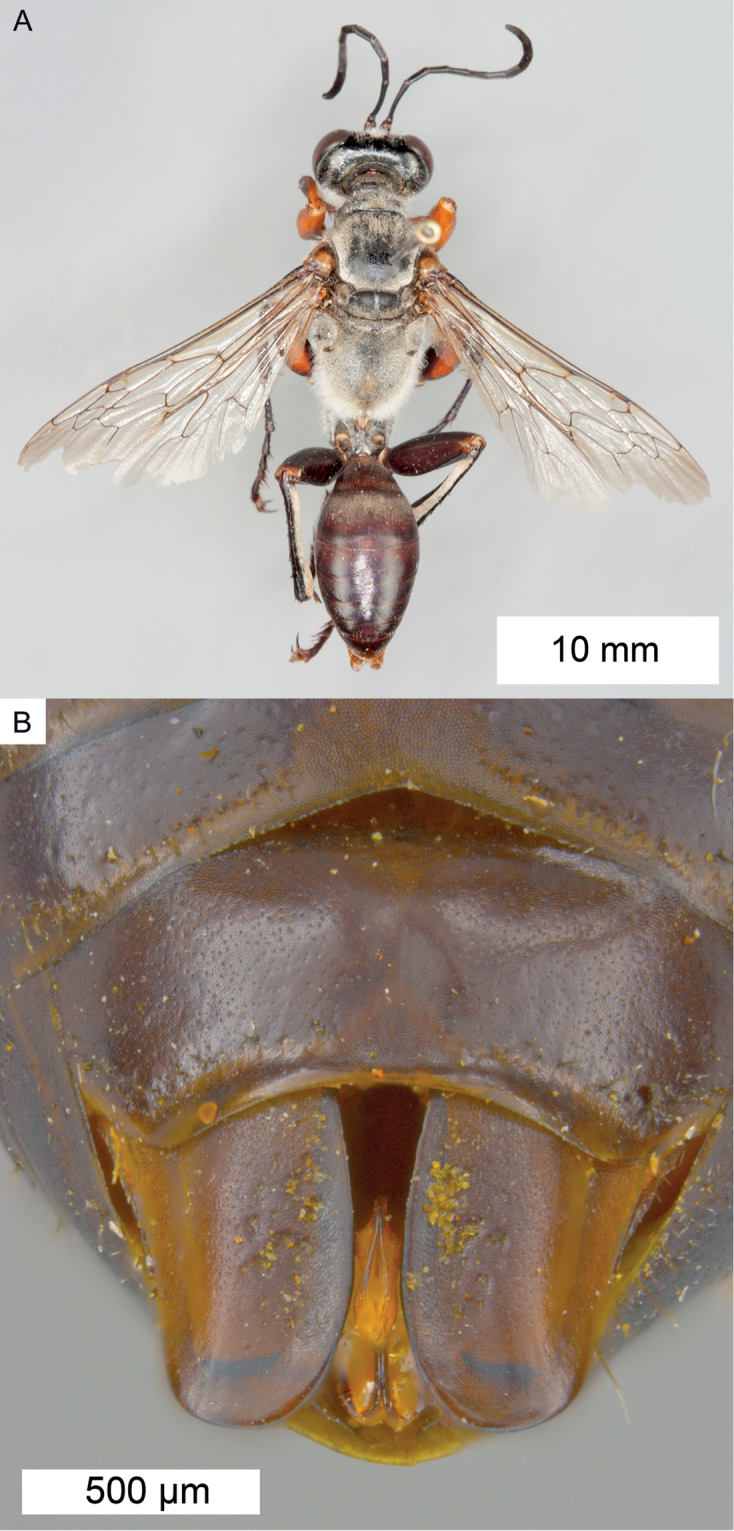
*Sphex latilobus*, ♂. **A** habitus **B** ventral view of metasomal sterna VII and VIII.

The males of this species (females are unknown) are easily identifiable by the bright orange coloration along the free clypeal margin, combined with the visible part of the metasomal sternum VIII modified into two completely separate lobes (Fig. 38B). *Sphex bilobatus* and *Sphex basilicus* possess similar structures, but the free clypeal margin is black in both species, and the latter also has golden pubescence on the propodeal enclosure, whereas the pubescens is silvery-white in *Sphex latilobus*.

##### Description.

*Female*: Unknown.

*Male*: Body length 18.8–24.5 mm. Body black, but the following are orange: mandible from base up to base of inner tooth, clypeus along free margin, fore- and at least partially midfemur, posterior half of tegula. Wing membrane almost completely hyaline, wing veins dark brown. Free clypeal margin straight or minimally convex. Appressed pubescence and erect setae on clypeus and frons silvery-white. Clypeus with medial glabrous stripe. Distance between hind-ocelli equal to or slightly smaller than their shortest distance to compound eyes. Pubescence on mesosoma silvery-white, on scutum denser laterally and posteriorly. Scutellum convex, with distinct medial impression. Pubescence on propodeal enclosure short, not completely concealing sculpture. Length of petiole 1.3× length of flagellomere II. Tomentum moderately dense on metasomal tergum I, sparse on tergum II. Metasomal terga V and VI with only few bristles. Metasomal sterna II–VIII mostly glabrous. Visible part of metasomal sternum VIII forming two large, completely separated lobes (similar to *Sphex bilobatus*, shown in Fig. 28B; but lobes of *Sphex latilobus* are wider).

##### Variation.

Of the three examined specimens, the legs of one are almost entirely black, whereas the fore- and parts of the midfemur of the other two are orange.

##### Discussion.

*Sphex latilobus* is clearly an undescribed species. It also seems unlikely that it can be associated with one of the species based solely on females. Within the *Sphex subtruncatus* group, there are seven species of which males are unknown or where matching of males and females was first proposed in this study. Since most diagnostic characters of this species are suspected to be sexually dimorphic, the number of potential candidates was first narrowed down by eliminating all species where matching of males and females is well-founded (*Sphex jucundus*, *Sphex corporosus* and *Sphex pretiosus*), which leaves four species. One of them (*Sphex brevipetiolus*) has partially orange wing veins and a petiole that barely reaches the length of flagellomere II; another one (*Sphex ahasverus*) has dark setae on the clypeus, fuscous wings and golden propodeal pubescence. Of the remaining two, one (*Sphex argentatissimus*) has a clypeus with golden pubescence; the body of the other one (*Sphex flammeus*) is mostly bright orange. Thus, *Sphex latilobus* is unlikely to match any of the currently recognized species.

##### Etymology.

*Latilobus* is a composite of the Latin words *latus* (wide) and *lobus* (elongated projection), referring to the lobes on male sternum VIII which are similar to those of *Sphex bilobatus* but wider.

#### Sphex pretiosus sp. n.

Taxon classificationAnimaliaHymenopteraSphecidae

http://zoobank.org/FEEB3667-7B42-437A-8B2D-CB80A8181CEA

##### Material examined.

*Holotype*. ♂, **AUSTRALIA:** **NSW:** 56 miles W of Cobar, Baznatos Tank, 01.01.1966, O. W. Richards (BMNH). *Paratypes*. **AUSTRALIA:** **NSW:** Binnaway, 1♀, Dec 73, A. Smith (AMS); Gilgandra, 1♀, 06.11.1987, G. A. Holloway (AMS); between Whitecliff and Wilcannia, 2♂, 09.03.2001, M. Ohl (ZMB); **SA:** 27 km WSW Whyalla, 33°06.5'S, 137°19.0'E, 1♀, 28.12.2010, V. Ahrens & W. J. Pulawski (CAS).

##### Diagnosis.

**Figure 39.**
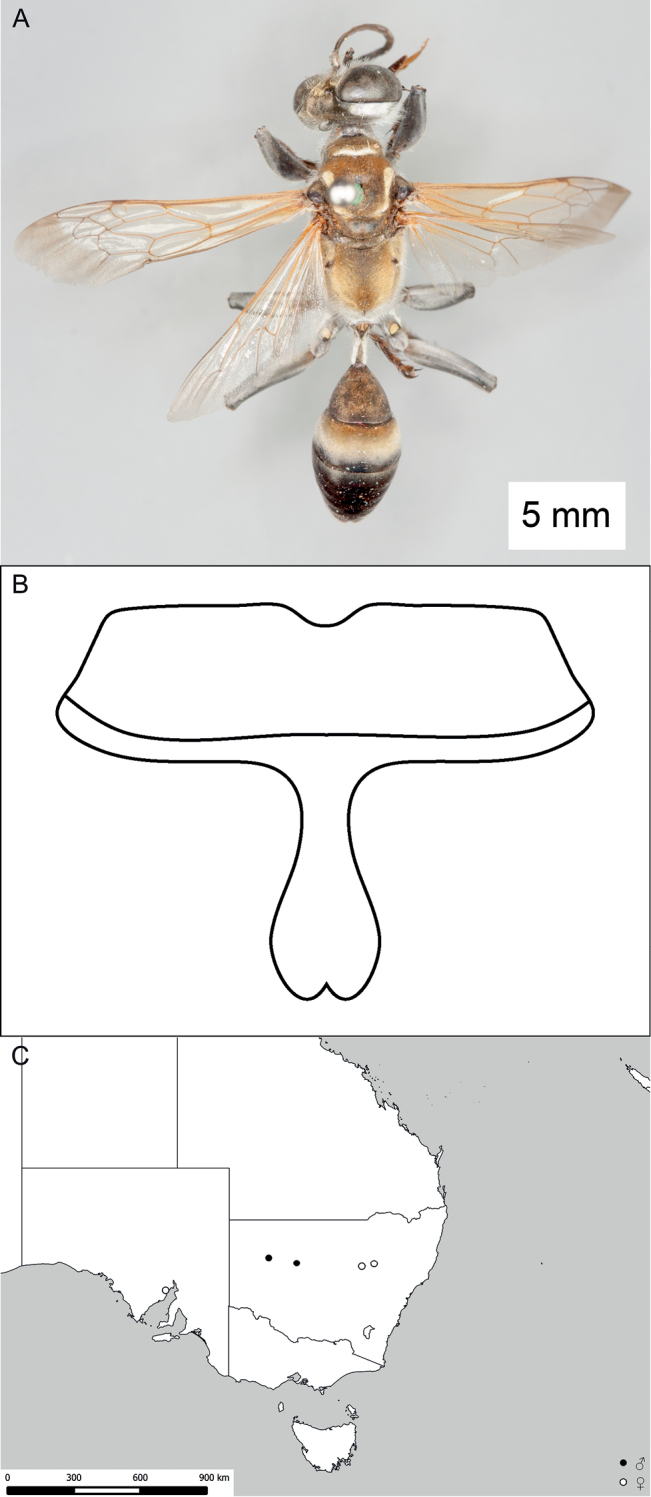
*Sphex pretiosus*. **A** ♂, habitus **B** ♂, sketch of metasomal sterna VII and VIII **C** collecting localities.

This species is unique among the Australian *Sphex* in having a more or less sharp transition in the color of the pubescence on the clypeus and the propodeal dorsum. On the ventral part of the clypeus and the outer and posterior margin of the propodeal enclosure, the pubescence is silvery-white; while it is golden on the dorsal part of the clypeus and silvery-golden to golden on the propodeal enclosure. Moreover, the metasomal sternum VIII in males is spoon-shaped and has a gentle notch at the apical margin (Fig. 39B).

##### Description.

Forewing membrane posterobasally with slight yellow tinge, with fuscous band at apex. Wing veins bright orange, brown near apex. Appressed pubescence on ventral part of clypeus silvery-white, on dorsal part and frons silvery-golden. Erect setae matching respective color of appressed pubescence on clypeus, silvery-white on frons. Clypeus almost entirely covered with pubescence. Distance between hind-ocelli 1.25× their shortest distance to compound eyes. Pubescence on scutum denser laterally and posteriorly. Scutellum convex, with medial impression. Pubescence on propodeal enclosure not completely concealing sculpture. Propodeal pubescence outside enclosure and on petiole silvery-white. Length of petiole nearly twice length of flagellomere II. Tomentum moderately dense on metasomal tergum I.

*Female*: Body length 17.7–18.8 mm. Body black except mandible reddish basally. Free clypeal margin with two lobes medially and with another less-pronounced lobe on each side of the aforementioned; distance between each two lobes less than 1/8 length of flagellomere II. Forebasitarsal rake with 12 long spines. Pubescence on collar, scutum and propodeal enclosure silvery-white to silvery-golden. Tomentum moderately dense on metasomal tergum II.

*Male*: Body length 18.4–18.7 mm. Body black. Free clypeal margin slightly concave, convex near center. Pubescence on collar and scutum silvery-golden to golden, on propodeal enclosure golden. Tomentum very dense on metasomal tergum II. Metasomal terga V and VI with few bristles. Metasomal sterna III–VIII with silvery pubescence. Metasomal sternum VIII mostly covered by VII; with long, narrow, spoon-shaped extension that has a small notch at apical margin.

##### Variation.

In a few of the examined specimens, the metanotum is moderately raised, and in one of these, there is a slight median impression on it. A raised metanotum with an impression is usually distinctive for species of the *Sphex argentatus* group, but since the raising of the metanotum and the depth of the impression in the most extreme known cases of this species are only comparable to the least extreme cases of examined specimens of the *Sphex argentatus* group, *Sphex pretiosus* is tentatively placed in the *Sphex subtruncatus* group.

##### Discussion.

Males and females of *Sphex pretiosus* have nearly identical features that are also unique among the Australian species, such as the transition between golden and silvery-white pubescence on the clypeus and the propodeal dorsum. Also, the conspicuously modified sternum VIII of the male substantiates a full species status. Fig. 39C shows that the proposed males and females at least occur in the same region.

##### Etymology.

*Pretiosus* is a Latin adjective, meaning “valuable” or “costly”. It metaphorically refers to the combination of golden and silvery pubescence on the mesosoma of this species.

#### Sphex semifossulatus

Taxon classificationAnimaliaHymenopteraSphecidae

van der Vecht, 1973

Sphex argentifrons F. Smith, 1868: 248, ♀, actually ♂ (see van der Vecht 1973: 349), junior primary homonym of *Sphex argentifrons* Lepeletier de Saint Fargeau, 1845. Holotype or syntypes: ♀, Australia: Western Australia: Champion Bay, now Geraldton (BMNH). Presumed holotype examined.
Sphex semifossulatus van der Vecht, 1973: 349. Substitute name for *Sphex argentifrons* F. Smith.

##### Material examined.

*Holotype* (presumed). ♂, **AUSTRALIA:** **WA:** Champion Bay, now Geraldton (BMNH).

##### Diagnosis.

**Figure 40.**
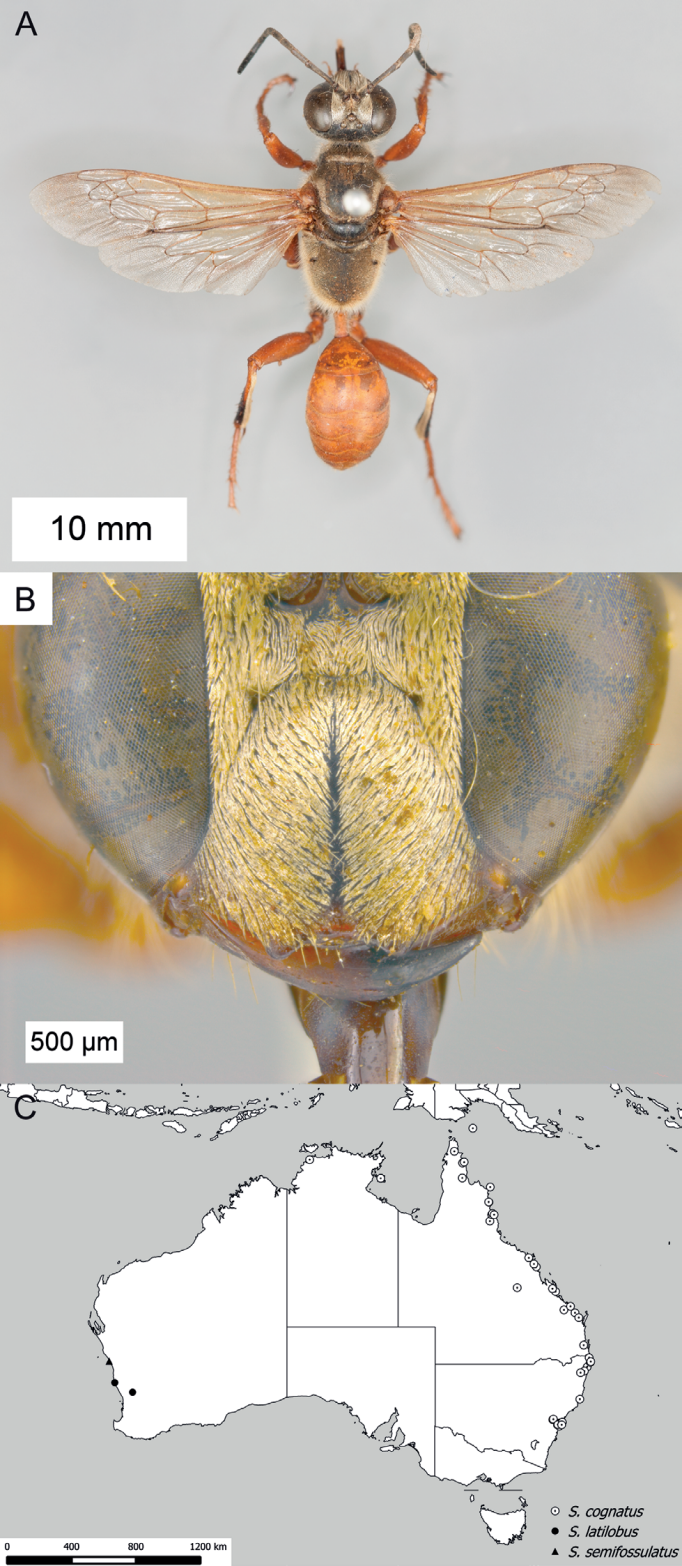
*Sphex semifossulatus*. **A** ♂, habitus **B** ♂, frontal view of clypeus **C** collecting localities, those of *Sphex cognatus* and *Sphex latilobus* are also shown.

Males of *Sphex semifossulatus* (females are unknown) are unique among the Australian *Sphex* in having two lobes on the free clypeal margin that are conspicuously far apart (Fig. 40B). The distance between them is equal to almost half the length of flagellomere II. Of the other examined species, only the males of *Sphex cognatus* also have two lobes on the clypeus, but those are merely separated by a small notch.

##### Description.

*Female*: Unknown.

*Male*: Body length 23.2 mm. Body black, but the following are orange: base of mandible, center of free clypeal margin, tegula, metasoma, legs excluding coxae and distal part of hindtibia as well as claw teeth and distal half of claw. Forewing membrane with yellow tinge near base and slightly fuscous band at apex, hindwing membrane hyaline. Wing veins orange, darker near apex. Free clypeal margin with two lobes, distance between them more than half length of flagellomere II. Appressed pubescence and erect setae on clypeus and frons silvery-white. Clypeus with narrow medial glabrous stripe. Distance between hind-ocelli 1.2× their shortest distance to compound eyes. Pubescence on collar and scutum silvery-golden, on the latter denser laterally and posteriorly. Scutellum convex, with distinct medial impression. Propodeal enclosure with silvery-golden pubescence, leaving sculpture mostly visible. Length of petiole 1.25× length of flagellomere II. Tomentum dense on metasomal tergum I and II. Metasomal terga V and VI covered with silvery bristles. Metasomal sterna II–VII each with increasing amount of erect silvery setae laterally. Metasomal sternum VIII with few erect silvery setae, notched apically, its lateral margin straight, convex posteriorly.

#### Sphex staudingeri

Taxon classificationAnimaliaHymenopteraSphecidae

Gribodo, 1894

Sphex staudingeri Gribodo, 1894: 3, ♂ (as *Staudingeri*, incorrect original capitalization). Holotype or syntypes: ♂, New Guinea: no specific locality (Genova). Presumed holotype examined.

##### Material examined.

*Holotype* (presumed). ♂, **[COUNTRY UNKNOWN]:** **[province unknown]:** New Guinea [no specific locality] (MSNG).

##### Other material.

**[COUNTRY UNKNOWN]:** **[province unknown]:** New Guinea [no specific locality], 1♀ (ZMB), 1♂ (NHMW).

##### Diagnosis.

*Sphex staudingeri* is unique among the Australian *Sphex* in combining orange scapes, golden propodeal pubescence and the absence of tubercles on the metanotum. *Sphex basilicus* differs in having black scapes and a black metasoma, whereas the metasoma of *Sphex staudingeri* is orange, although sometimes in a very dark tone.

##### Description.

Wing veins brown. Appressed pubescence and erect setae on clypeus and frons golden. Pubescence on mesosoma golden, on scutum denser laterally. Scutellum convex, with shallow medial impression. Propodeal enclosure with sparse, erect, golden pubescence; sculpture completely visible. Length of petiole approximately 1.25× length of flagellomere II. Tomentum dense on metasomal tergum I.

*Female*: Body length 29.0 mm. Body black, but the following are orange: mandible proximally, mouthparts, clypeus, scape, pedicel, scutellum, metanotum, metasoma, legs excluding coxa and proximal part of trochanter as well as claw teeth and distal half of claw. Wing membrane uniformly light brown. Forebasitarsal rake with nine long spines. Free clypeal margin with indistinct emarginations. Clypeus medioventrally with glabrous spot. Distance between hind-ocelli 0.8× their shortest distance to compound eyes. Tomentum moderately dense on metasomal tergum II.

*Male*: Body length 27.2–28.2 mm. Body black, but the following are orange: mandible proximally, mouthparts, metasomal segment I at least partially, legs excluding coxa and trochanter as well as claw teeth and distal half of claw. Wing membrane with yellow tinge in cellular area and with slightly fuscous border at apex. Free clypeal margin truncate, concave towards center. Clypeus with narrow medial glabrous stripe. Distance between hind-ocelli slightly smaller than their shortest distance to compound eyes. Tomentum dense on metasomal tergum II. Metasomal terga V and VI with few golden bristles. Metasomal sterna II–IV mostly glabrous, V–VIII with few reddish-golden bristles laterally. Metasomal sternum VIII entire, slightly arched towards ventral side, truncate at apical margin, its lateral margin straight.

**Figure 41.**
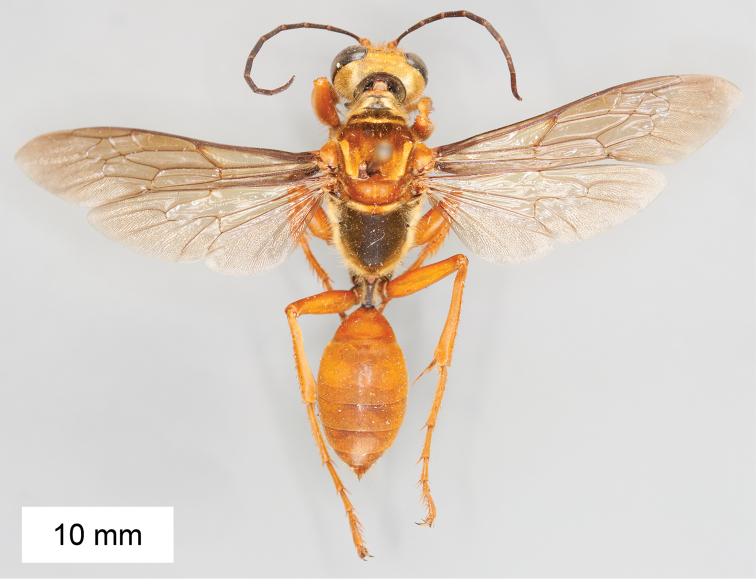
Habitus of *Sphex staudingeri*, ♀.

##### Geographical distribution.

Although this species is listed in the catalog of Australian Sphecidae (Cardale 1985), no indication of specimens actually collected within Australia was found; all examined individuals of *Sphex staudingeri* come from New Guinea, which is also the only country recorded by Hensen (1991). Berland (1928) records the species from Port Jackson, now part of Sydney, with reference to Dumont d’Urville as collector. This old record is likely to be inaccurate (see Discussion).

#### Sphex vestitus

Taxon classificationAnimaliaHymenopteraSphecidae

F. Smith, 1856

Sphex vestitus F. Smith, 1856: 248, ♀ (as *vestita*, incorrect original termination). Holotype or syntypes: ♀, Australia: no specific locality (BMNH). Not examined.

##### Material examined.

**AUSTRALIA:** **NSW:** 6 km NE of Bilpin, Blue Mountains, 1♀, 26.03.1982, N. W. Rodd (AMS), 1♀, 27.02.1983, N. W. Rodd (AMS); Brisbane Water National Park, Warrah Trig, 1♀, 15.02.1986, D. B. McCorquodale (ANIC); 13 km N of Coffs Harbour, 1♀, 05.01.1978, G. Daniels (AMS); Fairlight Road, Mulgoa, 1♀, 26.02.1985, C. A. P. Urquhart (AMS); Pearl Beach, 1♂, 01.01.1985, C. A. P. Urquhart (AMS); Sandy Creek W of Ebor, 1♂, 03.01.1978, G. Daniels (AMS); Sydney, 2♀, C. Gibbons (AMS); The Needles, Woronora River, Engadine, 1♀, 27.01.1979, R. Eastwood (AMS); Woy Woy, 1♀, 1♂, 08.05.1924, Nicholson (AMS); **QLD:** “North Queensland”, 1♂ (BMNH); N Bundaberg, 1♀, 1♂, 21.03.1972, H. Frauca (ANIC); Byfield State Forest, 1♀, 01.01.1976, G. Daniels (AMS); Claudie River, 4 miles W of Mount Lamond, 1♂, 24.12.1971, D. K. McAlpine, G. A. Holloway, D. P. Sands (AMS), 1♂, 12.01.1972, D. K. McAlpine & G. A. Holloway (AMS), 1♂, 13.01.1972, D. K. McAlpine & G. A. Holloway (AMS); Cockatoo Creek Xing, 17 km NW Heathlands, 11°39'S, 142°27'E, 1♀, 25.04.–07.06.1992, T. McLeod (ANIC); Division of Dawson, 1♀, Rothschild & Bequest (BMNH); Kamerunga near Cairns, 1♀, 27.12.1974, M. S. & B. J. Moulds (AMS); Kuranda, 1♂, 05.03.1950, A. N. Burns (ANIC); Lockerbie Scrub, Cape York, 1♀, 12.04.1975, M. S. Moulds (AMS); Moorooka, 1♀, 10.02.1945, E. F. Riek (ANIC); 9 miles W of Paluma, 1♂, 16.04.1969, I. F. B. Common & M. S. Upton (ANIC); Pinock River, Hogback range, WSW of Bundaberg via Gin Gin, 1♀, 11.03.1972, H. Frauca (ANIC).

##### Diagnosis.

**Figure 42.**
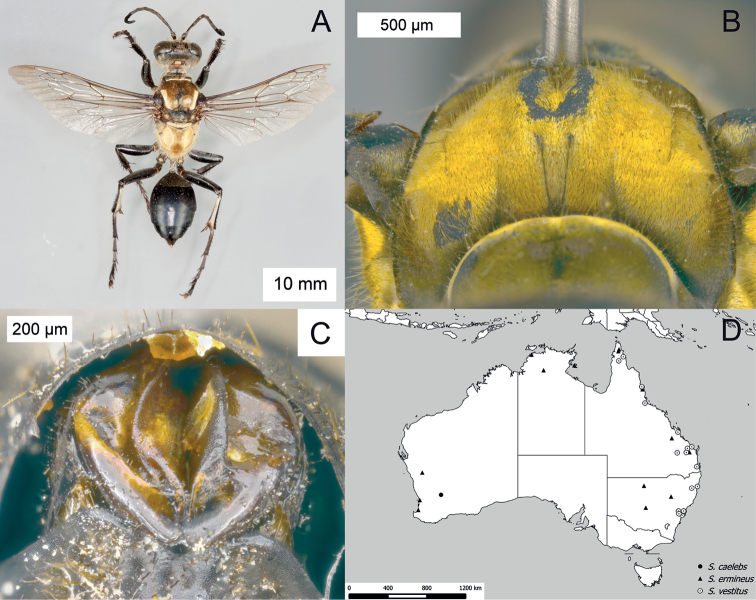
*Sphex vestitus*. **A** ♀, habitus **B** anterodorsal view of collar and scutum **C** ventral view of metasomal sternum VIII **D** collecting localities, those of *Sphex caelebs* and *Sphex ermineus* are also shown.

Females of *Sphex vestitus* can be identified by the pubescence on the clypeus and the propodeal enclosure being golden combined with the wing membrane hyaline, without yellow tinge. A unique character differentiating this species from similar ones (like *Sphex cognatus*) is a longitudinal median impression on the posterior half of the scutum (Fig. 42B). Males of *Sphex vestitus* can be identified by two longitudinal carinae on the metasomal sternum VIII which form a channel-like structure (Fig. 42C), and also by a single lobe in the center of the free clypeal margin.

##### Description.

Body black. Wing membrane hyaline, forewing with slight fuscous band at apex and light brown spot beyond marginal cell. Wing veins dark brown to black. Clypeus medioventrally with inconspicuous indentation. Appressed pubescence and erect setae on clypeus and frons golden. Pubescence on mesosoma golden, on scutum longer and denser laterally. Posterior half of scutum with longitudinal median impression. Scutellum convex, with medial impression. Pubescence on propodeal enclosure dense, completely concealing sculpture. Tomentum very dense on metasomal sternum I, dense on sternum II.

*Female*: Body length 26.3–30.0 mm. Forebasitarsal rake with ten long spines. Free clypeal margin with two small lobes medially, distance between them less than 1/8 length of flagellomere II. Clypeus medioventrally with triangular glabrous area. Distance between hind-ocelli almost equal to their shortest distance to compound eyes. Length of petiole 1.4× length of flagellomere II.

*Male*: Body length 23.0–26.6 mm. Free clypeal margin broadly emarginated, with a broad triangular median lobe. Clypeus glabrous directly above margin. Distance between hind-ocelli 1.1× their shortest distance to compound eyes. Length of petiole 1.5× length of flagellomere II. Metasomal terga V and VI with few golden bristles. Metasomal sterna II–VIII with increasing number of erect golden setae laterally. Metasomal sternum VIII with two markedly raised longitudinal carinae forming a channel-like structure, and with distinct notch at apical margin. Lateral margin of metasomal sternum VIII straight.

##### Notes on type material.

The types of *Sphex vestitus* and its synonyms were not examined, because the character combination in the original description (black body, uniformly golden pubescence on clypeus and mesosoma, hyaline wing membrane) is sufficient to unambiguously identify this species.

## Discussion

In the following paragraphs, a few of the problematic species whose geographic distribution is partially inconclusive are addressed, and relevant characters are discussed.

As mentioned earlier, no specimens of *Sphex finschii* from Australia were found among the studied material, but the notes on geographic distribution by Hensen (1991) imply that he has seen some.

For *Sphex staudingeri*, the situation is a bit more ambiguous. No Australian specimens could be examined, but Berland (1928) lists a specimen of *Sphex staudingeri* from Port Jackson (now part of Sydney). The species has a rather conspicuous appearance, so misidentification is quite unlikely. On the other hand, no specimens of *Sphex staudingeri* from Australia other than that listed by Berland are known, which means that this locality record, also given its age, is probably incorrect.

Almost nothing is known about *Sphex australis* (Gmelin, 1790). The type was destroyed in the past (Pulawski 2014), and the original description is short and vague. This species has not been mentioned in any other publication besides the initial description, and it seems likely that it is a synonym of another species, possibly even in a different genus (Pulawski 2014). Thus, *Sphex australis* was ignored in this work.

The geographic distribution of *Sphex habenus* (Say, 1832) is also not entirely clear. Kohl (1890) described *Sphex princeps*, which was synonymized with *Sphex habenus* (Fernald, 1931). *Sphex habenus* is found in the southern part of the USA and in Mexico, and localities outside of America are not known. However, Kohl stated the locality of the type specimen of *Sphex princeps* as “country unknown, probably Australia”. Based on the available information, his assumption is probably incorrect, since *Sphex habenus* was neither found by Hensen (1991) nor among the specimens that were examined in this study.

Based on Pulawski (2014), there are 118 described species of *Sphex* worldwide, and 37 of these can be found in the overall region of Malesia, the Bismarck Archipelago
(BA), the Solomon Islands
(SI), and Australia. Their currently known distribution is as follows:

§ 23 species found in Australia

§ 16 of them endemic

§ 21 species found in Malesia/BA/SI

§ 14 of them not yet detected in Australia

§ 7 species with known localities in Australia as well as Malesia/BA/SI

Taking into consideration the species that were newly discovered in the course of this study, the number of Australian species rises from 23 to 34. Only one of the newly described species, *Sphex corporosus*, is also known from outside of Australia, and this is supported by merely one specimen. The reason almost no new Malesian species were discovered here is probably the fairly small amount of material that was collected from the area; the region was also covered by Hensen (1991).

**Figure 43.**
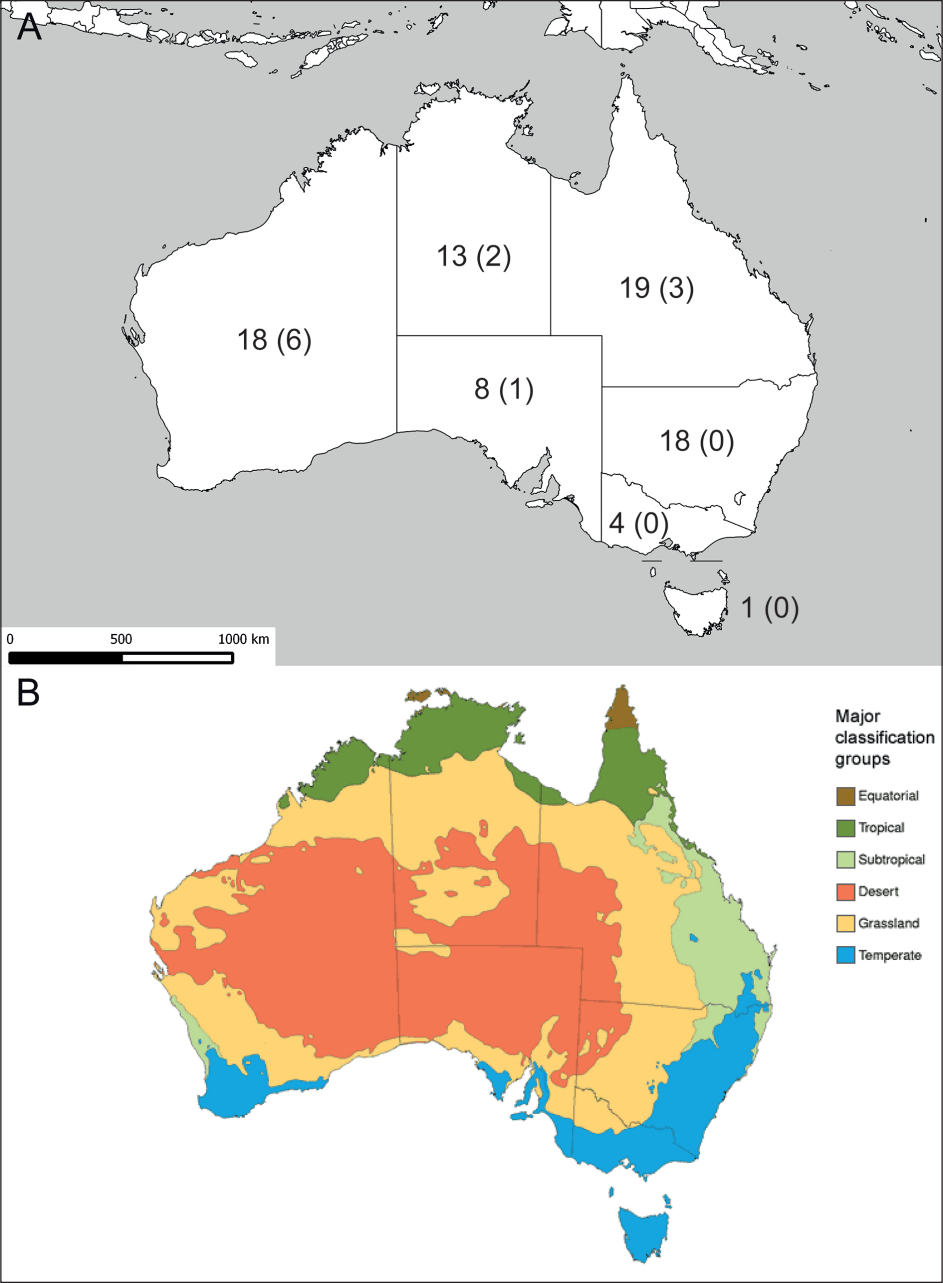
Species diversity compared to climate zones. **A** the numbers indicate how many different *Sphex* species were found in each of the Australian federal states (ACT not included); numbers in brackets depict how many of these are endemic to the state **B** Australian key climate groups based on a modified Köppen classification system; image source: Australian Government Bureau of Meteorology (Commonwealth of Australia 2014).

Using the gathered locality data of each species of *Sphex*, diversity and geographic distribution among countries and federal states was assessed at a more general level. Fig. 43A depicts this for Australia. As shown, Queensland is the state with the largest number of different *Sphex* species (19), while Western Australia is the one with the most endemic ones (6). New South Wales, while on par with Western Australia in its total number of species, completely lacks endemics. A possible explanation for this can be found in the Australian climate zones (Fig. 43B). Assuming that the deserts function more or less as a barrier against dispersal and hybridization, *Sphex* species from Western Australia (all of which are only known from the western half of the state) are much more geographically separated than those of the other states. On the other hand, New South Wales contains only a rather small desert area and shares all of its climate zones with at least one of the neighboring states, so species dispersal among these states is less constrained.

Even though it might be tempting, we do not feel qualified to estimate the number of still undiscovered *Sphex* species. There are many papers which attempt to assess global or local species richness based on the examination of a single taxon, and the resulting numbers are vastly different and likely unreliable (Erwin 1982; Hodkinson and Casson 1991; Colwell and Coddington 1994). Even when ignoring this fact, the Australian continent as a whole is much too heterogeneous to be used as a reference site, and on a smaller as well as on a greater scale, the sample size in this study is not large enough for a reasonable estimate.

Not all characters that are known to be useful for species delimitation in *Sphex* were utilized in this study. Since the main aim was to generate a key that is easy to use, does not involve inflicting damage on the individual, and also works on dried and older specimens, the use of genetic characters or that of features which require dissection of the specimen to be studied was ruled out from the start. Also, some morphological characters were not included for different reasons. For example, some traits seem useful for species discrimination at first glance, but have a high intraspecific variability. Such are the presence of an impression on the collar, the presence of a pale spot on the underside of the scape, and the color of compound eyes and ocelli. In these instances, it was found that the variation was independent of the specimens’ physical condition and preservation status.

A few characters that were mentioned by authors in the past have been omitted in this study, since they were not found to be of particular use. For instance, Bohart and Menke (1976) stated that *Sphex darwiniensis* and *Sphex rugifer* are atypical in the narrow ventral, terminal, blade-like setae of the last hindtarsomere. However, examination of the two species did not provide any significant differences between these and their congeners.

Characters whose thorough inspection across all species was deferred for time reasons, but which will probably be valuable for subsequent studies, include (among others): length/width relation of the clypeus, placoid pattern on the male antenna, and structure of the mesosomal sculpture. The latter was occasionally used to reinforce proposed matching of male and female specimens, but actual classification and delimitation of the different patterns as well as recognizing variation is undoubtedly a challenging task.

Finally, we are aware that description of a new species based solely on a single specimen can be regarded as problematic. It has been done in this paper for *Sphex caelebs*, *Sphex brevipetiolus*, and *Sphex fortunatus*. Still, at least in the former two, more than one character differentiates them from all other Australian *Sphex*, which reduces the chances that these are merely intraspecific variations of existing species. On the other hand, *Sphex fortunatus* differs indeed only in the wing color from *Sphex jucundus*, so conspecificity of these two seems possible. Nonetheless, the presence or absence of darkening on the base and center of the wing is apparently a rather invariable character in *Sphex*. Concerning the wings of the examined material, the only features where significant intraspecific differences were observed are the color of the veins, the presence of yellow tinge, slight variations in the extent of wing area coloration, and the intensity of the fuscous band near the apex. Based on this, it was deemed reasonable to grant *Sphex fortunatus* the status of a separate species.

## Supplementary Material

XML Treatment for Sphex argentatus

XML Treatment for Sphex carbonicolor

XML Treatment for Sphex decoratus

XML Treatment for Sphex ephippium

XML Treatment for Sphex finschii

XML Treatment for Sphex modestus

XML Treatment for Sphex sericeus

XML Treatment for Sphex darwiniensis

XML Treatment for Sphex fumipennis

XML Treatment for Sphex gilberti

XML Treatment for Sphex gracilis

XML Treatment for Sphex imporcatus

XML Treatment for Sphex luctuosus

XML Treatment for Sphex mimulus

XML Treatment for Sphex resplendens

XML Treatment for Sphex rhodosoma

XML Treatment for Sphex rugifer

XML Treatment for Sphex ahasverus

XML Treatment for Sphex argentatissimus

XML Treatment for Sphex basilicus

XML Treatment for Sphex bilobatus

XML Treatment for Sphex brevipetiolus

XML Treatment for Sphex caelebs

XML Treatment for Sphex cognatus

XML Treatment for Sphex corporosus

XML Treatment for Sphex ermineus

XML Treatment for Sphex flammeus

XML Treatment for Sphex formosellus

XML Treatment for Sphex fortunatus

XML Treatment for Sphex jucundus

XML Treatment for Sphex latilobus

XML Treatment for Sphex pretiosus

XML Treatment for Sphex semifossulatus

XML Treatment for Sphex staudingeri

XML Treatment for Sphex vestitus

## Supplementary materials

Supplementary material 1 Species data for genus *Sphex* Data type: species data This dataset is made available under the Open Database License (http://opendatacommons.org/licenses/odbl/1.0/). The Open Database License (ODbL) is a license agreement intended to allow users to freely share, modify, and use this Dataset while maintaining this same freedom for others, provided that the original source and author(s) are credited.Thorleif H. Dörfel, Michael Ohl
