# Supplementary figures and images for: A revision of the Australian digger wasps in the genus Sphex (Hymenoptera, Sphecidae)
Source: Zookeys. 2015 Sep 17;(521):1–104. doi: 10.3897/zookeys.521.5995 (PMC4591716; doi:10.3897/zookeys.521.5995)

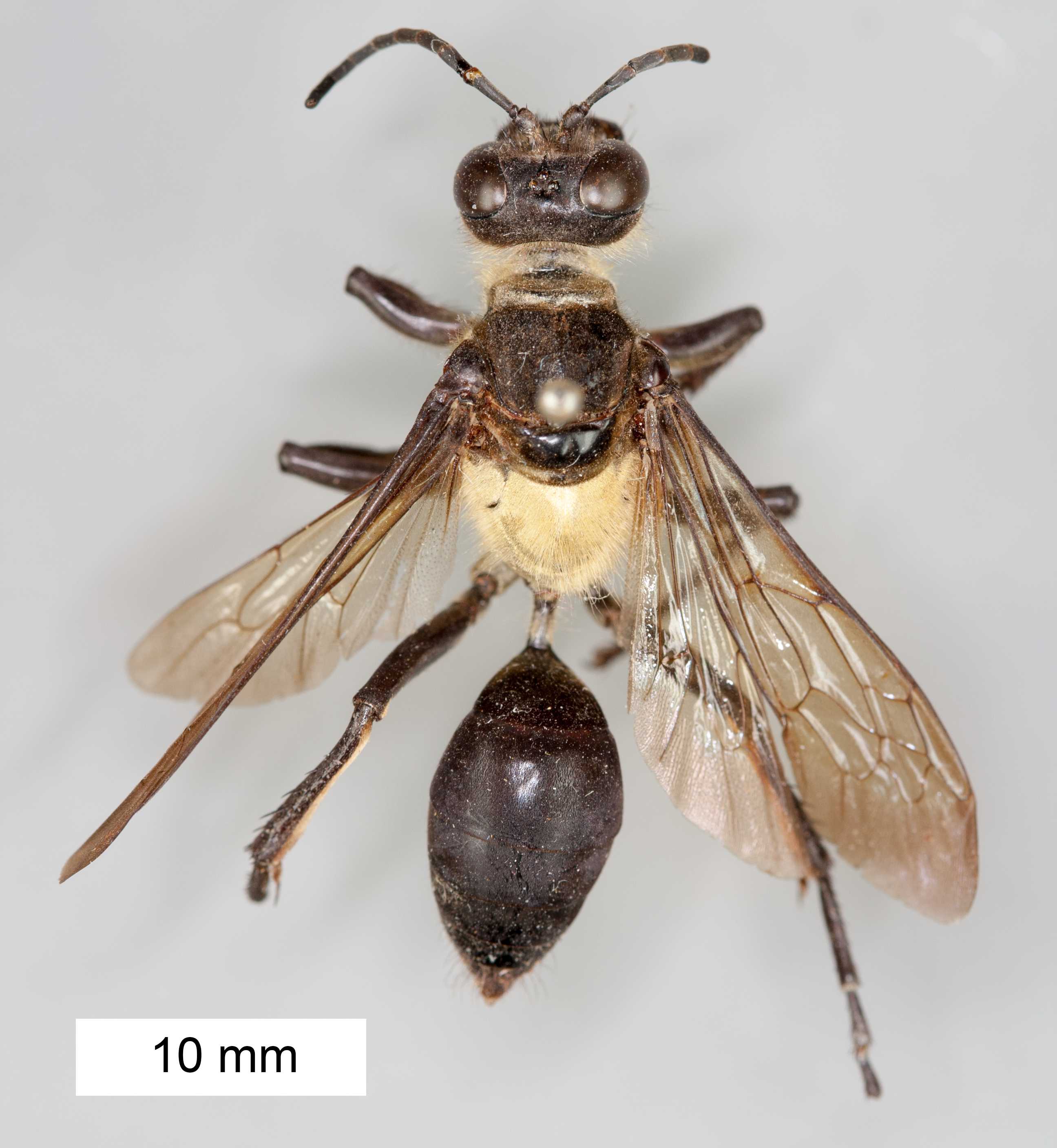

Supplement: Supplementary material 1 — Species data for genus Sphex [file zookeys-521-001-s001.zip › SphexDeltaFiles/Images/ahasverus_f.jpg]

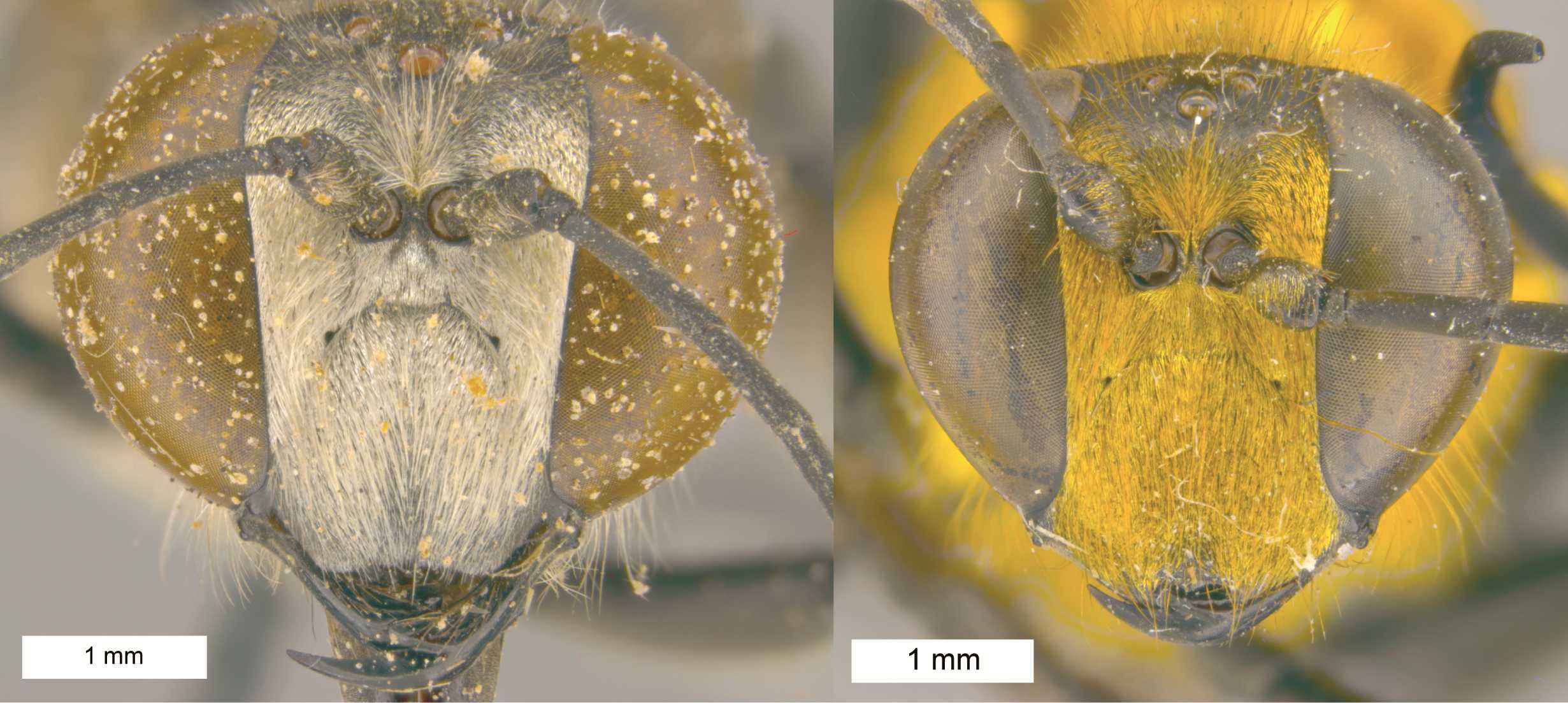

Supplement: Supplementary material 1 — Species data for genus Sphex [file zookeys-521-001-s001.zip › SphexDeltaFiles/Images/apressed_pubescence_clypeus_color.jpg]

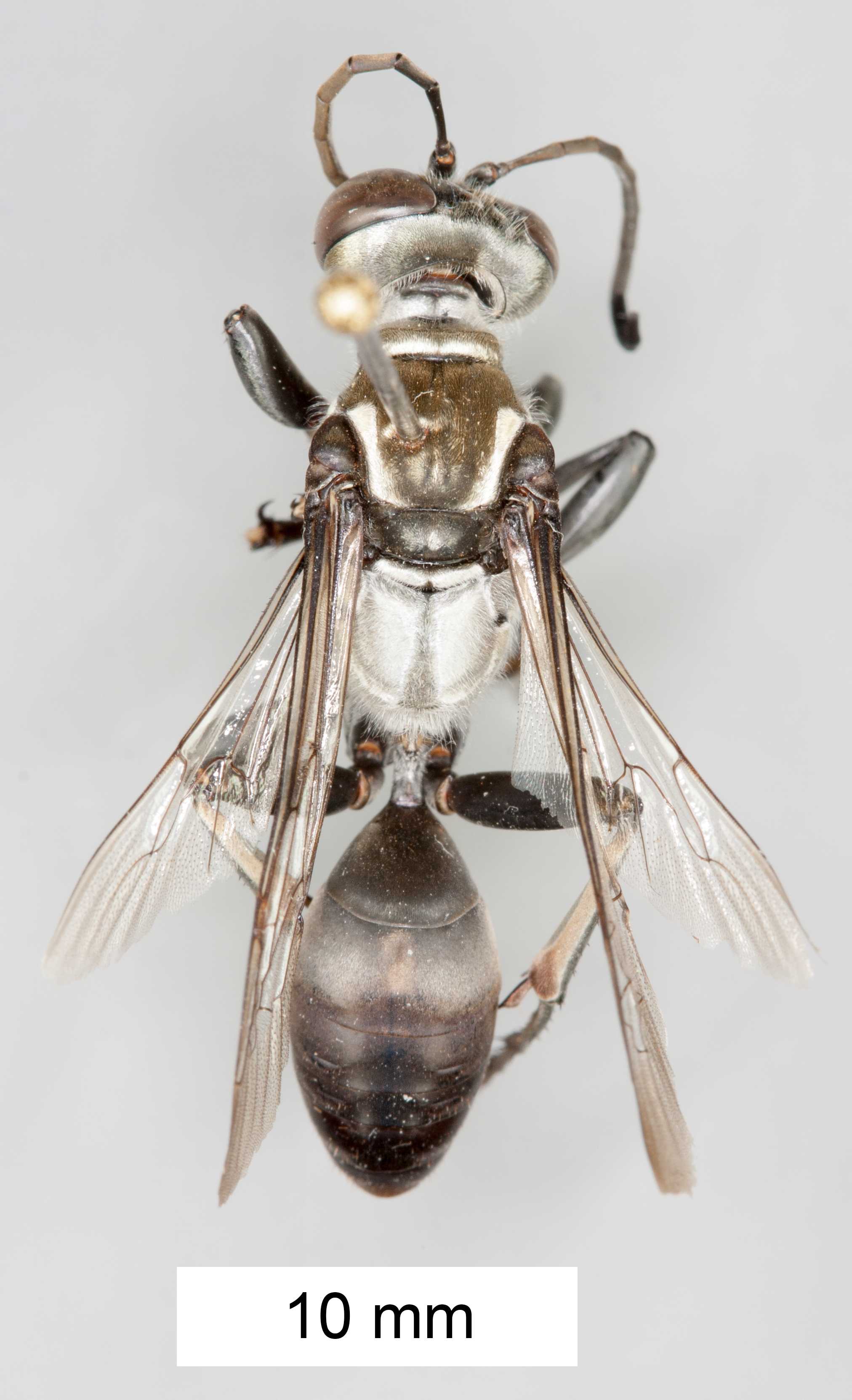

Supplement: Supplementary material 1 — Species data for genus Sphex [file zookeys-521-001-s001.zip › SphexDeltaFiles/Images/argentatissimus_m.jpg]

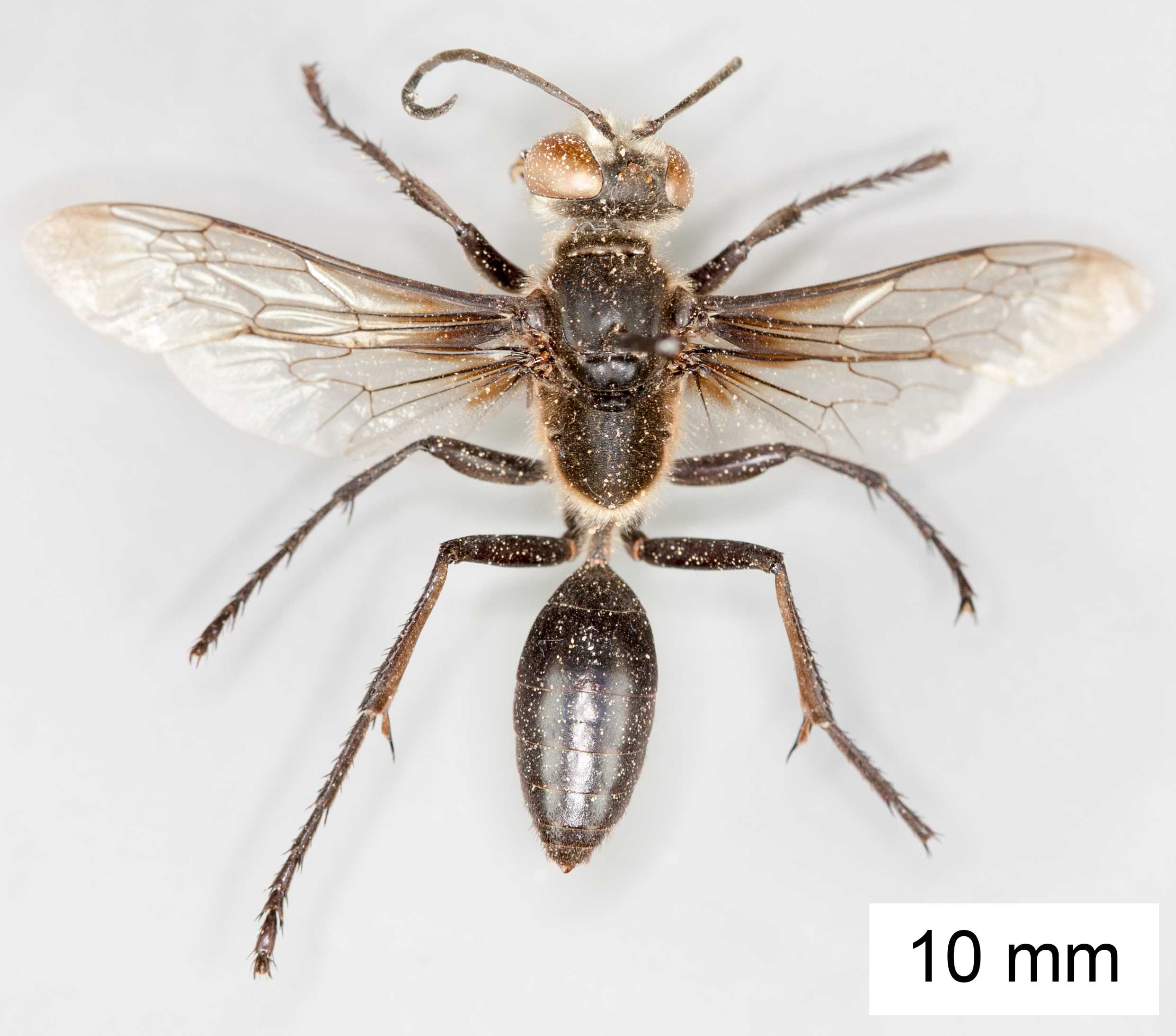

Supplement: Supplementary material 1 — Species data for genus Sphex [file zookeys-521-001-s001.zip › SphexDeltaFiles/Images/argentatus_m.jpg]

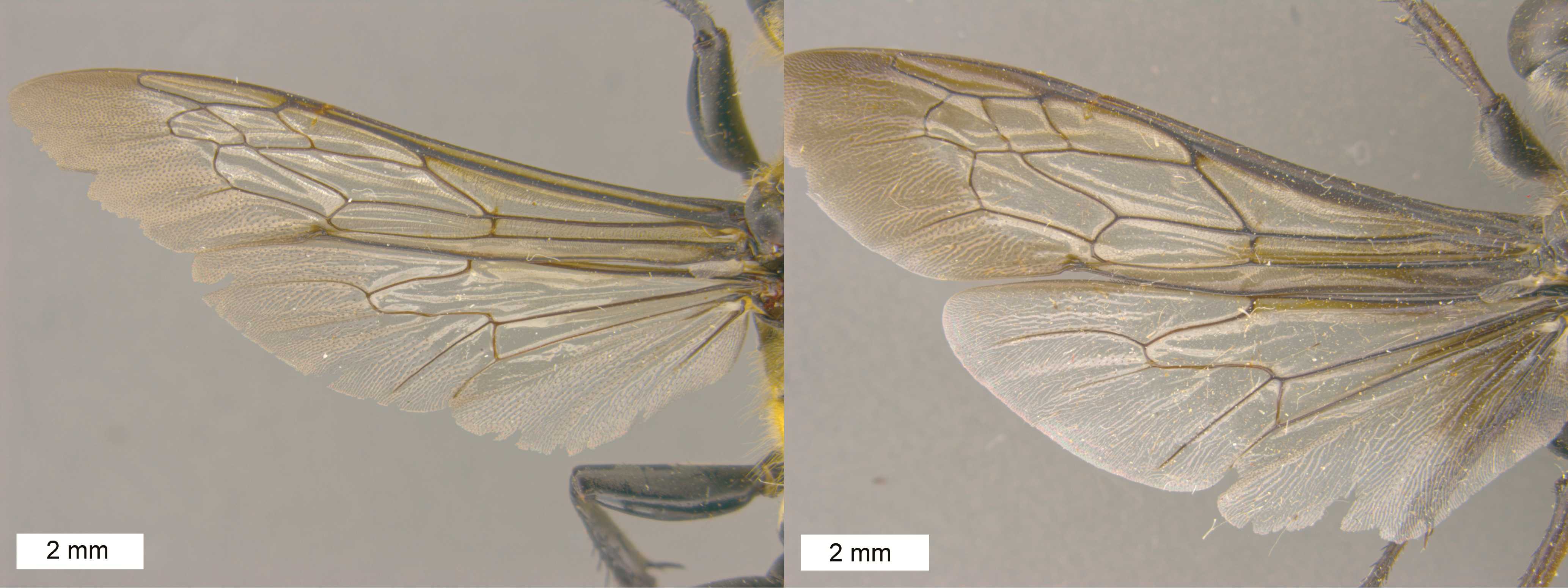

Supplement: Supplementary material 1 — Species data for genus Sphex [file zookeys-521-001-s001.zip › SphexDeltaFiles/Images/base_of_forewing.jpg]

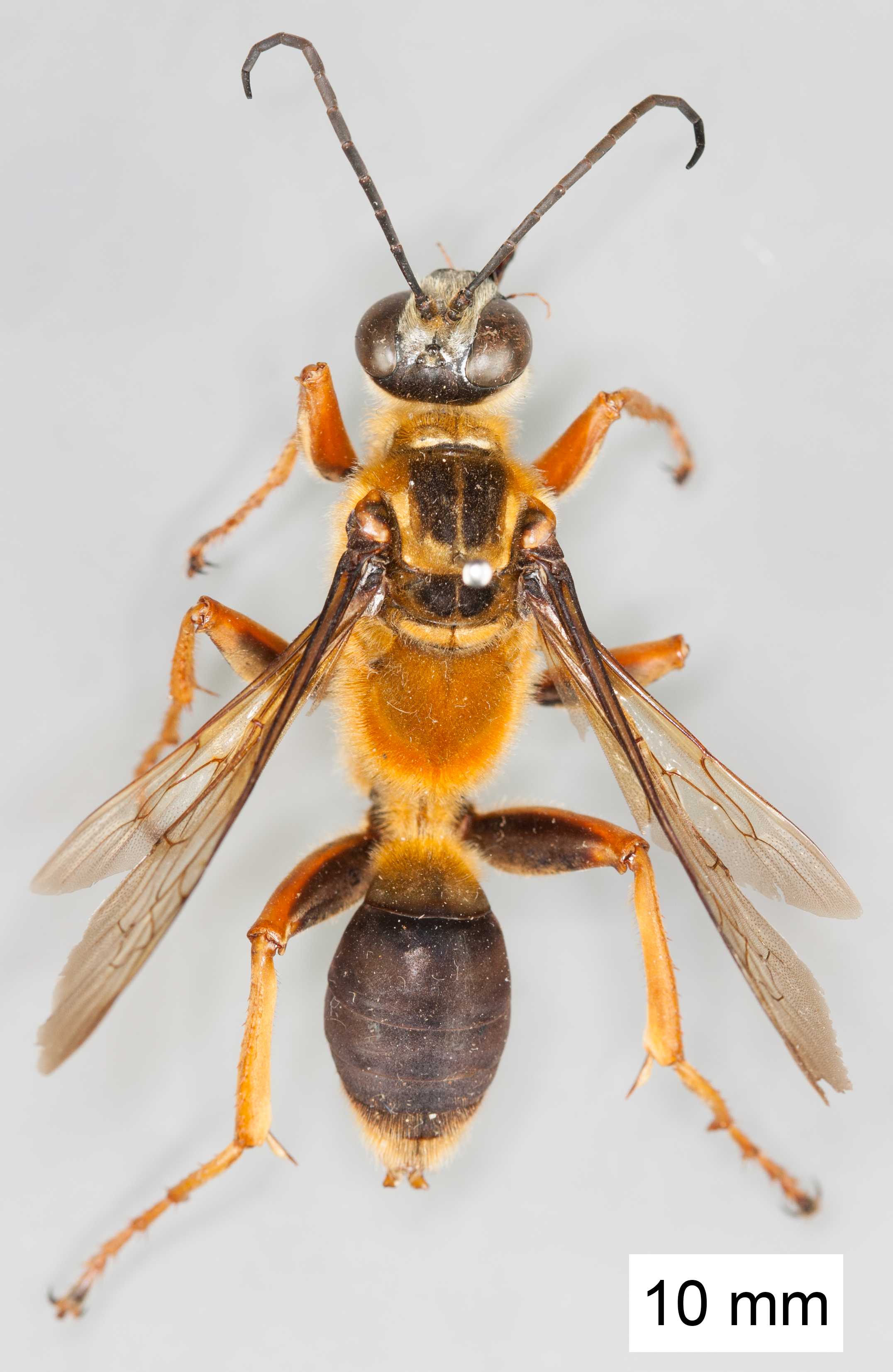

Supplement: Supplementary material 1 — Species data for genus Sphex [file zookeys-521-001-s001.zip › SphexDeltaFiles/Images/basilicus_m.jpg]

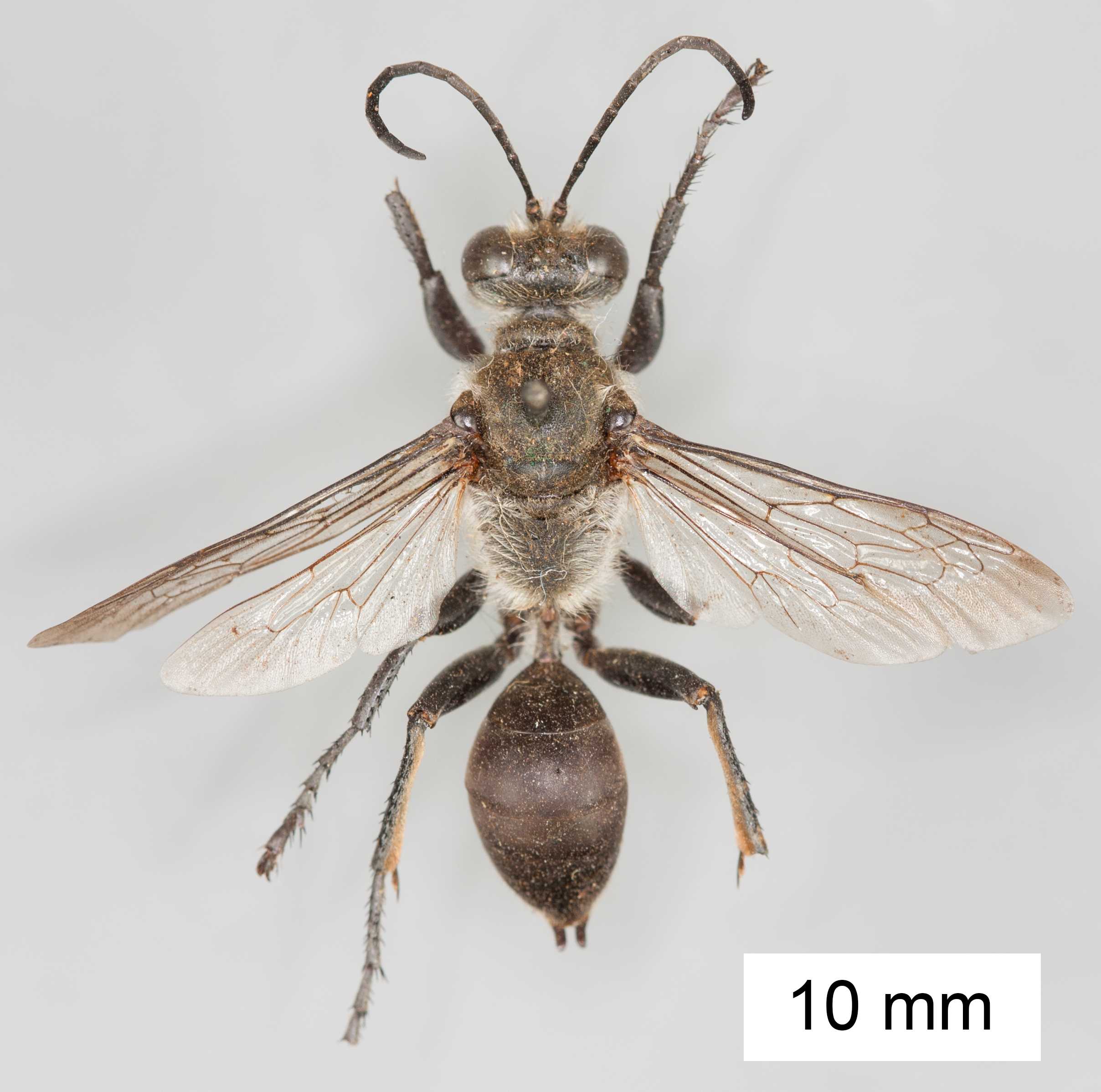

Supplement: Supplementary material 1 — Species data for genus Sphex [file zookeys-521-001-s001.zip › SphexDeltaFiles/Images/bilobatus_m.jpg]

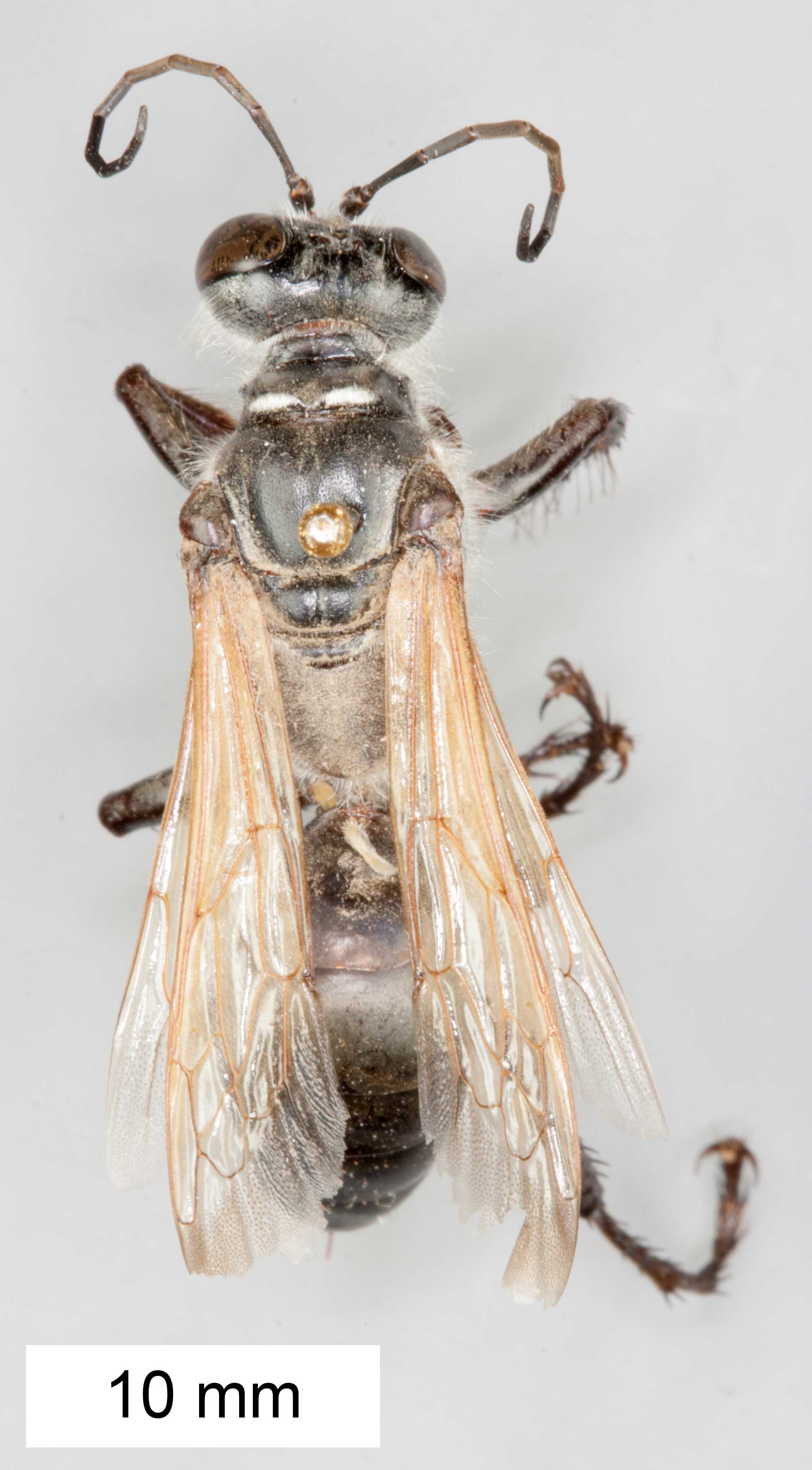

Supplement: Supplementary material 1 — Species data for genus Sphex [file zookeys-521-001-s001.zip › SphexDeltaFiles/Images/brevipetiolus_f.jpg]

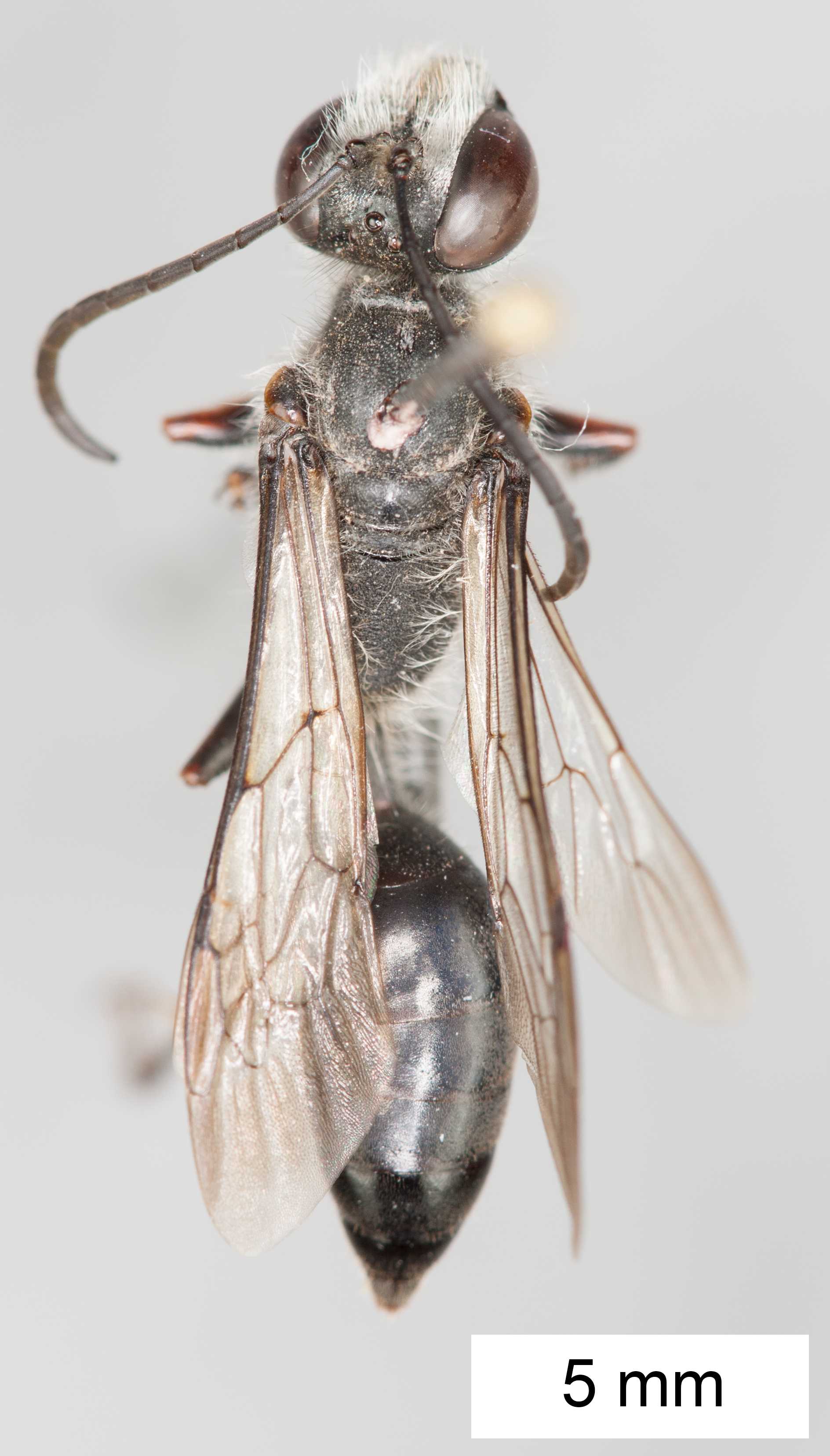

Supplement: Supplementary material 1 — Species data for genus Sphex [file zookeys-521-001-s001.zip › SphexDeltaFiles/Images/caelebs_m.jpg]

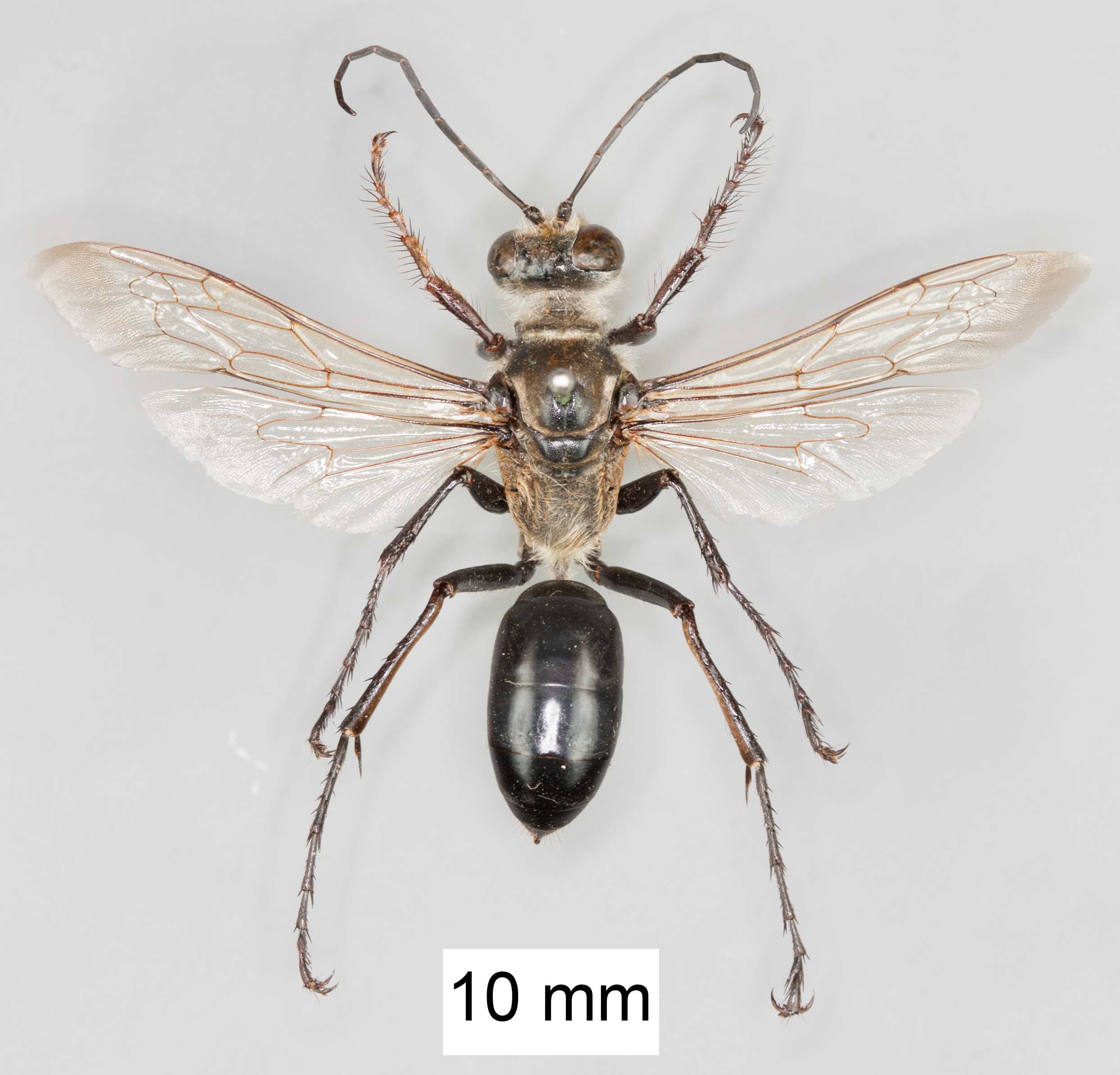

Supplement: Supplementary material 1 — Species data for genus Sphex [file zookeys-521-001-s001.zip › SphexDeltaFiles/Images/carbonicolor_f.jpg]

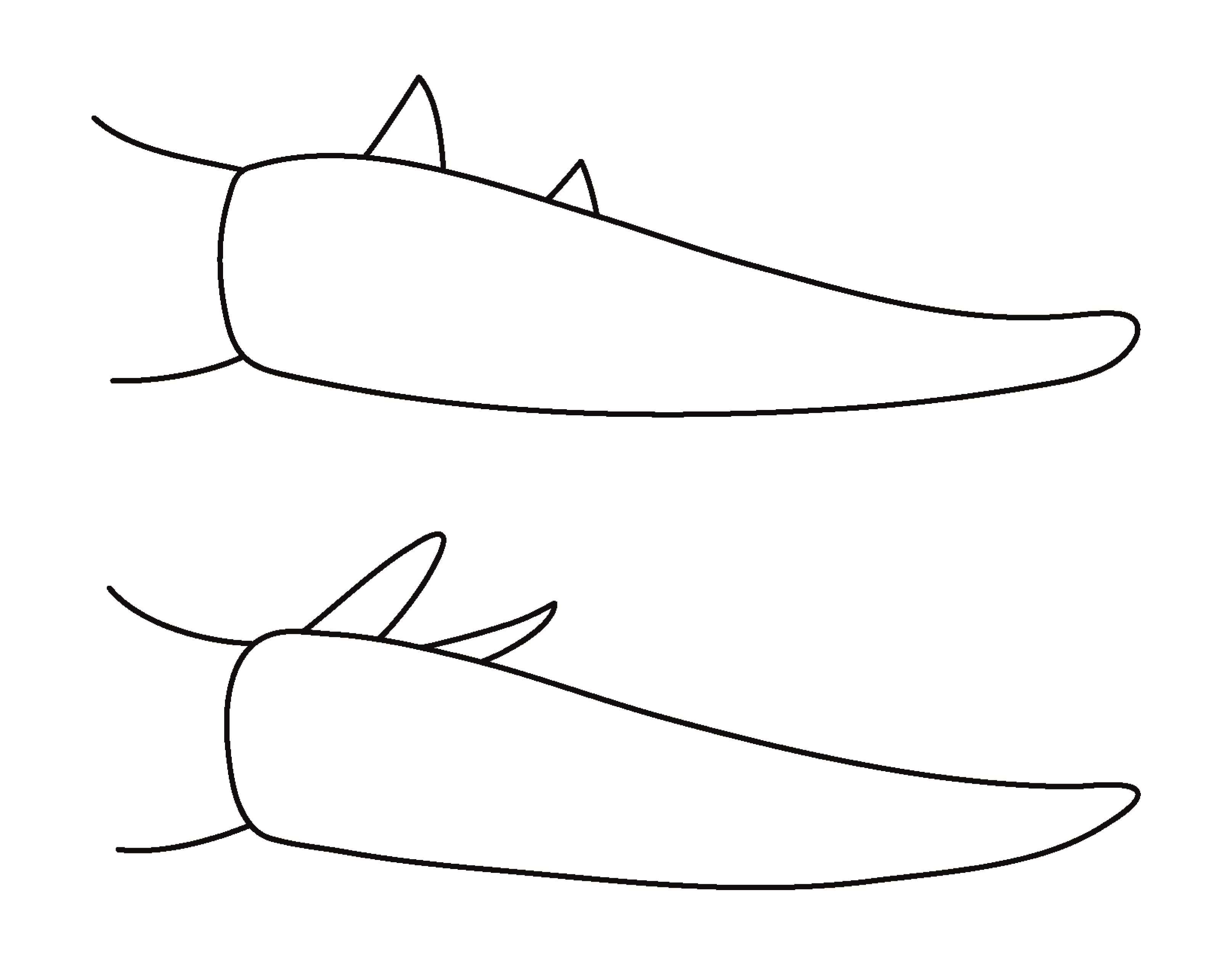

Supplement: Supplementary material 1 — Species data for genus Sphex [file zookeys-521-001-s001.zip › SphexDeltaFiles/Images/claw.jpg]

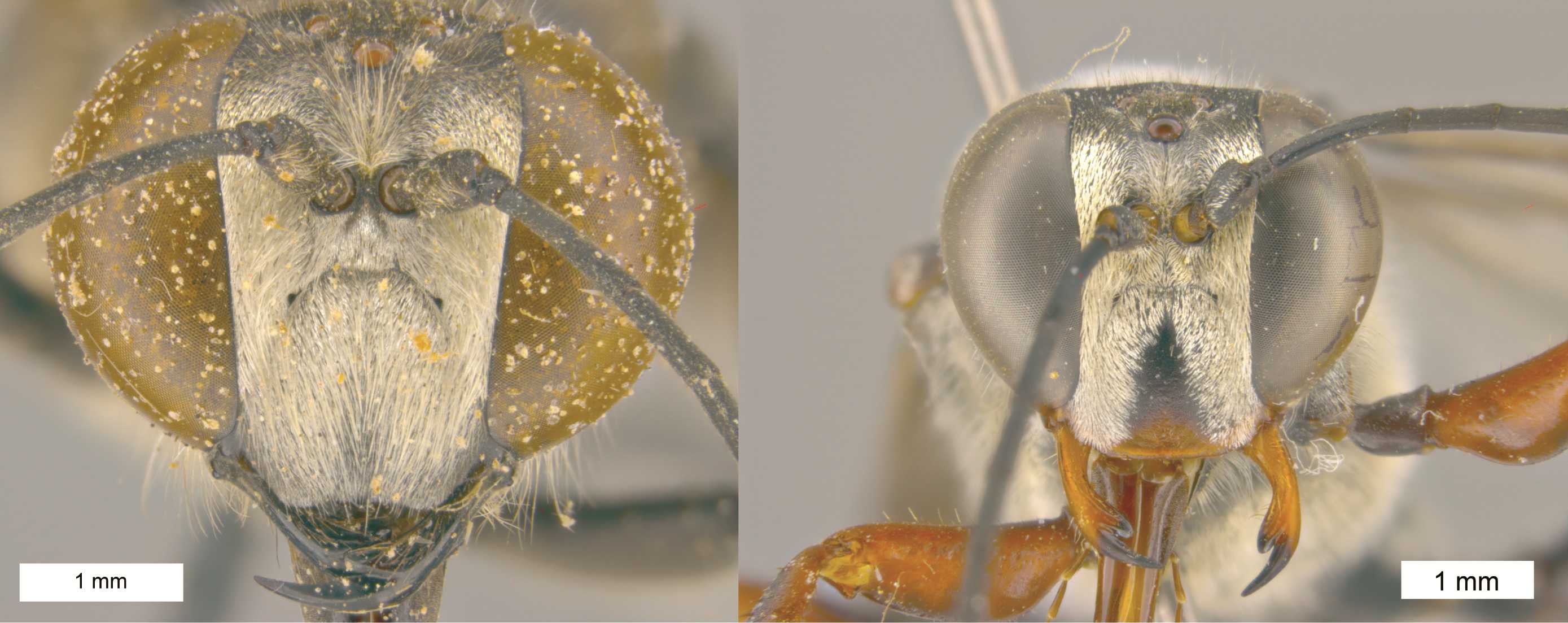

Supplement: Supplementary material 1 — Species data for genus Sphex [file zookeys-521-001-s001.zip › SphexDeltaFiles/Images/clypeus_coverage.jpg]

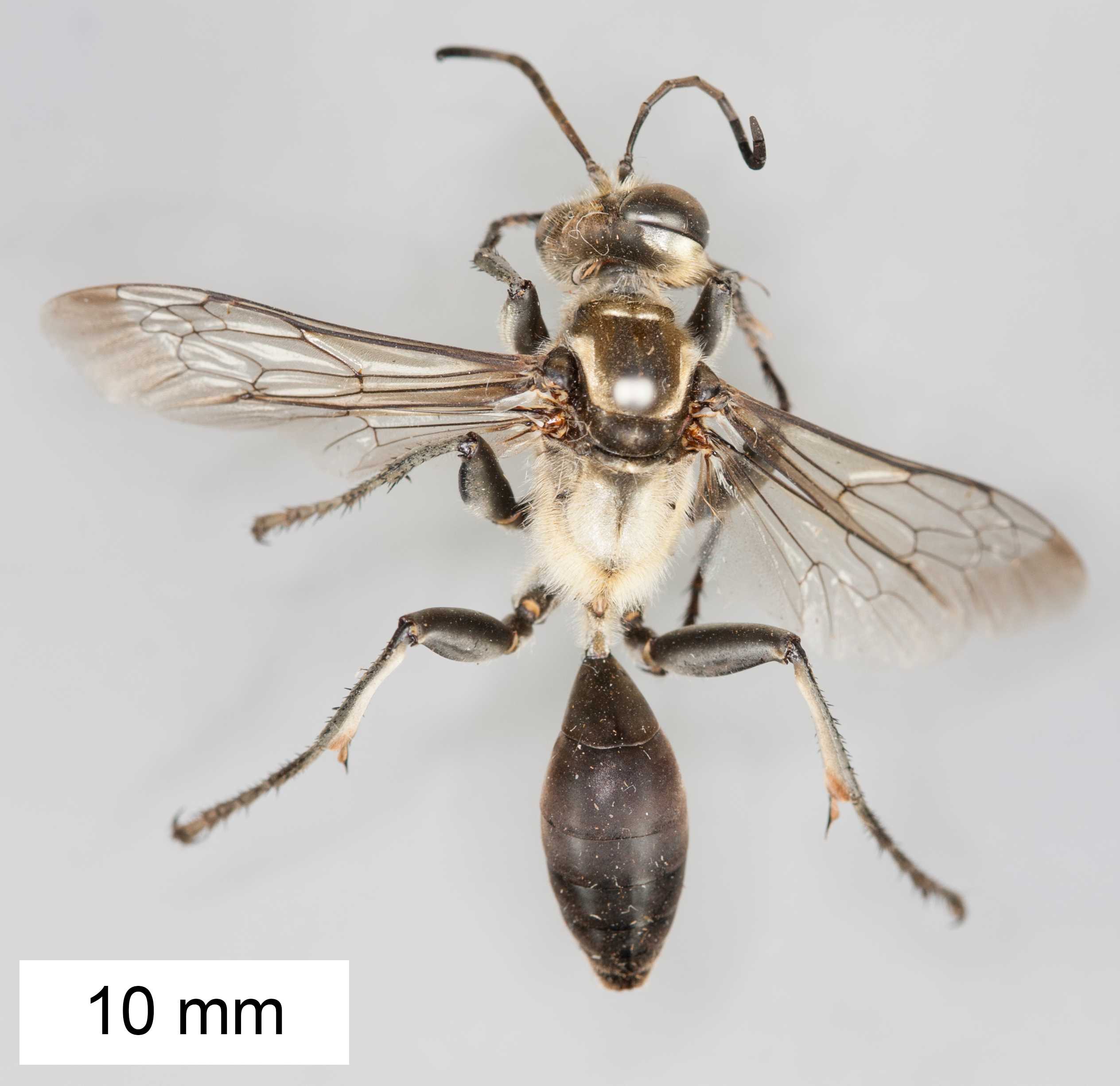

Supplement: Supplementary material 1 — Species data for genus Sphex [file zookeys-521-001-s001.zip › SphexDeltaFiles/Images/cognatus_m.jpg]

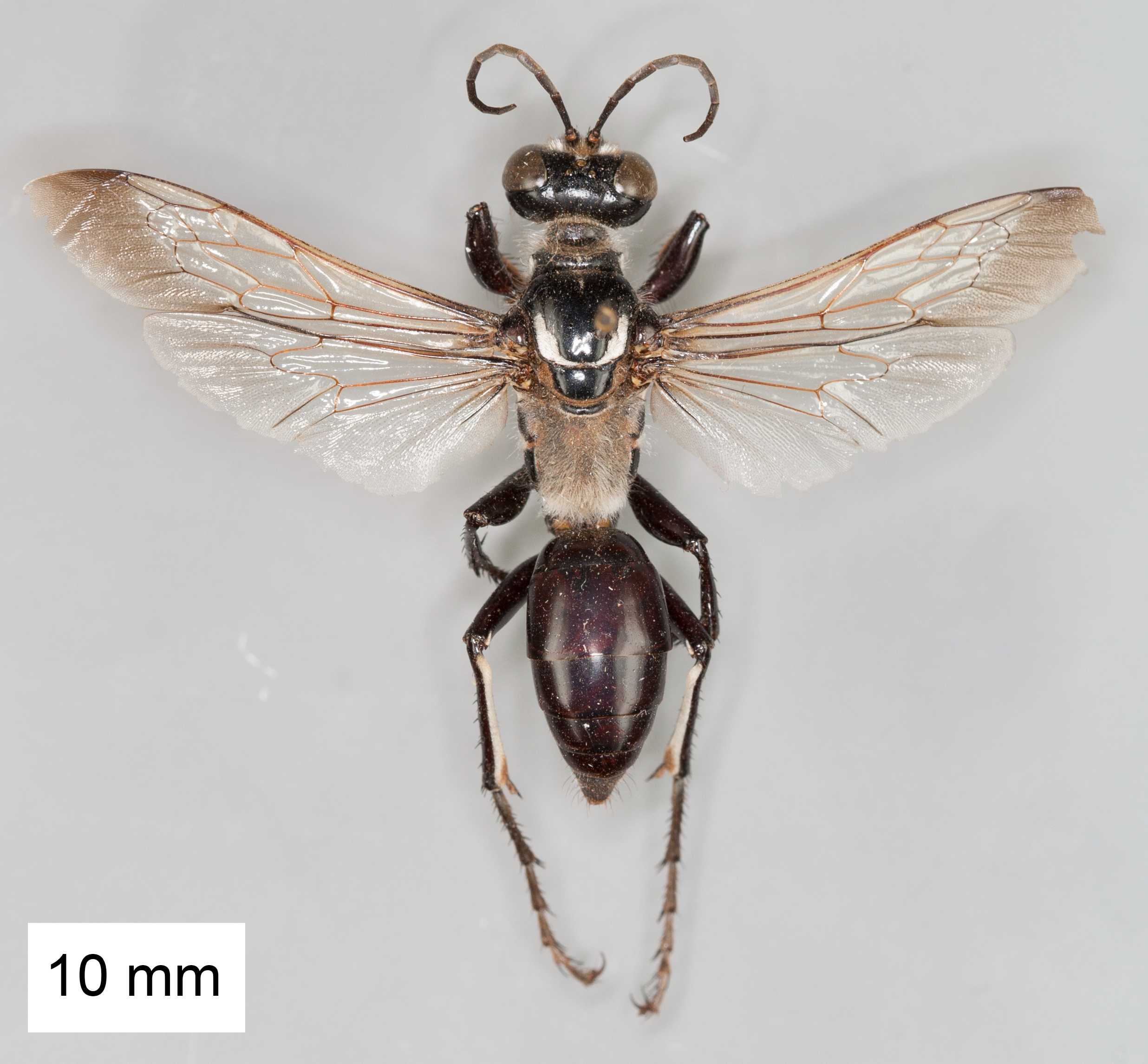

Supplement: Supplementary material 1 — Species data for genus Sphex [file zookeys-521-001-s001.zip › SphexDeltaFiles/Images/corporosus_f.jpg]

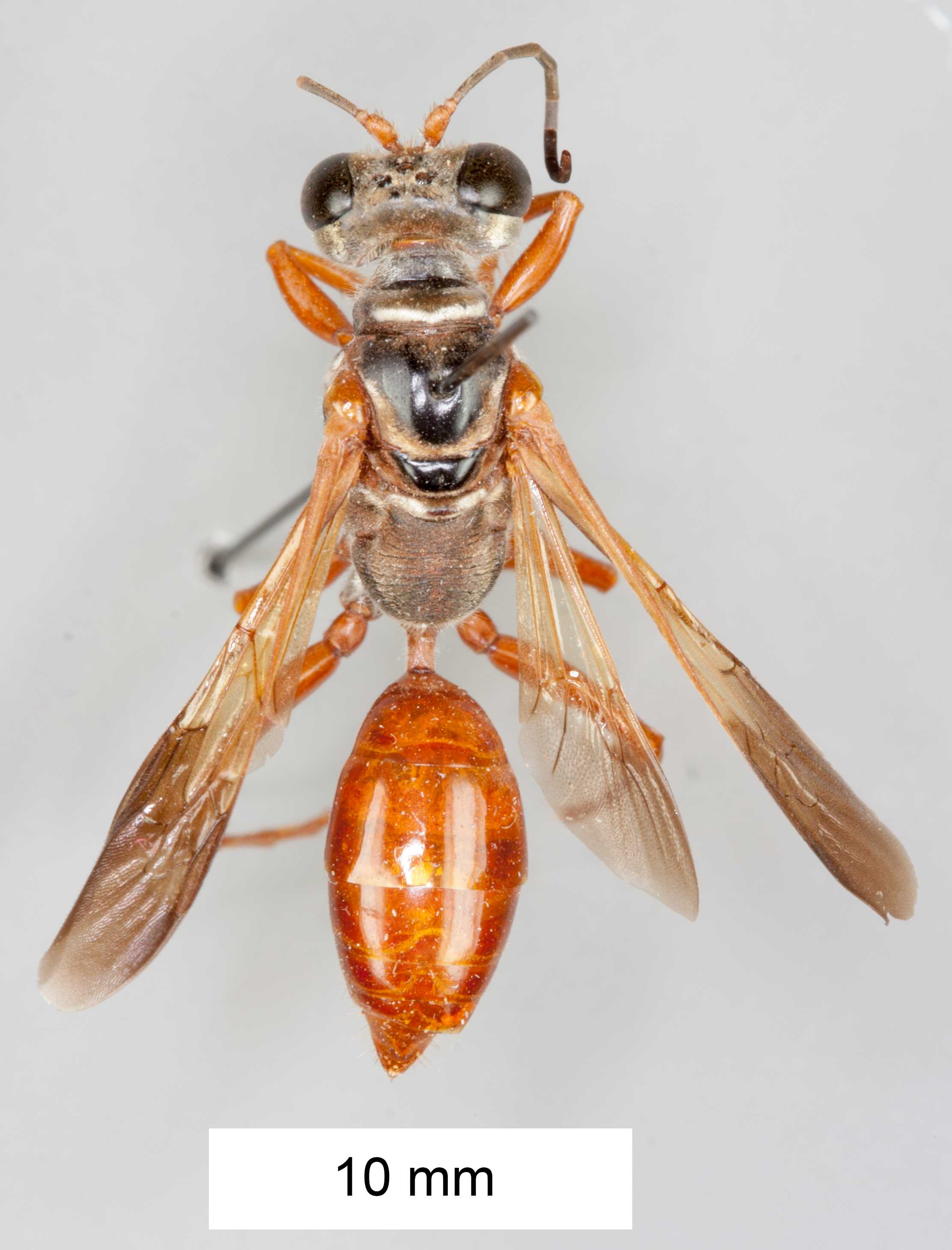

Supplement: Supplementary material 1 — Species data for genus Sphex [file zookeys-521-001-s001.zip › SphexDeltaFiles/Images/darwiniensis_f.jpg]

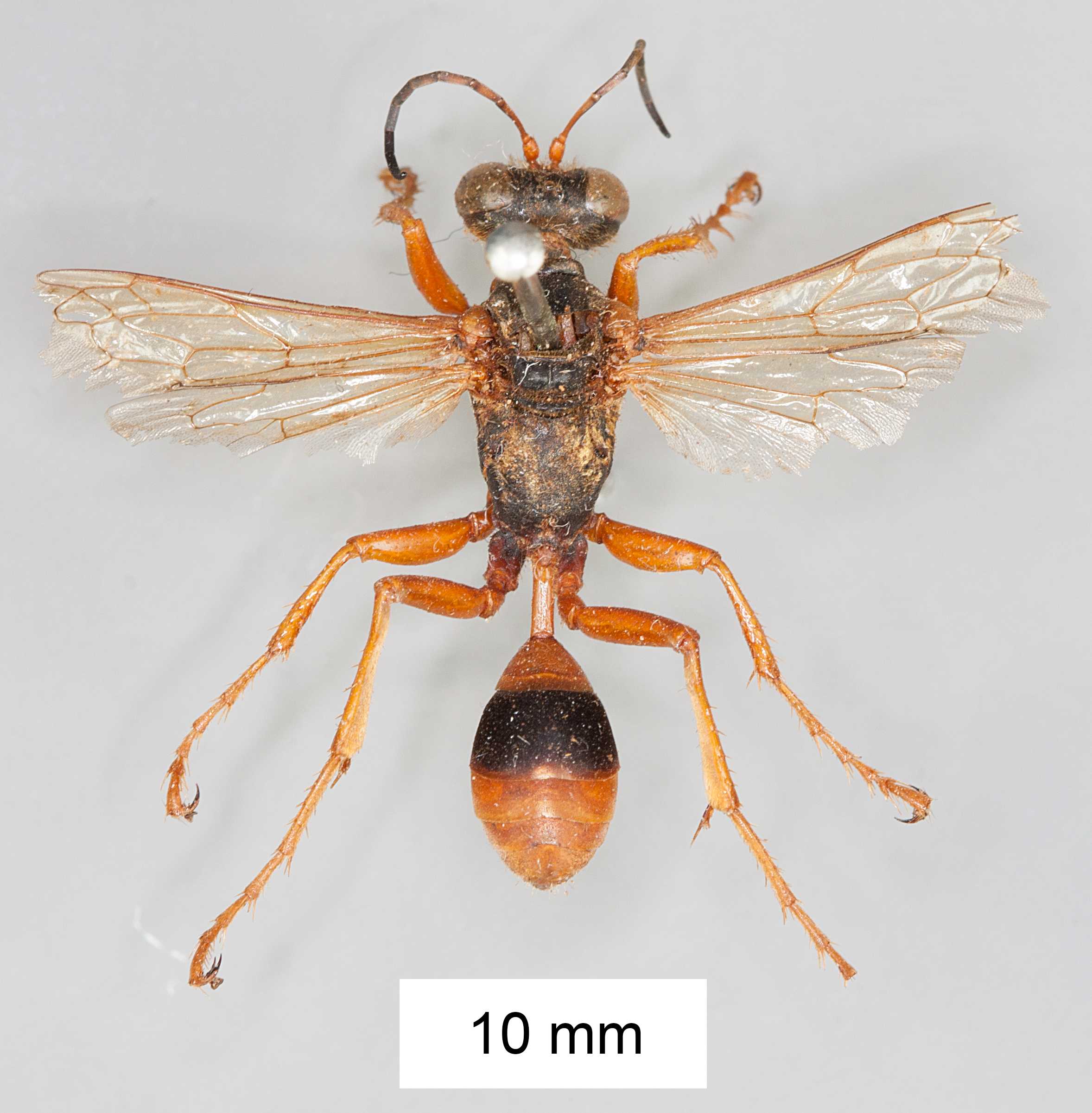

Supplement: Supplementary material 1 — Species data for genus Sphex [file zookeys-521-001-s001.zip › SphexDeltaFiles/Images/decoratus_f.jpg]

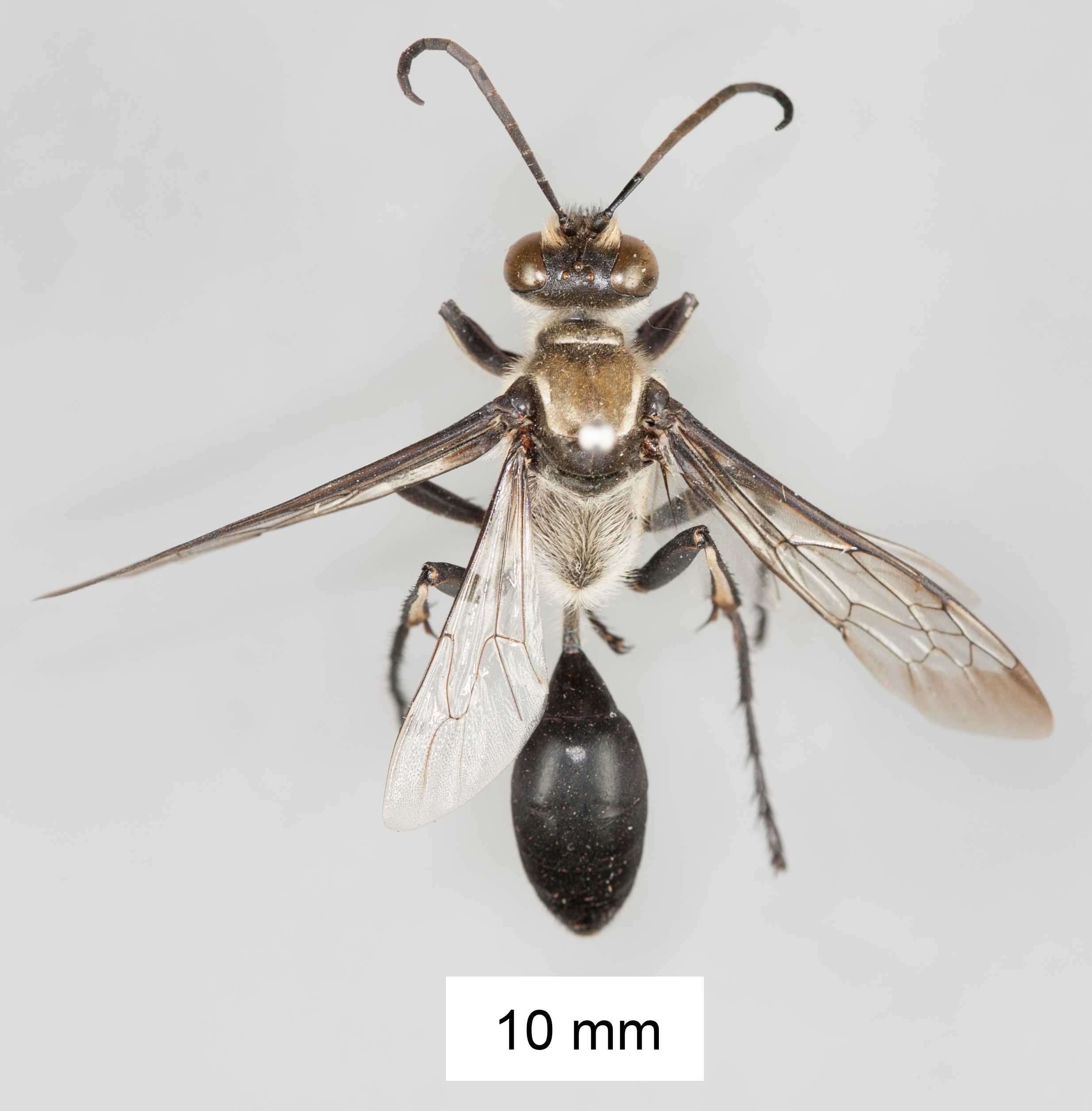

Supplement: Supplementary material 1 — Species data for genus Sphex [file zookeys-521-001-s001.zip › SphexDeltaFiles/Images/ephippium_m.jpg]

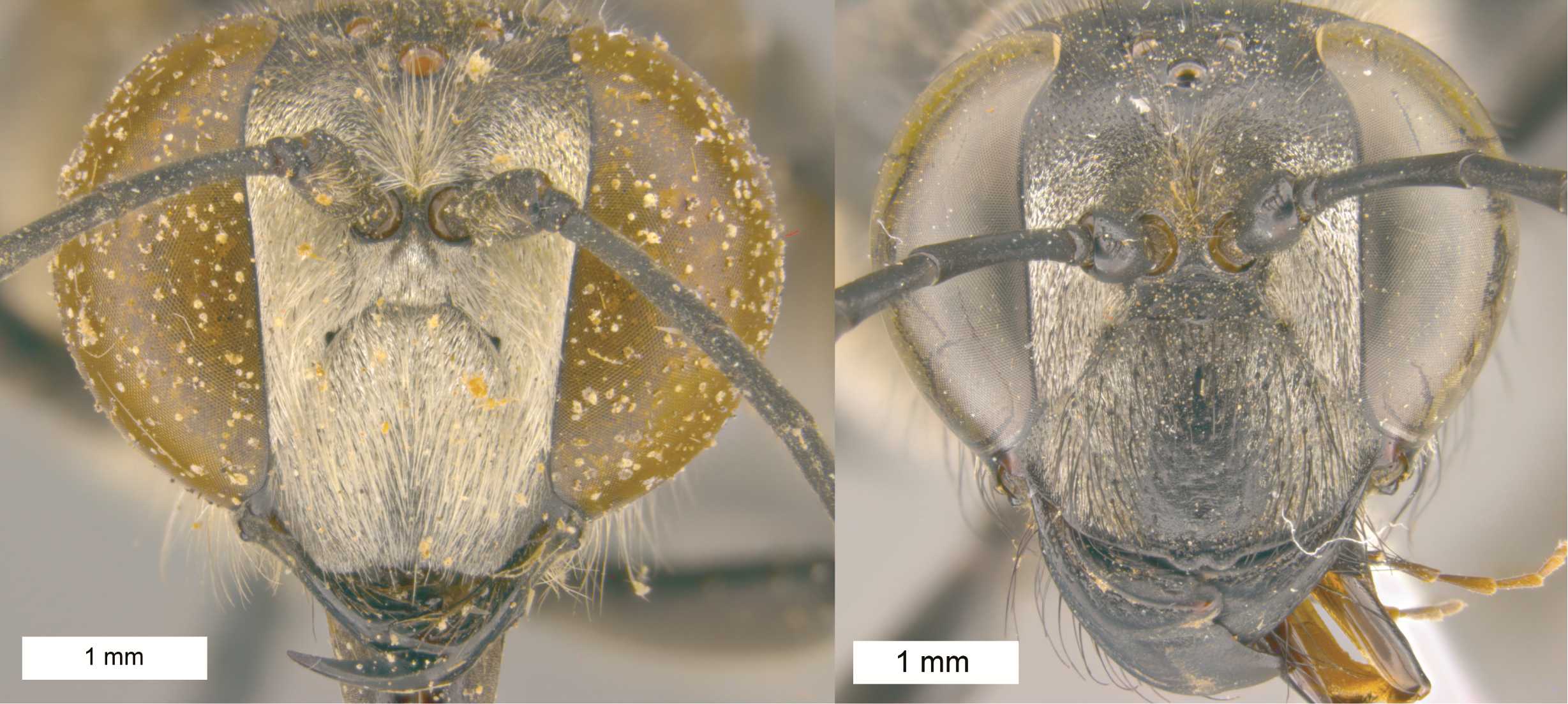

Supplement: Supplementary material 1 — Species data for genus Sphex [file zookeys-521-001-s001.zip › SphexDeltaFiles/Images/erect_setae_clypeus_color.jpg]

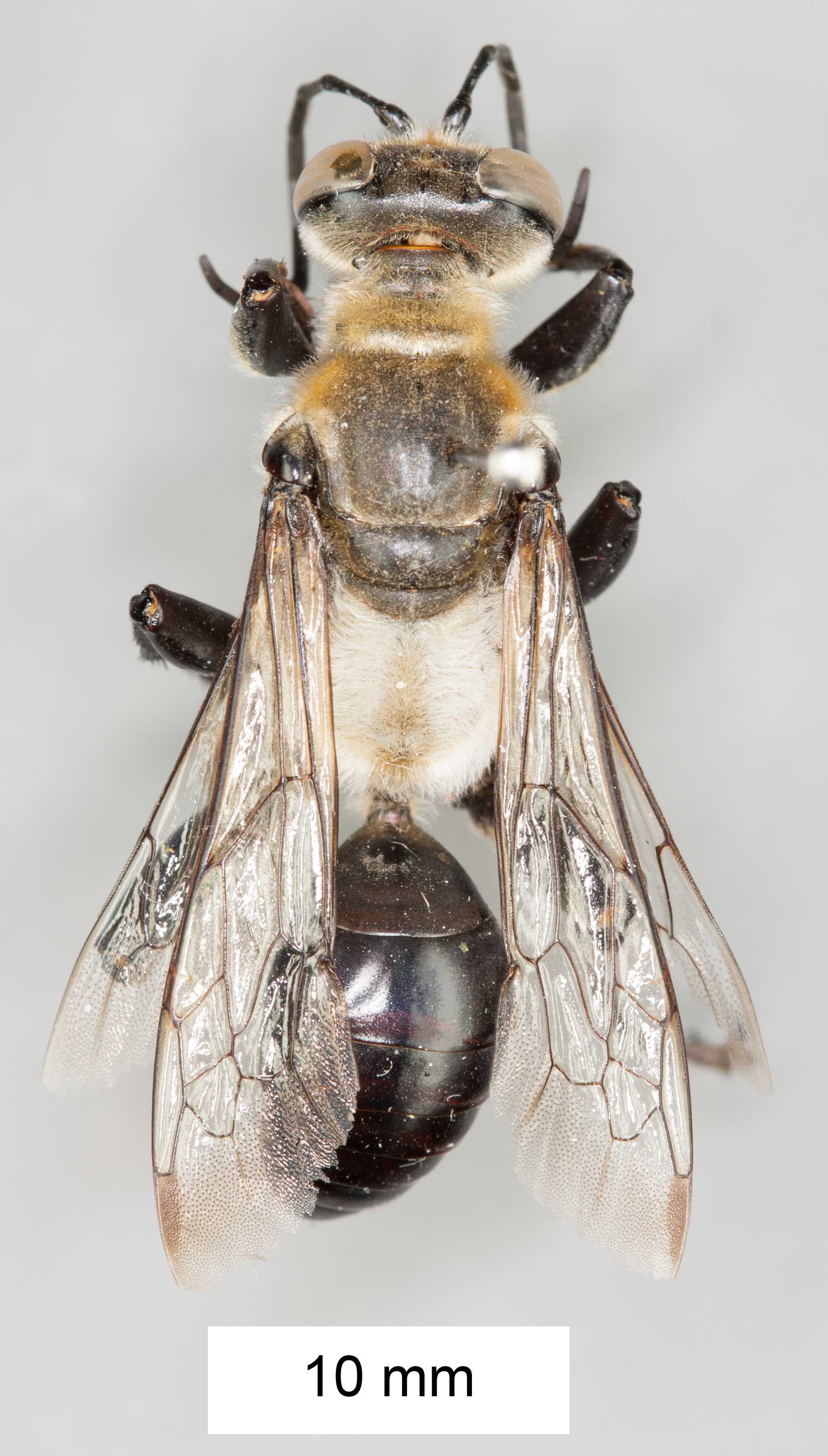

Supplement: Supplementary material 1 — Species data for genus Sphex [file zookeys-521-001-s001.zip › SphexDeltaFiles/Images/ermineus_m.jpg]

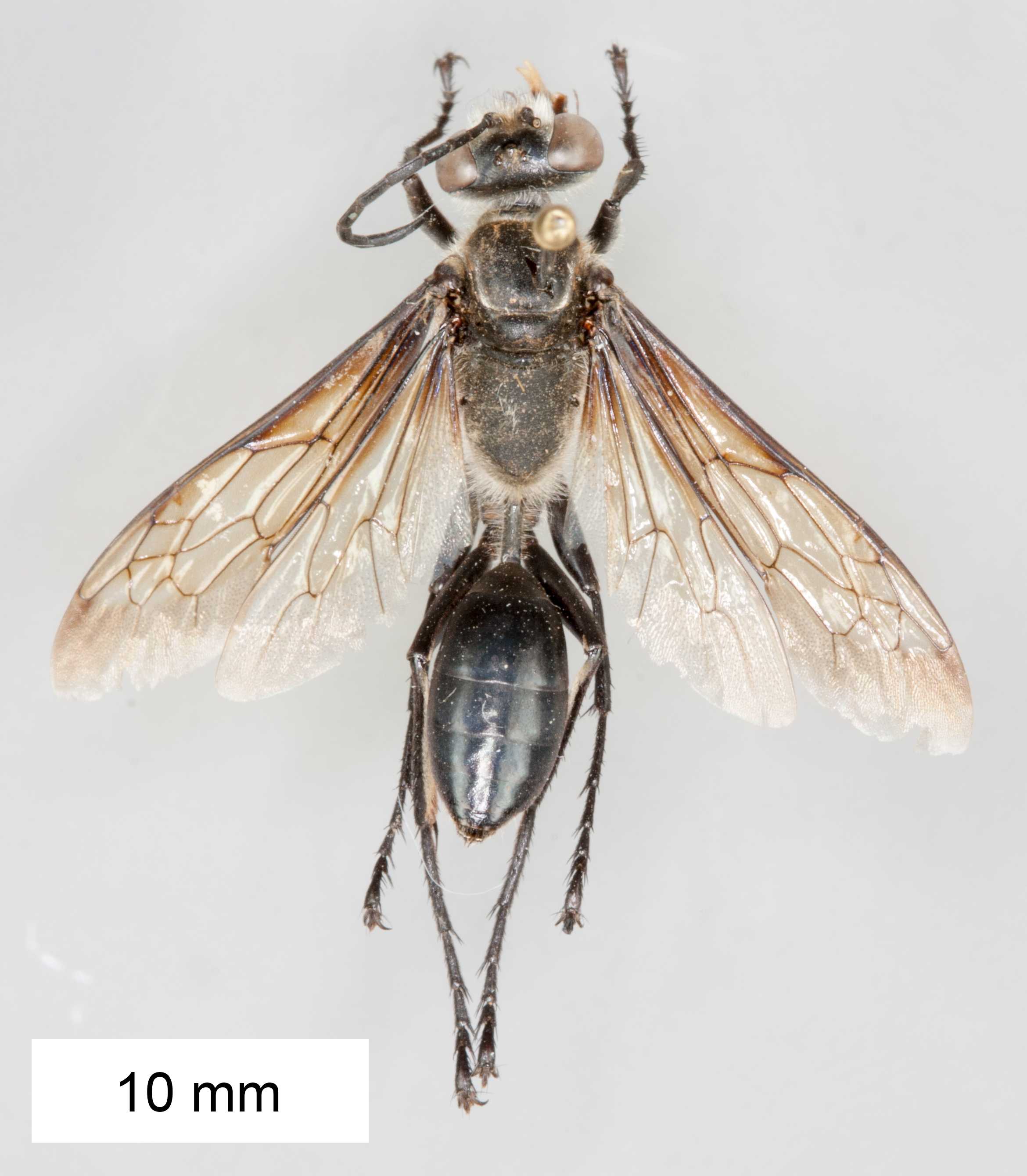

Supplement: Supplementary material 1 — Species data for genus Sphex [file zookeys-521-001-s001.zip › SphexDeltaFiles/Images/finschii_m1.jpg]

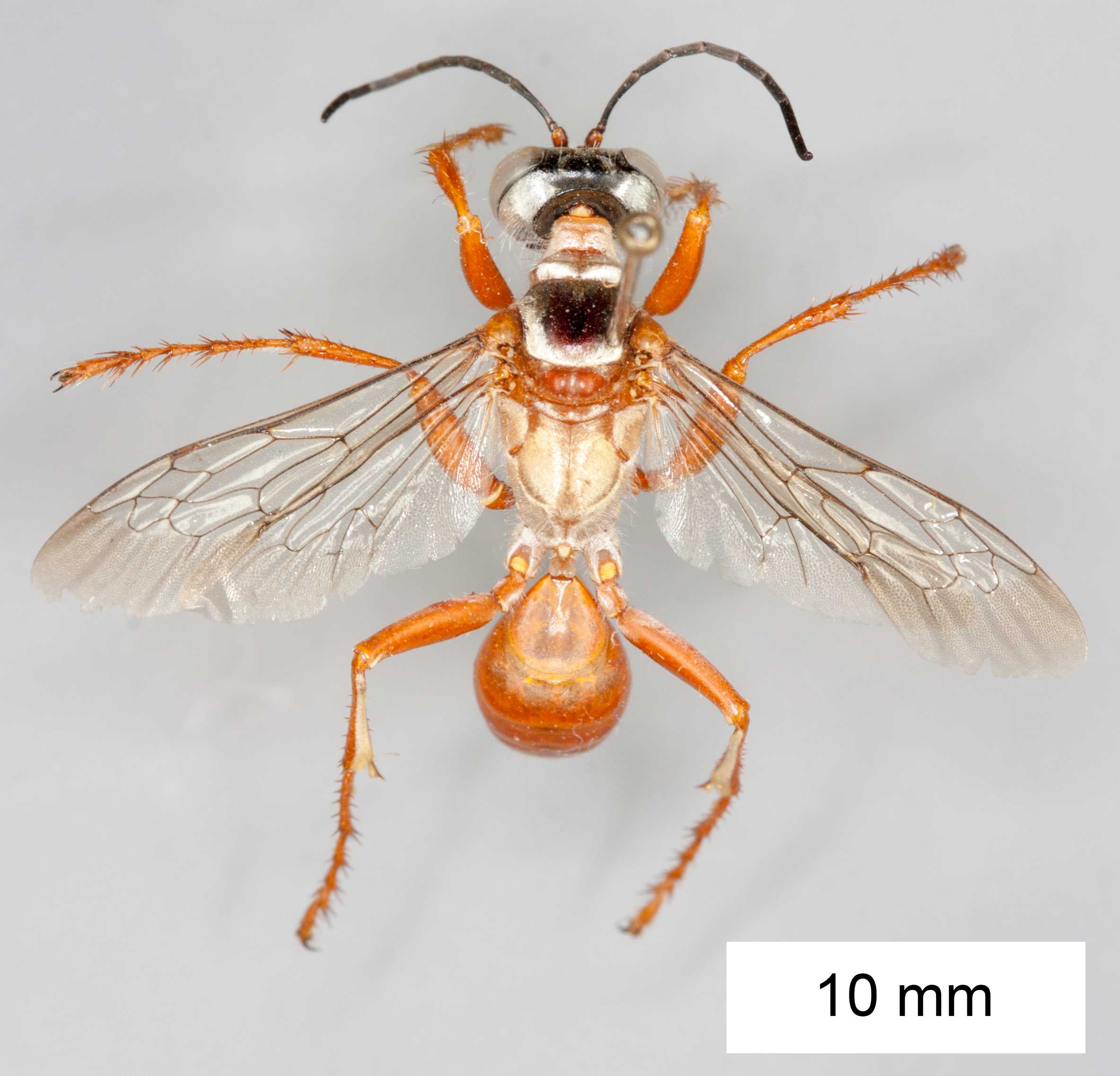

Supplement: Supplementary material 1 — Species data for genus Sphex [file zookeys-521-001-s001.zip › SphexDeltaFiles/Images/flammeus_f.jpg]

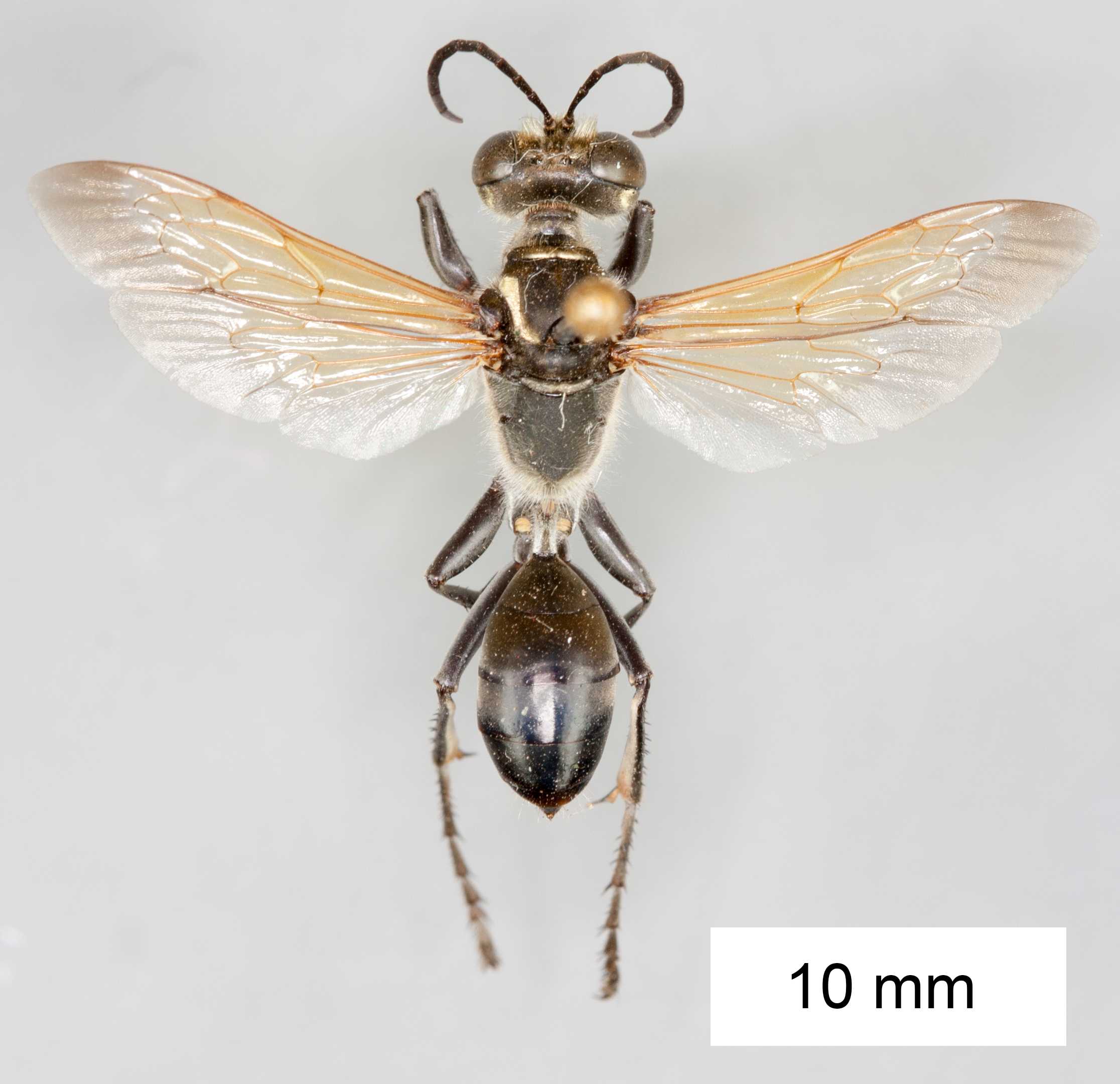

Supplement: Supplementary material 1 — Species data for genus Sphex [file zookeys-521-001-s001.zip › SphexDeltaFiles/Images/formosellus_f.jpg]

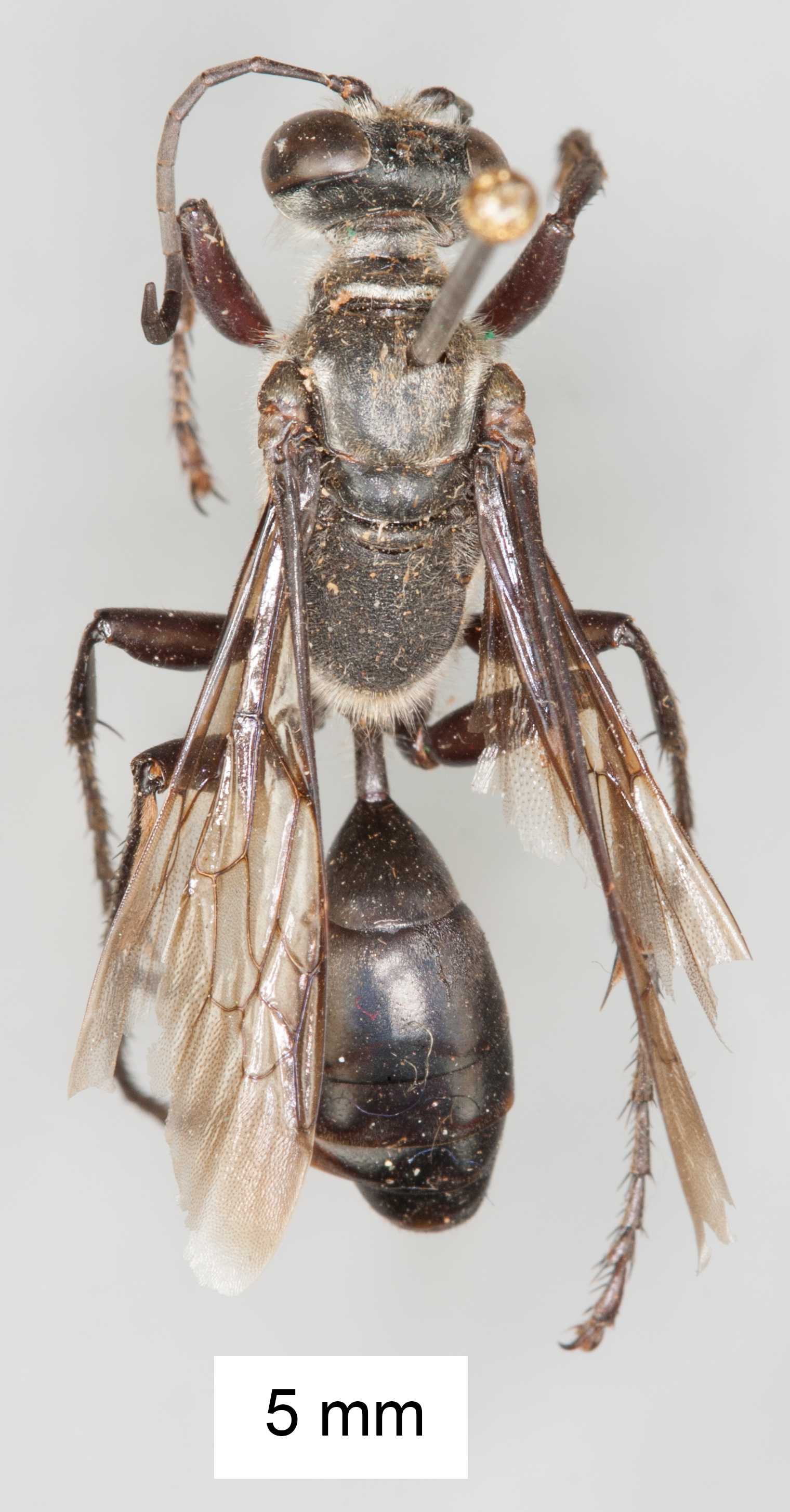

Supplement: Supplementary material 1 — Species data for genus Sphex [file zookeys-521-001-s001.zip › SphexDeltaFiles/Images/fortunatus_m.jpg]

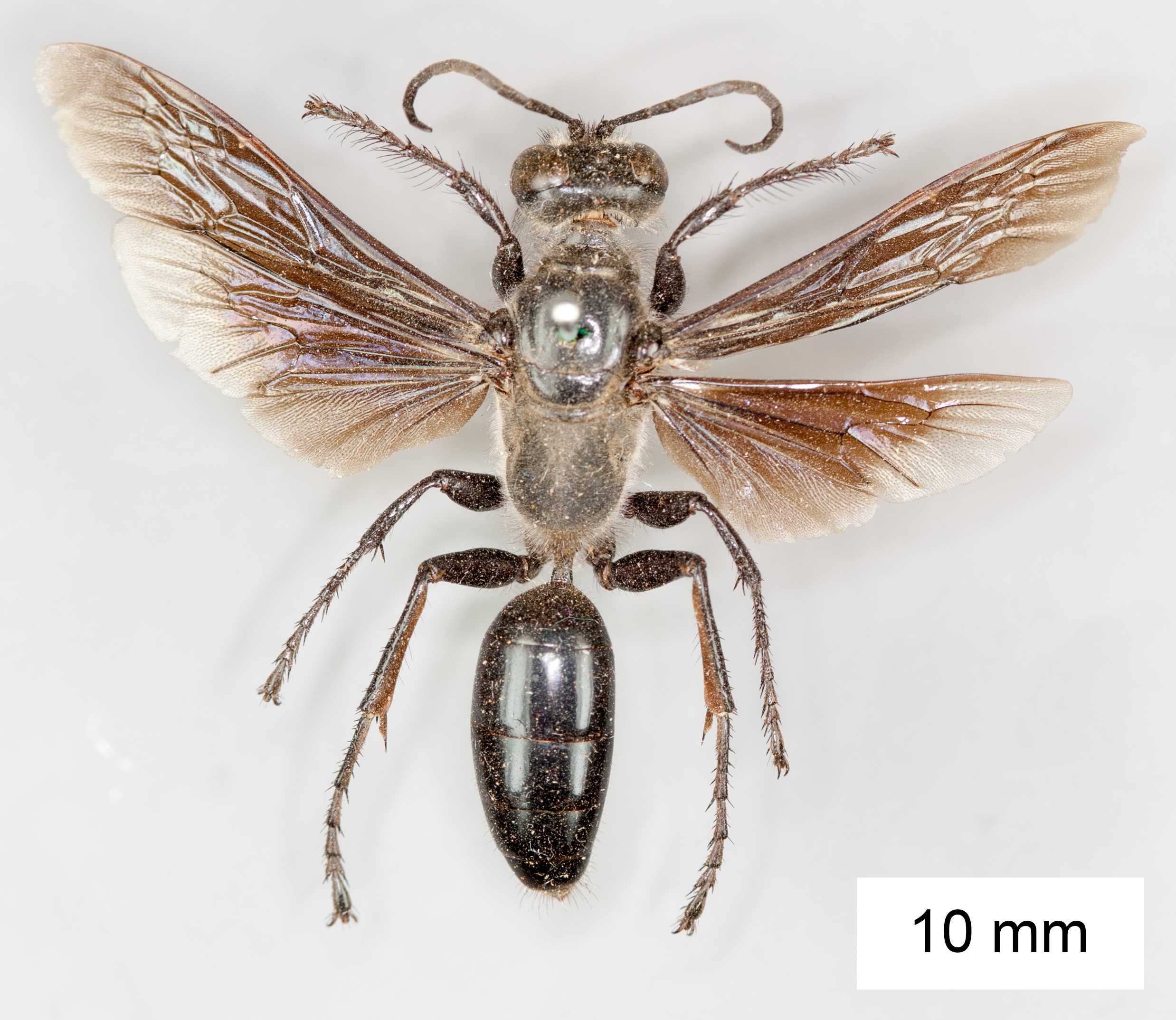

Supplement: Supplementary material 1 — Species data for genus Sphex [file zookeys-521-001-s001.zip › SphexDeltaFiles/Images/fumipennis_f.jpg]

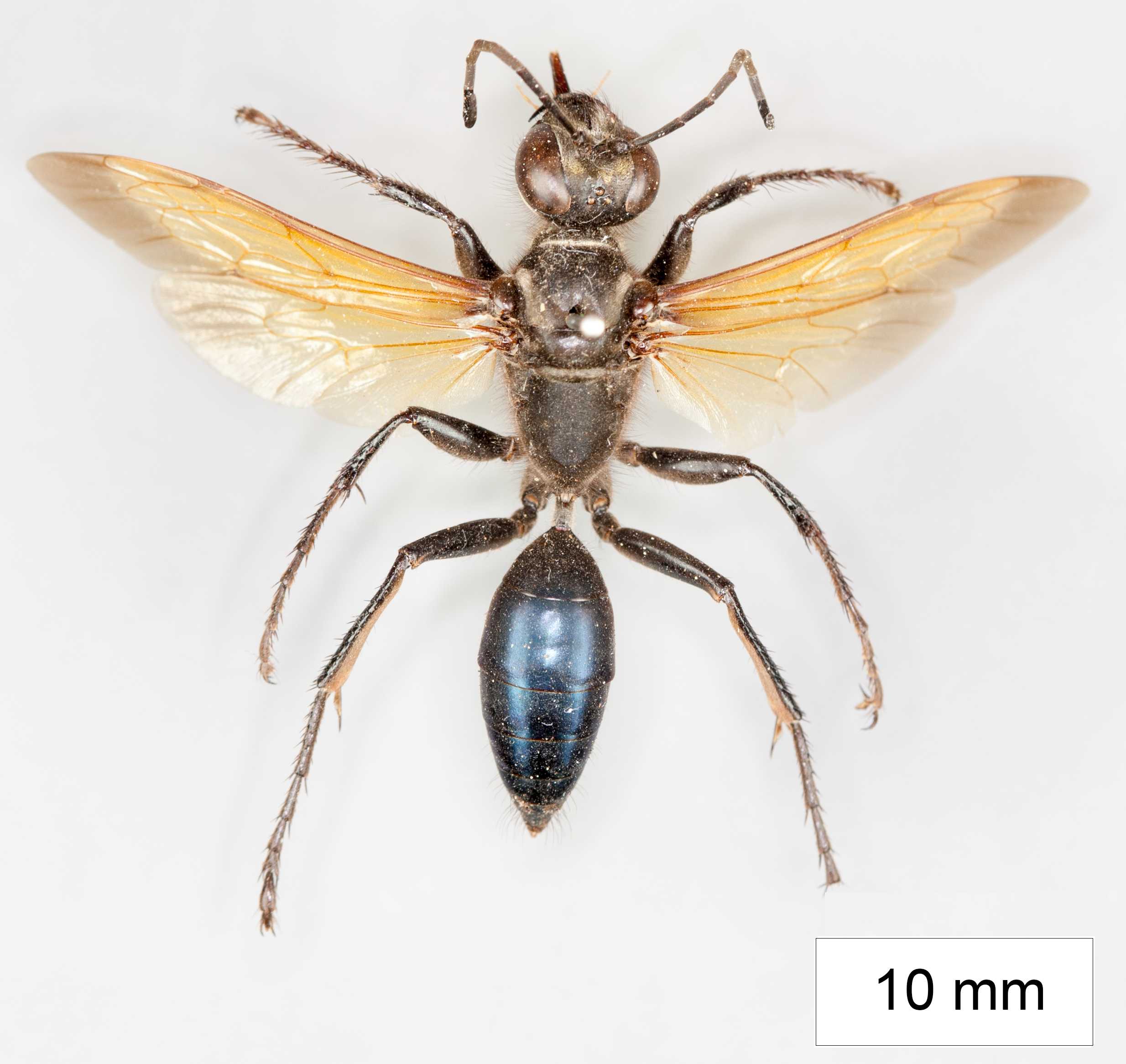

Supplement: Supplementary material 1 — Species data for genus Sphex [file zookeys-521-001-s001.zip › SphexDeltaFiles/Images/gilberti_f.jpg]

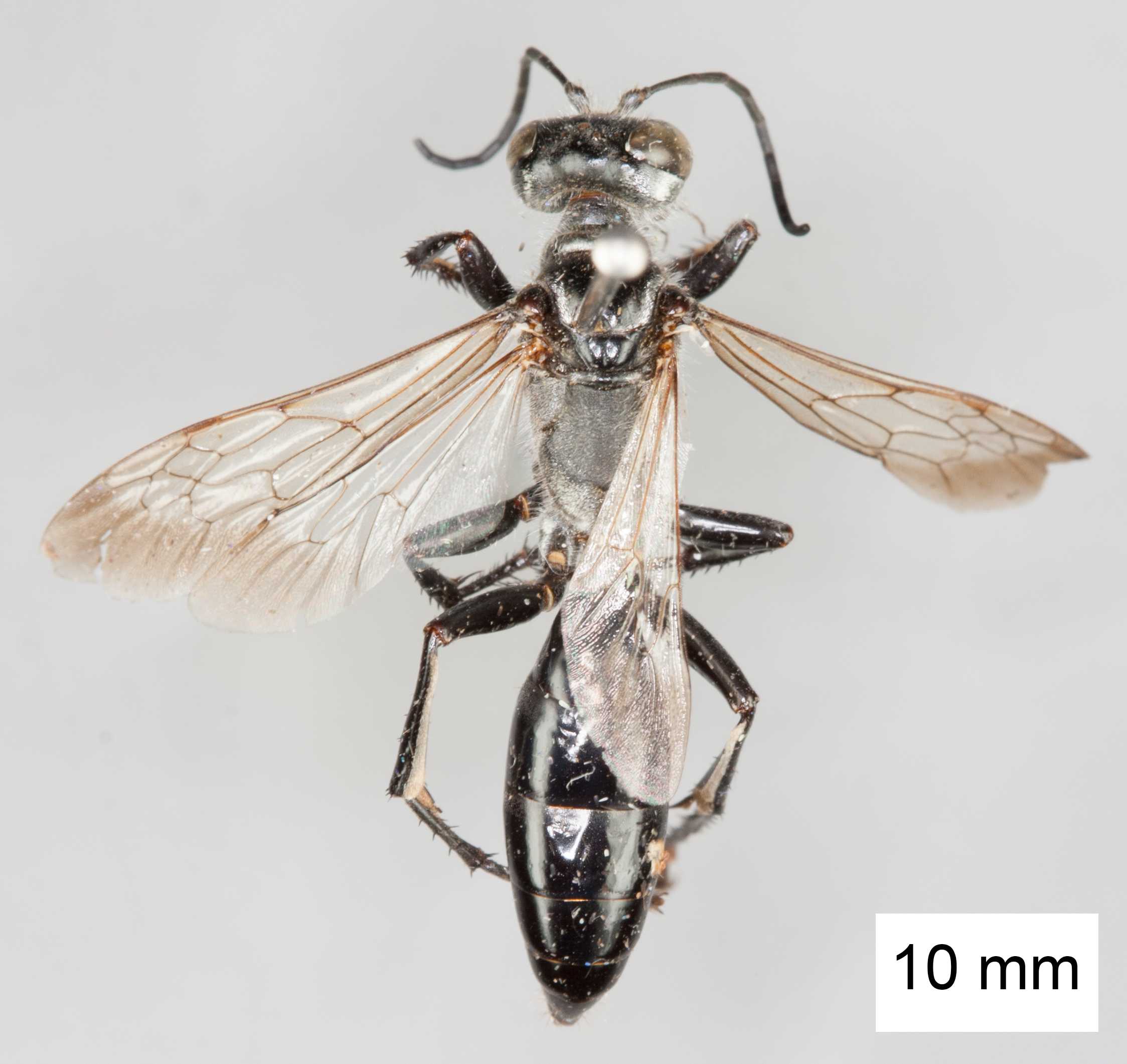

Supplement: Supplementary material 1 — Species data for genus Sphex [file zookeys-521-001-s001.zip › SphexDeltaFiles/Images/gracilis_f.jpg]

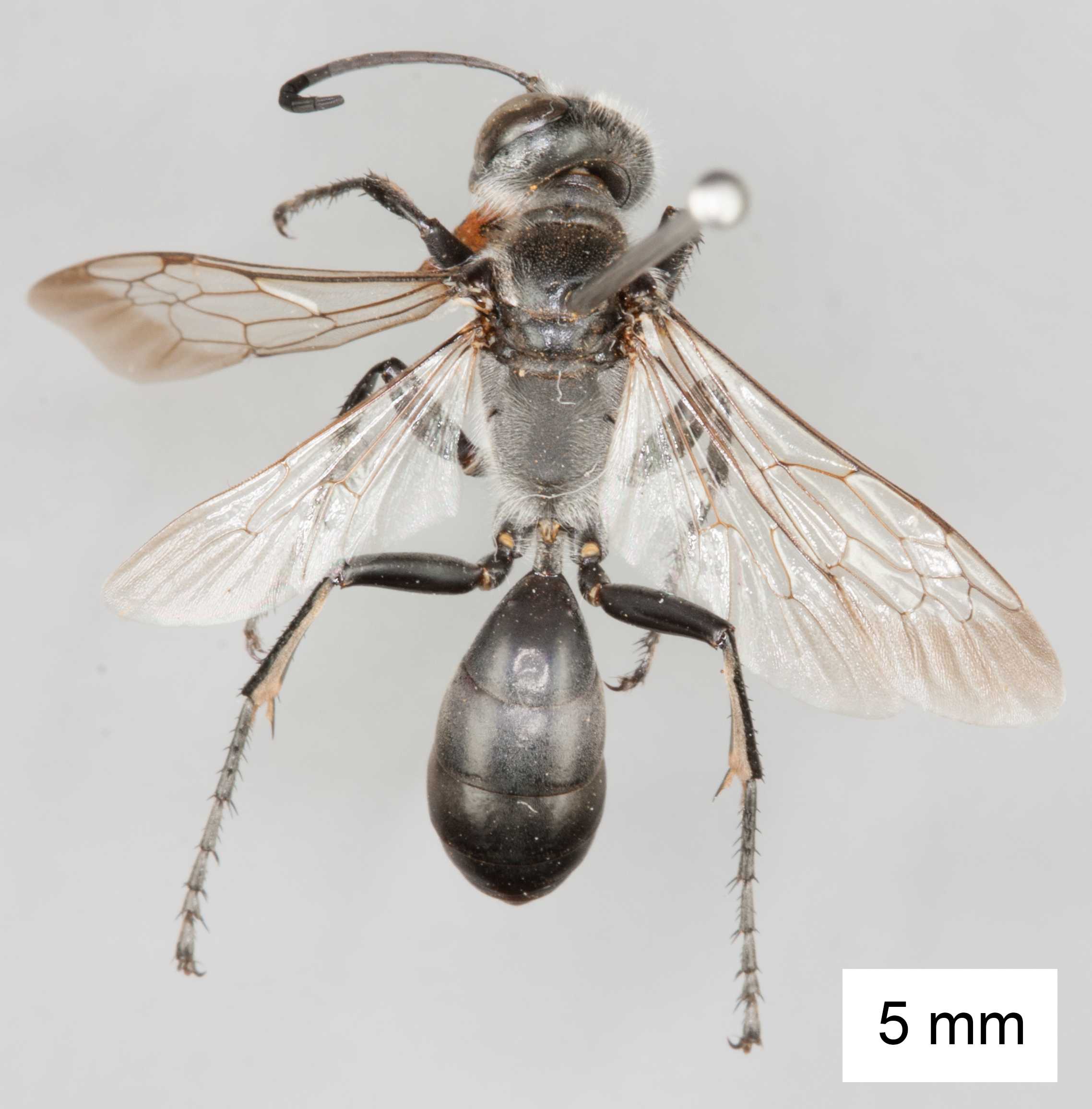

Supplement: Supplementary material 1 — Species data for genus Sphex [file zookeys-521-001-s001.zip › SphexDeltaFiles/Images/gracilis_m.jpg]

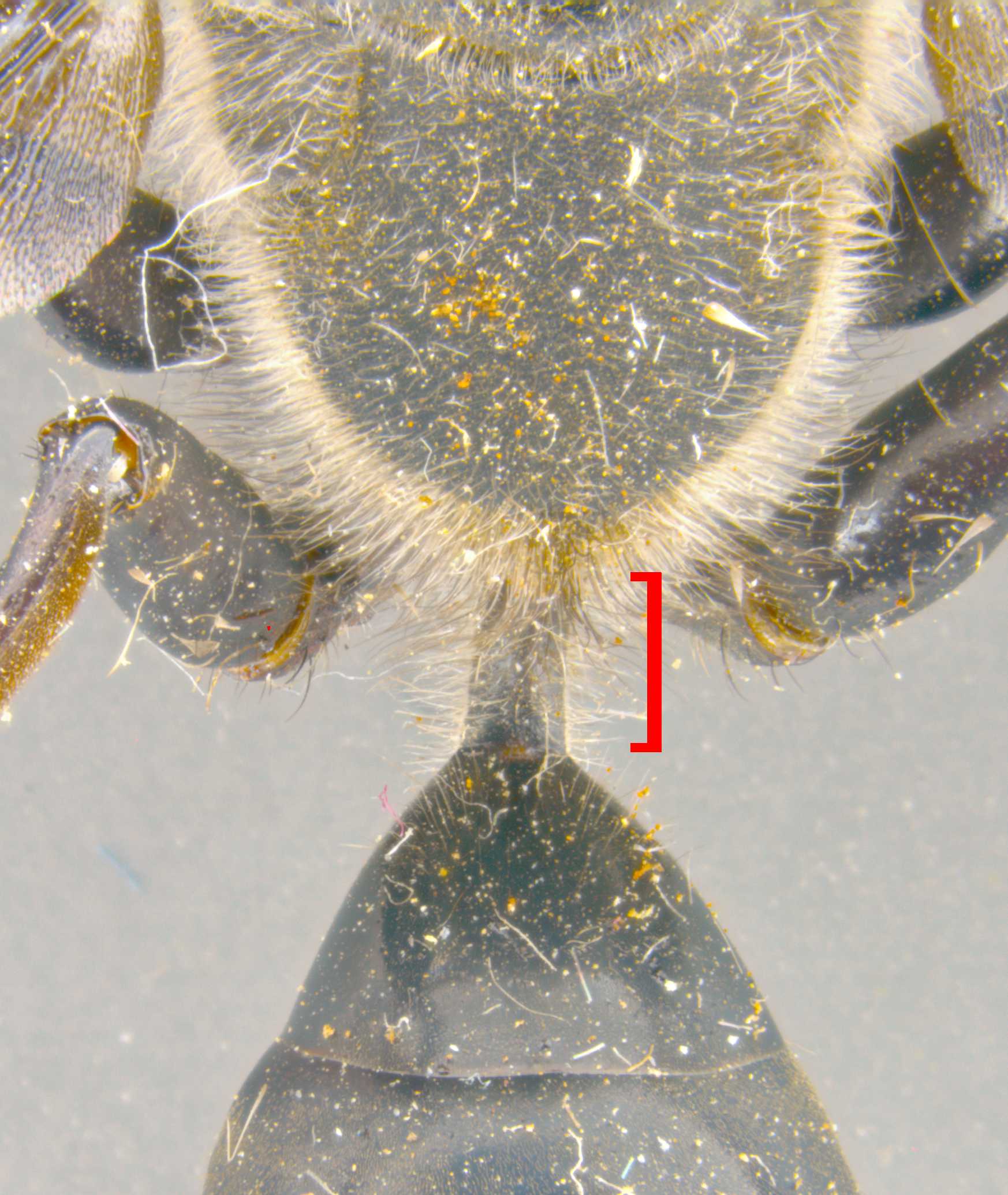

Supplement: Supplementary material 1 — Species data for genus Sphex [file zookeys-521-001-s001.zip › SphexDeltaFiles/Images/how_to_measure_petiole_length.jpg]

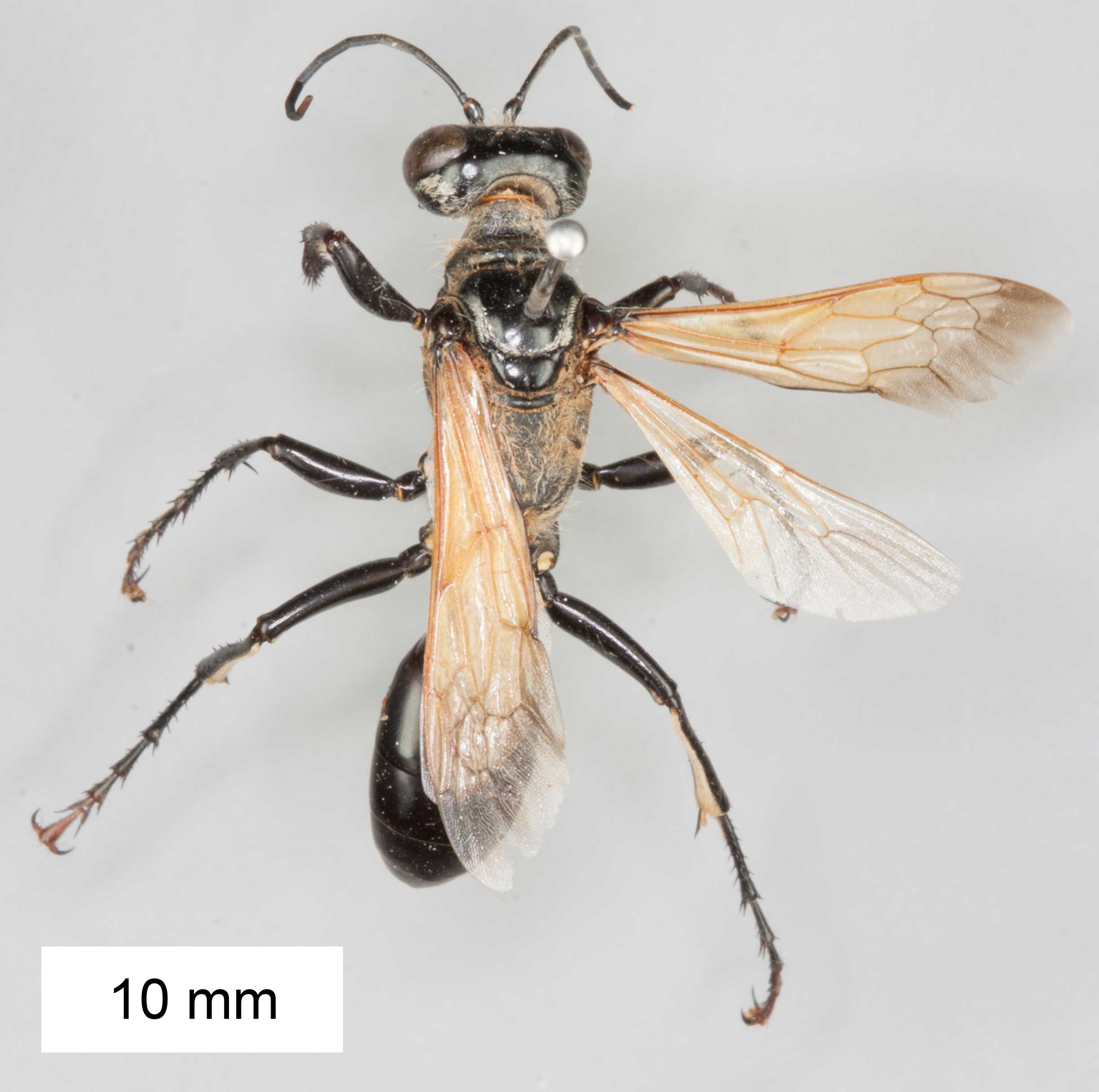

Supplement: Supplementary material 1 — Species data for genus Sphex [file zookeys-521-001-s001.zip › SphexDeltaFiles/Images/imporcatus_f.jpg]

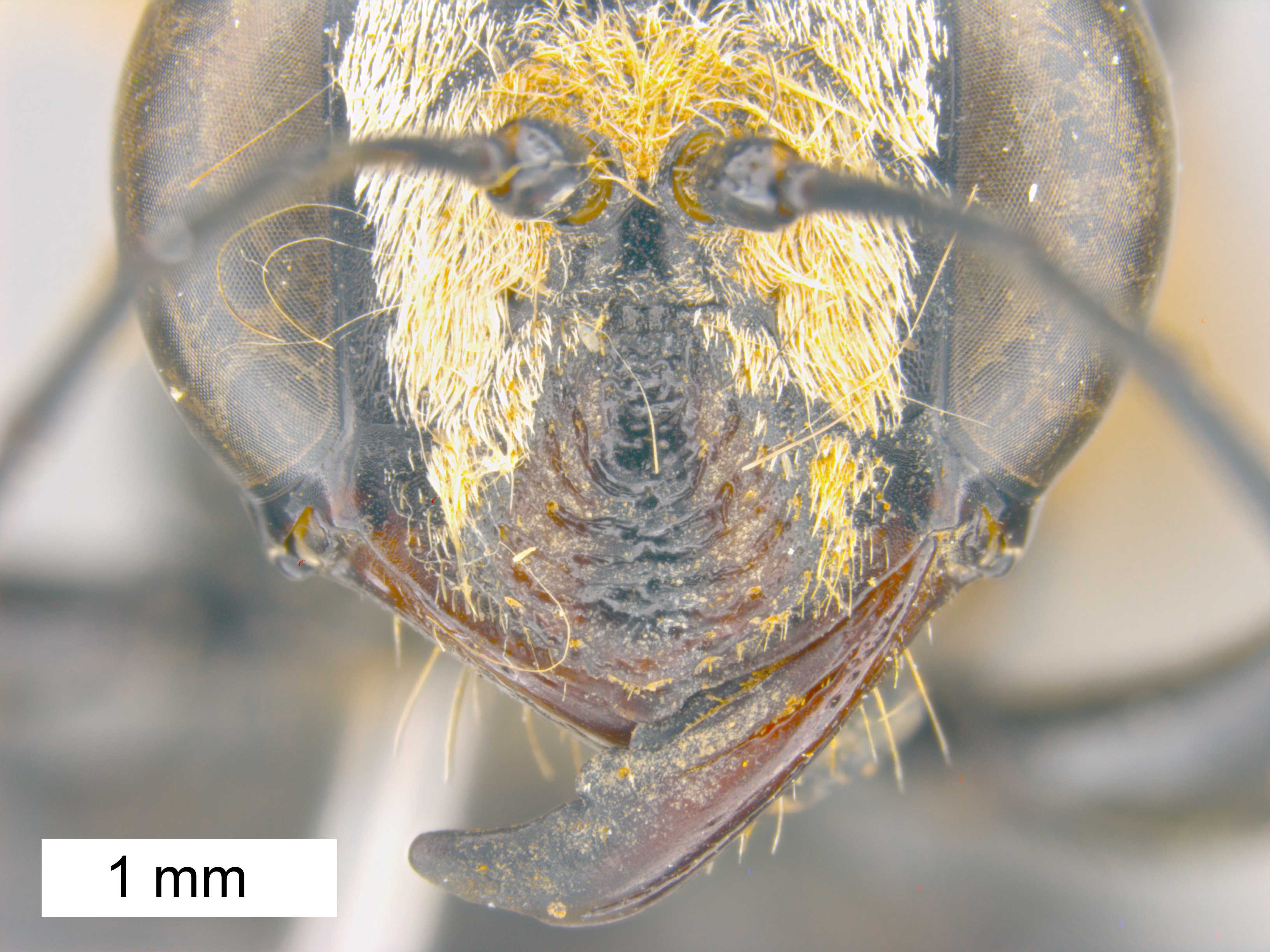

Supplement: Supplementary material 1 — Species data for genus Sphex [file zookeys-521-001-s001.zip › SphexDeltaFiles/Images/imporcatus_f_clypeus.jpg]

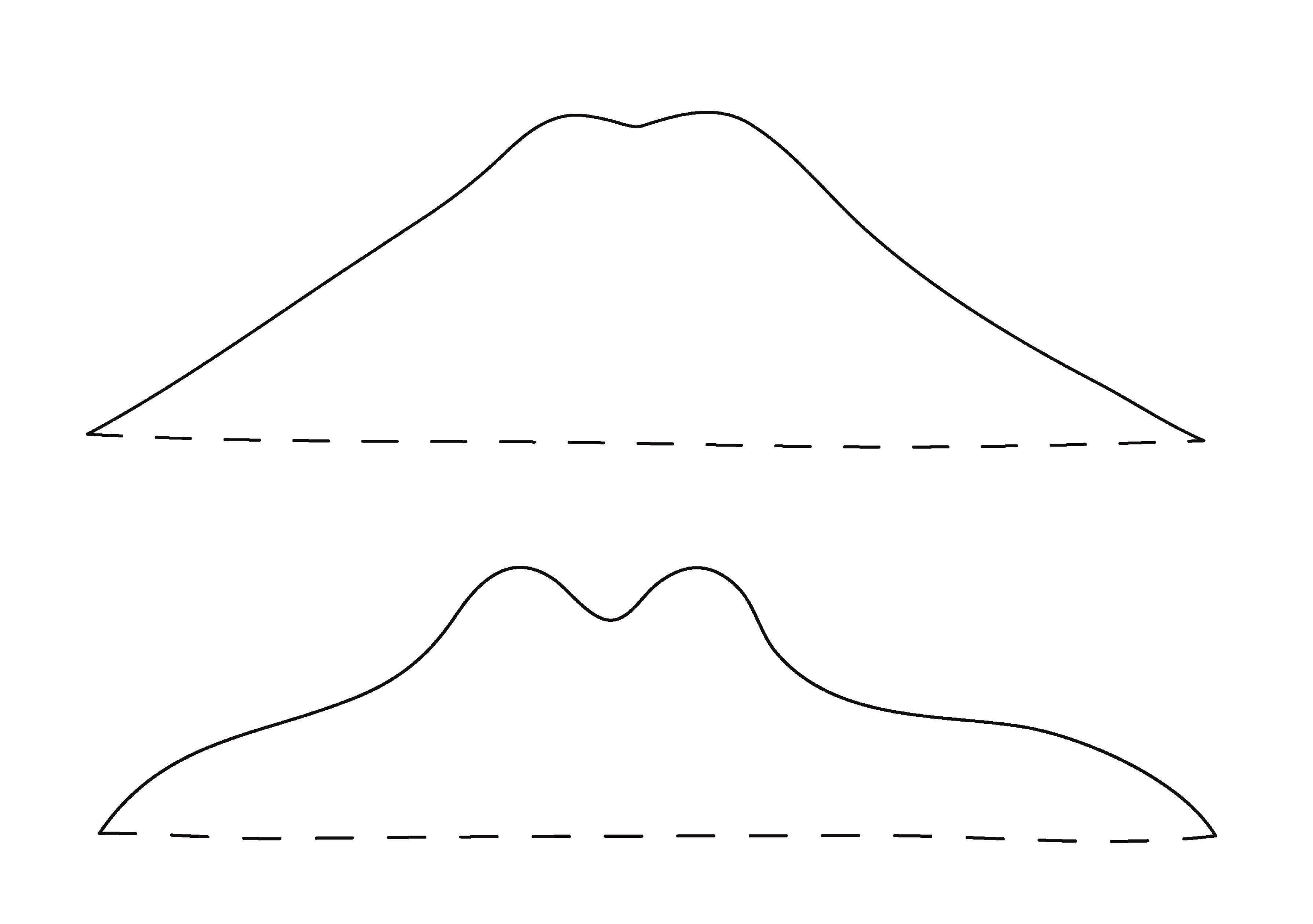

Supplement: Supplementary material 1 — Species data for genus Sphex [file zookeys-521-001-s001.zip › SphexDeltaFiles/Images/impression_on_metanotum.jpg]

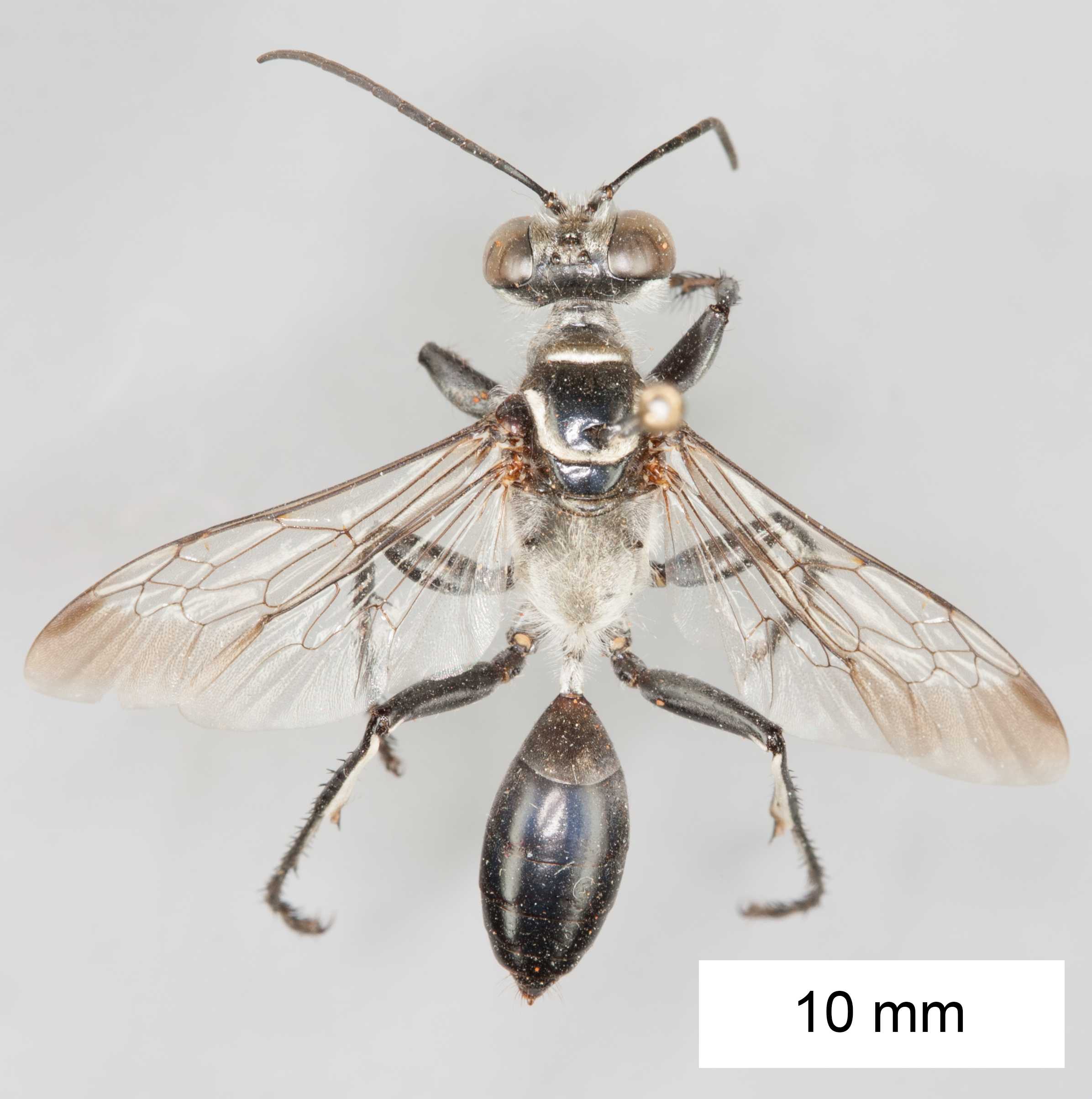

Supplement: Supplementary material 1 — Species data for genus Sphex [file zookeys-521-001-s001.zip › SphexDeltaFiles/Images/jucundus_f.jpg]

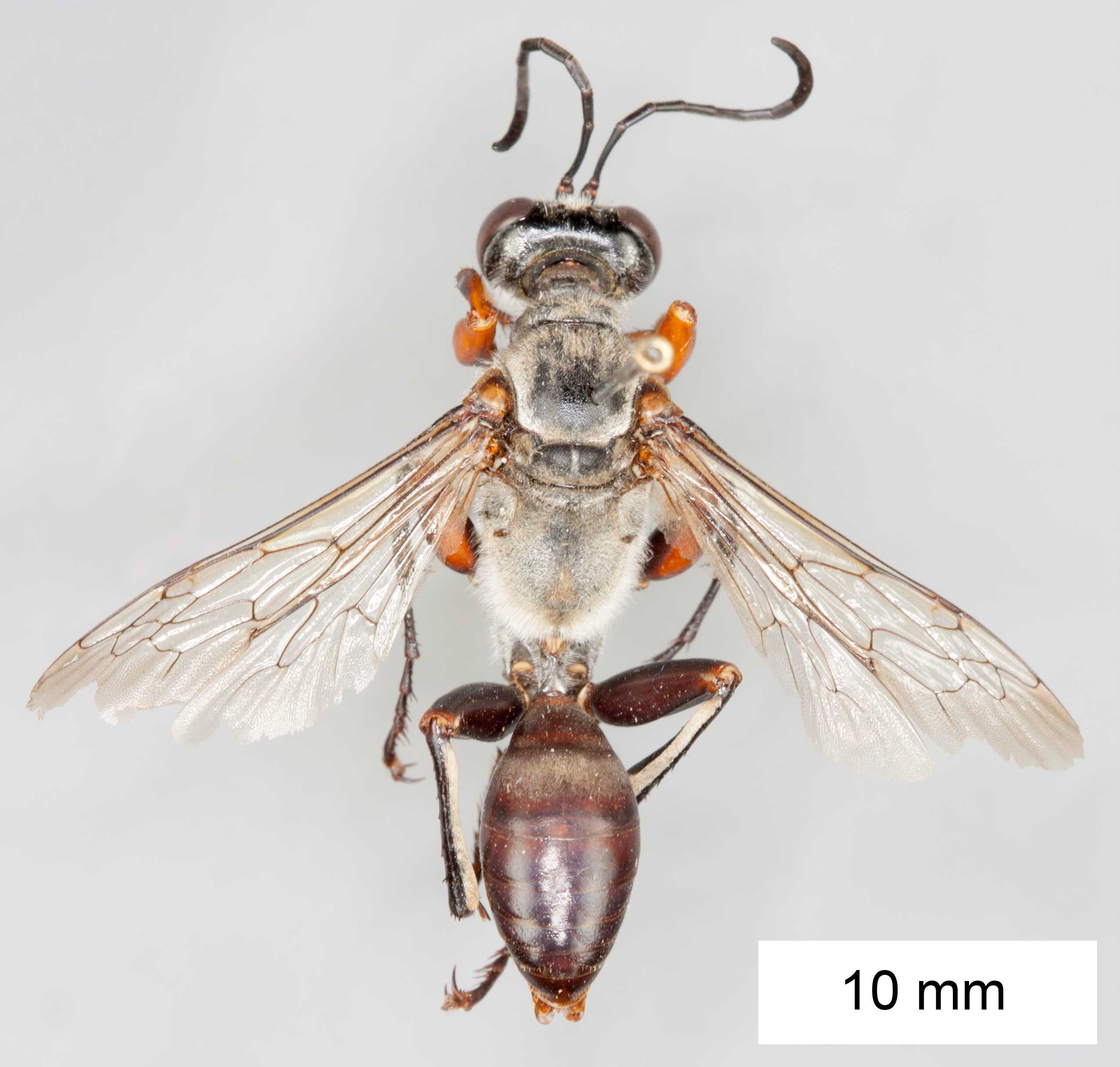

Supplement: Supplementary material 1 — Species data for genus Sphex [file zookeys-521-001-s001.zip › SphexDeltaFiles/Images/latilobus_m.jpg]

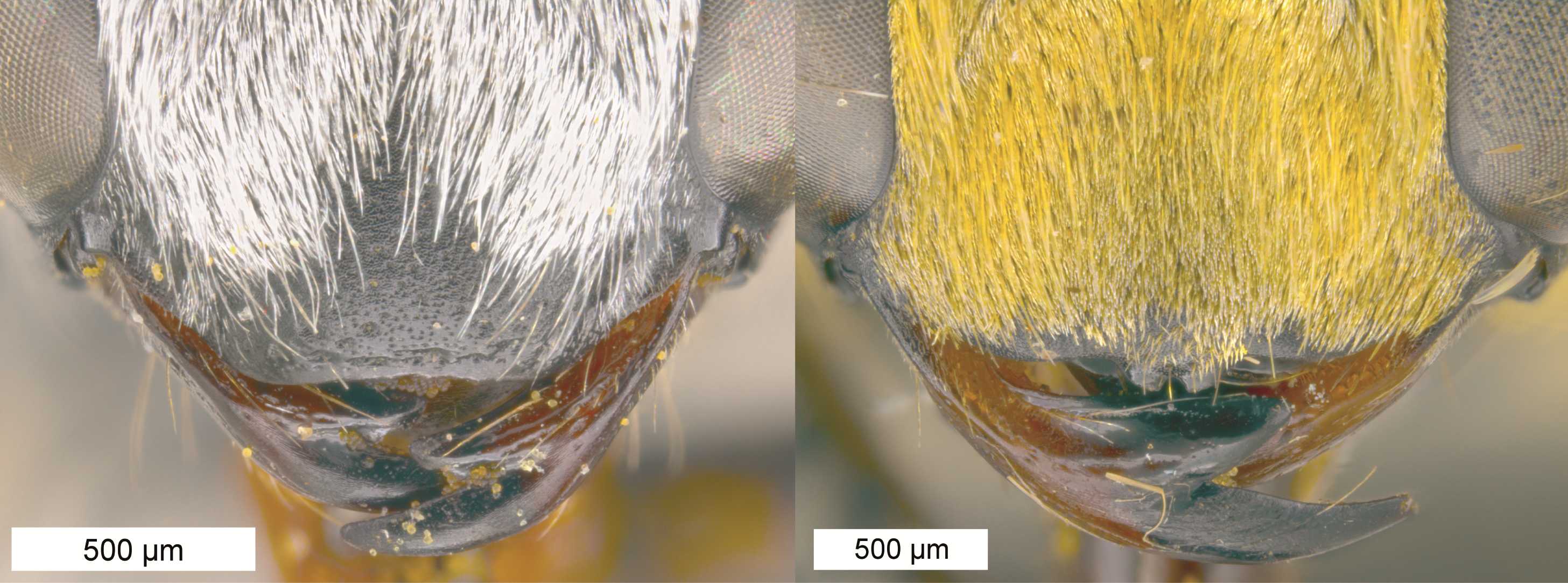

Supplement: Supplementary material 1 — Species data for genus Sphex [file zookeys-521-001-s001.zip › SphexDeltaFiles/Images/lobes_existing.jpg]

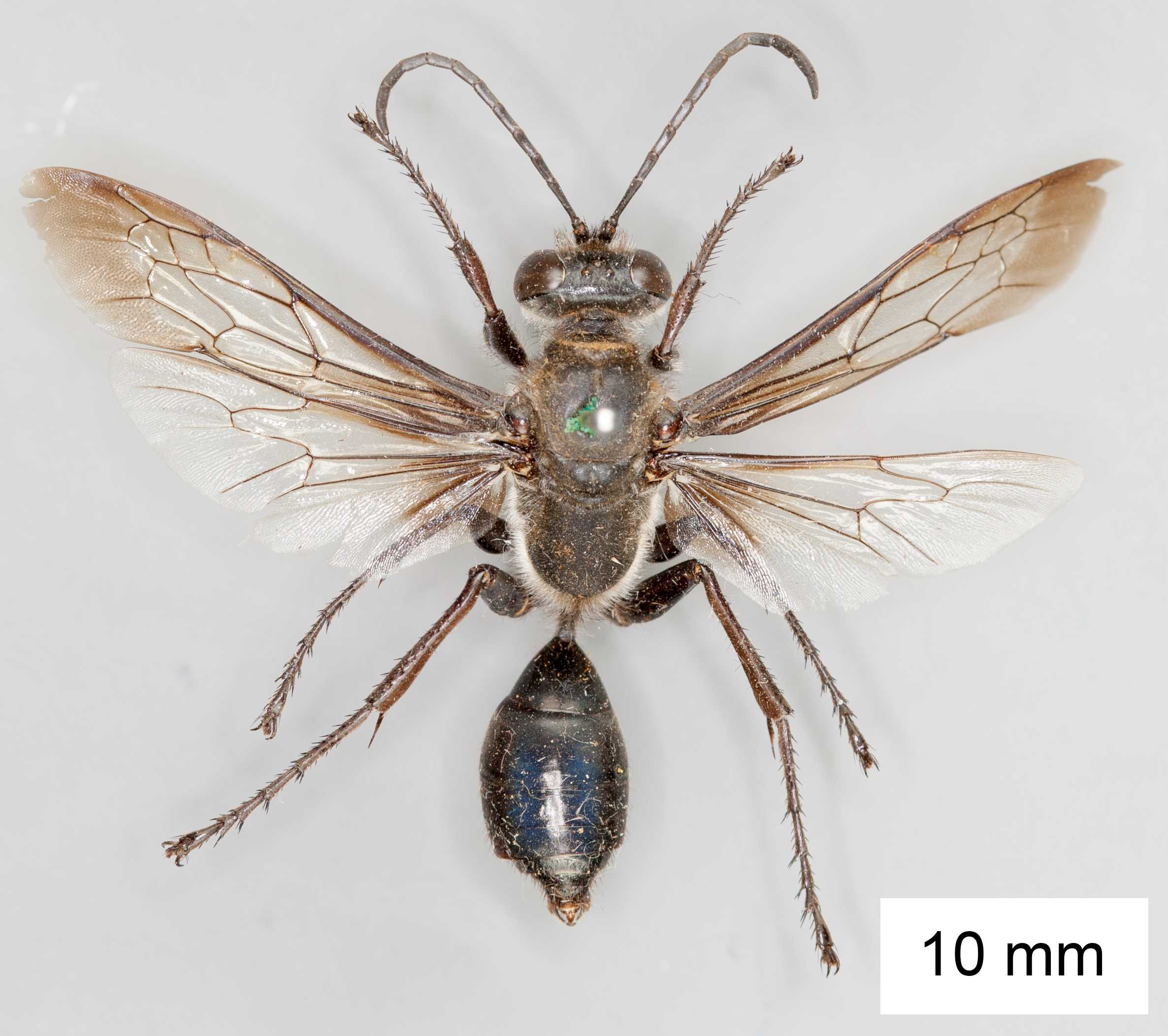

Supplement: Supplementary material 1 — Species data for genus Sphex [file zookeys-521-001-s001.zip › SphexDeltaFiles/Images/luctuosus_m.jpg]

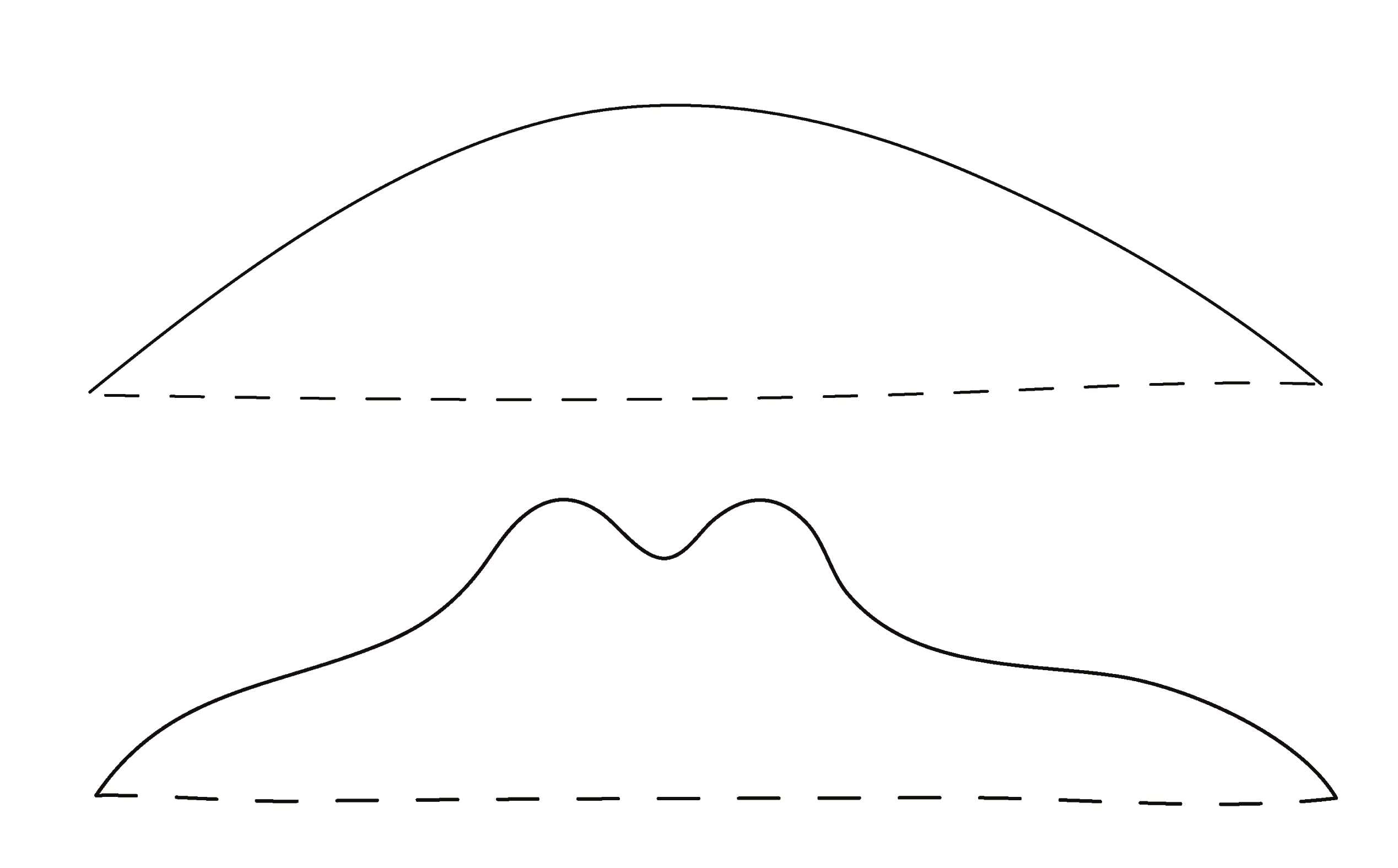

Supplement: Supplementary material 1 — Species data for genus Sphex [file zookeys-521-001-s001.zip › SphexDeltaFiles/Images/metanotum_flat_or_lobes.jpg]

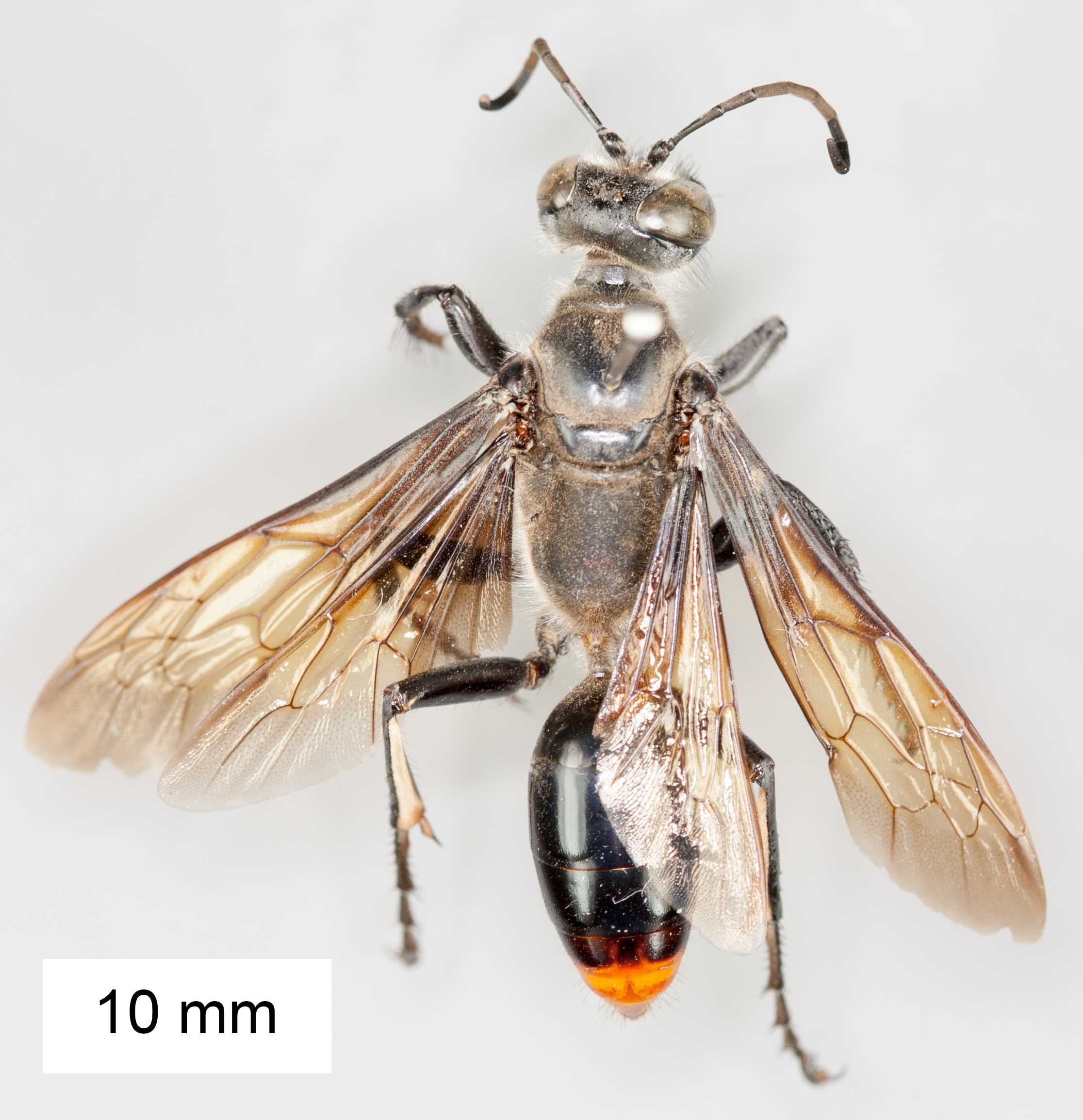

Supplement: Supplementary material 1 — Species data for genus Sphex [file zookeys-521-001-s001.zip › SphexDeltaFiles/Images/mimulus_f.jpg]

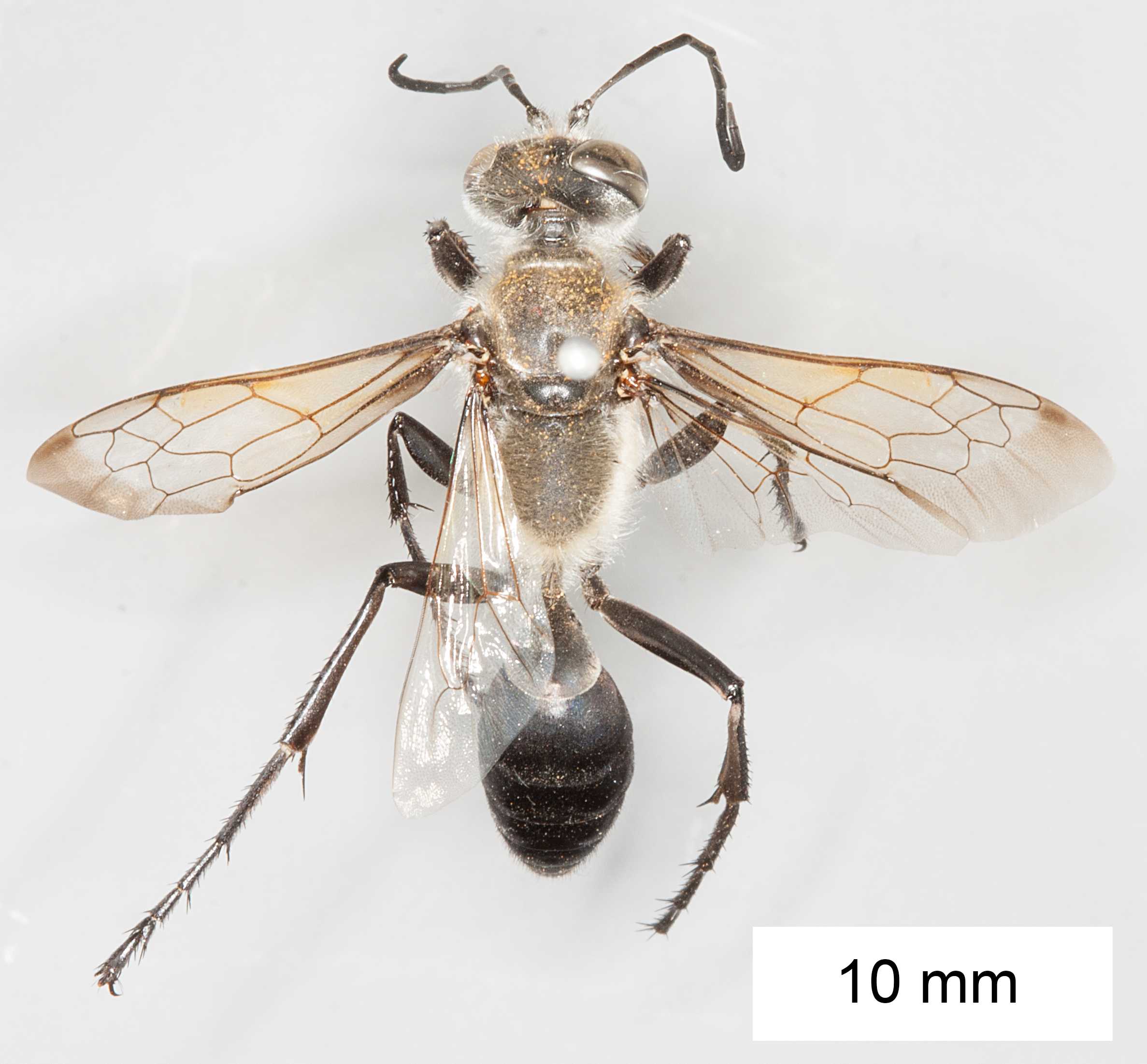

Supplement: Supplementary material 1 — Species data for genus Sphex [file zookeys-521-001-s001.zip › SphexDeltaFiles/Images/modestus_m1.jpg]

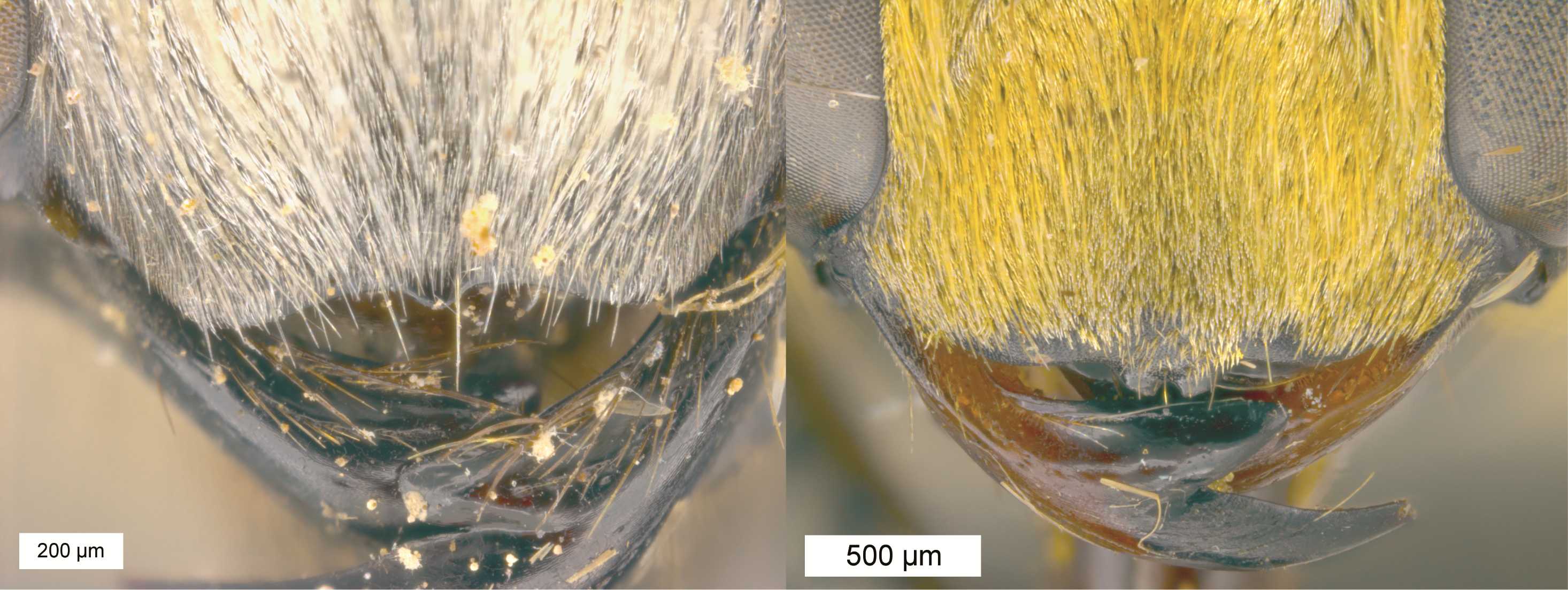

Supplement: Supplementary material 1 — Species data for genus Sphex [file zookeys-521-001-s001.zip › SphexDeltaFiles/Images/number_lobes.jpg]

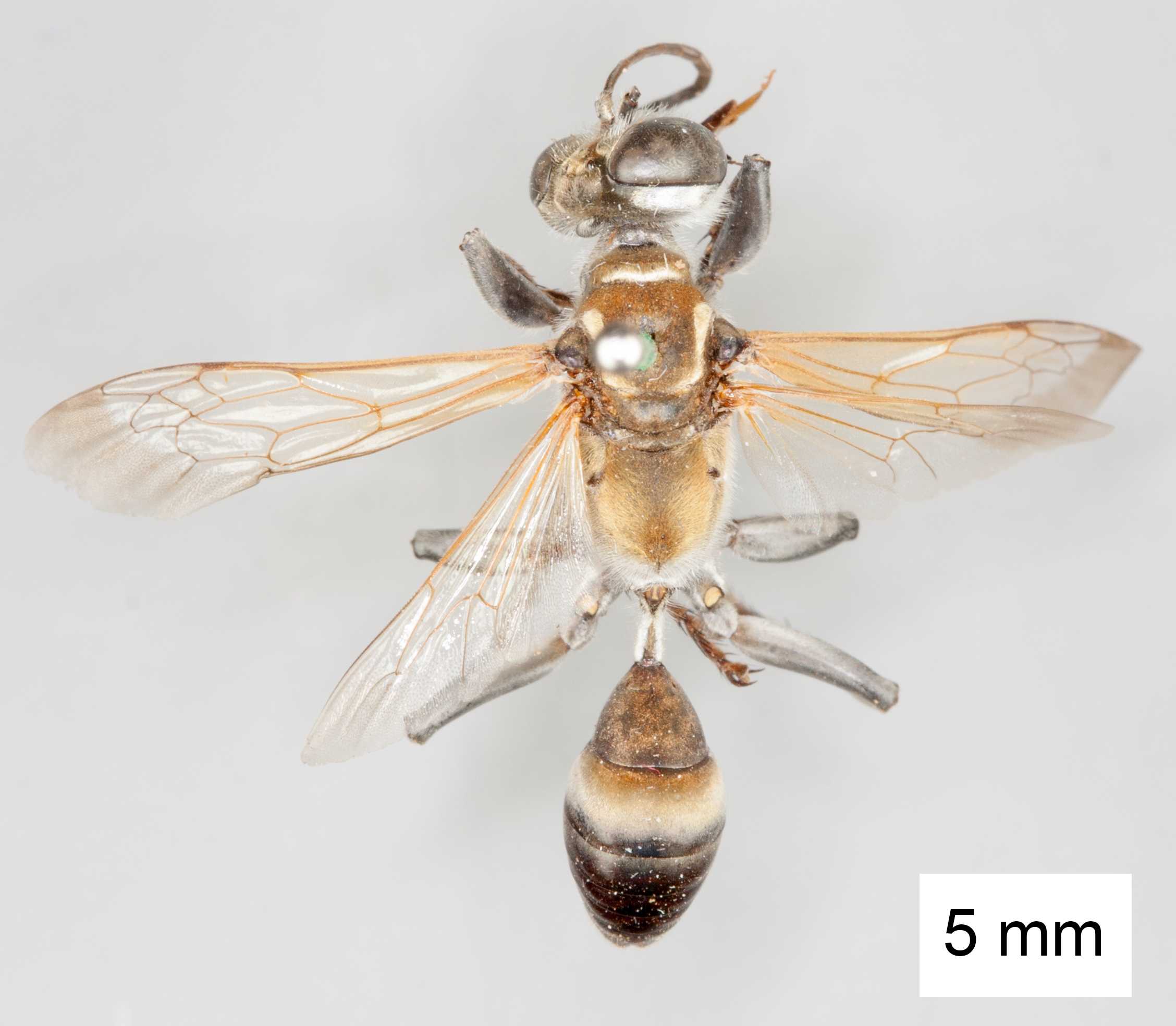

Supplement: Supplementary material 1 — Species data for genus Sphex [file zookeys-521-001-s001.zip › SphexDeltaFiles/Images/pretiosus_m.jpg]

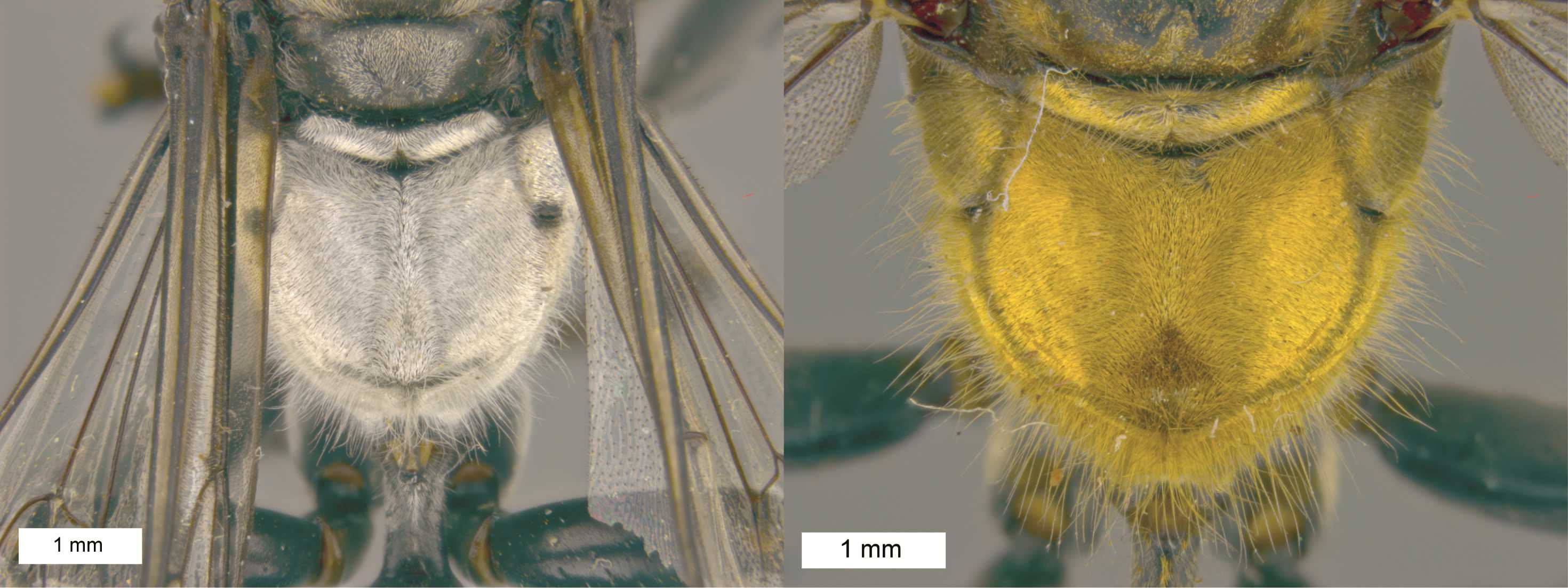

Supplement: Supplementary material 1 — Species data for genus Sphex [file zookeys-521-001-s001.zip › SphexDeltaFiles/Images/propodeal_pubescence_color.jpg]

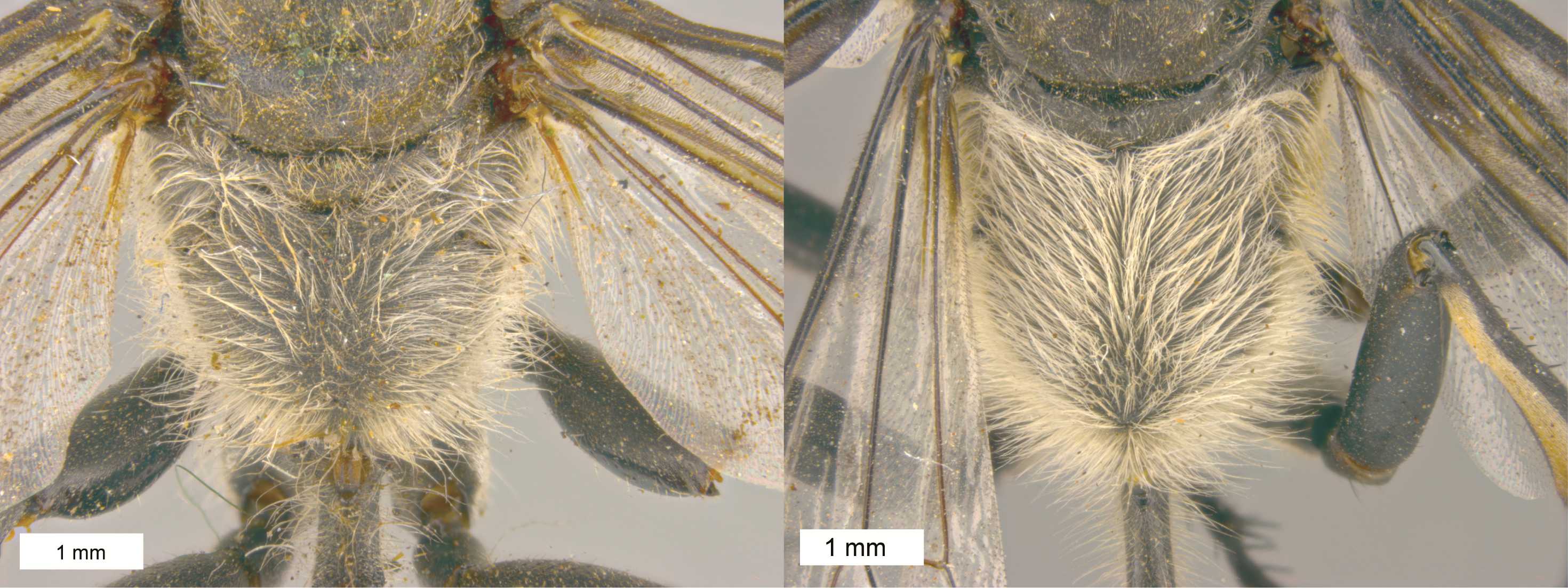

Supplement: Supplementary material 1 — Species data for genus Sphex [file zookeys-521-001-s001.zip › SphexDeltaFiles/Images/propodeal_pubescence_density.jpg]

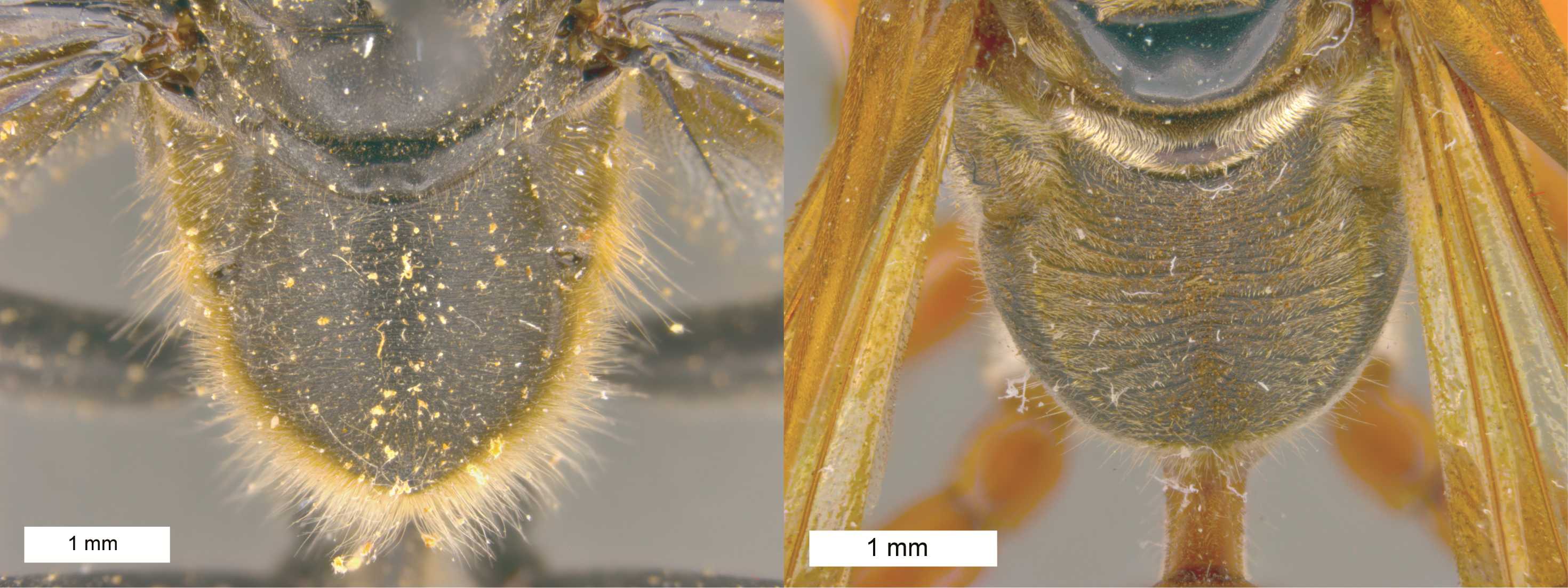

Supplement: Supplementary material 1 — Species data for genus Sphex [file zookeys-521-001-s001.zip › SphexDeltaFiles/Images/propodeal_sculpture.jpg]

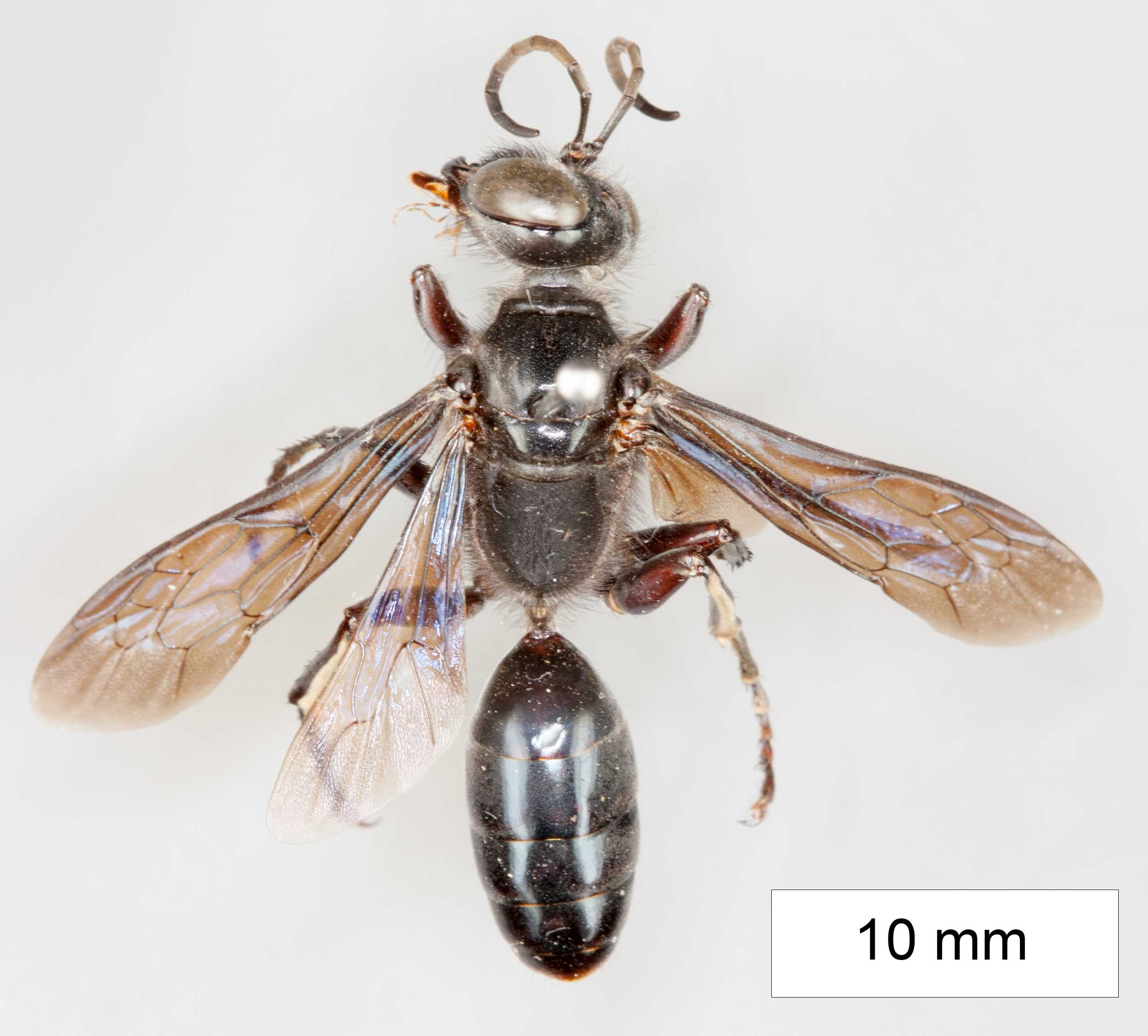

Supplement: Supplementary material 1 — Species data for genus Sphex [file zookeys-521-001-s001.zip › SphexDeltaFiles/Images/resplendens_f.jpg]

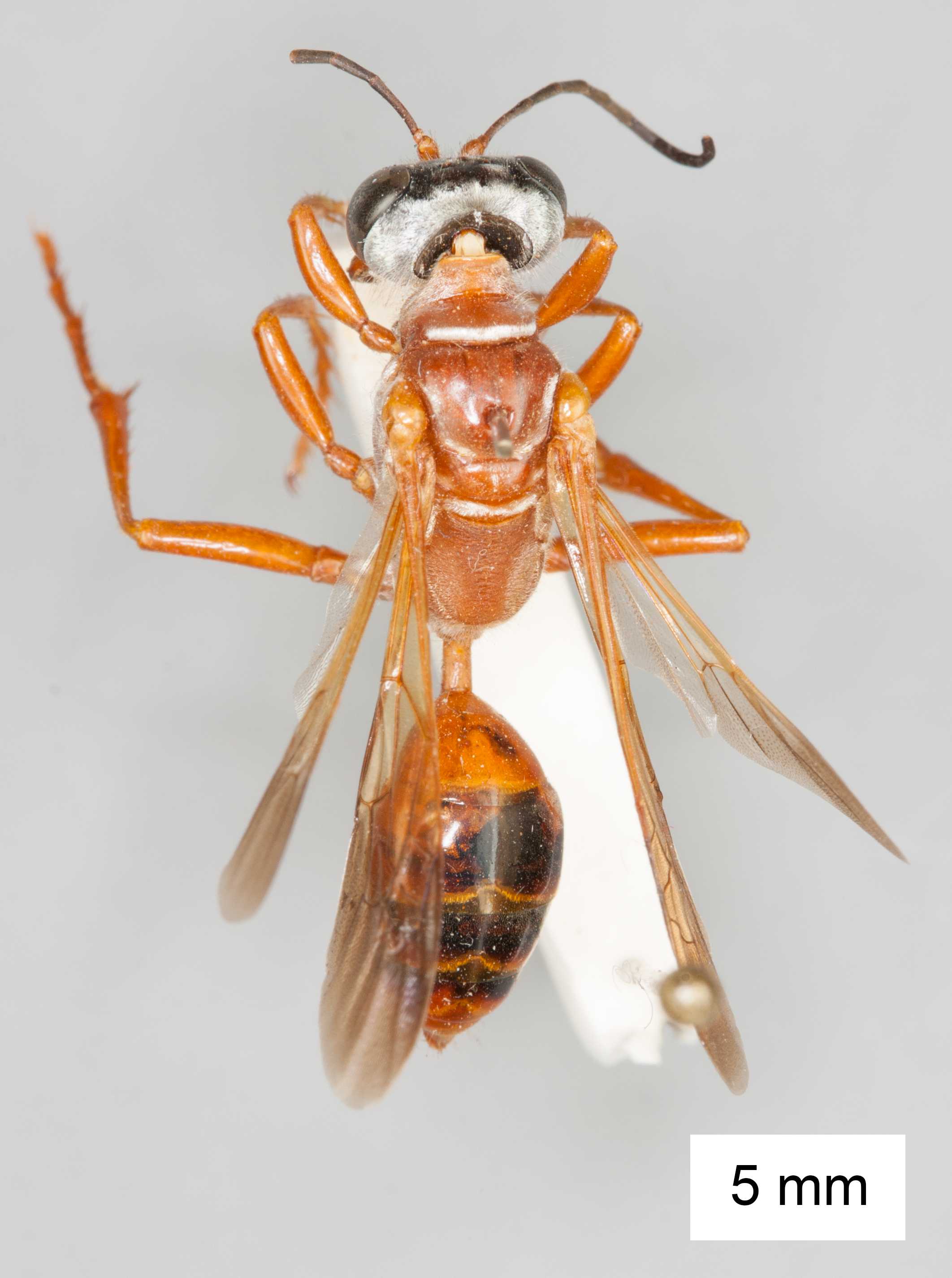

Supplement: Supplementary material 1 — Species data for genus Sphex [file zookeys-521-001-s001.zip › SphexDeltaFiles/Images/rhodosoma_f.jpg]

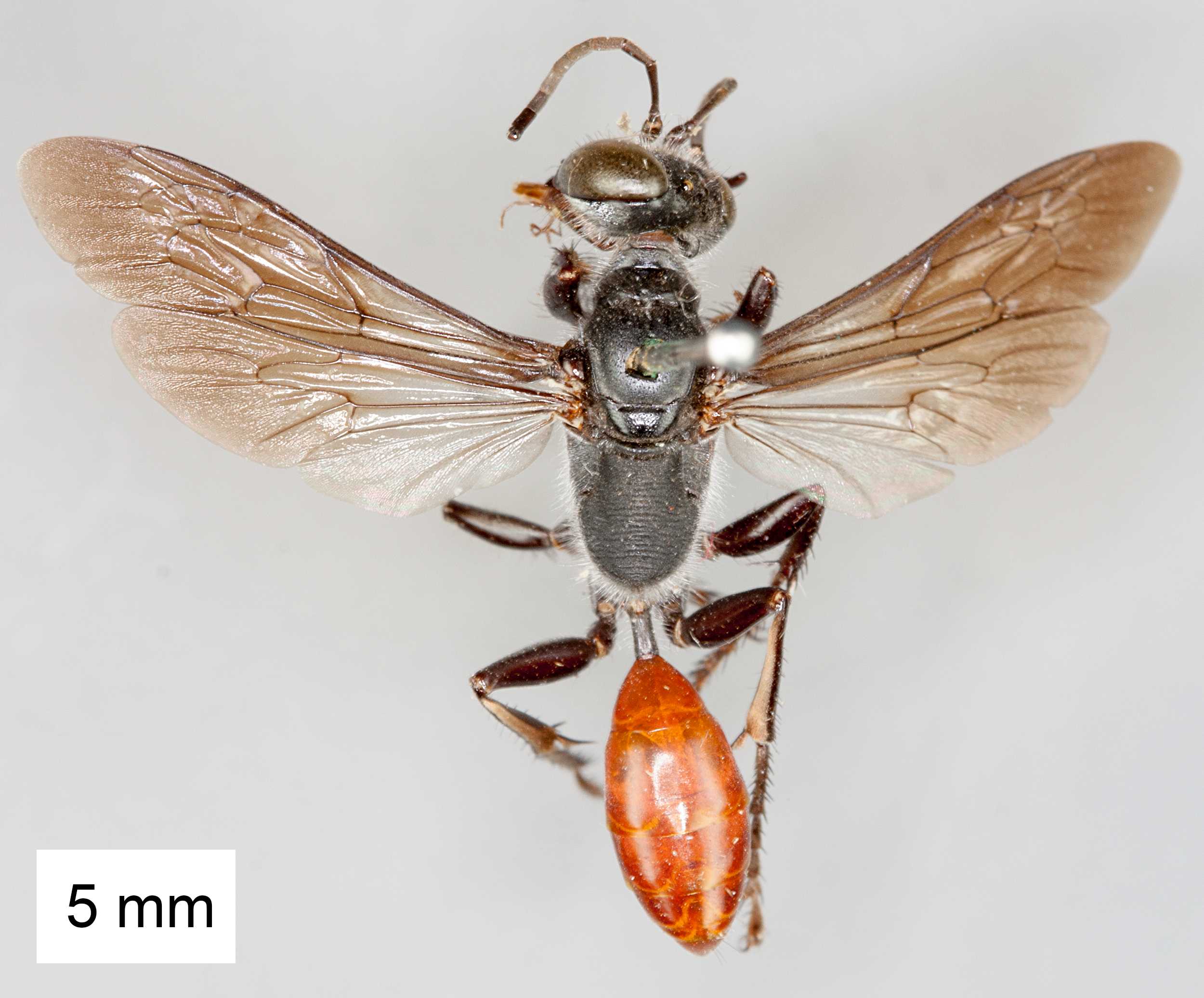

Supplement: Supplementary material 1 — Species data for genus Sphex [file zookeys-521-001-s001.zip › SphexDeltaFiles/Images/rugifer_f.jpg]

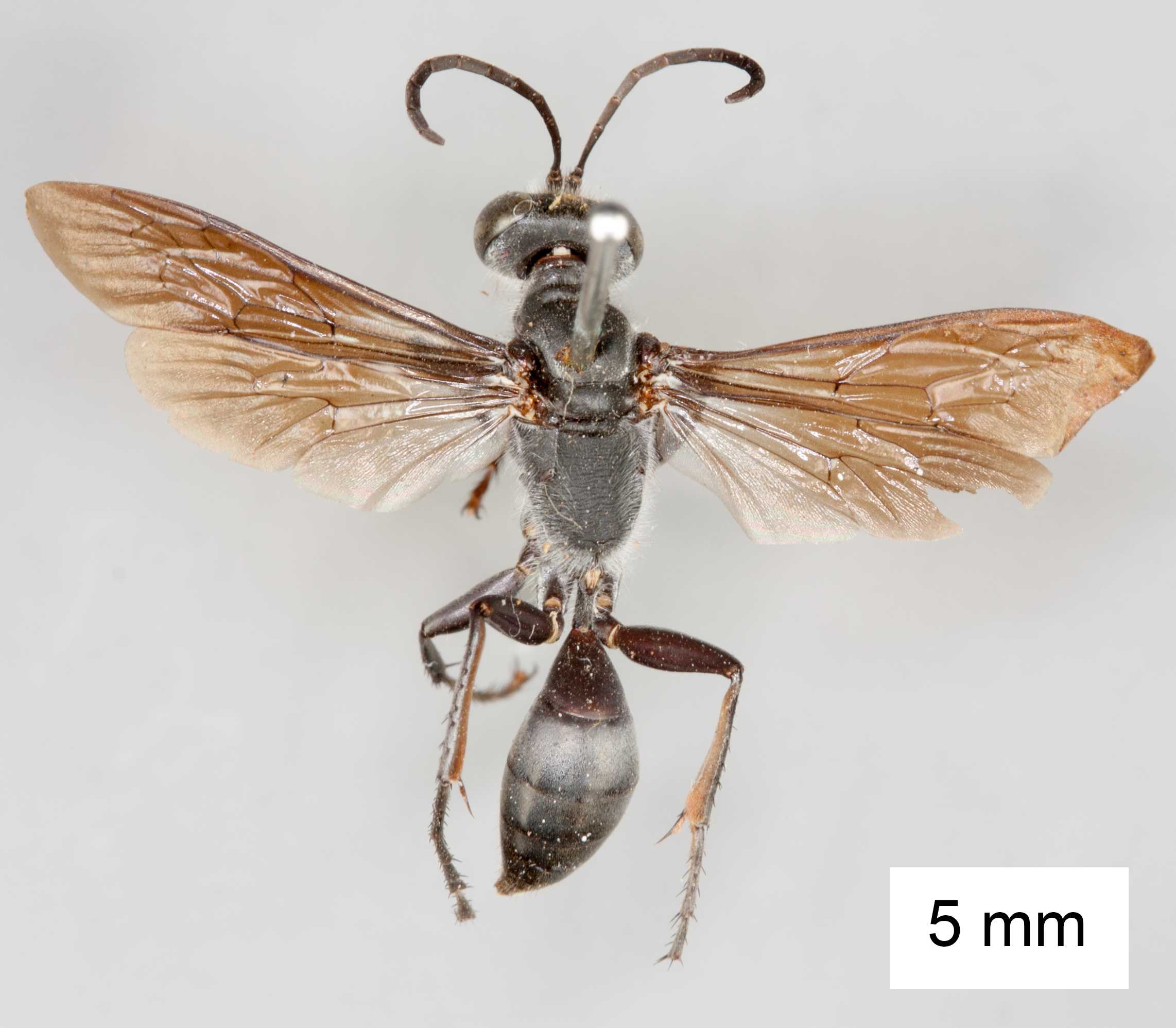

Supplement: Supplementary material 1 — Species data for genus Sphex [file zookeys-521-001-s001.zip › SphexDeltaFiles/Images/rugifer_m.jpg]

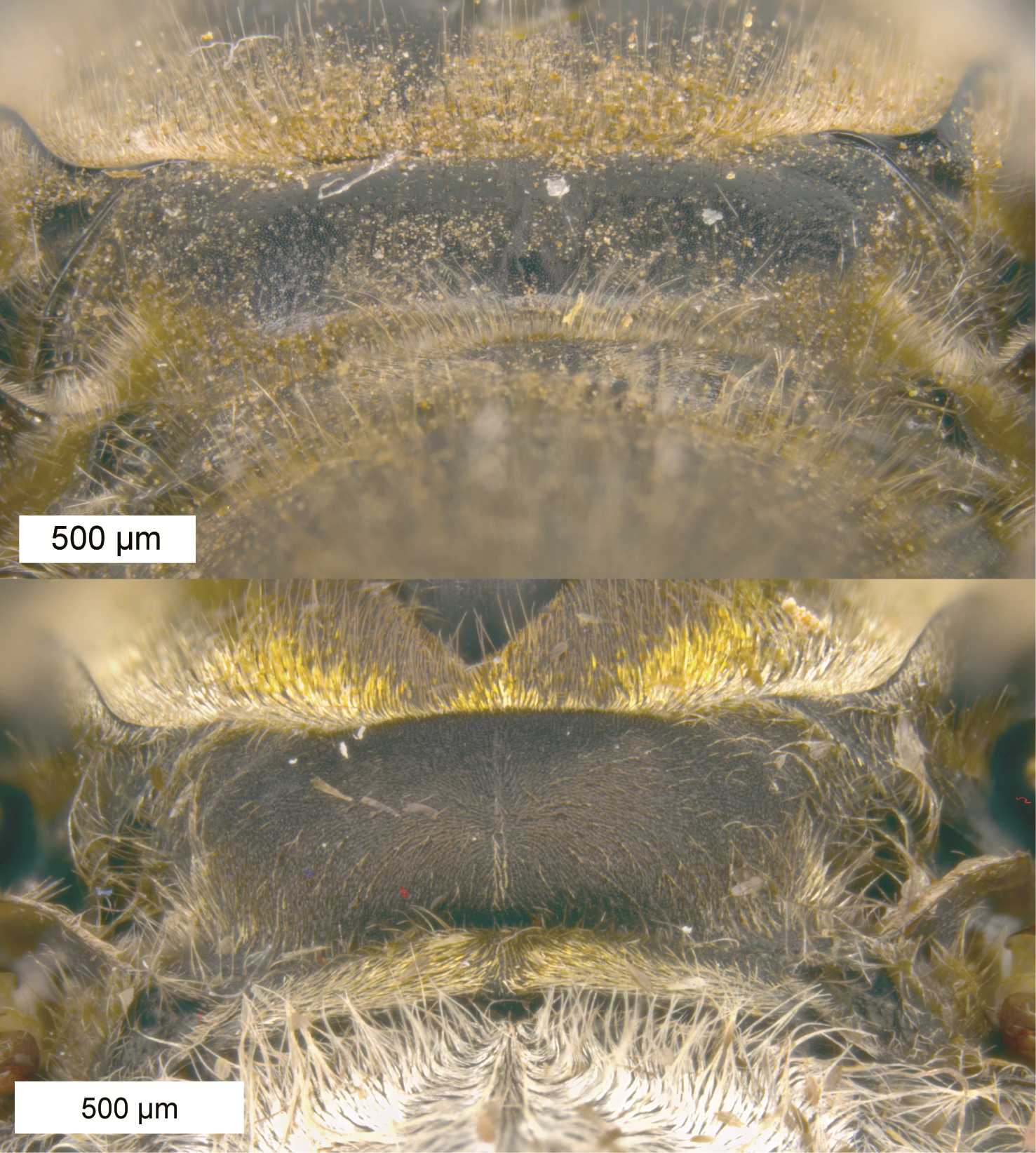

Supplement: Supplementary material 1 — Species data for genus Sphex [file zookeys-521-001-s001.zip › SphexDeltaFiles/Images/scutellum_flatness.jpg]

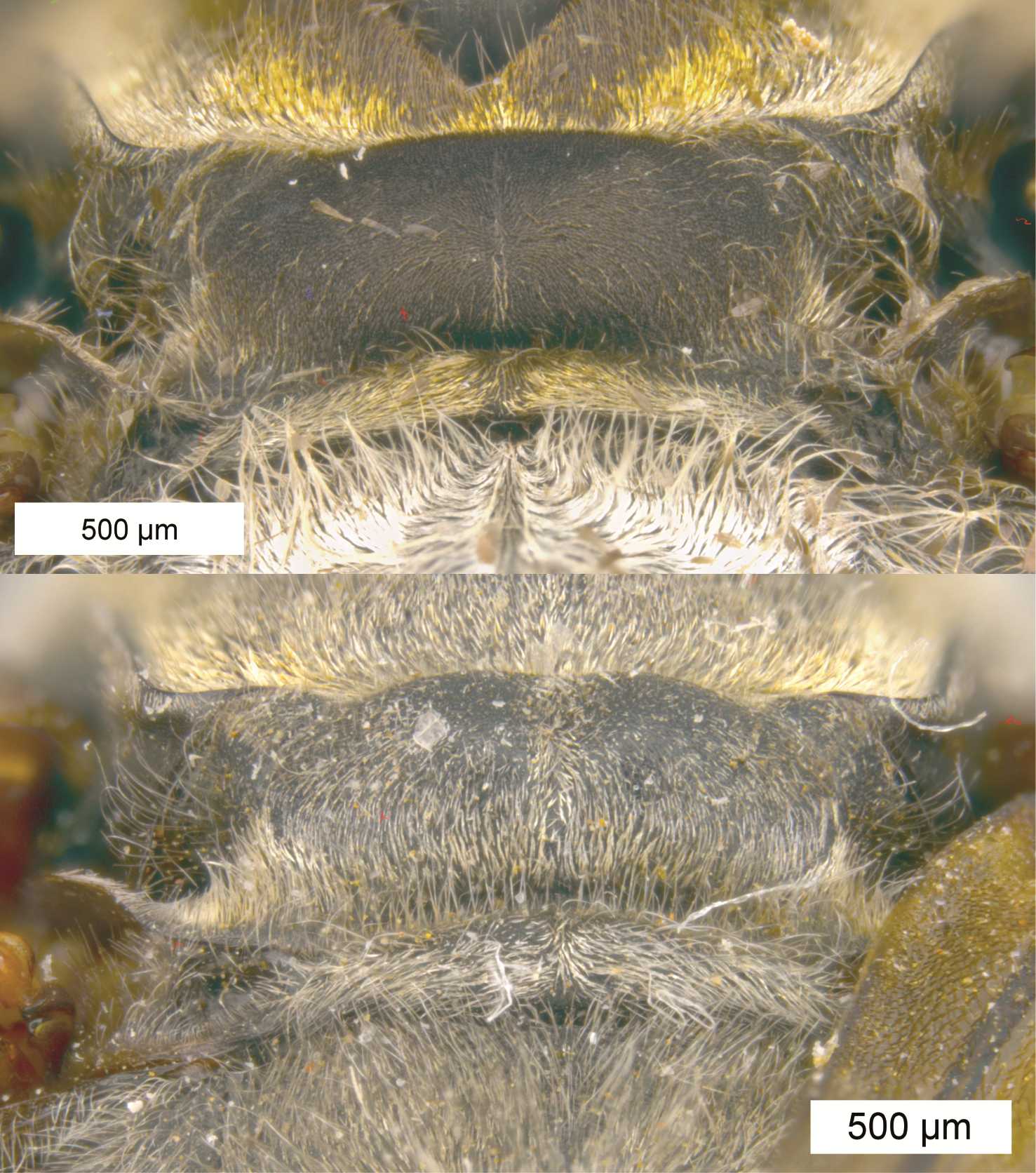

Supplement: Supplementary material 1 — Species data for genus Sphex [file zookeys-521-001-s001.zip › SphexDeltaFiles/Images/scutellum_impression.jpg]

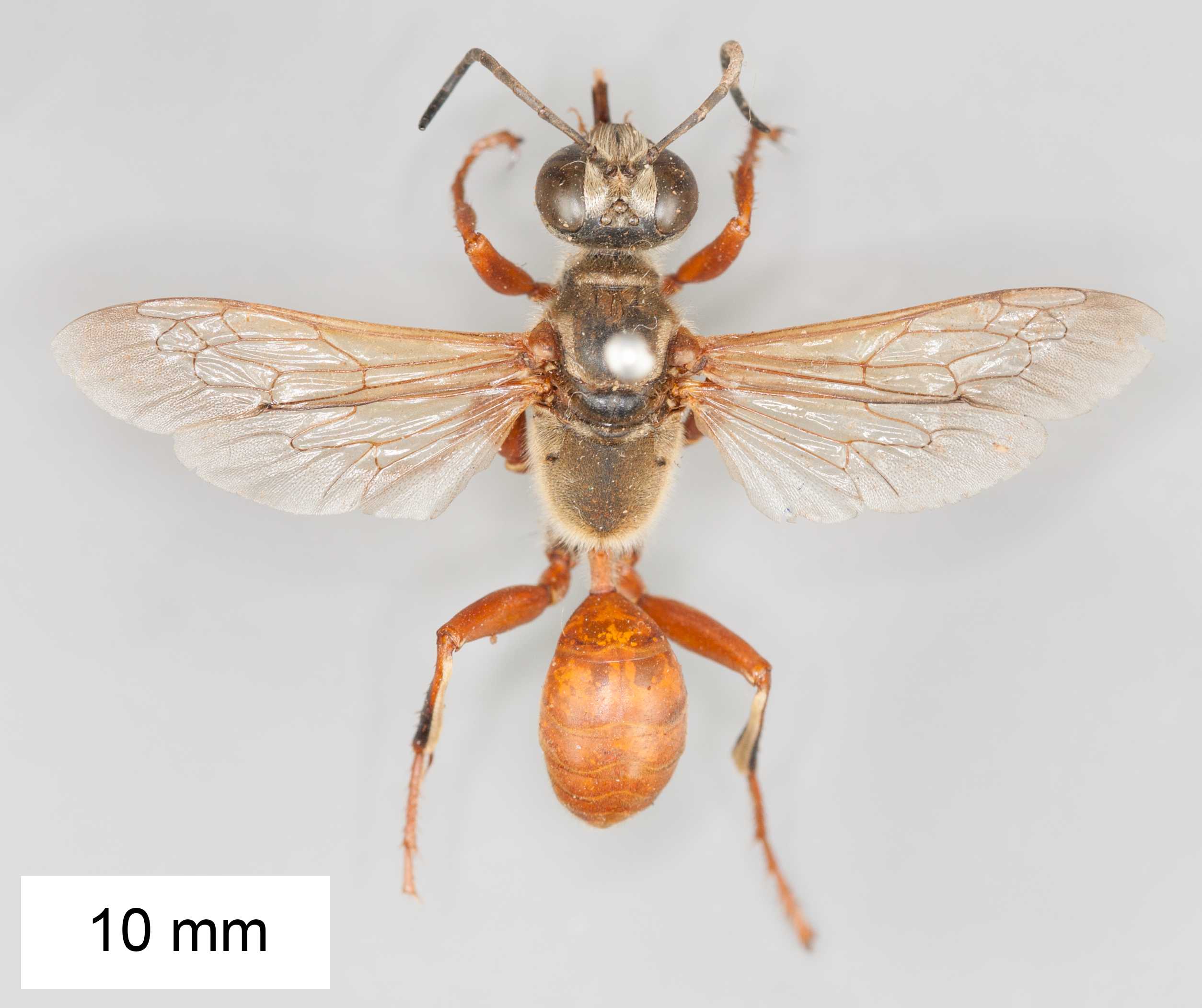

Supplement: Supplementary material 1 — Species data for genus Sphex [file zookeys-521-001-s001.zip › SphexDeltaFiles/Images/semifossulatus_m.jpg]

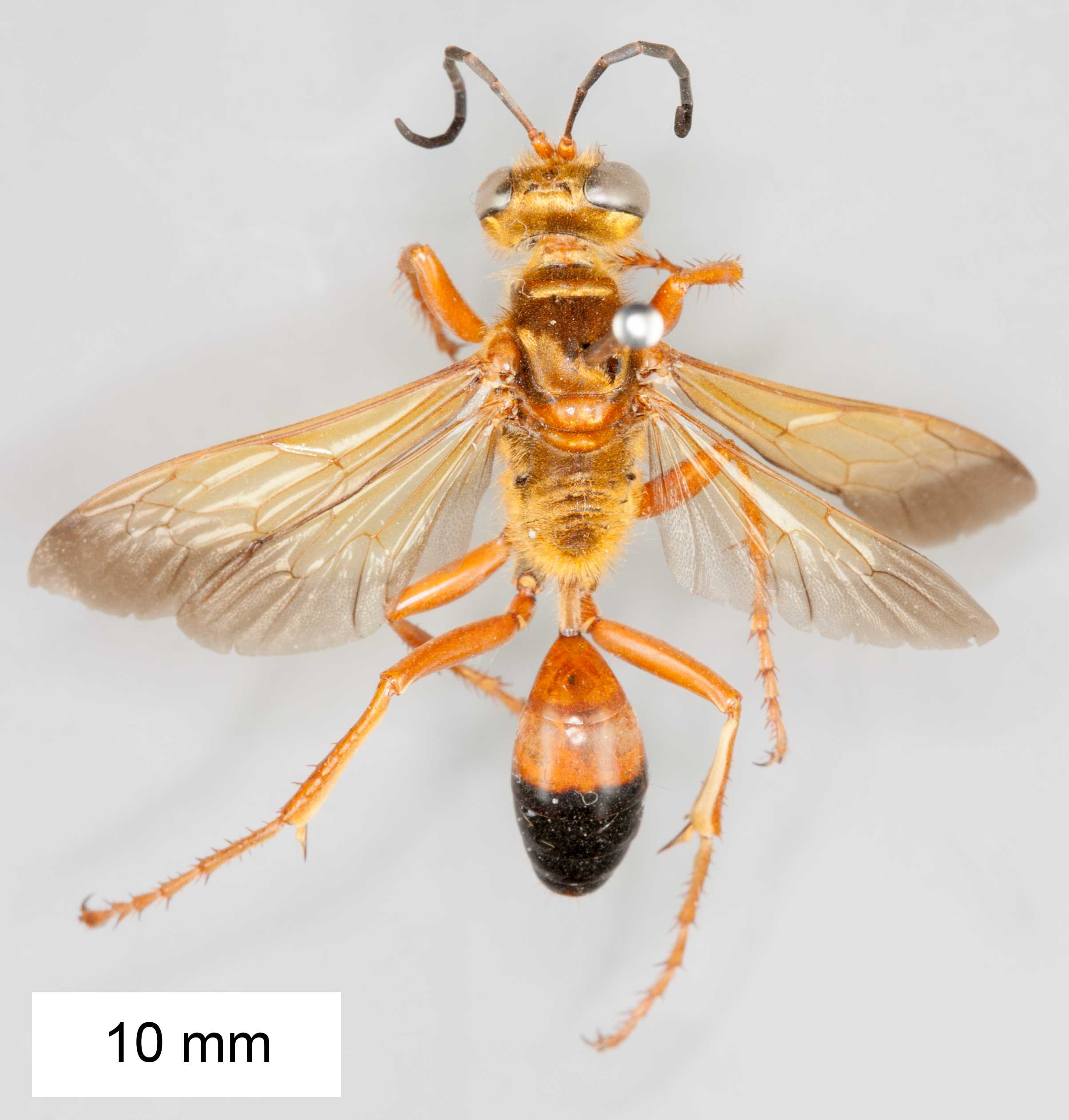

Supplement: Supplementary material 1 — Species data for genus Sphex [file zookeys-521-001-s001.zip › SphexDeltaFiles/Images/sericeus_f.jpg]

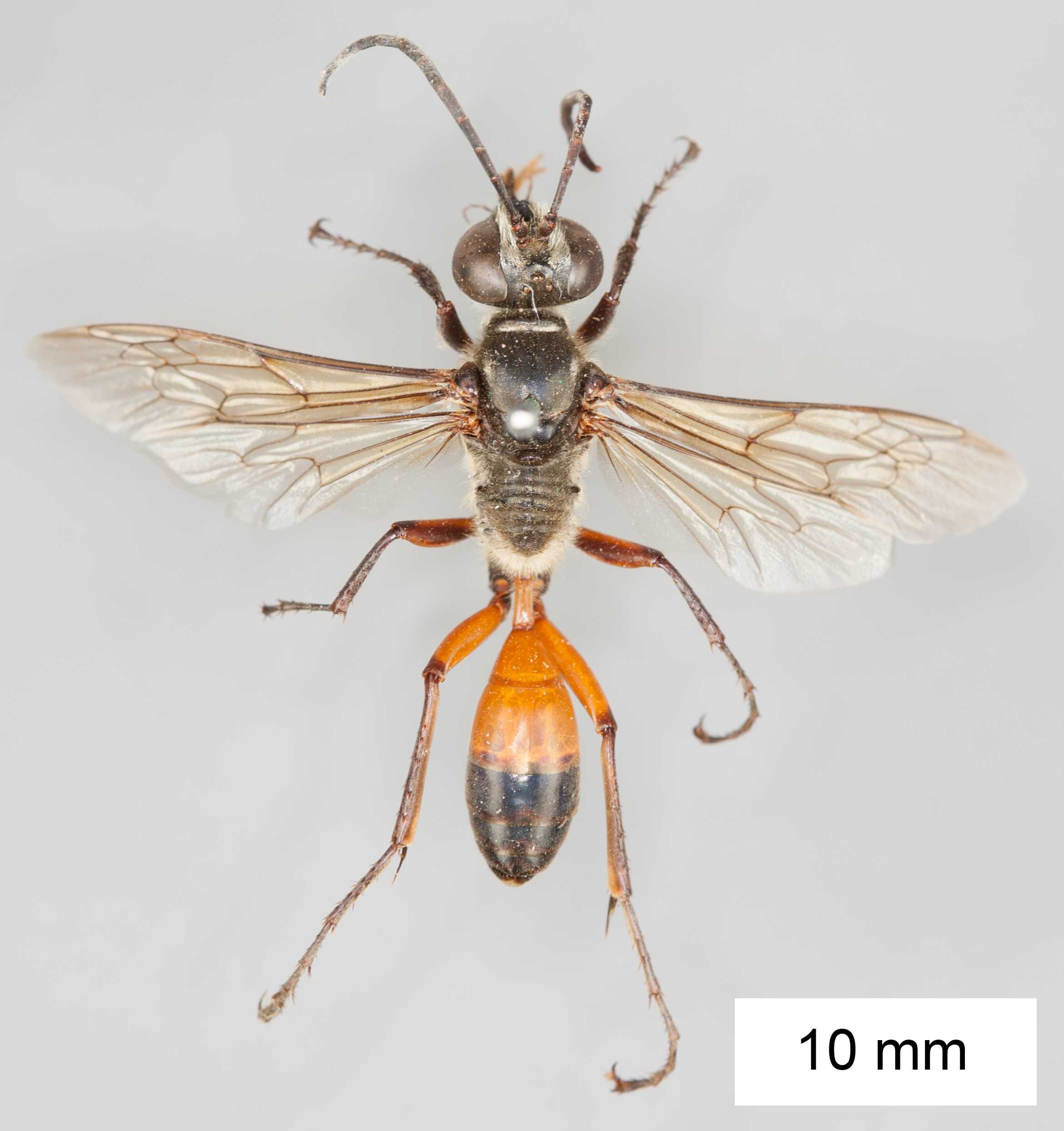

Supplement: Supplementary material 1 — Species data for genus Sphex [file zookeys-521-001-s001.zip › SphexDeltaFiles/Images/sericeus_m_Darwin.jpg]

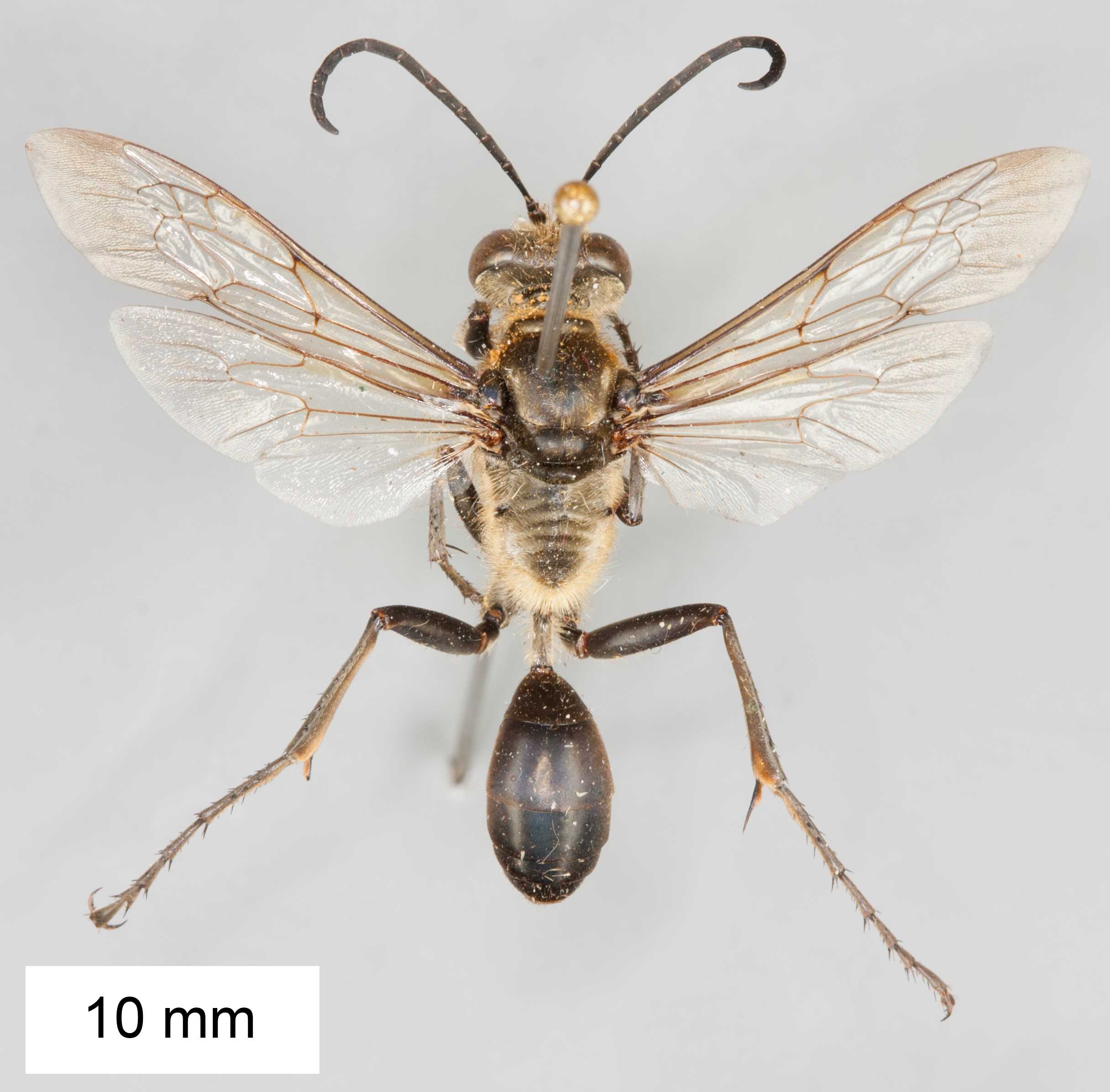

Supplement: Supplementary material 1 — Species data for genus Sphex [file zookeys-521-001-s001.zip › SphexDeltaFiles/Images/sericeus_m_westwood_QLD.jpg]

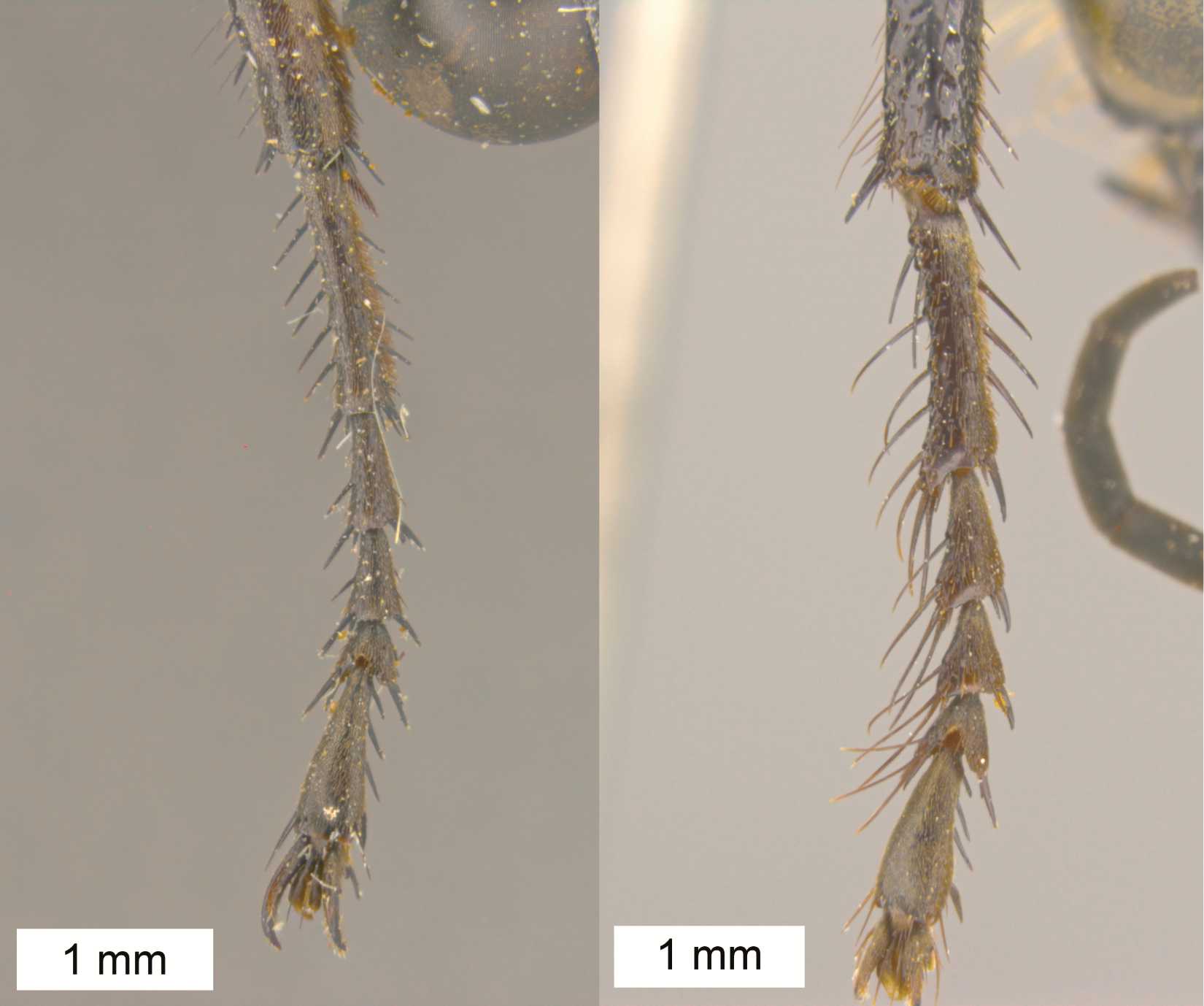

Supplement: Supplementary material 1 — Species data for genus Sphex [file zookeys-521-001-s001.zip › SphexDeltaFiles/Images/sex.jpg]

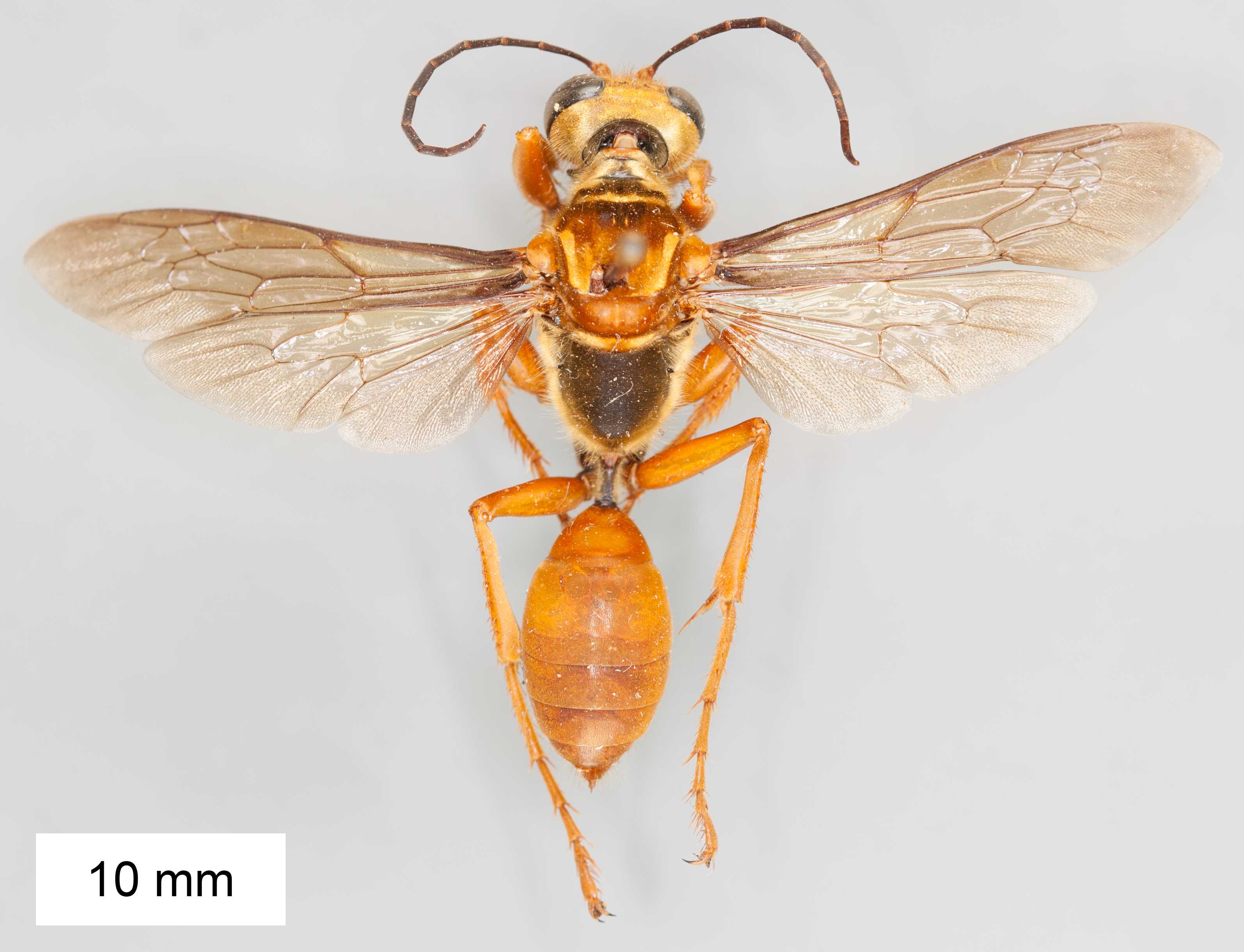

Supplement: Supplementary material 1 — Species data for genus Sphex [file zookeys-521-001-s001.zip › SphexDeltaFiles/Images/staudingeri_f.jpg]

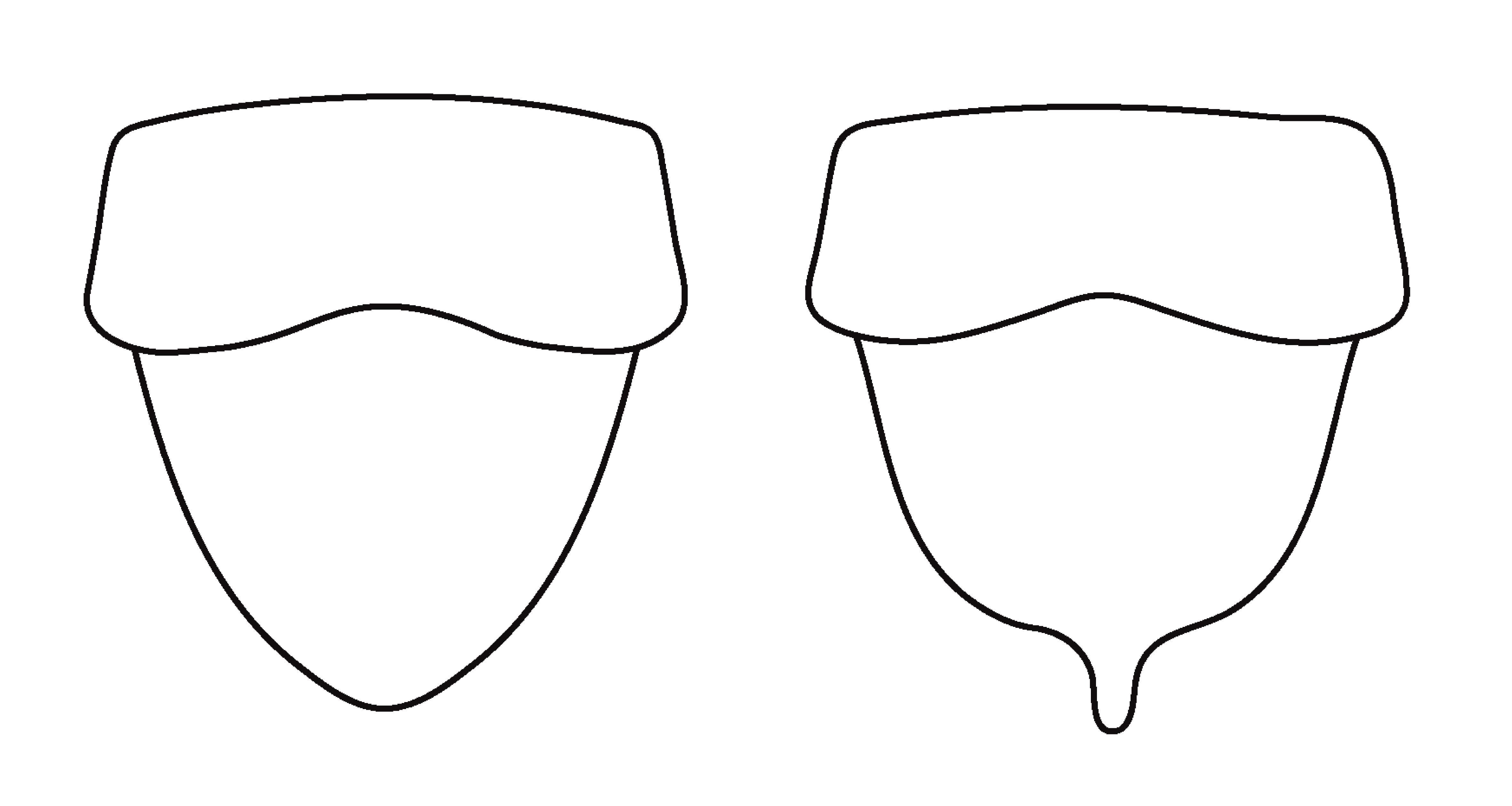

Supplement: Supplementary material 1 — Species data for genus Sphex [file zookeys-521-001-s001.zip › SphexDeltaFiles/Images/sternite_curvature.jpg]

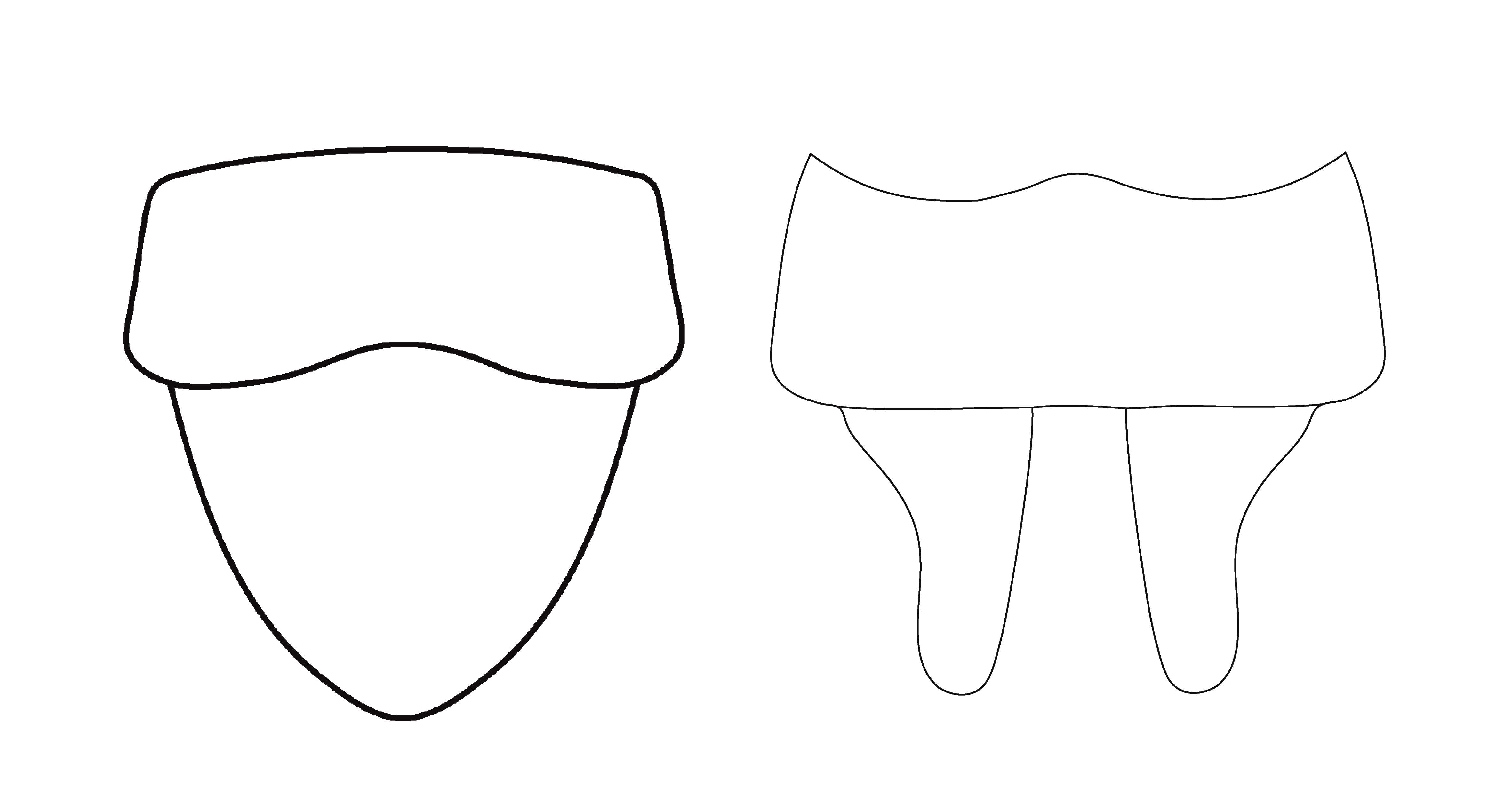

Supplement: Supplementary material 1 — Species data for genus Sphex [file zookeys-521-001-s001.zip › SphexDeltaFiles/Images/sternite_general.jpg]

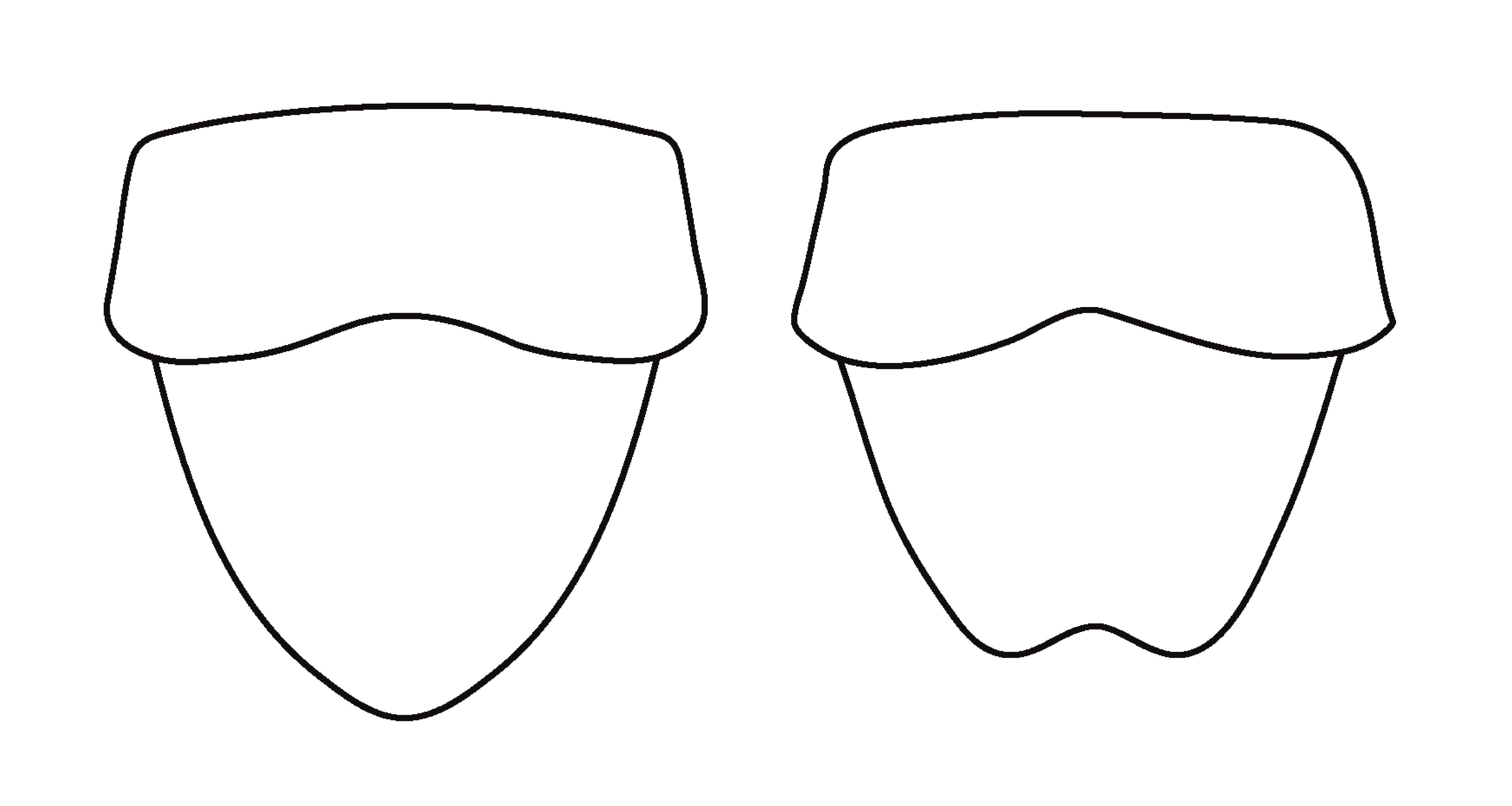

Supplement: Supplementary material 1 — Species data for genus Sphex [file zookeys-521-001-s001.zip › SphexDeltaFiles/Images/sternite_notched.jpg]

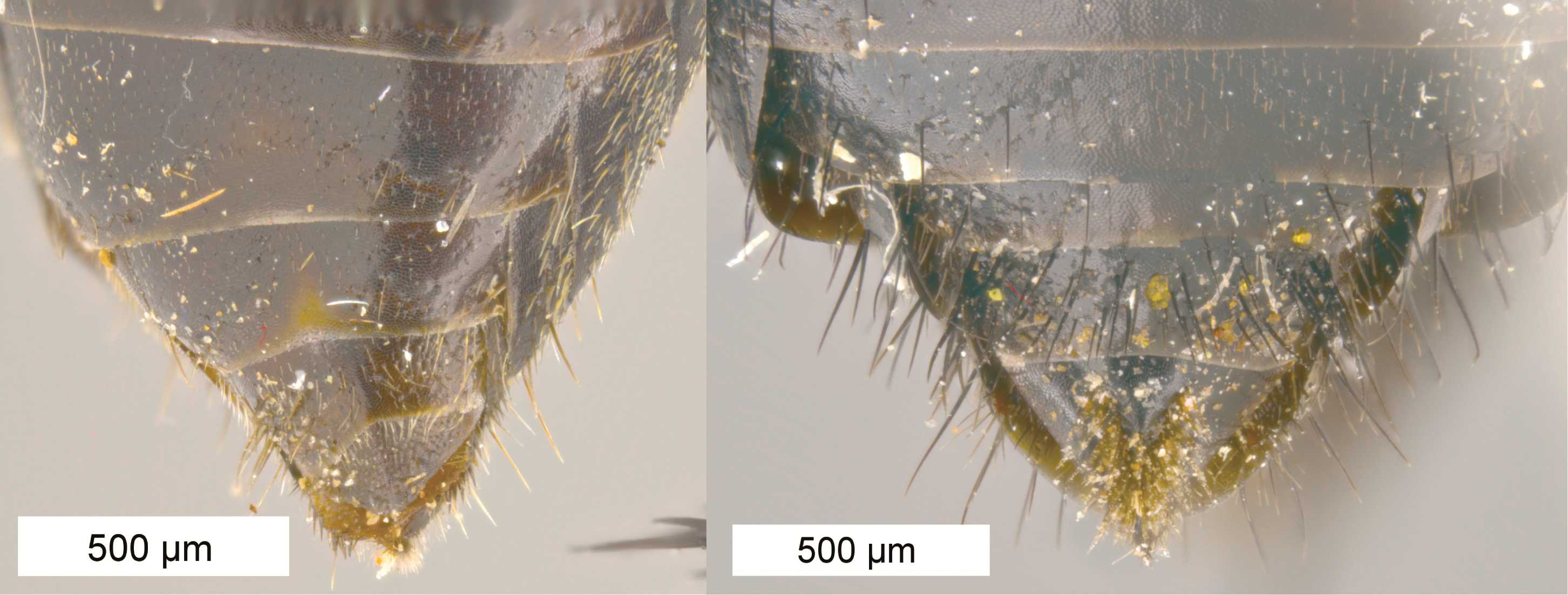

Supplement: Supplementary material 1 — Species data for genus Sphex [file zookeys-521-001-s001.zip › SphexDeltaFiles/Images/sternite_VIII_pubescence.jpg]

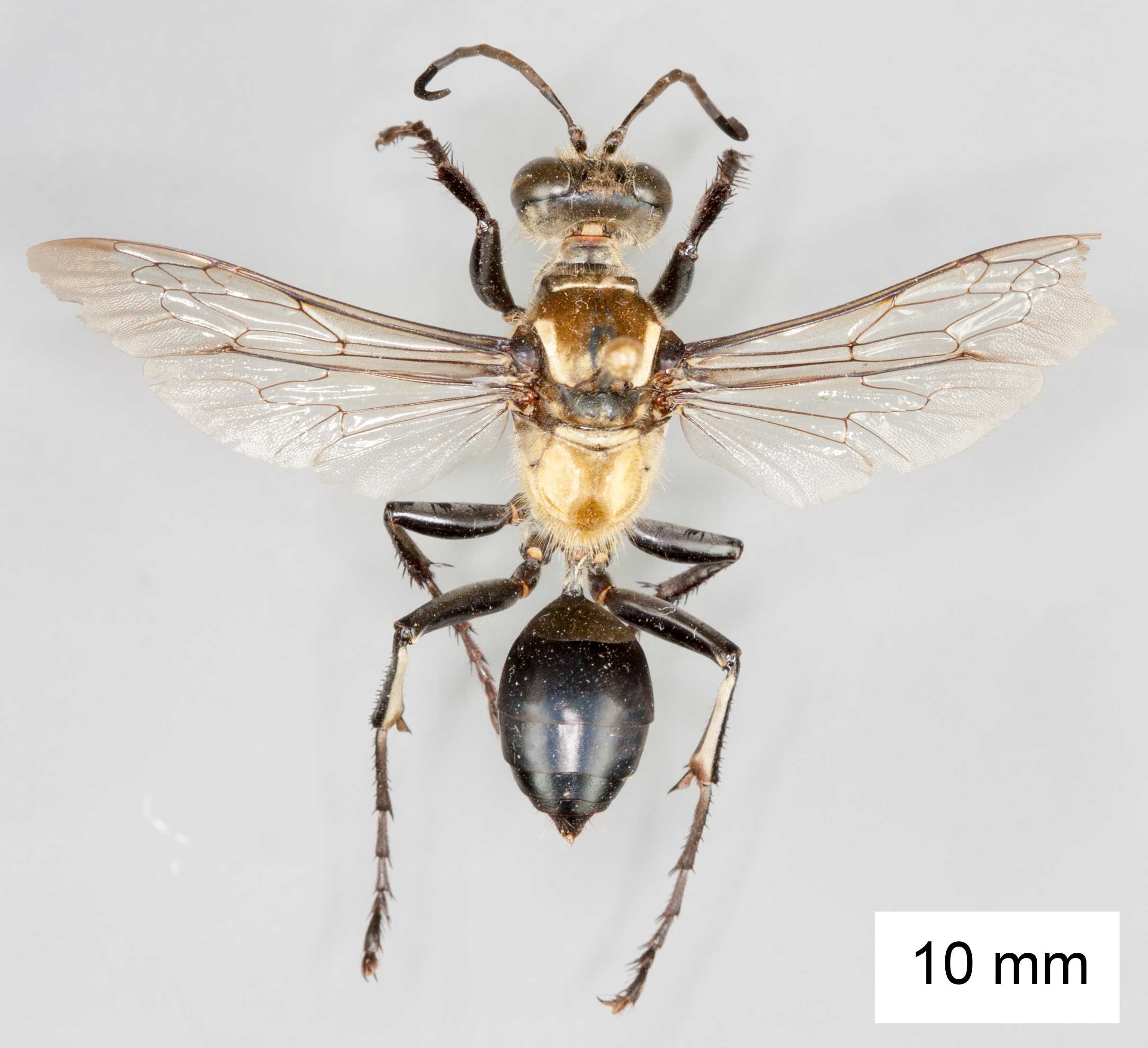

Supplement: Supplementary material 1 — Species data for genus Sphex [file zookeys-521-001-s001.zip › SphexDeltaFiles/Images/vestitus_f.jpg]

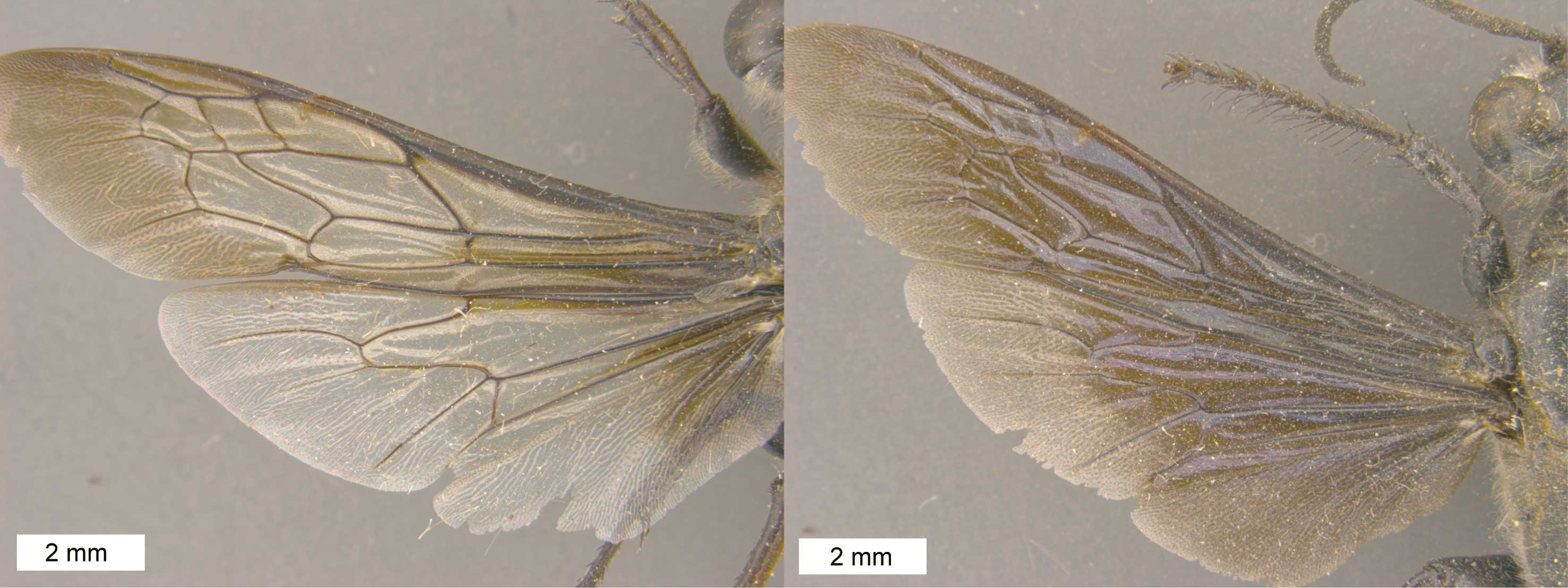

Supplement: Supplementary material 1 — Species data for genus Sphex [file zookeys-521-001-s001.zip › SphexDeltaFiles/Images/wing_darkening.jpg]

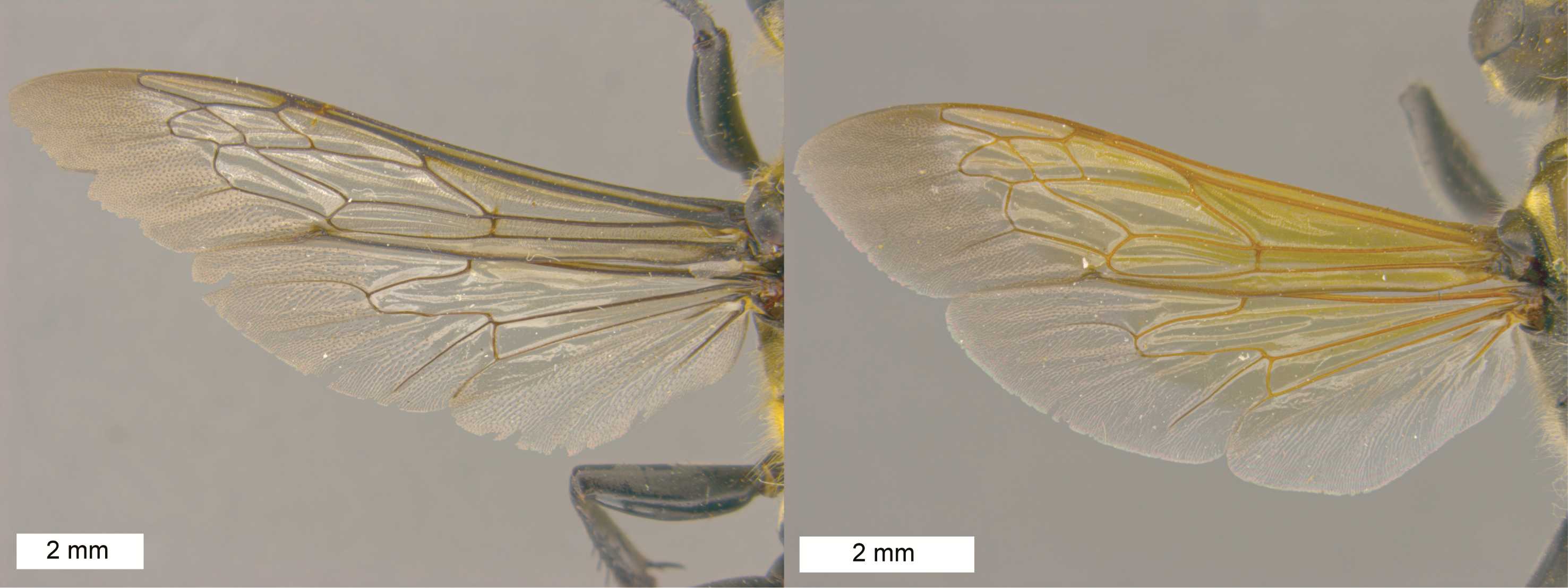

Supplement: Supplementary material 1 — Species data for genus Sphex [file zookeys-521-001-s001.zip › SphexDeltaFiles/Images/wing_with_yellow_tinge.jpg]
